# ﻿Lepidoptera of North America, north of Mexico: an annotated list containing geographic ranges and host-plant records

**DOI:** 10.3897/zookeys.1261.160796

**Published:** 2025-11-26

**Authors:** Kimberley J. Shropshire, Douglas W. Tallamy

**Affiliations:** 1 Department of Entomology & Wildlife Ecology, University of Delaware, 531 South College Avenue, Newark, DE 19716, USA University of Delaware Newark United States of America

**Keywords:** Butterfly, Canada, Lepidoptera, moth, skipper, UDELep, United States

## Abstract

We provide a list of all named Lepidoptera in the USA and Canada. Data include ranges, host plants, and synonymies. Information is annotated, including detailed range notes and host-plant records. Data are also provided in unique, easily filtered fields. The list establishes 12,541 native species, 325 exotic species, and 189 species straying occasionally into North America. In addition, we list 146 known but undescribed species, 112 species with uncertain or unresolved status, and 450 excluded species. 7423 of the described species in North America have host record data. Information is presented here as an Excel spreadsheet.

## ﻿Introduction

For 15-plus years we have been compiling lists of North American Lepidoptera and their host plants. The original impetus was a very simple question. Is there a way to compare the contributions of native and non-native plant genera to terrestrial food webs the Mid-Atlantic States of North America? We chose Lepidoptera because they are a preferred food of birds (Tallamy and Shriver 2021), transfer more energy from plants to animals than any other plant eaters (Janzen 1988), and have better host-plant records than other insect herbivores (Robinson et al. 2002). The results of this study were published (Tallamy and Shropshire 2009) and attracted enough interest to encourage us to expand our question beyond the Mid-Atlantic to all North America north of Mexico. Although we continued to hone this list, it was instrumental in determining that a small percentage of native plant genera contribute most of the Lepidoptera energy to food webs, regardless of latitude or bioregion (Narango et al. 2020). Here we present our most current list in a flexible version in Excel described in detail below (Suppl. material 1). Updates to the list will be available on University of Delaware’s Institutional Repository (https://guides.lib.udel.edu/udspace/home).

The information we provide includes searching and filtering fields of species name, genus name, family, subfamily, Hodges# established by Hodges (1983), MONA# Moths of North America number established by Pohl and Nanz (2023), geographic ranges, original combination, synonyms, subspecies, common name, exotic/native status, size, higher common categories, and caterpillar host records, including plants, insects, detritivores, fungi, mosses, lichens, and algae. All data are supported by citations. We do not attempt to be the final authority on over 13,000 species of Lepidoptera; our goal is to present a transparent compilation of the information we gathered which we believe is the current state of published knowledge on Lepidoptera and their host plant affiliations in North America.

A complete published list of all Lepidoptera in North America north of Mexico heretofore existed only as check lists (Hodges 1983; Poole 1996; Pohl et al. 2016; Pohl and Nanz 2023). Several books and guides promise comprehensive coverage (e.g. Holland 1934; Covell 1984; Beadle and Leckie 2012), but these understandably limit coverage mostly to common species. Useful websites such as Moth Photographers Group (http://mothphotographersgroup.msstate.edu) and Butterflies and Moths of North America, https://www.butterfliesandmoths.org/ (Lotts and Naberhaus 2025) are good sources, but they lack a mechanism to sort in multiple fields. Another important source, HOSTS - a Database of the World’s Lepidopteran Hostplants (Robinson et al. 2023), is a useful site for initial host-record research, but does not contain data tracing, uses continental-level range information, and does not always use current or consistent nomenclature. We note that our list is not a compilation of on-line sources; it is literature-based data. We do not envision our Lepidoptera list as a one-stop source, but rather a research tool to be used in concert with other resources.

## ﻿Methods

Our Lepidoptera list has gone through several iterations. The first was restricted to the Mid-Atlantic States of North America and relied heavily on data compiled by Robinson et al. (2002) (Tallamy and Shropshire 2009). The next major expansion was to the contiguous USA and Alaska for the National Wildlife Federation Native Plant Finder (www.nwf.org/nativeplantfinder/). Here, a list of North American Lepidoptera was compiled by merging lists from multiple sources (e.g. revisions, reviews, catalogues, fascicles, checklists) being careful not to duplicate entries under different names. Ranges, including Canadian, were assigned to each species and host plants researched. New host plant research was mostly focused on recent literature (post-2000).

The National Wildlife Federation update is based on USDA Plants nomenclature (https://plants.usda.gov/). The third major revamping of the list updated host plant data to modern nomenclature using The Biota of North America Program (hereafter noted as BONAP) (Kartesz 2015). This enabled us to define plant ranges by U.S. county using BONAP cross referenced with our Lepidoptera ranges (Narango et al. 2020). Old terminology was retained by placing the older names in brackets adjacent to the current names (e.g. Adoxaceae [Caprifoliaceae] *Schoenoplectus* [= *Bolboschoenus*], *Acmispon* [= *Lotus*]). This serves the dual purpose of eliminating redundant research and facilitating updates to the National Wildlife Federation list. Plants species not listed by BONAP follow Kew Royal Botanic Gardens’ Plants of the World Online nomenclature (KEW 2025).

Recent publications facilitated updating the list by including Canadian ranges (Pohl et al. 2018), incorporating Hodges numbers (Hodges 1983), P3 numbers (Pohl et al. 2016), and new MONA numbers (Pohl and Nanz 2023), and by completing North American synonymies (Pohl et al. 2016). Subspecies are listed in synonymy. Our records do not align exactly with Pohl and Nanz (2023) counts because we have included or reassigned some previously omitted and questioned records. The addition of new species and recent taxonomic reassignments have also affected the count. Not all records we choose to include on our list have been assigned a MONA number.

When possible, we have included Lepidoptera body size in our lists. Data for size are given in wingspan, total length, and forewing length. Size ranges incorporate both male and female sizes; average sizes are noted as such. We recognize that body size can vary by season and/or region.

More recent publications of new host plant records have been sought out and edited to current taxonomical usage regardless of publication date. Information lacking from recent 2024 and 2025 papers will be added in updates. Butterfly and skipper range data have been expanded beyond a few guides.

### ﻿Lepidoptera species

Our primary goal has been to create a list of North American Lepidoptera ranges and their corresponding host plant genera. Lepidopteran taxonomical updates rely on recent peer-reviewed publications. Our generic names of Lepidoptera may not conform to all published opinions, but our core range and host plant data for each species remains consistent. All names can be found by a search using the species name only or an expanded name search that includes synonyms. It is not our intent to resolve disputed usage or to establish proper current nomenclature.

Our list is in alphabetical order. The list can be filtered by butterflies, skippers, macromoths, micromoths, family, and subfamily. If taxonomic order is desired, the user can re-order the list by using the Hodges or MONA numbers. The species names follow various authors; as a result, proper species gender or suffix is not always clear. Routinely searching by a truncated species name may be the most successful search method.

Complications can occur when revisions contract or split a species. Every effort has been made to follow data to the logical conclusion and clearly attach records of former species to current usage. Not all species designations are static; suggested and accepted usage has been changed several times for some groups. Additionally, more often than not, DNA analyses identify new species. Some species, long accepted as Holarctic, have been separated from their European cousins (Gilligan et al. 2020). In a very few cases, the resulting new species have not been properly named but are retained on our list as near (nr.). Taxonomical updates are clearly attributed to the author(s). We note that the format we use is flexible enough to easily incorporate changes in nomenclature.

The common names column is not limited to currently accepted nomenclature. This column indiscriminately includes many common names which can be useful when tracking older lists. As a result, some common names may be linked to more than one species.

### ﻿Lepidoptera ranges

When possible, our primary source of range information is the most recent comprehensive publication. We started with current records and worked backwards. However, many species lack recent work, and, in a few cases, we needed to go back to the original source. Information was established by citing published literature including generic revisions, reviews, state lists, regional lists, guides, articles, compilations, and record lists. The resulting reference list contains some 4450 sources (Appendix 1). Occasionally, we cite our own personal observations. Some ranges are clarified by citing records such as Symbiota Collections of Arthropods Network (https://scan-bugs.org/portal/collections) and Bold Systems (Ratnasingham and Hebert 2007). We avoided unpublished records and unsupported information. Miscellaneous information found on the internet was researched but not included if we could not trace it to a citable source.

Ranges of Lepidoptera were created from states and provinces citations. A summary of the range is to the right of the equal sign (=) or a notation if parts of range are inferred (= infer:). Ranges compiled from a few sources occasionally skip over states (e.g. = OH AR NC), while a stated range will encompass all logical inclusions (e.g. in mountains from New York to Georgia = infer: NY PA NJ MD WV VA SC NC GA). Logical ranges often are supported and supplemented by several other references. In cases of conflict, the latest authority has precedence and is noted as such (defer to). Some reports are stated unlikely or seem so; these records are listed as fugitive and will appear in an expanded search.

The comprehensive compilation of cited sources of ranges is contained in a single column in our spreadsheet. Another column contains easily filtered and searched state and province abbreviations. Two more columns offer the option to search regions within the contiguous U.S. Excel enables filtering for the same field for two discreet codes. This makes it possible to confine searches to a single state or two states, or to expand searches to one or two regions. A fourth column notes Holarctic species and a partial list of Lepidoptera with Neotropical records. A fifth column can sort between exotic and native species. For example, a user could search for native, Holarctic Lepidoptera in Wisconsin and Michigan.

Lepidoptera range searches will return records that include 1) residents (those that overwinter) and 2) migrants (those that regularly appear and reproduce) but not strays, which are sporadic or rare visitors that offer no eco-services beyond pollination, nor fugitives, which are records that are unverified or in doubt for described reasons. There is a column expanding searchable established ranges to include stray and fugitive records. The words stray and fugitive do not contain any state nor province abbreviation to allow for inclusion in the search field.

Exotic and native Lepidoptera species can be filtered in a column. The exotic designation does not differentiate between accidental introductions and biological control introductions. The exotic status of a species is also identified at the beginning of the comprehensive justification of range (e.g. <EXOTIC> or <EXOTIC>. BIOCONTROL). Lepidoptera are considered exotic if there is a valid citation indicating that the species was introduced into North America. An expanded range of a North American species into new ranges is not consider exotic, but this information is noted as well. Neotropical strays into southern states are generally not consider exotics.

Excluded species are those Lepidoptera that have at some point been erroneously included on North American lists. Many are former usages that are still valid elsewhere. Some records represent misidentifications of species or locations. The reason for exclusion is noted and cited. Unconfirmed species are Lepidoptera that appear on current North American lists, but we have been unable to establish or exclude a North American range. Unconfirmed species and excluded species will not appear on any range search but will create a return in the strays and fugitive column. Host plant records of unconfirmed species will create a return in a search. The possibility of the presence of unconfirmed species in North America remains; these were not excluded.

The list of known but undescribed species is by no means a comprehensive list. These records will only be returned in a generic search and, occasionally, a host plant search. Although unnamed species have known locations, they will not be included in the discreet range columns until their status is established. It is possible some of these species also appear as recently described species.

Type location is given for many species. Unfortunately, original formats weighed type as a location record resulting a designation that might include holotypes, paratypes, syntypes, allotype, and synonymic types. While not incorrect, we note the possibility of confusion here and continue to update.

### ﻿Host-plant records

Host-plant records can be filtered by plant family or genus. These records are listed in three columns with a fourth containing appropriate citations.

Host-plant data were originally recorded only at genus level. This decision was based on the best available information. To illustrate, oaks (*Quercus*) had all host plants recorded to species whenever possible. However, of the 1014 Lepidoptera recorded on *Quercus*, 728 include a genus-only (*Quercus* sp.) records, and 263 include only a genus-level record. For our purposes the abbreviation sp. is used for unspecified species (example: *Quercus* sp. = oaks, red oak family, *Quercus* ssp., *Quercus* sp., or unidentified oak species). In addition, about 150 plant genus records required research for proper assignment to the current new genus. Of the 2068 lepidopteran species (comprising 73 families) which have been completely researched for host plant species, over 68% contain a record of a genus with an unspecified plant species. Fairly late in the process, we began to routinely include species records when they existed. Partial species level information can be found in the host plant reference column. Nevertheless, host genus remains the primary metric.

Some host plant genus names are embedded in another genus name. There is a column that creates discreet codes by inserting a period before and after the host plant (.*Pinus*.), eliminating false returns such as *Lupinus* (lupines) in a search for *Pinus* (pines). Another column offers the opportunity to return former generic usage excluded from the first search (*Symphyotrichum*[=*Aster*]). We established current uses for plant species nomenclature by using BONAP or, occasionally, for non-North American, plants Kew Plants of the World Online (https://powo.science.kew.org). We include records from beyond North America to paint a broader picture of Lepidoptera host ranges. These records are generally noted as such.

We have made every effort to clarify host plant genera to current usage using BONAP by tracing records at the species level. We provide a list of reexamined plant genera and reassignment explanations (Appendix 2). Some North American genus records use non-North American names; they remain unresolved (examples: *Acacia*[unresolved], *Aster*[unresolved], and *Eupatorium*[unresolved]. If there is a logical alternative, we include the alternate native genus in the search field (example: .*Symphyotrichum*.[unassigned*Aster*]). In this way the native host plant will be returned in a search. When there is no good alternative, the original host genus remains (example: .*Eupatorium*.[unassigned]). This treatment clearly maintains the progression of data and a user can choose to exclude unassigned genus.

Although we have corrected plant species names as described above, not all valid stand-alone generic host plant records are correct. For example, our records report 183 Lepidoptera with *Pyrus* (pear) as a host plant, but many of these citations pre-date reassignment of some *Pyrus* to *Sorbus* (mountain-ash), *Aronia* (chokeberry), and *Malus* (apple). Only 14 Lepidoptera with *Pyrus* records do not also contain a potential reassignment. Despite the potential for misreporting, records for *Pyrus* remain because we cannot confidently justify another assignment.

Efforts have been made to correct misinformation. Host plant records can create unresolvable conflicts. A Lepidoptera species may have host plant preferences that vary by region or subspecies (Tallamy et al. 2010). For example, one author may state that a Lepidoptera species is a specialist on one plant in Arkansas while another author claims it is a specialist on a different plant in Maryland. Both can be correct due to regional specialization. We avoid eliminating records unless we can cite a clear denial that covers all of North America. Occasionally, questionable records are noted but are still included in our records. Adult plant associations are not included in host plant data.

Host-plant genera are listed alphabetically within the search fields. Records for host plants do not include some redundant citations. Weight cannot be applied to more frequent citations, as authors could be repeating each other. The earliest version of our plant list relied heavily on Robinson et al. (2002). Host plants in it were listed in one field while citations without specifics were listed in another. In many cases, this system persists. We are confident the host-plant data we present are traceable to the sources in the host plant references field.

### ﻿Notes

Notes and clarifications made by us are in brackets. Brackets contain clarifications such as limitations of stated ranges, the taxonomical nomenclature used in the citation, and attribution of taxonomical updates. Data were assembled objectively; however, not all authors agree. Very rarely, we needed to arbitrate differences. Notes justifying our decisions and discussions noting discrepancies are also in brackets. Our goal is to report data as transparently as possible.

Parting with accepted use, we use no abbreviations except for states and provinces. This eliminates possible confusion, especially with binomial names and enables more specific searches. Synonymic lists contain no abbreviations. Additionally, synonymic lists can contain former combinations. The original combination is in a discreet column.

## ﻿Results

Our list establishes the following records in USA and Canada. There are 12546 native Lepidoptera species, 324 exotic species, and 183 species straying occasionally into these countries. In addition, we list 111 species with uncertain or unresolved status, 142 known but undescribed species, and 452 excluded species. There are 7424 described species with some host record. The spread sheet with details is contained in Suppl. material 1. Family counts are listed in Table 1.

**Table 1.** Number of species of Lepidoptera per family in Canada and United States. Native species are known to occur naturally. Exotic species are cited as being introduced. Stray species are those species that occur periodically but are not known to establish a permanent population. Unresolved species have been reported in our range, but insufficient citations exist to establish range.

Family	Native residents	Exotic residents	Strays	Unresolved	Total
Acanthopteroctetidae	4	0	0	0	4
Acrolepiidae	10	1	0	0	11
Acrolophidae	67	0	0	1	68
Adelidae	30	0	0	0	30
Alucitidae	5	0	0	0	5
Apatelodidae	6	0	0	0	6
Argyresthiidae	52	2	0	0	54
Attevidae	2	0	0	0	2
Autostichidae	11	3	0	0	14
Batrachedridae	23	1	0	0	24
Bedelliidae	1	1	0	0	2
Blastobasidae	69	2	0	2	73
Bucculatricidae	102	0	0	3	105
Carposinidae	12	0	0	0	12
Choreutidae	49	3	0	0	52
Coleophoridae	137	13	0	3	153
Copromorphidae	5	0	0	0	5
Cosmopterigidae	183	1	0	2	186
Cossidae	47	1	0	0	48
Crambidae	842	31	4	10	887
Dalceridae	1	0	0	0	1
Depressariidae	195	9	0	0	204
Doidae	3	0	0	0	3
Douglasiidae	10	0	0	0	10
Drepanidae	20	0	0	0	20
Dryadaulidae	2	1	0	0	3
Dudgeoneidae	1	0	0	0	1
Elachistidae	150	4	0	1	155
Epermeniidae	12	0	0	0	12
Epimarptidae	1	0	0	0	1
Epipyropidae	1	0	0	0	1
Erebidae	977	11	10	8	1006
Eriocraniidae	13	0	0	0	13
Euteliidae	19	0	0	0	19
Galacticidae	0	1	0	0	1
Gelechiidae	872	27	0	9	908
Geometridae	1426	12	0	8	1446
Glyphidoceridae	10	0	0	0	10
Glyphipterigidae	40	1	0	0	41
Gracillariidae	303	9	0	4	316
Heliodinidae	33	0	0	0	33
Heliozelidae	30	0	0	0	30
Hepialidae	18	1	0	0	19
Hesperiidae	267	2	65	10	344
Hyblaeidae	1	0	0	0	1
Incurvariidae	6	0	0	0	6
Lacturidae	6	0	0	0	6
Lasiocampidae	33	0	0	0	33
Lecithoceridae	1	2	0	0	3
Limacodidae	46	0	1	0	47
Lycaenidae	156	1	21	3	181
Lyonetiidae	18	3	0	0	21
Lypusidae	0	1	0	0	1
Meessiidae	3	0	0	0	3
Megalopygidae	11	0	0	1	12
Micropterigidae	3	0	0	0	3
Millieriidae	1	0	0	0	1
Mimallonidae	5	0	0	0	5
Momphidae	42	5	0	0	47
Nepticulidae	89	9	0	0	98
Noctuidae	2513	24	1	6	2544
Nolidae	38	2	0	0	40
Notodontidae	179	0	0	1	180
Nymphalidae	212	2	36	10	260
Oecophoridae	30	9	0	1	40
Opostegidae	10	0	0	0	10
Papilionidae	29	1	11	2	43
Pieridae	72	1	10	0	83
Plutellidae	16	1	0	0	17
Praydidae	4	1	0	0	5
Prodoxidae	64	1	0	1	66
Psychidae	22	4	0	1	27
Pterolonchidae	15	1	0	0	16
Pterophoridae	166	3	0	0	169
Pyralidae	654	24	0	4	682
Riodinidae	24	0	5	0	29
Saturniidae	88	1	1	1	91
Schreckensteiniidae	3	0	0	0	3
Scythrididae	41	2	0	0	43
Sematuridae	2	0	0	0	2
Sesiidae	135	4	1	1	141
Sphingidae	117	2	14	3	136
Stathmopodidae	3	0	0	2	5
Thyrididae	13	0	0	0	13
Tineidae	102	24	0	0	126
Tischeriidae	49	0	0	0	49
Tortricidae	1384	47	1	12	1444
Tridentaformidae	1	0	0	0	1
Uraniidae	9	0	2	1	12
Urodidae	2	0	0	0	2
Yponomeutidae	17	9	0	0	26
Ypsolophidae	27	2	0	0	29
Zygaenidae	24	1	0	1	26
Unplaced Apoditrysia [formerly in Yponomeutidae]	1	0	0	0	1
Totals	12548	324	183	112	13167

## ﻿Discussion

Creating a list for public consumption was not our original goal, but the Excel format has been a constant. Thus, search functions required establishing discreet usage and workarounds for conflicts. Occasionally, we depart from convention, in particular, lack of commas and italics in some fields. Each updated list has required some functionality changes. Older formatting and usages have been corrected when found, but, undoubtedly, a few remain. We will continue to maintain our list, correcting errors as we discover them.

Host plant associations have been the core of our research. For example, we have shown through our research that plants vary widely in their ability to host Lepidoptera and thus support food webs (Narango et al. 2020). Moreover, not all plant species have been equally studied for host relationships. Plants associated with agriculture or lumber factor disproportionally on our lists. Humans have spent thousands of years selecting plants to be more palatable, and then have carefully inventoried all insect pests, such a cataloged by Zhang (1994). Other examples include apples (Chapman and Lienk 1971), grapes (McGiffen and Neunzig 1985), tomatoes (Okumura 1974), and conifers (Maier et al. 2011). Our list has non-native *Glycine* (soybeans) as a host plant for 67 native Lepidoptera species. Caterpillars of these species are also recorded on at least two native plant genera, and, on average, eat 32 other plant genera. All exotic host plant records are listed, but readers should be cognizant that economically important plant records are likely skewed simply because they have received more attention.

Other host-plant issues warrant mention. Some records may be the result of incidental feeding. Others may be from captive trials. Lab rearing often does not reflect the reality of natural host associations. Techniques that offer gravid females oviposition choices may not reveal actual host use in the field. A caterpillar found wandering on the ground may be offered nearby plants, or perhaps *Lactuca* (lettuce) or *Plantago* (plantain). Records like these may make some Lepidoptera species appear to have a broader host range than they actually do. Many records will contain clarifying information within the citation, such as captive reared, tropical records, European records, and oviposition records.

A search for a host plant will return all records. Not all records apply to a particular Lepidoptera species everywhere, as every acceptable host plant may not occur in all parts of that species’ range. For our research, we used BONAP to create regional plant lists to county; at this point, it is not possible to use our Lepidoptera data sheet to confine searches to plant ranges.

In general, our host plant data include all of the observed host records that we have found, as well as nearly 500 non-plant host records. Despite some shortcomings, the over 30,000 plant genus–Lepidoptera species records we have cited represent the best state of current knowledge concerning Lepidoptera host-plant associations in North America. Furthermore, because we cite sources, data for individual Lepidoptera can be interpreted by users more easily than in other sources.

The minimal searchable range section of our list is to state and province. One obvious constraint is that Lepidoptera ranges do not conform to political boarders nor do states and counties lie completely within an Ecological Region (Ecological Regions of North America 2024). Some cited ranges specify geological features, such as mountain ranges or a portion of a state. With over 3000 counties in the U.S., a comprehensive list with more specific ranges is beyond the scope of this project.

Our list makes no attempt at taxonomic identification. However, our list can facilitate identification of Lepidoptera adults and caterpillars by filtering by family, range, and host plant. Honing in on fewer options makes image searches more manageable.

We have assembled the most comprehensive compilation of North American Lepidoptera data in its entirety released to date. Because this project will never be done, we present it here as a work in progress. Data gathering is an ongoing effort, and new data are periodically uploaded to University of Delaware’s Institutional Repository. Efforts are underway to format our data into an easily navigated website and to a PDF format.

Updates to appear in University of Delaware’s Institutional Repository, https://guides.lib.udel.edu/udspace/home.

## ﻿Additional information

### Use of AI

No use of AI was reported.

### Author ORCIDs

Kimberley J. Shropshire https://orcid.org/0000-0002-6236-370X

Douglas W. Tallamy https://orcid.org/0000-0002-9207-6293

### Data availability

All of the data that support the findings of this study are available in the main text or Supplementary Information.

## Supplementary materials

Supplementary material 1 An annotated list Lepidoptera of USA & Canada with ranges & host records Data type xlsx Explanation note An excel spreadsheet containing multiple filterable fields. https://binary.pensoft.net/file/1478226This dataset is made available under the Open Database License (http://opendatacommons.org/licenses/odbl/1.0/). The Open Database License (ODbL) is a license agreement intended to allow users to freely share, modify, and use this Dataset while maintaining this same freedom for others, provided that the original source and author(s) are credited.Kimberley J. Shropshire, Douglas W. Tallamy

Supplementary material 2 Reference list for Lepidoptera of North American north of Mexico: An annotated list containing geographic ranges and host plant records (May 2025 version) Data type doc Explanation note 4485 references correspond to annotations in excel sheet Lepidoptera of North American north of Mexico: An annotated list containing geographic ranges and host plant records. https://binary.pensoft.net/file/1478227This dataset is made available under the Open Database License (http://opendatacommons.org/licenses/odbl/1.0/). The Open Database License (ODbL) is a license agreement intended to allow users to freely share, modify, and use this Dataset while maintaining this same freedom for others, provided that the original source and author(s) are credited.Kimberley J. Shropshire, Douglas W. Tallamy

Supplementary material 3 Species-level examinations to catagorize host plant records into proper genus Data type pdf Explanation note Taxonomic revisions reclassified these genera. Species-level examination of records was required. These tables help clarify the plant species that required in depth inspection to find current nomenclature of host plant genus. Searches were confined to host plant records. https://binary.pensoft.net/file/1478228This dataset is made available under the Open Database License (http://opendatacommons.org/licenses/odbl/1.0/). The Open Database License (ODbL) is a license agreement intended to allow users to freely share, modify, and use this Dataset while maintaining this same freedom for others, provided that the original source and author(s) are credited.Kimberley J. Shropshire, Douglas W. Tallamy
