# Supplementary material for: ﻿Lepidoptera of North America, north of Mexico: an annotated list containing geographic ranges and host-plant records
Source: Zookeys. 2025 Nov 26;1261:101–13. doi: 10.3897/zookeys.1261.160796 (PMC12676573; doi:10.3897/zookeys.1261.160796)
Supplement: Supplementary material 2 — Reference list for Lepidoptera of North American north of Mexico: An annotated list containing geographic ranges and host plant records (May 2025 version) [file zookeys-1261-101_article-160796__-s002.doc]

AUG 2025 Lepidoptera reference list

[REFERENCE list is also incorporated as a tab of UDELep excel sheet]

Aarvik, L. 1987. Contribution to the knowledge of the Norwegian Lepidoptera 11. Fauna Norvegica Ser. B 34(1): 7-13.

Aarvik, L., B.Å. Bengtsson, H. Elven, P. Ivinskis, U. Jürivete, O. Karsholt, M. Mutanen, and N. Savenkov. 2017. Nordic-Baltic Checklist of Lepidoptera. Norwegian Journal of Entomology. Supplement 3: 1-236.

Abbot, J. and J.E. Smith. 1797. The natural history of the rarer lepidopterous insects of Georgia: including their systematic characters, the particulars of their several metamorphoses, and the plants on which they feed. Collected from the observation of Mr. John Abbot, many years resident in that country. T. Bensley, London.

Abdelfattah, M.A. Salem. 2020. Revision of family Noctuidae of Egypt (3) Subfamilies “Acontiinae, Agaristinae, Amphipyrinae and Hadeninae” (Lepidoptera, Noctuidae). Egyptian Academic Journal of Biological Sciences A. Entomology 13(1): 59-88.

Abreu, J.M. de. 1968. Título: Problemas entomológicos da cacauicultura no Espiritu Santo. Turrialba 18(2): 182-186.

Adaime, R., R. Souza Santos, A. Lopes Lima, J.E. Veloso dos Santos, and A. Specht. 2020. First record of Chilomima clarkei (Amsel)(Lepidoptera: Crambidae) in Manihot esculenta Crantz (Euphorbiaceae) in Amapá state, Brazil. EntomoBrasilis 13: e914; 1-4.

Adamczewski, S. 1947. Notes on the Lepidoptera of Poland. Entomologist 80: 102-106, 133-136.

Adamczewski, S. 1951. On the systematic and origin of the generic group *Oxyptilus* Zeller (Lep. Alucitidae). Bulletin of the British Museum, Natural History (Entomology) 1: 301-388.

Adams, J.K. 1983. An old first United States record finally published: *Papilio victorinus* (Papilionidae) in Laredo, Texas. Journal of the Lepidopterists' Society 37: 318-318.

Adams, J.K. 2000. State coordinator reports, Georgia. News of Southern Lepidopterists' Society 22(2) 37-40.

Adams, J.K.2003. *Bagisara laverna* from southeastern Arizona: a new United States record (Lepidoptera: Noctuidae: Bagisarinae). Holarctic Lepidoptera 7(2): 48

Adams, J.K. 2006. Moths and Butterflies of Georgia and the Southeastern United States. www.daltonstate.edu/galeps/index.htm. Dalton State College, Dalton, GA.

Adams, J.K. 2013. Georgia Lepidoptera - Moths and Butterflies of Georgia and the Southeastern United States. https://dscweb.daltonstate.edu/galeps/ [accessed 30-Apr-2014]

Adams, J.K. 2019. From the Editor’s Desk. News of the Lepidopterists’ Society 61(3): 125.

Adams, J.K. and J.D. Lafontaine. 2009. A new species of *Plagiomimicus* Grote (Noctuidae: Stiriinae) from northern Arizona and southeastern Utah. Journal of the Lepidopterists’ Society 63(3): 173-176.

Adams, J.K. and B.C. Schmidt. 2018. A new species of *Sympistis* Hübner from Sapelo Island, Georgia, USA (Lepidoptera, Noctuidae, Oncocnemidinae). In: B.C. Schmidt and J.D. Lafontaine. (Eds.). Contributions to the systematics of New World macro-moths VII. ZooKey 788: 79-86. https://doi.org/10.3897/zookeys.788.26484

Adams, M.S. 2001. A revision of the moth genus *Leucania* Ochsenheimer in the Antilles (Insecta: Lepidoptera: Noctuidae). Annals of Carnegie Museum 70(3): 179-220.

Adamski, D. 1990. Tineidae *in* primary types of Microlepidoptera in the Museum of Comparative Zoology (with a discussion of V.T. Chambers’ work). In: S.E. Miller and R.W. Hodges (eds.). Bulletin of the Museum of Comparative Zoology 152(2): 45-87.

Adamski, D. 1998. On the identity of *Holcocera* Guilandinae (Busck 1900) (Lepidoptera: Gelechioidea: Coleophoridae: Blastobasinae). Proceedings of the Entomological Society of Washington 100: 731-741.

Adamski, D. 1999. *Blastobasis graminea*, new species (Lepidoptera: Gelechioidea: Coleophoridae: Blastobasinae), a stem borer of sugar cane in Colombia and Venezuela. Proceeding of the Entomological Society of Washington 101(1): 164-174.

Adamski, D. 2003a. A new North American *Calosima* (Lepidoptera: Coleophoridae: Blastobasinae). Tropical Lepidoptera Research 11(1-2): 46-48.

Adamski, D. 2003b. A new *Blastobasis* associated with a corns and pecans in the southeastern and Southcentral United States (Lepidoptera: Coleophoridae: Blastobasinae). Holarctic Lepidoptera 7(2): 51-53.

Adamski, D. 2004. A new *Holcocera* Clemens (Lepidoptera: Gelechioidea: Coleophoridae) from mountainous southeastern Arizona. Proceedings of the Entomological Society of Washington 106: 649-653.

Adamski, D. and R.L. Brown. 1987. A new Nearctic *Glyphidocera* with descriptions of all stages (Lepidoptera: Blastobasidae: Symmocinae). Proceedings of the Entomological Society of Washington 89(2): 329-343.

Adamski, D. and R.L. Brown. 1989 Morphology and systematics of North American Blastobasidae (Lepidoptera: Gelechioidea). Mississippi Agricultural and Forestry Experiment Station Technical Bulletin 165: 1-70.

Adamski, D. and M. Hoddle. 2009. A new *Holcocera* Clemens from Guatemala and redescription of *H. iceryaeella* (Riley) from the United States (Lepidoptera: Coleophoridae: Blastobasinae: Holcocerini): two congeners with incidental preference for Avocado. Proceedings of the Entomological Society of Washington 111(1): 254-262.

Adamski, D. and R.W. Hodges. 1996. An annotated list of North American Blastobasinae (Lepidoptera: Gelechioidea: Coleophoridae). Proceedings of the Entomological Society of Washington 98(4): 708-740.

Adamski, D. and E.H. LaGasa. 2009. Description of larva, adults, and bionomics of *Filatima demissae* Keifer (Lepidoptera: Gelechiidae) in Washington, USA. Entomological News 120(5): 453-463.

Adamski, D. and C.T. Maier. 2003. A new *Holcocera* Clemens (Lepidoptera: Gelechioidea: Coleophoridae: Holcocerini) associated with Pinaceae in North America. Proceedings of the Entomological Society of Washington 105(1): 144-148.

Adamski, D. and E.H. Metzler, 2000. A new species of *Glyphidocera* Walsingham from southwestern Ohio (Lepidoptera: Gelechioidea: Glyphidoceridae). Proceedings of the Entomological Society of Washington 102(2): 301-307.

Adamski, D. and O. Pellmyr. 2003. Redescription of *Blastobasis yuccaecolella* Dietz 1910 (Lepidoptera: Gelechioidea: Coleophoridae: Blastobasini), with observations on its biology. Proceedings of the Entomological Society of Washington 105(2): 388-396.

Adamski, D. and T.M. Peters, 1986. A review of Nearctic *Apotomis* Hübner (Lepidoptera: Tortricidae: Olethreutini). Canadian Entomologist 118: 649-689.

Adamski, D., J.W. Brown, and W.H. White. 2006. Description of the immature stages of *Pyroderces badia* Hodges (Lepidoptera: Cosmopterigidae) with a new host record from Louisiana. Proceedings of the Entomological Society of Washington 108: 341-346.

Adamski, D., G.E. Hevel, and A. Pultyniewicz. 2009a. Redescription and immature stages of *Promalactis suzukiella* (Matsumura) (Gelechioidea: Oecophoridae), a new introduction into the United States. Proceeding of the Entomological Society of Washington 11(1): 204-214.

Adamski, D., J.L. Apple, and J.G. Bishop. 2009b. A new *Filatima* Busck (Lepidoptera: Gelechiidae) associated with lupine and early herbivore colonization on Mount St. Helens. Proceedings of the Entomological Society of Washington 111(2): 293-304.

Adamski, D., P.J. Johnson, A.A. Boe, J. Bradshaw, and A. Pultyniewicz. 2010a. Descriptions of life-stages of *Blastobasis repartella* (Lepidoptera: Gelechioidea: Coleophoridae: Blastobasinae) and observations on its biology in switchgrass. Zootaxa 2656: 41-54.

Adamski, D., J.F. Landry, S. Passoa, and R.A. Tracy. 2010b. History, distribution, and identification of *Exoteleia dodecella* (L.) (Lepidoptera: Gelechiidae) in North America, with insights into the systematics of *Exoteleia* Wallengren using characters of the adult, immatures, bionomics, and DNA barcodes. Proceedings of the Entomological Society of Washington 112(2): 183-206.

Adamski, D., J.F. Landry, V. Nazari, and R.J. Priest. 2014. Three new species of leaf-mining Gelechiidae (Lepidoptera) from Canada and Northeastern United States. Journal of the Lepidopterists’ Society 68(2): 101-123.

Adamski, D., R. R. Kula, M. W. Gates, Javier Torrens, P. Fidalgo, and M. L. Buffington. 2018. Immature stages of *Scrobipalpula patagonica* Povolný, 1977 (Lepidoptera: Gelechiidae: Gnorimoschemini), a gall inquiline of *Suaeda divaricata* MOQ. (Amaranthaceae) in Argentinawith a summary of its parasitoids. Proceedings of the Entomological Society of Washington 120(4: 659-669.

Ades, G. and R.C. Kendrick. 2004. Hong Kong Fauna: A selected Checklist, 2nd Edition. Fauna Conservation Department, Kadoorie Farm and Botanica Garden Corporation.

Aditya, J.P., A. Bhartiya, R.K. Chahota, D. Joshi, N. Chandra, L. Kant, and A. Pattanayak. 2019. Ancient orphan legume horse gram: A potential food and forage crop of future. Planta 250: 891-909.

Adler, P.H. 1982. Soil- and puddle-visiting habits of moths. Journal of the Lepidopterists’ Society 36(3): 161-173.

Agassiz, D.J.L, 1997. Yponomeutidae (including Roeslerstammiidae) pp. 39-115 *in* A.M. Emmet (ed.), The Moths and Butterflies of Great Britain and Ireland, Volume 3, Yponomeutidae - Elachistidae. Harley Books, Colchester, Essex, England.

Agassiz, D. and J. Langmaid. 2004. The *Eucosma hohenwartiana* group of species (Tortricidae). Nota Lepidopterologica 27: 41-50.

Agnew, C.W., L. A. Rodriguez-del-Bosque, and J.W. Smith, Jr. 1988. Misidentification of Mexican stalkborers in the subfamily Crambinae (Lepidoptera: Pyralidae). Folia Entomológica Mexicana 75: 63-76.

Agrawal, A.A., M.T. Johnson, A.P. Hastings, and J.L. Maron. 2013. A field experiment demonstrating plant life-history evolution and its eco-evolutionary feedback to seed predator populations. The American Naturalist 181(S1): S35-S45.

Águila, R.N. & L.F. de Armas. 2015. The unusual natural history of the Cuban Endemic skipper Chioides Marmorosa (Lepidoptera: Hesperiidae). Boletín de la Sociedad Entomológica Aragonesa 56: 319-325.

Aguila, R. N. and A.B. Cañamero. 2012. A list of Cuban Lepidoptera (Arthropoda: Insecta). Zootaxa 3384(1): 1-59.

Ahola, M. and J. Silvonen. 1981. Immature stages of *Xestia kongsvoldensis* and *Xestia tecta* (Lepidoptera, Noctuidae). Notulae Entomologicae 61(4): 183-189.

Ahola, M. and K. Silvonen. 2008. Larvae of Northern European Noctuidae, Volume 2. Kuva Seppälä Yhtiöt Oy, Vaasa, Finland. 672 pp.

Ahola, M. and J.D. Lafontaine. 1990. Larvae of *Xestia kolymae* (Herz) and *X. lorezi* (Staudinger) Lepidoptera: Noctuidae), with notes on the geographical variation of the latter. Insect Systematics & Evolution 21(1): 77-90.

Ahola, M., K. Silvonen, H. Kronholm, K.-E. Lundsten and P.R. Sundell. 2006. Description of the larva of *Xestia atrata* (Morrison, 1874) (Lepidoptera: Noctuidae) with notes on the biology of the species. Entomologica Fennica 17: 65-72.

Ahola, M., D.R. Davis, J. Itämies, R. Leinonen, and M. Mutanen. 2017. Description of immature stages of *Nemophora bellela* (Walker, 1863) (Lepidoptera: Adelidae). Entomologica Fennica 28: 49-56.

Aiello, A. 1979. Life history and behavior of the Case-Bearer *Phereoca alluella* (Lepidoptera: Tineidae). Psyche 86: 125-136.

Aiello, and Solis. 2003. Defense mechanisms in Pyralidae and Choreutidae: fecal stalactites and escape holes, with remarks aboutcocoons, camouflage and aposematism. Journal of the Lepidopterists' Society 57(3): 168-175.

Ainslie, C.N. 1910. The New Mexico range caterpillar. U. S. Department of Agriculture, Bureau of Entomology, Bulletin 85(5): 59-96.

Akkuzu, E., H. Ayberk, and S. Inac. 2007 Hawk moths (Lepidoptera: Sphingidae) of Turkey and their zoogeographical distribution. Journal of Environmental Biology 28(4): 723-730.

Albaugh, D. 1986. Foodplants of thirtysix worldwide Saturniidae (Lepidoptera: Saturniidae). Young Entomologists' Society Quarterly 1986: 39-43.

Albu, V. 1997. Notes on the Sesiid fauna of southwestern West Virginia. Journal of the Lepidopterists’ Society 51(3): 249-256.

Albu, V. and E. Metzler. 2004. Lepidoptera of North America 5. Contributions to the knowledge of southern West Virginia Lepidoptera. Contributions of the C. P. Gillette Museum of Arthropod Diversity Colorado State University. 82 pp.

Alexander, C.P. 1940. The Presidential Range of New Hampshire as a biological environment, with particular reference to the insects. American Midland Naturalist 24(10): 104-132.

Alford, D.V. 1995. A Color Atlas of Pest of Ornamental Trees, Shrubs and Flowers. John Wiley and Sons, Inc., New York.

Alford, D.V. 2012. Pests of Ornamental Trees, Shrubs and Flowers: A Colour Handbook. CRC Press. Boca Raton, Florida.

Aliniaze, M.T. and E.M. Stafford, 1972. Notes on biology, ecology, and damage of Platynota stultana Lepidoptera-Tortrricidae on grapes. Journal of Economic Entomology 65(4): 1042-1044.

Alipanah, H. 2009. A brief study on the tribes Tortricini and Archipini (Lepidoptera: Tortricinae) from Iran. Entomofauna Zeitschrift für Entomologie 30(10): 137-152.

Alipanah, H. and J. Baixeras. 2011. A new species of *Hedya* Hübner from Iran with proposed rearrangement of some species currently assigned to *Metendothenia* Diakonoff (Lepidoptera: Tortricidae). Zootaxa, 2879, 33-44.

Alipanah, H., C. Gielis, A. Sari, A. Sarafrazi, and S. Manzari. 2011. Phylogenetic relationships in the tribe Oxyptilini (Lepidoptera, Pterophoridae, Pterophorinae) based on morphological data of adults. Zoological Journal of the Linnean Society 163(2): 484-547.

Allan, P.B.M. 1949. Larval Foodplants, A comprehensive guide to the foodplants of British Lepidoptera with alternative plants. Warkins and Doncaster, Hawkhurst, Kent, Great Britain.

Allen, A.A. 1992. *Cacoecimorpha pronubana* (Hübner) (Lep., Tortricidae) bred from flowers and seed-head of *Aegopodium.* Entomologist’s Record and Journal of Variation 104(11-12): 288.

Allen, R.T. and R.L. Brown. 1991. The biota of Magazine Mountain (II): A preliminary list of the macrolepidoptera fauna. Proceedings of the Arkansas Academy of Sciences 45: 18-21.

Allen, T.J. 1997. The Butterflies of West Virginia and Their Caterpillars. University of Pittsburgh Press, Pittsburgh, Pennsylvania. 388pp.

Allen, T.J., J.P. Brock and J. Glassberg, 2005. Caterpillars in the Field and Garden, A field Guide to the Butterfly Caterpillars of North America. Oxford University Press, New York.

Allred, D.M. 1969. Lepidoptera of the Nevada Test Site. Western North American Naturalist 29(1): 42.

Allyson, S. 1976. North American larvae of the genus *Loxostege* Hübner (Lepidoptera: Pyralidae: Pyraustinae). The Canadian Entomologist 108(1): 89-104.

Allyson, S. 1977. A study of some North American larvae of the genus *Tetralopha* Zeller (Lepidoptera: Pyralidae: Epipaschiinae). The Canadian Entomologist 109(3): 329-336.

Allyson, S. 1981. Last instar larvae of Pyraustini of America north of Mexico (Lepidoptera: Pyralidae). The Canadian Entomologist 113(06): 463-518.

Allyson, S. 1984. Description of last-instar larvae of 22 species of North American Spilomelini (Lepidoptera: Pyralidae: Pyraustinae) with a key to species. The Canadian Entomologist 116(10): 1301-1334.

Allyson, S. 1986. Sod webworms: the larva of *Microcrambus elegans (*Clem.) (Pyralidae: Crambinae). Journal of the Lepidopterists’ Society 40(4): 315-317.

Almeida J.V.C.S., R.F. da Silva, J.O. Dantas, and A.E.O. Alves 2024 Capítulo 4, Diversidade de Borboletas (Lepidoptera), Polinização e Herbivoria em um Remanescente de Mata Atlântica Em Sergipe pp. 50-70 In: C.T.A. Oliveira and F.L.D. Oliveira (eds.). Zoologia: Divulgando o Conhecimento Científico, Volume 2. Editora Científica Digital Ltda, Guarujá, São Paulo, Brasil.

Alston, D. and M. Murray. 2007. Peach Trwig Borer (*Anarsia lineatella*). Utah Pest Fact Sheet ENT-36-07.

Altesor, P., V.R. Horas, M.P. Arcia, C. Rossini, P.H.G. Zarbin, A. González. 2010 Reproductive behaviour of *Crocidosema (=Epinotia) aporema* (Walsingham) (Lepidoptera: Tortricidae): temporal pattern of female calling and mating. Neotropical Entomology 39(3): 324-329.

Althoff, D.M. and O. Pellmyr. 2002. Examining genetic structure in a bogus yucca moth: a sequential approach to phylogeography. Evolution 56(8): 1632-1643.

Althoff, D.M., J.D. Groman, K.A. Segraves and O. Pellmyr. 2001. Phylogeographic structure in the bogus yucca moth *Prodoxus quinquepunctellus* (Prodoxidae): comparisons with coexisting pollinator yucca moths. Molecular Phylogenetics and Evolution, 21: 117-127.

Amante, E. 1965. Observações bionômicas sobre *Hypocala andremona* (Cram.) (Lepidoptera-Noctuidae) praga do caquizeiro. O Biológico 32(5): 97-101.

Amer, A.F.M. 2013. Catocalinae of Egypt (Lepidoptera, Noctuidae). Al-Azhar Bulletin of Science 24(1-C): 1-16.

Anagnostakis S.L., K.M. Welch, J.W. Snow, K. Scarborough, and T.D. Eichlin. 1994. The rediscovery of the clearwing chestnut moth, *Synanthedon castaneae* (Busck) (Lepidoptera: Sesiidae) in Connecticut. Journal of the New York Entomological Society 102(1): 111-112.

Anderson, R.A. 1974. Three new United States records (Lycaenidae and Nymphalidae) and other unusual captures from the lower Florida Keys. Journal of the Lepidopterists' Society 28(4): 354-358.

Andre, E., [1907-1908]. Élevage des vers a soie sauvages. Gustave Ficker, Paris. 252 pp.

Anikin, V.V., S.A. Sachkov, V.V. Zolotuhin, and P.Y. Ustjuzhanin. 2003. “Fauna Lepidopterologica Volgo-Uralensis" 150 years later: changes and additions. Part 7. Pyrales et Pterophores. Atalanta 34(1-2): 223-250.

Anikin, V.V., S.A. Sachkov, V.V. Zolotuhin. 2004. "Fauna Lepidopterologica Volgo-Uralensis" 150 years later: changes and additions. Part 8. Gracillarioidea. Atalanta 35(1-2): 2141-151.

ANSP:ENT. 2015. Academy of Natural Sciences Entomology Collection, Drexel University, Philadelphia, Pennsylvania. In: SCAN. P.L. Heinrich, E. Gilbert, N.S. Cobb, and N. Franz. Symbiota collections of arthropods network (SCAN): A data portal built to visualize, manipulate, and export species occurrences. [Dataset]. http://scan-bugs.org/portal/collections/index.php [Accessed 08 March 2019]

Antonelli, A.L., C.H. Shanks, Jr., and G.C. Fisher. 2004. Small Fruit Pests Biology, Diagnosis and Management. Washington State University Extension and the U.S. Department of Agriculture, EB1388.

Anweiler, G.G. 2008. Status of the Verna's Flower Moth (*Schinia verna*) in Alberta. Alberta Wildlife Status Report No. 65.

Anweiler, G.G. 2009. Revision of the New World *Panthea* Hübner (Lepidoptera, Noctuidae) with descriptions of 5 new species and 2 new subspecies. In: B.C. Schmidt and J.D. Lafontaine (Eds.). Contributions to the Systematics of New World Macro-Moths. ZooKeys 9: 97-134. doi: 10.3897/zookeys.9.157

Arakelian, G. 2007. New agricultural pest for Southern California, Bougainvillea Looper (*Disclisioprocta stellata*). Los Angeles County Agricultural Commissioner/Weights & Measures Department. Los Angeles, California.

Arbogast, R.T., R.V. Byrd, G. Chauvin, and R.G. Strong. 1984. The egg of *Hofmannophila pseudospretella* (Oecophoridae): fine structure of the chorion. Journal of the Lepidopterists’ Society 38(3): 202-208.

Arbogast, R.T., J.H. Brower, and R.G. Strong. 1989. External morphology of the eggs of *Tinea pallescentella* Stainton, *Tinea occidentella* Chambers, and *Niditinea fuscella* (L.) (Lepidoptera: Tineidae). International Journal of Insect Morphology and Embryology 18(5): 321-328.

Arias-Penna, D.C., J.B. Whitfield, D.H. Janzen, W. Hallwachs, L.A. Dyer, S.M. Smith, P.D.N. Hebert, and J.L. Fernández-Triana. 2019. A species-level taxonomic review and host associations of *Glyptapanteles* (Hymenoptera, Braconidae, Microgastrinae) with an emphasis on 136 new reared species from Costa Rica and Ecuador. ZooKeys 890: 1-685.

Arnett, R.H. Jr. 1993. American Insects. A handbook of the insects of America North of Mexico. Sandhill Cranes Press, Gainesville, USA

Arnett, R.H. Jr. 2000. American Insects. A handbook of the insects of America North of Mexico. 2^nd^ Edition. CRC Press, Boca Raton, FL.

Arnscheid, W.R. and M. Weidlich. 2017. Psychidae. Microlepidoptera of Europe, Volume: 8. Brill, Leiden, Netherlands.

Arthur, A.P. and Y.M. Powell. 1990. Description of the last-instar larva of *Cochylis arthuri* (Lepidoptera: Cochylidae) and characters for separating it from last-instar larva of *Cochylis hospes* Walsingham. Canadian Entomologist 122: 627-631.

Artz, D.R., C.A. Villagra, and R.A. Raguso. 2010. Spatiotemporal variation in the reproductive ecology of two parapatric subspecies of *Oenothera cespitosa* (Onagraceae). American Journal of Botany 97(9): 1498-1510.

Askew, R.R. and P.A. van B. Stafford. 2008. Butterflies of the Cayman Islands. Apollo Books, Stenstrup.

Asselbergs, J.E.F. 2008. Order Lepidoptera, superfamily Pyraloidea. Pp. 469-561 In: A. van Harten, (ed.). Arthropod Fauna of the UAE Volume I. Dar Al Ummah Printing, Publishing, Distribution & Advertising, Abu Dabi, United Arab Emirates.

ASU:ASUHIC. 2015. Arizona State University Hasbrouck Insect Collection, Arizona State University, Tempe, Arizona. In: SCAN. P.L. Heinrich, E. Gilbert, N.S. Cobb, and N. Franz. Symbiota collections of arthropods network (SCAN): A data portal built to visualize, manipulate, and export species occurrences. [Dataset]. http://scan-bugs.org/portal/collections/index.php [Accessed 09 March 2019]

Atkins, E. L. 1963. Macadamia moth pests in California: known and potential. California Macadamia Society Yearbook 9: 51 54.

Aurelian, V.M., M.L. Evenden, and G.J. Judd. 2012. Small‐plot studies comparing pheromone and juice baits for mass‐trapping invasive *Synanthedon myopaeformis* in Canada. Entomologia Experimentalis et Applicata 145(2): 102-114.

Austin, G.T. 1998. A new subspecies of Euphilotes pallescens Lycaenidae from the northern Great Basin of Nevada. pp. 815-818. In: T.C. Emmel (ed). Systematics of western North American butterflies. Mariposa Press, Gainesville, Florida.

Austin, G.T. 2010. Scientific Note: Moth community from a northcentral Florida location - a taxonomic checklist. Tropical Lepidoptera Research 20(1): 41-44.

Austin, G.T. and J.F. Emmel. 1998. New subspecies of butterflies (Lepidoptera) from Nevada and California. pp. 501-522 In: T.C. Emmel (ed.). Systematics of Western North American Butterflies, Marioposa Press, Gainesville, Florida.

Austin, G.T. and P.J. Leary. 2008. Larval hostplants of butterflies in Nevada. Holarctic Lepidoptera 12(1-2): 11-150.

Austin, K.A. and J.J. Dombroskie. 2020a. A taxonomic revision of the Archipini of the Caribbean (Lepidoptera, Tortricidae, Tortricinae). ZooKeys 982: 33-147. <https://doi.org/10.3897/zookeys.982.52363>.

Austin, K.A. and J.J. Dombroskie. 2020b. New combinations in Neotropical Archipini and Atteriini (Lepidoptera: Tortricidae: Tortricinae), with the description of a new genus. Proceedings of the Entomological Society of Washington 122(1): 1-11.

Austin, K., V. Nazari, J.-F. Landry, and S.R. Johnson. 2017. *Lepidotarphius perornatella* (Walker, 1864) (Lepidoptera: Glyphipterigidae) new to North America. News of the Lepidopterists’ Society 59(4): 182-184.

Averill, A.L. and M.M. Sylvia. 1998. Cranberry insects of the Northeast: A guide to identiﬁcation, biology and management. Department of Entomology and Cranberry Experiment Station, University of Massachusetts, Amherst, Massachusetts.

Ayre, G.L. 1980. The biology and life history of the cutworm *Amphipoea interoceanica* (Lepidoptera: Noctuidae), a new pest of strawberry in Manitoba. The Canadian Entomologist 112(2): 127-130.

Bächtold, A. and E. Alves-Silva. 2013. Behavioral strategy of a lycaenid (Lepidoptera) caterpillar against aggressive ants in a Brazilian savanna. Acta Ethologica 16(2): 83-90.

Back, W. 2013. Verbreitung von *Euchloe ausonia* (Hübner, 1804), *Euchloe daphalis* (Moore, 1865) und *Euchloe persica* Verity, 1908 stat. nov. im Iran. Atalanta 44: 109 - 117.

Back, W. 2015. Molekularbiologische Bestatigung des Holotypus von Euchloe ausonides ogilvia Back, 1990 und ein Vergleich mit weiteren Unterarten von *Euchloe ausonides* (Lucas, 1852) und *Euchloe ausonia kamchatkensis* Back, 2008 (Lepidoptera, Pieridae). Atalanta 46: 107-110.

Backstrom, P. and M.J. Lynch. 2011. First encounters: a (moth) night to remember. Southern Lepidopterists' News 33(4): 157-166.

Baer, C.S. 2018. Shelter building and extrafloral nectar exploitation by a member of the *Aristotelia corallina* species complex (Gelechiidae) on Costa Rican acacias The Journal of the Lepidopterists' Society 72(1): 44-52.

Baerg, W.J. 1935. Three shade tree insects. II. Great elm leaf beetle, catalpa sphinx and eastern tent caterpillar. Bulletin. Arkansas Agricultural Experiment Station 317: 1-28.

Bailey, T.E. and L.T. Kok. 1982. Biology of *Frumenta nundinella* (Lepidoptera: Gelechiidae) on horsenettle in Virginia. The Canadian Entomologist 114: 139-144.

Bailowitz, R.A. 1985. Systematics and biology of Ascia (Ganyra) populations in the Sonoran Desert. The University of Arizona. Masters Thesis.

Bailowitz, R.A. 1988. Systematics of *Ascia* (Ganyra) (Pieridae) populations in the Sonoran Desert. Journal of Research on the Lepidoptera, 6: 73-81.

Bailowitz, R.A. 2007. Finding Butterflies in Arizona: A Guide to the Best Sites. Big Earth Publishing, Boulder, Colorado.

Bailowitz, R.A. and J. P. Brock. 1991. Butterflies of Southeastern Arizona. Sonoran Arthropod Studies, Inc., Tucson, Arizona. 342 pages.

Baker, B.R. 1985. Sesiidae. pp. 369-388 *in* A.M. Emmet (ed.), The Moths and Butterflies of Great Britain and Ireland, Volume 2, Cossidae- Heliodinidae. Harley Books, Colchester, Essex, England.

Baker, W.L. 1972. Eastern Forest Insects. U.S. Department of Agriculture, Forest Service, Miscellaneous Publications 1175: 1-642.

Baker, J.H. 1959. A list of Geometridae from Baker County, Oregon. Journal of the Lepidopterists' Society 13(4): 217-220.

Balcázar Lara, M.A. 2018. Biodiversidad de la Sierra Tarahumara. Lepidoptera: Mimallonoidea, Lasiocampoidea, Bombycoidea y Pyraloidea. Comisión nacional para el conocimiento y uso de la biodiversidad. https://doi.org/10.15468/8aqxgq [accessed 11 APRIL 2019 via (SCAN 2015)].

Balciunas, J.K. and M.C. Minno 1985. Insects damaging to *Hydrilla* in the USA. Journal of Aquatic Plant Management 23: 77-83.

Baldizzone, G. 2020. The South American Coleophoridae of the Zoological Museum of Copenhagen. Contribution to the knowledge of the Coleophoridae. CXLIII (Lepidoptera: Coleophoridae). SHILAP Revista de Lepidopterología 48(190): 197-222.

Baldizzone, G., and J.-F. Landry 1993. *Coleophora cratipennella* Clemens, 1864 and *C. tamesis* Waters, 1929, two distinct species (Lepidoptera, Coleophoridae). Nota Lepidopterologica 16: 2-12.

Baldizzone, G., H.V.D. Wolf, and L.-F. Landry. 2006. World Catalogue of Insects. Volume 8: Coleophoridae, Coleophorinae (Lepidoptera). Apollo Books, Senstrup, Denmark.

Balduf, W.V. 1959. Obligatory and facultative insects in rose hips, their recognition and bionomics. Illinois Biological Monograph 26. 194 pp.

Bali, G.P. K., A.S. Kaleka, and D. Singh. 2021. A new combination of a tussock moth species under genus *Himala* Moore (Lepidoptera: Erebidae: Lymantriinae) and its range extension. Records of the Zoological Survey of India 120(4): 357-362.

Ballmer, G.R. and G.F. Pratt. 1989a. A survey of the last instar larvae of the Lycaenidae (Lepidoptera) of California. Journal of Research on the Lepidoptera 27(1): 1-81.

Ballou, H.A. 1912. Insect pests of the Lesser Antilles. Imperial Department of Agricaulture for the West Indies Pamphlet Series 71. Commissioner of Agriculture, Bridgetown, Barbados.

Balmer, H. 1982. Vergleichende Untersuchungen zue Biologie schweizerischer Cnephasiini (Lep., Tortricidae). Bulletin Societe Entomologique Suisse 55: 349-382.

Balogh, G.J. 1987. New localities for *Schinia indiana* (Smith) (Noctuidae). Ohio Lepidopterists 9: 15-16.

Balogh, G.J. and J.H. Wilterding. 1998. A new species of *Pyla* Grote and redescription of *Pyla aequivoca* Heinrich (Lepidoptera: Pyralidae: Phycitinae). Proceedings of the Entomological Society of Washington 100 (4): 704-723.

Ban, X.S., N. Jiang, R. Cheng, D.Y. Xue, and H.X. Han. 2018. Tribal classification and phylogeny of Geometrinae (Lepidoptera: Geometridae) inferred from seven gene regions. Zoological Journal of the Linnean Society 184(3): 653-672.

Bänziger, H. 1987. Biological and taxonomic studies on immature and adult fruit-piercing moths in Nepal, with reference to Thailand. Natural History Bulletin of the Siam Society 35: 1-17.

Baraniak, E. 2007. Taxonomic revision of the genus *Plutella* Schrank, 18O2 (Lepidoptera: Plutellidae) from the Palaearctic region with notes on its phytogeny. Polskie Pismoi Entomologiczne 76(Supplement): 1-53

Barbehen, R.V. 1994. Host plants of *Poanes melane* (Hesperiidae). Journal of the Lepidopterists' Society 48(4): 386-388.

Barbosa, P., A. Segarra and P. Gross, 2000. Structure of two macrolepidopteran assemblages on *Salix nigra* (Marsh) and *Acer negundo* L.: abundance, diversity, richness, and persistence of scarce species. Ecological Entomology 25(4): 374-379.

Barbour, J.G. and E. Kiviat, 1997. Introduced purple loosestrife as a host of native Saturniidae (Lepidoptera). Great Lakes Entomologist 30: 115-122.

Barnes, W. and F.R. Benjamin. 1922a. Notes: New genera and species Noctuidae. Contributions to the Natural History of the Lepidoptera of North America 5(1): 41-51.

Barnes, W. and F.R. Benjamin. 1922b. A revision of the noctuid moths heretofore referred to the genus *Grotella*. Contributions to the Natural History of the Lepidoptera of North America 5(1): 8-27, pI. 1.

Barnes, W. and F.H. Benjamin. 1923a. Notes and new species (Geometridae, Lepid.). The Lepidopterists 4(1): 9-12.

Barnes, W. and F.H. Benjamin. 1923b. Nomenclature notes and new species. Contributions to the Natural History of the Lepidoptera of North America 5(2): 52-96.

Barnes, W. and F.R. Benjamin. 1924. Notes and new species. Contributions to the Natural History of the Lepidoptera of North America 5(3): 99-199.

Barnes, W. and F.R. Benjamin. 1925a. Notes and new species (Lepidoptera). Proceedings of the Entomological Society of Washington 27(6): 123-129.

Barnes, W. and F.R. Benjamin. 1925b. Notes on Arizona Phalaenidae (Lepid.). The Pan-Pacific Entomologist 2(1): 16-21.

Barnes, W. and F.R. Benjamin. 1926a. New U. S. Lepidoptera records with notes. Proceedings of the Entomological Society of Washington 28(1): 16-21.

Barnes, W. and F.R. Benjamin. 1926b. Notes on the *egans* group of *Oligia* (Phalaenidae, Lepid.). Canadian Entomologist 60(11): 264-265.

Barnes, W. and F.R. Benjamin. 1926c. Notes and new species (Lepid. Phalarnidae). Pan Pacific Entomologist 2(3): 106-110.

Barnes, W. and F.R. Benjamin. 1926d. Notes and new species (Lepid., Phalaenidae). The Canadian Entomologist 58(12): 303-310. [note: species described in this article are dated “1927”]

Barnes, W. and A. Busck. 1920. Notes and new species. Contributions to the Natural History of the Lepidoptera of North America 4(4): 211-278 & index.

Barnes, W. and A.W. Lindsey. 1921a. The Pterophoridae of American, North of Mexico. Contributions to the Natural History of the Lepidoptera of North America 4(4): 281-452, plates 41-54.

Barnes, W. and A.W. Lindsey. 1921b. Notes on Noctuidæ with descriptions of some new species (Lep.). Psyche 18(5/6): 156-159.

Barnes, W. and A.W. Lindsey. 1922. A new genus and species of Noctuidae (Lep.). Bulletin of the Brooklyn Entomological Society 52:56-57.

Barnes, W.M. and J.H. McDunnough. 1910a. New species and varieties of North American Lepidoptera. Journal of the New York Entomology Society 18: 149-162.

Barnes, W.M. and J.H. McDunnough. 1910b. List of Sphingidae of America north of Mexico. Psyche 17(5): 190-206.

Barnes, W.M. and J.H. McDunnough. 1911a. On *Cea immacula* and allied species. Canadian Entomologist 43: 318-320.

Barnes, W.M. and J.H. McDunnough. 1911b. Revision of the Cossidae of North America. Contributions to the Natural History of the Lepidoptera of North America 1(1): 1-13 plus I-VII plates.

Barnes, W.M. and J.H. McDunnough. 1911c. The lasiocampid genus *Gloveria* and its allies. Contributions to the Natural History of the Lepidoptera of North America 1(2): 1-17.

Barnes, W.M. and J.H. McDunnough. 1912a. Fifty new species and varieties. Contributions to the Natural History of the Lepidoptera of North America 1(5): 1-36, pls 1-3.

Barnes, W.M. and J.H. McDunnough. 1912b. The genus *Alpheias* Rag. and its allies. Contributions to the Natural History of the Lepidoptera of North America 1(5): 37-42.

Barnes, W.M. and J.H. McDunnough. 1912c. New species and genera of North American Lepidoptera. The Canadian Entomologist 44: 90-93.

Barnes, W.M. and J.H. McDunnough. 1912d. New species and genera of North American Lepidoptera. The Canadian Entomologist 44: 52-57.

Barnes, W.M. and J.H. McDunnough. 1912e. New species and genera of North American Lepidoptera. The Canadian Entomologist 44: 17-22.

Barnes, W.M. and J.H. McDunnough. 1912f. New species and genera of North American Lepidoptera. The Canadian Entomologist 44: 122-127.

Barnes, W.M. and J.H. McDunnough. 1912g. Illustrations of rare and typical Lepidoptera. Contributions to the Natural History of the Lepidoptera of North America 1(4): 1-57, plus 27 plates and index.

Barnes, W.M. and J.H. McDunnough. 1912h. Revision of the Megathymidae. Contributions to the Natural History of the Lepidoptera of North America 1(3): 1-43, I-VI.

Barnes, W.M. and J.H. McDunnough. 1912i. On the larval stages of certain artican species. The Canadian Entomologist 44: 188-191.

Barnes, W.M. and J.H. McDunnough. 1913. New North American Lepidoptera with notes on described species. Contributions to the Natural History of the Lepidoptera of North America 2(3): 93-165, pl. 1-9.

Barnes, W.M. and J.H. McDunnough. 1914. Some new North American Pyraustinae. Contributions to the Natural History of the Lepidoptera of North America 2(6): 224-250, pls 1-2.

Barnes, W.M. and J.H. McDunnough. 1916a. Notes on Walker’s types of Geometridae in the D’Urban collection. Contributions to the Natural History of the Lepidoptera of North America 3(1): 35-42.

Barnes, W.M. and J.H. McDunnough. 1916b. New species and varieties of North American Lepidoptera. Contributions to the Natural History of the Lepidoptera of North America 3(1): 5-34.

Barnes, W.M. and J.H. McDunnough, 1917a. Check list of Lepidoptera of Boreal America. Herald Press, Decatur, Illinois.

Barnes, W.M. and J.H. McDunnough, 1917b. A revision of the genus *Hydriomena* based on male genitalia. Contributions to the Natural History of the Lepidoptera of North America 4(1): 6-61.

Barnes, W.M. and J.H. McDunnough, 1918a. Life histories of North American species of the genus *Catocala*. Bulletin of the American Museum of Natural History 38(5): 147-177.

Barnes, W.M. and J.H. McDunnough. 1918b. Notes and new species. Contributions to the Natural History of the Lepidoptera of North America 4(2): 61-213, plates 11-25.

Barnes, W.M. and J.H. McDunnough. 1918c. Illustrations of the North American species of the genus *Catocala*. Memoirs of the American Museum of Natural History, N.S. 3(1): 1-48 plus plates I-XXII.

Barnes, W.M. and J.H. McDunnough. 1920. Notes and new species. Contributions to the Natural History of the Lepidoptera of North America 4(3): 211-248, plates I-XI, plus index.

Barr, N.B., L.A. Ledezma, R.E. Farris, M.E. Epstein, and T.M. Gilligan. 2011. A multiplex real-time polymerase chain reaction assay to diagnose *Epiphyas postvittana* (Lepidoptera: Tortricidae). Journal of Economic Entomology 104(5): 1706-1719.

Barry, J.D., C.R. Rodriguez-Saona, D.F. Polk, and A. Zhang. 2010. Seasonal abundance, life history, and parasitism of *Caloptilia porphyretica* (Lepidoptera: Gracillariidae), a leafminer of highbush blueberry. Journal of Economic Entomology 103(2): 284-291.

Bassi, G. and W. Mey. 2011. Crambidae, Crambina (Lepidoptera, Pyraloidea). In: W. Mey. Basic pattern of Lepidoptera diversity in southwestern Africa. Esperiana Memoir 6: 1-320.

Bates, M. 1928. Notes on the Cypress Sphinx (*Isoparce cupressi*). The Florida Entomologist 12(2): 20-21.

Bates, M. 1945. The Butterflies of Cuba. Bulletin of the Museum of Comparative Zoology at Harvard College 78(2): 61-258.

Batiste, W.C., J. Joos, and R.C. King. 1970. Studies on sources of the tomato pinworm attacking tomatoes in northern California. Journal of Economic Entomology 63: 1484-1486.

Bauder, J.A.-S., S. Handschuh, B.D. Metscher, and H.W. Krenn. 2013. Functional morphology of the feeding apparatus and evolution of proboscis length in metalmark butterflies (Lepidoptera: Riodinidae). Biological Journal of the Linnean Society 110(2): 291-304.

Bauer, D.L. 1948. Methods for collecting buck-moths (*Hemileuca*, Saturniidae). Lepidopterists’ News 2: 81-82

Baumberger, J.P., 1917. Hibernation: A periodical phenomenon. Annals of the Entomological Society of America 10(2): 179-186.

Beacher, J.H. 1947. Studies of pistol case-bearer parasites. Annals of the Entomological Society of America 40(3): 530-544.

Beadle, D. and S. Leckie. 2012. Peterson Field Guide to Moths of Northeastern North America. Houghton Mifflin Harcourt, New York, New York.

Beans, B.E. and L. Niles. 2003. Endangered and Threatened Wildlife of New Jersey. Rutgers University Press, New Brunswick, NJ.

Beardsley, J.W. Jr. 1979. New immigrant insects in Hawaii: 1962 through 1976. Proceedings, Hawaiian Entomological Society 1937(61): 1962-76.

Beavan, S.D. and R.J. Heckford. 2016. *Chionodes fumatella* (Douglas, 1850) (Lepidoptera: Gelechiidae): Discovery of the larva in the British Isles. Entomologist’s Gazette 67: 3-14.

Beccaloni, G. W., M.J. Scoble, G.S. Robinson, and B. Pitkin. (Ed.). 2003. The Global Lepidoptera Names Index (LepIndex). World Wide Web electronic publication. http://www.nhm.ac.uk/entomology/lepindex [accessed April 2005].

Beccaloni, G., M. Scoble, I. Kitching, T Simonsen, G. Robinson, B. Pitkin, A. Hine, and C. Lyal. (eds.). 2003b. The Global Lepidoptera Names Index (LepIndex). World Wide Web electronic publication. http://www.nhm.ac.uk/our-science/data/lepindex/lepindex/ [accessed 22 December 2018].

Beccaloni, G.W., S.K. Hall, A.L. Viloria, and G. S. Robinson. 2008. Catalogue of the hostplants of the Neotropical Butterflies, Catálogo de las plantas huéspedes de las mariposas Neotropicales. Monografias del Tercer Milênio 8: 1-536.

Beck, A.F. 1987. 1986 Season Summary. Zone 2. Southeast: Alabama, Florida, Georgia, Mississippi, North Carolina, South Carolina, Tennessee, Virginia, Bermuda. News of the Lepidopterists’ Society 30(2): 36-37.

Beck, H. 1960. Abhandlungen zur Larvalsystematik der insekten. no 4. Die Larval Systematik der Eulen. Berlin. Akademis Verlag.

Becker, V.O. 1981. Identities and provenance of the gelechioid moths originally described by Francis Walker from ‘unknown countries’. Systematic Entomology 6(2): 137-141.

Becker, V.O. 2001a. A new Antillean moth of the genus *Enigmogramma* Lafontaine & Poole (Noctuidae, Plusiinae). Revista Brasileira de Zoologia 18: 145-147. doi: 10.1590/S0101-81752001000100016.

Becker, V.O. 2001b. The identity of *Hemeroblemma lienaris* Hübner and a review of the neotropical moths of the pannosa-complex of Epidromia Guenée (Noctuidae, Catocalinae). Revista Brasileira de Zoologia 18): 961-964.

Becker, V.O. 2002. The Noctuoidea (Lepidoptera) from Cuba described by Herrich-Schäffer and Gundlach in the Gundlach Collection, Havana. Revista Brasileira de Zoologia 19: 349-391.

Becker, V.O. 2009. A review of the New World *Atteva* Walker moths (Yponomeutidae, Attevinae). Revista Brasileira de Entomologia 53(3): 349-355.

Becker, V.O. 2013. Taxonomic changes in the Neotropical Pericopina and Ctenuchina moths (Erebidae, Arctiinae, Arctiini), with description of new taxa. The Journal of Research on the Lepidoptera 46: 53-66.

Becker, V.O. 2014. Checklist of New World Notodontidae (Noctuoidea). Lepidoptera Novae, 7(1): 1-40.

Becker, V.O. 2023. A review of the New World genera *Alatuncusia* Amsel, 1956 and *Dichochroma* Forbes, 1944 with new synonymies, new combinations, and description of two new species (Lepidoptera: Crambidae, Glaphyriinae). Shilap. Revista de Lepidopterologia 51(201): 27-36.

Becker, V.O. and S.E. Miller. 2002. The large moths of Guana Island, British Virgin Islands: a survey of efficient colonizers (Sphingidae, Notodontidae, Noctuidae, Arctiidae, Geometridae, Hyblaeidae, Cossidae). Journal of the Lepidopterists' Society 56(l): 9-44.

Behr, H. 1870. Synopsis Noctuidarum Hucusque in Californa repertarum. Transactions of the American Entomological Society 3: 23-28.

Behr, H. 1882. On the habits and economy of some species of Sphingidae. Papilo 2: 1-7.

Belik, A.G. 2000. On the correct placement of Erebia epipsodea Butler, 1868 within the genus *Erebia* Dalman, 1816 (Lepidoptera: Satyridae). Journal of Research on Lepidoptera 36: 16-23.

Beljaev, E.A. and S.V. Vasilenko. 2002. An annotated checklist of geometrid moths (Lepidoptera: Geometridae) from the Kamchatka Peninsula and adjacent islands. Entomologica Fennica 13: 195-235.

Benda, N. and D. Bracey. 2022. *Endothenia microptera* (Clarke), a tortricid moth, a new Florida State record. Tri-ology 61(4): 8.

Bendicho-Lopez, A. 1998. New distributional and foodplant records for twenty Cuban moths. Journal of the Lepidopterists’ Society 52: 212-214.

Bengtsson, B.Å. 1984. The Scythrididae (Lepidoptera) of Northern Europe. Fauna Entomologica Scandinavica 13: 1-137.

Bengtsson, B.Å. and R. Johansson. 2011. Nationalnyckeln till Sveriges flora och fauna. Fjärilar: Bronsmalar-rullvingemalar. Lepidoptera: Roeslerstammiidae-Lyonetiidae. ArtDatabanken, SLU, Uppsala, 494 pp.

Benjamin, F.H. 1934. Notes on the genus *Rothschildia* in the United States. The Pan-Pacific Entomologist 10(1): 12-16.

Benjamin, F.H. 1935. Notes and new species (Lepidoptera, Phalaenidae). Bulletin of the Southern California Academy of Sciences 34(3): 194-210.

Bennett, S.M. 1961. The Strawberry Leaf Roller Complex in Tennessee. Masters’ Thesis. University of Tennessee, Knoxville, Tennessee.

Bento, J. M.S., D.E. Nava, M.C.M. Chagas, A.H. Costa, D.J. Libardi, and J.R.P. Parra. 2006. Biology and mating behavior of the coconut moth *Atheloca subrufella* (Lepidoptera: Phycitidae). Florida Entomologist 89(2): 199-203.

Beregovoy, V.H. and J.G. Riemann. 1987. Infestation phenology of sunflowers by the banded sunflower moth, *Cochylis hospes* (Cochylidae: Lepidoptera) in the northern Plains. Journal of the Kansas Entomological Society 60(4): 517-527.

Berenbaum, M. 1978. *Taenidia interregima*, a new foodplant record for *Papilio polyxenes* (Papilionidae). Journal of the Lepidopterists’ Society 32(4): 303-304.

Berenbaum, M. 1982. New host plan records for *Agonopteris clemensella* (Oecophoridae). Journal of the Lepidopterists’ Society 36(2): 160.

Berenbaum, M.A. and T.L. Harrison 1994. *Agonopterix alstroemeriana* (Oecophoridae) and other Lepidopteran associates of poison hemlock (*Conium maculatum*) in east central Illinois. The Great Lakes Entomologist 27(1): 1-5.

Berenbaum, M. and S. Passoa, 1983. Notes on the biology of *Agonopterix alstroemeriana* (Clerck), with descriptions of the immature stages (Oecophoridae). Journal of the Lepidopterists' Society 37: 38-45.

Berg, T.B., N.M. Schmidt, T.T. Høye, P.J. Aastrup, D.K. Hendrichsen, M.C. Forchhammer, and D.R. Klein. 2008 High-Arctic plant - herbivore interactions under climate influence. Advances in Ecological Research 40: 275-298.

Bergh, J.C., T.C. Leskey, J.F. Walgenbach, W.E. Klingeman, D.P. Kain, and A. Zhang. 2009. Dogwood borer (Lepidoptera: Sesiidae) abundance and seasonal flight activity in apple orchards, urban landscapes, and woodlands in five eastern states. Environmental Entomology 38(3): 530-538.

Berneys, E.A. and D.H. Janzen. 1988. Saturniid and Sphingid caterpillars: two ways to eat leaves. Ecology 69(4): 1153-1160.

Bernays, E.A. and C.B. Montllor. 1989. Aposematism of *Uresiphita reversalis* larvae (Pyralidae). Journal of the Lepidopterists' Society 43(4): 261-273.

Berry, G.W. 1891. A sphinx larva feeding on mints. Insect Life 4: 203.

Bertkau, P. 1882. Bericht über die Leistungen im Gebiete der Anthropoden wahrend des Jahres 1881. Archiv für Naturgeschichte 48(2): 1-299.

Bess, J. 2005a. Conservation assessment for the brick-red borer moth (*Papaipema marginidens* (Guenee)). USDA Forest Service, Eastern Region. Milwaukee, Wisconsin. 25 pp.

Bess, J. 2005b. Conservation Assessment for the mayapple borer moth Papaipema rutila (Guenee). USDA Forest Service, Eastern Region. Milwaukee, Wisconsin. 25 pp.

Bess, J. 2005c. Conservation assessment for the jaguar flower moth (*Schinia jaguarina* Guenee). USDA Forest Service, Eastern Region. Milwaukee, Wisconsin. 34 pp.

Bess, J. 2005d. Conservation assessment for stoneroot borer moth (*Papaipema astuta* Bird). USDA Forest Service, Eastern Region. Milwaukee, Wisconsin. 26 pp.

Bethke, J.A. and B. Vander Mey. 2012. European pepper moth: a new invasive moth threatens California agriculture. UCNFA News, University of California 15(3): 2-10.

Betti, A. 1983. Contributo alla conoscenza dell’entomofauna del Cytisus scoparius. Italia Forestale e Montana 38: 98-103.

Betz, R.F., W.R. Rommel, and J.J. Dichtl. 1997. Insect herbivores of 12 milkweed (Asclepias) species. Pp. 7-19 In: Proceedings of the Fifteenth North American Prairie Conference, Bend, OR.

Beutelspacher, C.R. 1980. Mariposas diurnas del Valle de México. México, Ediciones Científicas La Prensa Médica Mexicana. 134 pp.

Beutenmüller, W. 1884. Food-plants of Lepidoptera, Papilo 4: 155-157.

Beutenmüller, W. 1889. Catelogue of Lepidoptera found within fifty miles of New York City. Annals of the New York Academy of Sciences 5(3): 199-230.

Beutenmüller, W. 1893. Notes on tranformations of some North American moths. Bulletin American Museum of Natural History 5: 87-94.

Beutenmüller, W. 1895. Descriptive catalogue of the Spingidae found within fifty miles of New York City. Bulletin of the American Museum of Natural History, 7: 275-320.

Beutenmüller, W. 1896a. Critical review of the Sesiidae found in America, north of Mexico. Bulletin American Museum of Natural History 8(7): 111-148.

Beutenmüller, W. 1896b. Transformations of some North American hawk-moths. Bulletin of the American Museum of Natural History 8: 291-298.

Beutenmüller, W. 1898. Descriptive catalogue of the bombycine moths found within fifity miles of New York City. Bulletin of the American Museum of Natural History 10: 353-448.

Beutenmüller, W. 1901. Monograph of the Sesiidae of America, north of Mexico. Memoirs of the American Museum of Natural History 1(4): 215-352.

Bibbs, C.S. and J.H. Frank. 2012. Saddleback Caterpillar Acharia stimulea (Clemens) (Insecta: Lepidoptera: Limacodidae). Entomology and Nematology Department, Florida Cooperative Extension Service, University of Florida. EENY-522

Biddinger, D. J. and A. J. Howitt. 1992. The food plants and distribution of the American plum borer (Lepidoptera: Pyralidae). Great Lakes Entomologist 25(3): 149-158.

Bidzilya, O. 2005. A review of the genus *Athrips* (Lepidoptera, Gelechiidae) in the Palaearctic region. Mitteilungen aus dem Museum für Naturkunde in Berlin, Deutsche Entomologische Zeitschrift 52(1): 3-72.

Bidzilya, O. 2021. New host-plants records of Afrotropical Gelechiidae (Lepidoptera), with description of three new species. Zootaxa 4952(3): 495-522.

Biederman, C.R. 1908. A new *Anisota* from Arizona (Lepidoptera, Heterocera, Ceratocampidae). Entomological News 19: 77.

Biezanko, C.M., R.E. Bertholdi and O. Baucke, 1949. Relacao dos principals insetos prejudiciais observados nos arredores de Pelotas nas plantas cultivadas e selvagens. Agros, Pelotas 2: 156-213.

Biezanko, C.M., A. Ruffinelli, and D. Link. 1974. Plantas y otras sustancias alimenticias de las orugas de los lepidópteros Uruguayos. Revista do Centro de Ciencias Rurais 4(2): 107-147.

Bigot, L. 2021. Précision concernant *Oidaematophorus poulini* Bigot & Picard, 2005 (Lepidoptera Pterophoridae). L’Entomologiste 77(2): 142.

Bigot, L. and J. Etienne. 2009. Les Pterophoridae de l’île de la Guadeloupe. Bulletin de la Société Entomologique de France 114: 463-467.

Bigot, L. and J. Picard. 2005. Nouvelles données sur les Ptérophores de la région du Bas Saint-Laurent (Quebec, Canada). Description d’ *Oidaematophorus poulini* n.sp. Bulletin de la Société Entomologique de France 110: 407-411.

Bippus, M. 2016a. Notes on Lepidoptera from the Seychelles. Phelsuma 24: 35-71.

Bippus, M. 2016b. New or poorly known Microlepidoptera from the Mascarenes (Lepidoptera: Autostichidae, Bedellidae, Batrachedridae, Carposinidae, Epermeniidae, Gelechiidae, Tineidae, Tortricidae). Contributions to Entomology 66(2):347-370.

Bippus, M. 2019. Pyraloidea of Mauritius and neighbouring islands (Lepidoptera). Phelsuma 27: 36-57.

Bippus, M. 2020. Records of Lepidoptera from the Malagasy region with description of new species (Lepidoptera: Tortricidae, Noctuidae, Alucitidae, Choreutidae, Euteliidae, Gelechiidae, Blastobasidae, Pterophoridae, Tonzidae, Tineidae, Praydidae, Cosmopterigidae, Batrachedridae). Phelsuma 28: 60-100.

Bird H. 1902. New histories and species in *Hydroecia*. Canadian Entomologist 34: 107-118.

Bird H. 1907. New histories and species in *Papaipema* (*Hydroecia*). Canadian Entomologist 39: 269-276, 309-317.

Bird H. 1908. New histories and species in *Papaipema* (*Hydroecia*). Canadian Entomologist 40: 25-30.

Bird H. 1911. New histories and species in *Papaipema* and *Hydroecia*. Canadian Entomologist 48(2): 37-47.

Bird H. 1913. On the larval habits of two species of *Oligia* (Lepidoptera, Noctuidae). Insecutor Inscitiae Menstruus, 1: 123-124.

Bird H. 1915a. New histories and species in *Papaipema* Sm. (Lepidoptera). Canadian Entomologist 47: 109-115.

Bird H. 1915b. New histories and species in *Papaipema* Sm. (Lepidoptera). Canadian Entomologist 47: 145-151.

Bird H. 1917. New histories and species in *Papaipema* Sm. (Lepidoptera) no. 19. Canadian Entomologist 49: 121-128.

Bird H. 1923. New histories and species in *Papaipema* Sm. (Lepidoptera) no. 22. Canadian Entomologist 55: 106-109.

Bird, R.D. 1927. Notes on insects bred from native and cultivated fruit trees and shrubs of southern Manitoba. Canadian Entomologist 59: 124-128.

Bird, H. and F.M. Jones A new Papaopema from Delaware (Lepidoptera, Noctuidae). Canadian Entomologist 53: 137-139.

Bissell, T. L. 1945. Lesser cornstalk borer. pp. 63-64 In: Annual Report, 1944-1945. Georgia Agricultural Experimental Station Georgia.

Blackman, M.W. and M.S. Stage. 1924. On the succession of insects living in the bark and wood, dead and decaying hickory. Technical publication of the New York State College of Forestry at Syracuse University 24(17): 3-240, plates I-XIV.

Blackmore, E.H. 1918. Further additions to the list of British Columbia Geometridae. Journal of the Entomological Society of British Columbia 8: 14-20.

Blakeslee, E.B. 1915. American plum borer. Bulletin of the U.S. Department of Agriculture 261.

Blanchard, A. 1963. Contribution to the life history of *Schoenobius maximellus* (Pyralididae). Journal of the Lepidopterists’ Society 17: 234-235.

Blanchard, A. 1966. A new species of *Glaucina* (Geometridae) from Texas. Journal of the Lepidopterists' Society 20(4): 247-250.

Blanchard, A. 1968. New moths from Texas (Noctuidae, Tortricidae). Journal of the Lepidopterists' Society 22(3): 133-145.

Blanchard, A. 1970. Observations on some Phycitinae (Pyralidae) Texas with descriptions of two new species. Journal of the Lepidopterists’ Society 24(4): 249-255.

Blanchard, A. 1971a. A new species in the genus *Ursia* Barnes & McCunnough (Lepidoptera: Notodontidae). Proceedings of the Entomological Society of Washington 73(3): 303-305.

Blanchard, A. 1971b. Notes on three species of *Heterocampa* Doubleday with description of a new species (Lepidoptera: Notodontidae). Proceedings of the Entomological Society of Washington 73(3): 249-254.

Blanchard, A.1973a. Two new species of Phycitinae from Texas, with description of two new genera (Pyralidae). Journal of the Lepidopterists' Society 27(3): 219-225.

Blanchard, A. 1973b. Record and illustration of some interesting moths flying in Texas (Sphingidae, Ctenuchidae, Noctuidae, Notodontidae, Geometridae, Pyralidae, Cossidae). Journal of the Lepidopterists' Society 27(2): 103-109.

Blanchard, A. 1973c. A new species of the genus *Glenoides* McDunnough (Geometridae). Journal of the Lepidopterists' Society 27(2): 141-143.

Blanchard, A. 1975a. A new phycitine genus and species (Pyraloidea). Journal of the Lepidopterists’ Society 29: 95-97.

Blanchard, A. 1975b. A new Schoenobine genus and species (Pyraloidea). Journal of the Lepidopterists’ Society 29: 98-101.

Blanchard, A. 1976a. A new species of the genus *Bertelia* B. & McD. (Pyralidae). Journal of the Lepidopterists' Society 30(3): 211-213.

Blanchard, A. 1976b. Two new species of Phycitine moths with description of a new genus (Pyralidae). Journal of the Lepidopterists' Society 30(4): 284-288.

Blanchard, A. 1978. The status of *Ollia parvella* Dyar: Redescription in a new genus (Pyralidae). Journal of the Lepidopterists’ Society 32: 103-106.

Blanchard, A. 1979a. New status for *Epiblema minutana* (Kearfott) and new species of *Epiblema* Hubner and *Sonia* Heinrich (Tortricidae). Journal of the Lepidopterists' Society 33(3): 179-188.

Blanchard, A.1979b. Five new species of the tribe Eucosmini (Tortricidae). Journal of the Lepidopterists' Society 33(4): 209-215

Blanchard, A. 1980. A new species of the genus *Peoria* Ragonot (Pyralidae). Journal of the Lepidopterists' Society 34(4): 338-339.

Blanchard, A. and D.C. Ferguson. 1975. *Rostrolaetilia* - A new North American genus of the subfamily Phycitinae, with descriptions of seven new species (Pyralidae). Journal of the Lepidopterists’ Society 29(3): 131-150.

Blanchard, A., and J.G. Franclemont. 1982a. *Marilopteryx carancahua*, a new genus and new species from East Texas (Lepidoptera: Noctuidae: Hadeninae). Proceedings of the Entomological Society of Washington 84(2): 270-276.

Blanchard, A., and J.G. Franclemont. 1982b. A new species of *Zale* Hubner from Texas and New Mexico (Lepidoptera: Noctuidae: Catocalinae). Proceedings of the Entomological Society of Washington 84(1): 134-137.

Blanchard, A. and E.C. Knudson. 1981a. Two new species of *Eucosma* Hubner (Tortricidae) from Texas. Journal of the Lepidopterists’ Society 35(3): 173-178.

Blanchard, A. and E.C. Knudson. 1981b. A new species of *Ozamia* Ragonot (Pyralidae) from Texas. Journal of the Lepidopterists' Society 35(3): 233-235.

Blanchard, A. and E.C. Knudson. 1981c. Two new species of the tribe Eucosmini (Tortricidae). Journal of the Lepidopterists' Society 35(3): 169-172.

Blanchard, A. and E.C. Knudson. 1982. A new species of *Symmetrischema* Povolny (Lepidoptera: Gelechiidae) from Texas. Proceedings of the Entomological Society of Washington 84(3): 628-631.

Blanchard, A. and E.C. Knudson. 1983a. Two new species of Pyralidae (Lepidoptera) from Texas. Proceedings of the Entomological Society of Washington 85: 59-63.

Blanchard, A. and E.C. Knudson. 1983b. A new species of *Dioryctria* Zeller (Lepidoptera: Pyralidae) from Texas. Proceedings of the Entomological Society of Washington 85: 116-120.

Blanchard, A. and E.C. Knudson. 1983c. A new species of *Psorosina* Dyar (Lepidoptera: Pyralidae) from Texas. Proceedings of the Entomological Society of Washington 85: 619-621.

Blanchard, A. and E.C. Knudson. 1983d. New North American species of Eucosimi (Lepidoptera: Tortricidae). Proceedings of the Entomological Society of Washington 85(4): 845-852.

Blanchard, A. and E.C. Knudson. 1984a. A new species of *Hypomecis* Hübner (Lepidoptera: Geometridae) from Texas and Florida. Proceedings of the Entomological Society of Washington 86(2): 291-294.

Blanchard, A. and E.C. Knudson. 1984b. A new *Stibadium* from Texas and a rediescription of *Stiriodes edentates* (Grote) (Noctuidae: Lepidoptera). Proceedings of the Entomological Society of Washington 86(2): 346-348.

Blanchard, A. and E.C. Knudson. 1984c. Three new tortricids (Lepidoptera) from Texas. Proceedings of the Entomological Society of Washington 86(2): 446-451.

Blanchard, A. and E.C. Knudson. 1984d. A new species of *Tripudia* Grote (Lepidoptera: Noctuidae) from western Texas. Proceedings of the Entomological Society of Washington 86(3): 639-642

Blanchard, A. and E.C. Knudson. 1985a. New US records and other interesting moths from Texas. Journal of the Lepidopterists’ Society 39(1): 1-8.

Blanchard, A. and E.C. Knudson. 1985b. A new species of *Bucculatrix* Zeller Lepidoptera Lyonetiidae from Texas. Proceedings of the Entomological Society of Washington 87(2): 371-374.

Blanchard. A. and E.C. Knudson. 1985c. The *Eupithecia* (Lepidoptera, Geometridae) of Texas, with the description of a new species. Proceedings of the Entomological Society of Washington 87(3): 662-674.

Blanchard, A. and E.C. Knudson. 1985d. Two new Phycitinae (Lepidoptera: Pyralidae) from Texas. Proceedings of the Entomological Society of Washington 87: 475-479.

Blanchard, A. and E.C. Knudson. 1985f. *Psychonoctua masoni* (Schaus), new combination (Lepidoptera: Cossidae: Zeuzerinae), redescription and first records from Texas and USA. Proceedings of the Entomological Society of Washington, 87(2): 426-431.

Blanchard, A. and E.C. Knudson. 1985g. Two new species of *Hexorthodes* (Lepidoptera: Noctuidae) from Texas and Arizona. Proceedings of the Entomological Society of Washington 87(4) 777-782.

Blanchard, A. and E.C. Knudson. 1985h. New species of Phycitinae (Lepidoptera, Pyralidae) from Texas, with description of a new genus. Proceedings of the Entomological Society of Washington 87(1): 231-238.

Blanchard, A. and E.C. Knudson. 1985i. *Ethmia angustalatella* Powell (Lepidoptera, Oecophoridae): Description of the female and first U.S. records. Proceedings of the Entomological Society of Washington 87: 680-68l.

Blanchard, A. and E.C. Knudson. 1986a. Four new moths from Texas (Lepidoptera, Geometridae, Noctuidae). Proceedings of the Entomological Society of Washington 88(1): 134-141.

Blanchard, A. and E.C. Knudson. 1986b. A new *Chlamydastis* (Oecophoridae, Lepidoptera) from Texas. Proceedings of the Entomological Society of Washington 88(1): 185-188.

Blanchard, A., J.E. Gillaspy, D.F. Hardwick, and J.H. Johnson. 1985. Checklist of Lepidoptera of the Rob and Bessie Welder Wildlife Refuge near Sinton, Texas. The Southwestern Entomologist 10(3): 195-214.

Bland, K.P., A.M. Emmet, R.J. Heckford and T. Rutten, 2002a. Anomologinae. pp. 67-117 *in* A.M. Emmet and J.R. Langmaid (eds.), The Moths and Butterflies of Great Britain and Ireland, Volume 4, part 2, Gelechiidae. Harley Books, Colchester, Essex, England.

Bland, K.P., M.F.V. Corley, A.M. Emmet, R.J. Heckford, P. Huemer, J.R. Langmaid, S.M. Palmer, M.S. Parsons, L.M. Pitkin, K. Sattler and A.N.B. Simpson, 2002b. Gelechiinae. pp. 118-203 *in* A.M. Emmet and J.R. Langmaid (eds.), The Moths and Butterflies of Great Britain and Ireland, Volume 4, part 2, Gelechiidae. Harley Books, Colchester, Essex, England.

Bland, K.P., R.J. Heckford, and J.R. Langmaid, 2002c. Anacampsinae. pp. 204-220 *in* A.M. Emmet and J.R. Langmaid (eds.), The Moths and Butterflies of Great Britain and Ireland, Volume 4, part 2, Gelechiidae. Harley Books, Colchester, Essex, England.

Bland, K.P. and A.M. Emmet. 2002. Pexicopiinae. pp. 239-256 *in* A.M. Emmet and J.R. Langmaid (eds.), The Moths and Butterflies of Great Britain and Ireland, Volume 4, part 2, Gelechiidae. Harley Books, Colchester, Essex, England.

Błeszyński, S. 1963. Studies on the Crambidae (Lepidoptera). Part 41. On some tropical Crambidae with descriptions of new genera and species. Acta Zoologica Cracoviensia 8: 133-181.

Bleszynski, S. 1965. Crambinae. Microlepidoptera Palaearctica 1. Verlag Georg Fromme & Co., Wien, Germany.

Bleszynski, S. 1970. A revision of the world species of Chilo Zincken (Lepidoptera: Pyralidae). Bulletin of the British Museum Natural History. Entomology 25(4): 99-195.

Blomefield, T.L. and H. Geertsema. 1990. First record of the Oriental fruit moth, *Cydia molesta* (Lepidoptera: Tortricidae: Olethreutinae), a serious pest of peaches, in South Africa. Phytophylactica 22: 355-357.

Bodenham, J. and R.E. Stevens. 1981. Insects associated with second-year ponderosa pine cones, Larimer and Boulder counties, Colorado. The Southwestern Naturalist 26(4): 375-378.

Boisduval, J.B.A.E., [1875] 1874. *Sphingides, Sésiides, Castnides*, vol. 1. Librairie Encyclopédique de Roret, Paris, 568 pp. (Boisduval, J.B.A.E. and A. Guenée (eds.) Histoire naturelle des insectes. Species général des Lépidoptères Hétérocères).

Bolte, K.B. 1977. A new species of *Eupithecia* (Lepidoptera: Geometridae) from Alaska. The Canadian Entomologist 109(0): 1019-1020.

Bolte, K.B. 1990. Guide to the Geometridae of Canada (Lepidoptera).6. Subfamily Larentiinae.1. Revision of the genus *Eupithecia*. Memoirs of the Entomological Society of Canada 151: 1-253.

Bordelon, C.W. Jr. 1996. 1995 Season Summary. Zone 6 South Central. Oklahoma, Texas, Arkansas, Louisiana. News of the Lepidopterists’ Society 52(S1): 35-41.

Bordelon, C.W. Jr. 1997. 1996 Season Summary. Zone 6 South Central. Oklahoma, Texas, Arkansas, Louisiana. News the Lepidopterists’ Society 39: 39-53.

Bordelon, C.W. Jr. 1998. 1997 Season Summary. Zone 6 South Central. Oklahoma, Texas, Arkansas, Louisiana. News the Lepidopterists’ Society 38(2): 35-45.

Bordelon, C.W. Jr. 1999. 1998 Season Summary. Zone 6 South Central. Oklahoma, Texas, Arkansas, Louisiana. News the Lepidopterists’ Society 39: 41-53.

Bordelon, C.W. Jr. 2000. 1999 Season Summary. Zone 6 South Central, Oklahoma, Texas, Arkansas, Louisiana. News of the Lepidopterists’ Society 42(S1): 33-50.

Bordelon, C.W. Jr. 2001. 2000 Season Summary. Zone 6 South Central, Oklahoma, Texas, Arkansas, Louisiana. News of the Lepidopterists’ Society 43(2): 28-38.

Bordelon, C.W. Jr. 2002. 2001 Season Summary. Zone 6 South Central, Oklahoma, Texas, Arkansas, Louisiana. News of the Lepidopterists’ Society 44(S1): 28-41.

Bordelon, C.W. Jr. 2003. 2002 Season Summary. Zone 6 Texas, Texas. News of the Lepidopterists’ Society 45(S1): 37-41.

Bordelon, C.W. Jr. 2004. 2003 Season Summary. Zone 6 Texas, Texas. News of the Lepidopterists’ Society 46(S1): 41-49.

Bordelon, C.W. Jr. 2005. 2004 Season Summary. Zone 6 Texas, Texas. News of the Lepidopterists’ Society 47(S1): 41-58.

Bordelon, C.W. Jr. 2006. 2005 Season Summary. Zone 6 Texas, Texas. News of the Lepidopterists’ Society 48(S1): 56-68.

Bordelon, C.W. Jr. 2007. 2006 Season Summary. Zone 6 Texas, Texas. News of the Lepidopterists’ Society 49(S1): 63-77.

Bordelon, C.W. Jr. 2008. 2007 Season Summary. Zone 6 Texas, Texas. News of the Lepidopterists’ Society 50(S1): 83-98.

Bordelon, C.W. Jr. 2009. 2008 Season Summary. Zone 6 Texas, Texas. News of the Lepidopterists’ Society 51(S1): 72-82.

Bordelon, C.W. Jr. 2010. 2009 Season Summary. Zone 6 Texas, Texas. News of the Lepidopterists’ Society 52(S1): 86-95.

Bordelon, C.W. Jr. 2011. 2010 Season Summary. Zone 6 Texas, Texas. News of the Lepidopterists’ Society 53(S1): 86-100.

Bordelon, C.W. Jr. 2012. 2011 Season Summary. Zone 6 Texas, Texas. News of the Lepidopterists’ Society 54(S1): 71-82.

Bordelon, C.W. Jr. 2013. 2012 Season Summary. Zone 6 Texas, Texas. News of the Lepidopterists’ Society 55(S1): 59-86.

Bordelon, C.W. Jr. 2014. 2013 Season Summary. Zone 6 Texas, Texas. News of the Lepidopterists’ Society 56(S1): 73-118.

Bordelon, C.W. Jr. 2015. 2014 Season Summary. Zone 6 Texas, Texas. News of the Lepidopterists’ Society 57(S1): 99-143.

Bordelon, C.W. Jr. 2016. 2015 Season Summary. Zone 6. Texas. News of the Lepidopterists' Society 58(S1): 99-153.

Bordelon, C.W. Jr. and E. Knudson. 1998. Abundant Occurrence of the "rare" *Vitacea admiranda* (Hy. Edw.) (Sesiidae) in the coastal Bend of Texas. News of the Lepidopterists' Society 40(1): 31.

Bordelon, C.W. Jr. and E. Knudson. 2000. New records of Lepidoptera from Texas and the USA, and illustrations of other interesting species. News the Lepidopterists’ Society 42: 3-7, 19.

Bordelon, C.W. Jr. and E. Knudson. 2009a. Reprise: *Gonodonta* (Fruit-piercing Moths) (Noctuidae, Calpinae) from Texas with yet another new USA record from South Texas. News of the Lepidopterists’ Society 51(4): 118-119.

Bordelon, C.W. Jr. and E. Knudson. 2009b. The genus *Hamadyas* (Nymphalidae: Biblidinae: Aoroniini) in Texas, with a report of a new-record for Texas and the USA. News of the Lepidopterists' Society 51(1): 6-9.

Bordelon, C.W. Jr. and E. Knudson. 2011a. Some new U.S.A. records and other interesting Pyraloidea from Texas. News of the Lepidopterists’ Society 53(2): 44-45.

Bordelon, C.W. Jr. and E. Knudson. 2011b. *Ebrietas* Godman & Salvin 1896: A new hesperiid genus (Pyrginae: Erynnini) for the USA from South Texas. News of the Lepidopterists’ Society 53(1): 25.

Borth, R.J. and H.L. Kons. 2016b. A new species of *Catocala* (Lepidoptera: Noctuidae) from California. Bulletin of the Peabody Museum of Natural History 57(2): 241-252.

Boscoe, R. and B. Nall. 2012. Life history notes on Evans’ Skipper, *Panoquina evansi* (Hesperiidae: Hesperiinae). News of the Lepidopterists’ Society 64(2): 72-73

Bottimer, L.J. 1926. Notes on some Lepidoptera from eastern Texas. Journal of Agricultural Research 33(9): 797-819.

Bottimer, L.J. 1942. Brief notes on two recently described species of *Mompha* (Lepidoptera). Bulletin of the Southern California Academy of Sciences 41(1): 48.

Boughton, A.J. and R.W. Pemberton, R. 2009. Establishment of an imported natural enemy, *Neomusotima conspurcatalis* (Lepidoptera: Crambidae) against an invasive weed, Old World climbing fern, *Lygodium microphyllum*, in Florida. Biocontrol science and technology 19(7): 769-772.

Boughton, A.J. and R.W. Pemberton. 2012. Biology and reproduction parameters of the brown Lygodium moth, *Neomusotima conspurcatalis* - a new biological control agent of Old World climbing fern in Florida. Environmental Entomology 41: 308-316.

Bourchier, R.S., N. Cappuccino, A. Rochette, J. des Rivières, S.M. Smith, L. Tewksbury, and R. Casagrande. 2019. Establishment of Hypena opulenta (Lepidoptera: Erebidae) on *Vincetoxicum rossicum* in Ontario, Canada. Biocontrol Science and Technology 29: 917-923. DOI: 10.1080/09583157.2019.1608511.

Bower, H.M. 1961. Foodplants of the Sphingidae in Wisconsin. Journal of the Lepidopterists’ Society 15: 64.

Bower, H.M. 1963. Additional note on the foodplant of *Sphinx kalmiae*. Journal of the Lepidopterists’ Society 17: 36.

Bowles, G.J. 1871. List of Lepidoptera taken at Quebec. Canadian Entomologist 3: 144-146.

Bowman, K. 1951. An annotated list of the Lepidoptera of Alberta. Canadian Journal of Zoology 29: 121-165.

Box, H E. 1931. The Crambine genera *Diatraea* and *Xanthopherne* (Lep., Pyral.). Bulletin of Entomological Research 22(1): 1-50.

Box, H.E. 1935. New records and three new species of American *Diatraea* (Lep.: Pyral.). Bulletin of Entomological Research 26(3): 323-333.

Box, H.E. 1955. New Crambine genera allied to *Diatraea* guilding (Lepidoptera: Pyralidae). III. Systematic Entomology 24(11-12): 197-200.

Bradley, J. D. 1953. Some important species of genus *Cryptophlebia* Walsingham, 1899, with descriptions of three new species (Lepidoptera: Olethreutidae). Bulletin of Entomological Research 43: 679-689.

Bradley, J. D., and H. E. Hinton, 1955. A new genus of Tineinae (Lep., Tineidae) from North America. Entomological Monthly Magazine 91: 307-308.

Bradley, J. D., W.G. Tremewan, and A. Smith. 1973. British Tortricoid moths. Cochylidae and Tortricidae: Tortricinae. The Ray Society, London. 251 pp.

Bradley, J.D., W.G. Tremewan and A. Smith, 1979. British Tortricoid Moths. Tortricidae: Olethreutinae. The Ray Society, London. 336 pp.

Braga, S.M.P., M.M. Dias, and A.M. Penteado-Dias. 2001. Aspectos bionômicos de *Eois tegularia* (Guenée) e *Eois glauculata* (Walker) (Lepidoptera, Geometridae, Larentiinae) e seus parasitóides. Revista Brasileira de Zoologia, 18(3), 837-840.

Bragard, C., K. Dehnen-Schmutz, F. Di Serio, P. Gonthier, M.-A. Jacques, J.A.J. Miret, A. Fejer Justesen, A. MacLeod, C.S. Magnusson, J.A. Navas-Cortes, S. Parnell, R. Potting, P.L. Reignault, H.-H. Thulke, W. Van der Werf, A.V. Civera, J. Yuen, L. Zappala, J.-C. Gregoire, V. Kertesz and P. Milonas. 2019. Pest categorisation of non-EU Acleris spp. EFSA Journal 17(10); 5856; 1-37.

Brambila, J. and I. Stocks. 2010. Pest Alert. The European pepper moth, *Duponchelia fovealis* Zeller (Lepidoptera: Crambidae), a Mediterranean pest moth discovered in Central Florida. Florida Department of Agriculture and Consumer Services, Division of Plant Industry DACS-P-01752. FDACS - Division of Plant Industry. http://www.freshfromflorida.com/pi/pest_alerts/pdf/duponchelia_fovealis.pdf (29 September 2011).

Braun, A.F. 1908. Revision of the North American species of the genus *Lithocolletis* Hübner. Transactions of the American Entomological Society 34(4): 269-357.

Braun, A. F. 1912. Notes on some North American Tineina. Canadian Entomologist 44(5): 159-161.

Braun, A. F. 1914. Notes on North American Tineina, with descriptions of new species (Lepid.). Entomological News 25: 113-117.

Braun, A.F. 1916. New species of microlepidoptera. The Canadian Entomologist 48(4): 138-141.

Braun, A.F. 1916b. Notes on *Lithocolletis* with descriptions of new species (Lep.). Entomological News 27: 82-84.

Braun, A.F. 1917. Nepticulidae of North America. Transactions of the American Entomological Society 43: 155-209.

Braun, A.F. 1918a. New species of microlepidoptera. The Canadian Entomologist 50(7): 229-236.

Braun, A.F. 1918b. New genera and species of Lyonetiidae (Microlepidoptera). Entomological News 29(7): 245-251.

Braun, A.F. 1920a. Notes on *Elachista* with description of new species (Microlepidoptera). Ohio Journal Science 20(5): 167-172.

Braun, A.F. 1920b. New species of *Scythris* (Microlepidoptera). Canadian Entomologist 52(2): 40-41.

Braun, A.F. 1921a. Two weeks collecting in Glacier Nation Park. Proceedings of the Academy of Natural Sciences of Philadelphia 73: 1-23.

Braun, A.F. 1921b. Notes on Microlepidoptera with descriptions of new species. Entomological News 32: 8-18.

Braun, A.F. 1923a. Superfamily Nepticuloidea, Family 5. Nepticulidae. *and* 8. Cremastobombycia *and* 9. Lithocolletis. Pp. 79-98, 185-186, 186-202 *in* W.T.M. Forbes. Lepidoptera of New York and neighboring states. Part I. Primitive forms, Microlepidoptera, Pyraloids, Bombyces. Memoirs, Cornell University Agricultural Experimental Station, No. 68.

Braun, A.F. 1923b. Microlepidoptera: Notes and a new species. Transactions of the American Entomological Society 49: 115-127.

Braun, A.F. 1923c. Expedition of the California Academy of Sciences to the Gulf of California in 1921. Proceedings of the California Academy of Sciences 7(10): 117-122.

Braun, A.F. 1925a. Microlepidoptera of northern Utah. Transactions of the American Entomological Society 51(3):183-226.

Braun, A.F. 1925b. Some undescribed Microlepidoptera and notes on life histories. Transactions of the American Entomological Society 51: 13-17.

Braun, A.F. 1930. Notes and new species of Microlepidoptera from the Mineral Springs region of Adams County, Ohio. Transactions of the American Entomological Society 56(1): 1-17.

Braun, A.F. 1935. Notes and new species of Microlepidoptera. Transactions of the American Entomological Society 61: 45-52.

Braun, A.F. 1939. Notes and new species: Gracilariidae (Microlepidoptera). Annals of the Entomological Society of America 32: 279-287.

Braun, A. 1940a. Aster and goldenrod seed-feeding species of *Coleophora* (Lepidoptera). The Canadian Entomologist 72(9): 178-182.

Braun, A. 1940b. Notes and new species in the Yponomeutoid Group (Microlepidoptera). Transactions of the American Entomological Society 66(4): 273-282.

Braun, A.F. 1942. A remarkable new Heliodinid (Microlepidoptera). Annals of the Entomological Society of America 35(4): 373-378.

Braun, A.F. 1948. Elachistidae of North America. Memoirs of the American Entomological Society 13: 1-110.

Braun, A.F. 1951. The *Aesculus*-feeding species of *Exartema* with description of a new species (Lepidoptera, Eucosmidae). The Ohio Journal of Science 51(6): 353-357.

Braun, A.F. 1963. The Genus *Bucculatrix* in America North of Mexico (Microlepidoptera). Memoirs of the American Entomological Society 18: 1-208.

Braun, A.F. 1972. Tischeriidae of America North of Mexico (Microlepidoptera). Memoirs of the American Entomological Society 28: 1-148.

Brechlin, R. and F. Meiste, 2011. Neue Taxa der Gattung Automeris Huebner [1819]. Entomo-Satsphingia 4(1): 5-89.

Brechlin, R. and E. Van Schayck. 2016. Einige Anmerkungen zur Gattung *Coloradia* Blake, 1863 aus den USA mit Beschreibung einer neuen Art (Lepidoptera: Saturniidae). Entomo-Satsphingia 9(2): 5-9.

Brehm, G. 2002. Diversity of geometrid moths in a montane rainforest in Ecuador. PhD Dissertation. University of Bayreuth, Bayreuth, Germany.

Brehm, G., L. Murillo-Ramos, P. Sihvonen, A. Hausmann, B.C. Schmidt, E. Ounap, A. Moser, R. Mörtter, D. Bolt, F. Bodner, A. Lindt, L.E. Parra, N. Wahlberg, 2019. New World geometrid moths (Lepidoptera: Geometridae): Molecular phylogeny, biogeography, taxonomic updates and description of 11 new tribes. Arthropod Systematics & Phylogeny 77(3): 457-486.

Bretherton, R.F., B. Goater and R.I. Lorimer, 1979. Noctuidae: Noctuinae to Hadeniinae. pp. 120-280 *in* J. Heath and A.M. Emmet (eds.), The Moths and Butterflies of Great Britain and Ireland, Volume 9, Sphingidae - Noctuidae (Noctuinae to Hadeniinae). Curwen Books, London, England.

Bretherton, R.F., B. Goater and R.I. Lorimer, 1983. Noctuidae: Cuculliinae to Hypeninae. pp. 36-413 *in* J. Heath and A.M. Emmet (eds.), The Moths and Butterflies of Great Britain and Ireland, Volume 10, Noctuidae (Cuculliinae to Hypeninae) and Agaristidae. Harley Books, Colchester, Essex, England.

Brévignon, C. 2009. Nouvelles observations sur le genre *Junonia* en Guyane Française. (Lepidopterar: Nymphalidae) Première Partie. Labillionea 109: 3-7.

Britt, K.E., T.P. Kuhar, W. Cranshaw, C.T. McCullough, S.V. Taylor, B.R. Arends, H. Burrack, M. Pulkoski, D. Owens, T.A. Tolosa, S. Zebelo, K.A. Kesheimer, O.S. Ajayi, M. Samuel-Foo, J.A. Davis, N. Arey, H. Doughty, J. Jones, Marguerite Bolt, Bradley J. Fritz, J.F. Grant, J. Cosner, and M. Schreiner. 2021. Pest management needs and limitations for corn earworm (Lepidoptera: Noctuidae), an emergent key pest of hemp in the United States. Journal of Integrated Pest Management 12(21): 34; 1-11.

Britton, W.E. 1920. Checklist of insects of Connecticut. Connecticut Geological and Natural History Survey Bulletin Number 31. 397 pp.

Brock, J.P. and K. Kaufman. 2003. Field Guide to Butterflies of North America. Houghton Mifflin Harcourt, Boston, MA. 392 pp.

Brockerhoff, E.G. and M. Kenis. 1996. Parasitoids associated with *Cydia strobilella* (L.) (Lepidoptera: Tortricidae) in Europe, and considerations for their use for biological control in North America. Biological Control 6(2): 202-214.

Brodie, H.J. 1929. Notes on the early stages of Anisota *Manitobensis* (Lepid.). The Canadian Entomologist 61(5): 98-100.

Brodie, W. 1882a. Food plants of *Platysamia cecropia*. Papilio 2: 32-33.

Brodie, W. 1882b. Food plants of *Telea Polyphemus* in the county York, Ontario. Papilio 2: 32-33.

Brou Jr., V.A. 1985. A new species of *Catocala* (Lepidoptera: Noctuidae) from the Gulf South, USA. Proceeding of the Entomological Society of Washington 87(4): 889-892.

Brou Jr., V.A. 1988. A new species of *Catocala* from the southeast United States. Journal of the Lepidopterists' Society 42(2): 116-119.

Brou Jr., V.A. 1993. Range extension of the moth *Parapoynx diminutalis* Snellen (Lepidoptera: Pyralidae. Southern Lepidopterists’ News 15: 33-34.

Brou Jr., V.A. 1994a. A new species of *Lapara* (Sphingidae) from southeastern United States. Journal of the Lepidopterits Society 48(1): 51-57.

Brou Jr., V.A. 1994b. A new US Pyralidae species record. Southern Lepidopterists’ News 16(3): 26.

Brou Jr., V.A. 1996a. Westward range extension for *Franclemontia interrogans* (Walker) a rarely encountered and little known species. Southern Lepidopterists’ News 18: 17.

Brou Jr., V.A. 1997. A new *Chaetaglaea* from the southeastern United States (Noctuidae: Cuculliinae). Journal of the Lepidopterists Society 51(2): 135-138.

Brou Jr., V.A. 2001. The genus *Heliothis* Ochsenheimer (Noctuidae) in Louisiana. News of the Southern Lepidopterists’ Society 23(2): 15-17.

Brou Jr., V.A. 2002a. *Syngamia florella* (Stoll) (Pyralidae) in Louisiana. News of the Southern Lepidopterists’ Society 24(2): 24.

Brou Jr., V.A. 2002b. *Catocala ilia* (Cramer) and form *umbrosa* in Louisiana. News of the Southern Lepidopterists’ Society 24(3): 48-50.

Brou Jr., V.A. 2002c. The *Euzophera* (Pyralidae: Phycitinae) species of Louisiana. News of the Southern Lepidopterists’ Society 24(3): 51-52.

Brou Jr., V.A. 2002d. *Hemileuca maia* (Drury) in Louisiana. News of the Southern Lepidopterists’ Society 24(3): 56.

Brou Jr., V.A. 2002e. The *Dioryctria* (Pyralidae) species of Loiusiana. News of the Southern Lepidopterists’ Society 24(3): 58-59.

Brou Jr., V.A. 2002f. Type designation of *Catocala umbrosa* Brou. News of the Southern Lepidopterists’ Society 24(4): 85-86.

Brou Jr., V.A. 2002g. *Catocala grynea* (Cramer) in Louisiana. News of the Southern Lepidopterists’ Society 24(4): 93.

Brou Jr., V.A. 2002h. *Catocala ultronia* (Hbn.) in Louisian*a*. News of the Southern Lepidopterists’ Society 24(4): 94.

Brou Jr., V.A. 2002i. *Sphacelodes vulnerarja* (Hbn.) (Geometridae) in Louisiana. News of the Southern Lepidopterists’ Society 24(1): 7.

Brou Jr., V.A. 2002j. The occurrence of *Nepytia semiclusaria* (Walker) in Louisiana. News of the Southern Lepidopterists’ Society 24(1): 8.

Brou Jr., V.A. 2003a. *Metaxaglaea* of Louisiana. Southern Lepidopterists' News 25(2): 41-42.

Brou Jr., V.A. 2003b. *Schinia trifascia* Hubner in Louisiana. Southern Lepidopterists' News 25(2): 48

Brou Jr., V.A. 2003c. The occurrence of *Bagisara brouana* Ferguson in southeast Louisiana. Southern Lepidopterists' News 25: 8.

Brou Jr., V.A. 2003d. *Schinia bimatris* (Harvey) in Louisiana. Southern Lepidopterists' News 25: 7.

Brou Jr., V.A. 2003e. *Ascalapha odorata* (L.) and *Thysania zenobia* (Cram.) in Louisiana. Southern Lepidopterists' News 25(3): 91

Brou Jr., V.A. 2004a. *Symmerista albifrons* (J. E. Smith) in southeast Louisiana. Southern Lepidopterists’ News 26(4): 103.

Brou Jr., V.A. 2004b. The *Pyreferra* of Louisiana. Southern Lepidopterists’ News 26(4): 104.

Brou Jr., V.A. 2004c. Two new species of *Baileya* Grote (Nolidae: Risobinae) from the southeastern United States. Journal of the Lepidopterists Society 58(2): 94-99.

Brou Jr., V.A. 2004d. *Eutrapela clemataria* (J. E. Smith) in Louisiana. Journal of the Lepidopterists Society 26(3): 75.

Brou Jr., V.A. 2004e. *Thysanopyga intractata* (Wlk.) in LouisianaJournal of the Lepidopterists Society 26(3): 79.

Brou Jr., V.A. 2004f. *Artace cribraria* (Ljungh) in Louisiana. Journal of the Lepidopterists Society 26(3): 80.

Brou Jr., V.A. 2005. *Iridopsis defectaria* (Gn.) in Louisiana. Southern Lepidopterists’ News 27(4): 103.

Brou Jr., V.A. 2006a. A new US record for the tropical fruit-piercing moth *Eudocima serpentifera* (Walker, [1858]). Southern Lepidopterists’ News 28: 105-108.

Brou Jr., V.A. 2006b. *Melipotis fasciolaris* (Hubner) in Louisiana. Southern Entomologist’s News 28: 16.

Brou Jr., V.A. 2006c. *Melipotis indomita* Walker in Louisiana. Southern Entomologist’s News 28(4): 116.

Brou Jr., V.A. 2006d. *Holomelina opella* (Grote) in Louisiana. Southern Entomologist’s News 28(4): 119.

Brou Jr., V.A. 2006e. *Panula inconstans* Guenee, new state record for Louisiana. Southern Entomologist’s News 28(3): 82.

Brou Jr., V.A. 2006f. *Gramma virgo* (L.) in Louisiana. Southern Entomologist’s News 28(4): 136.

Brou Jr., V.A. 2006g. *Epidroma fergusoni* (Solis) in Louisiana. Southern Entomologist’s News 28(3): 81.

Brou Jr., V.A. 2006h. *Catocala alabamae* Grote in Louisiana. Southern Entomologist’s News 28(3): 83-84.

Brou Jr., V.A. 2006i. *Harrisimemna trisignata* (Walker) in Louisiana. Southern Entomologist’s News 28(1): 7.

Brou Jr., V.A. 2006j. *Catocala micronympha* Guenee in Louisiana. Southern Entomologist’s News 28(1): 26.

Brou Jr., V.A. 2006k. *Metarranthis obfirmaria* (Hbn.) in southeast Louisiana. Southern Entomologist’s News 28(1): 32.

Brou Jr., V.A. 2007a. *Epitausa prona* (Moschler, 1880) in Louisiana. Southern Lepidopterists News 29(4): 153-155.

Brou Jr., V.A. 2007b. *Helvibotys subcostalis* (Dyar, 1912) and *Helvibotys pucilla* (Druce, 1895) in Louisiana. Lepidopterists' News 29(4): 121-122.

Brou Jr., V.A. 2008a. *Catocala carissim*a Hulst (Lepidoptera: Noctuidae) 1880 in Louisiana. Southern Entomologist’s News 30(1): 15-16.

Brou Jr., V.A. 2008b. Update on *Chaetaglaea fergusoni* Brou, 1997 (Lepidoptera: Noctuidae) in Louisiana. Southern Entomologist’s News 30(1): 17.

Brou Jr., V.A. 2008c. *Satyrium kingi* (Klots & Clench, 1952) in Louisiana. Southern Entomologist’s News 30(1): 18-19.

Brou Jr., V.A. 2008d. *Exyra semicrocea* (Guenee) in Louisiana. Southern Entomologist’s News 30(1): 19-20.

Brou Jr., V.A. 2008e. Spotlight on rearing: *Apantesis nais* (Drury) (Lepidoptera: Arctiidae) in Louisiana. Southern Entomologist’s News 30(1): 21-23.

Brou Jr., V.A. 2008f. *Fania nona* (Strecker, 1876) (Lepidoptera: Cossidae) in Louisiana. Southern Entomologist’s News 30(1): 40.

Brou Jr., V.A. 2008g. *Oreta rosea* (Walker, 1855) (Lepidoptera: Drepanidae) in Louisana. Southern Entomologist’s News 30(2): 41-42.

Brou Jr., V.A. 2008h. *Dryocampa rubicunda* (F.) in Louisiana. Southern Entomologist’s News 30(2): 51-52.

Brou Jr., V.A. 2008i. Variations of *Phoberia atomaris* Hubner (Lepidoptera: Noctuidae) in Louisiana. Southern Entomologist’s News 30(2): 57-58.

Brou Jr., V.A. 2008j. *Acrolophus mycetophagus* Davis, 1990 (Lepidoptera: Acrolophidae) in Louisiana. Southern Entomologist’s News 30(2): 59.

Brou Jr., V.A. 2008k. The genus P*hytometra* Haworth, 1809 (Lepidoptera: Noctuidae) in Louisiana. Southern Entomologist’s News 30(2): 60-61.

Brou Jr., V.A. 2008l. *Holomelina rubicundaria* (Hubner) (Lepidoptera: Arctiidae) in Louisiana. Southern Entomologist’s News 30(2): 61-63.

Brou Jr., V.A. 2008m. *Apatelodes torrefacta* (J.E. Smith, 1797) (Lepidoptera: Apatelodidae) in Louisiana. Southern Entomologist’s News 30(3): 108-109.

Brou Jr., V.A. 2008n. *Agathodes designalis* Guenee, 1854 (Lepidoptera:Pyralidae) in Louisiana. Southern Entomologist’s News 30(3): 124.

Brou Jr., V.A. 2008o. *Stiria rugifrons* Grote, 187 4 (Lepidoptera: Noctuidae) in Louisiana. Southern Entomologist’s News 30(4): 141-142.

Brou Jr., V.A. 2008p. Zale horrida Hubner, 1818 (Lepidoptera: Noctuidae) in Louisiana. Southern Entomologist’s News 30(4): 142-143.

Brou Jr., V.A. 2008q. *Diphthera festiva* (Fabricius, 1775) (Lepidoptera: Noctuidae) in Louisiana. Southern Entomologist’s News 30(4): 147-148.

Brou Jr., V.A. 2008r. *Basicladus tracyi* (Jones, 1911) (Lepidoptera: Psychidae) in Louisiana. Southern Lepidopterists' News 30(4): 149.

Brou Jr., V.A. 2008s. *Terastia meticulosalis* Guenee (Lepidoptera: Pyralidae) in Louisiana. Southern Entomologist’s News 30(4): 164.

Brou Jr., V.A. 2010a. Adult phenotypes of *Epimecis hortaria* (Fabricius, 1794) (Lepidoptera: Geometridae) from St. Tammany Parish, Louisiana. Southern Entomologist’s News 32(2): 56-57.

Brou Jr., V.A. 2010b. The genus *Dargida* Walker, 1856 (Lepidoptera: Noctuidae) in Louisiana. Southern Entomologist’s News 32(2): 71-72.

Brou Jr., V.A. 2010c. *Catocala andromedae* Guenee, 1852 (Lepidoptera: Noctuidae) in Louisiana. Southern Entomologist’s News 32(2): 80-81.

Brou Jr., V.A. 2010d. *Phrygionis privignaria* Guenée 1857 (Lepidoptera: Geometridae) in Louisiana. Southern Entomologist’s News 32: 35-39.

Brou Jr., V.A. 2011a. The *Eubaphe* Hübner, 1823 (Geometridae) of Louisiana. Southern Lepidopterists’ News 33(4): 146-147.

Brou Jr., V.A. 2011b. *Condica claufacta* (Walker, 1857) (Lepidoptera: Noctuidae) in Louisiana. Southern Lepidopterists’ News 33(4): 151.

Brou Jr., V.A. 2012a. *Pyrrhia aurantiago* (Guenee, 1852) (Lepidoptera: Noctuidae) in Louisiana. Southern Lepidopterists’ News 34(4): 186.

Brou Jr., V.A. 2016a. A new Louisiana record for an Asian pyralid species, *Nacoleia charesali*s (Walker, 1859). Southern Lepidopterists' News 38: 90-91.

Brou Jr., V.A. 2017a. *Apocheima* Hübner, [1825] of Louisiana (Lepidoptera: Geometridae) in Louisiana. Southern Lepidopterists' News 39: 37-39.

Brou Jr., V.A. and C.Z. Brou. 1997. Distribution and phenologies of Louisiana Sphingidae. Journal of the Lepidopterists' Society 51(2): 156-175.

Brou Jr., V.A. and C.D. Brou. 2019. The genus *Callosamia* Packard (Lepidoptera: Saturnidae) in Louisiana. Southern Lepidopterists’ News 41: (2) 1-6.

Brou Jr., V.A. and C.D. Brou. 2020a. *Iscadia aperta* Walker, 1857 (Lepidoptera: Nolidae) in Louisiana. Southern Lepidopterists’ News 42: 59-60.

Brou Jr., V.A. and C.D. Brou. 2020b. The genus *Doryodes* Guenée, 1857 (Lepidoptera: Erebidae) in Louisiana. Southern Lepidopterists’ News 42(4): 345-350.

Brou Jr., V.A. and C.D. Brou. 2024a. *Apantesis phalerata* (Harris, 1841) (Lepidoptera: Erebidae) in Louisiana. Southern Lepidopteraists' News 46: 132-134.

Brou Jr., V.A. and J.D. Lafontaine. 2009. A new species of *Lithophane* Hbn. (Lepidoptera, Noctuidae, Xyleninae) from southeastern United States. In: B.C. Schmidt and J.D. Lafontaine, (Eds.). Contributions to the Systematics of New World Macro-Moths. ZooKeys 9: 11-20.

Brou Jr., V.A., B. Mather, C. Watson, and J. Hyatt. 1985. Zone Reports. Zone II: Ala., La., Miss.,Tenn. Southern Lepidopterists’ News 7(1): 4.

Brou Jr., V.A., M.T. Lefort, and K.J. Cunningham. 2008. *Anartia jatrophae guantanamo* Munroe in Louisiana. Southern Entomologist’s News 30(3): 100-101.

Brou Jr., V.A.., A.R. Giese, and D.H. Miller. 2013. A new U.S. state record for a tropical fruit-piercing moth in the family Erebidae Leach. Southern Lepidopterists’ News 35: 27-28.

Brou Jr., V.A., C.D. Brou, R. Kergosien, L.G. Crabo and S.R. Nanz. 2024. *Ponometia bruchi* (Bryer, 1931) (Noctuidae, Acontiinae) in Arizona, Louisiana, Mississippi and Texas. Southern Lepidopterists' News, 46(3): 204-206.

Brower, A.E. 1974. A list of the Lepidoptera of Maine, part I Macrolepidoptera. Life Science and Agriculture Experiment Station University of Maine Technical Bulletin 66: 1-136.

Brower, A.E. 1976. New *Catocala* of North America (Noctuidae). Journal of the Lepidopterists' Society 30(1): 33-37.

Brower, A.E. 1983. A List of the Lepidoptera of Maine. Part 2. The Microlepidoptera. Section 1 Limacodidae through Cossidae. Maine Agricultural Experiment Station Technical Bulletin 109.

Brower, A.E. 1984. A list of the Lepidoptera of Maine: Part 2. The Microlepidoptera section 2, Cosmopterigidae through Hepialidae. Maine Agricultural Experiment Station Technical Bulletin 114.

Brown, A.W.A. and W.C. McGuffin, 1942. New descriptions of larvae of forest insects, III. Zanclognatha, Palthis and Autographa. (Lepidoptera, Phalaenidae). Canadian Entomologist 74: 52-56.

Brown, F.M. 1964. W. H. Edwards' life histories of North American *Coenonympha*. Journal of Research on the Lepidoptera 3(2): 121-128.

Brown, J.A. and J.A. Powell. 1991. Systematica of the *Chrysoxena* Group Genera (Lepidoptera: Tortricidae: Euliini), University of California Publications in Entomology 111: 1-87.

Brown, J.A., O. Pellmyr, J.N. Thompson, and R.G. Harrison. 1994. Phylogeny of *Greya* (Lepidoptera: Prodoxidae), based on nucleotide sequence variation in mitochondrial cytochrome oxidase I and II: congruence with morphological data. Molecular Biology and Evolution 11(1): 128-141.

Brown, J.W. 1981. Notes on the life history and Baja California distribution of *Chlorostrymon simaethis sarita* (Skinner) (Lepdioptera: Lycaenidae). The Journal of Research on the Lepidoptera 20(4): 207-213.

Brown, J.W. 1990. New species and first U.S. record of *Auratonota* (Lepidoptera: Tortricidae). Florida Entomologist 73: 153-157.

Brown, J.W. 2001. Species turnover in the leafrollers (lepidoptera: Tortricidae) of Plummers Island, Maryland: Assessing a century of inventory data. Proceedings of the Entomological Society of Washington 103: 673-682.

Brown, J.W. 2004. Preliminary assessment of Lepidoptera diversity on the Peninsula of Baja California, Mexico, with a list of documented species. Folia Entomologica Mexicana 43(1): 87-114.

Brown, J.W. 2008. Review of the Neotropical genus *Cacocharis* Walsingham (Lepidoptera: Tortricidae: Olethreutini), with a new synonymy and comments on its host plants and geographic distribution. Proceedings of the Entomological Society of Washington 110(3): 533-542.

Brown, J.W. 2009. The discovery of *Megalota* in the Neotropics, with a revision of the New World species (Lepidoptera: Tortricidae: Olethreutini). Zootaxa 2279(1): 1-50.

Brown, J.W. 2013. A new genus of pine-feeding Cochylina from the western United States and northern Mexico (Lepidoptera: Tortricidae: Euliini). Zootaxa 3640: 270-283.

Brown, J.W. 2014. New Tortricid taxa described in 2012. TORTS 15(1): 2-18.

Brown, J.W. 2016. A new generic assignment for *Tortrix baboquavariana* Kearfott, 1907 (Lepidoptera: Tortricidae) with comments on its tribal assignment. The Journal of the Lepidopterists' Society 7(2): 173-175.

Brown, J.W. 2019a. New genera, new species, and new combinations in New World Cochylina (Lepidoptera: Tortricidae: Tortricinae). Zootaxa 4671(2): 195-222.

Brown, J.W. 2019b. Two new species and two new combinations in *Saphenista Walsingham*, 1914 from western North America (Lepidoptera: Tortricidae). Insecta Mundi 0738: 1-8.

Brown, J.W. 2019c. New Combinations in Sparganothini (Lepidoptera: Tortricidae: Tortricinae). Proceedings of the Entomological Society of Washington 121(3) 366-371.

Brown, J.W. and K. Bash. 2000. The Lepidoptera of Marine Corps Air Station Miramar: calculating faunal similarity among sampling sites and estimating total species richness. Journal of Research on the Lepidoptera 36: 45-78.

Brown, J.W. and R.L. Brown, 2004. A new species of *Cryptaspasma* (Lepidoptera: Tortricidae: Olethreutinae) from Central America, the Caribbean, and southeastern United States, with a catalogue of the world fauna of Microcorsini. Proceedings of the Entomological Society of Washington 106: 288-297.

Brown, J.W. and R.L. Brown. 2018. Revised status of *Epinotia cupressi* Heinrich and descriptions of two new species of *Epinotia* Hübner, [1825], from the western USA (Lepidoptera: Tortricidae: Olethreutinae). Proceedings of the Entomological Society of Washington 120(4): 675-686.

Brown, J.W. and A. Cramer. 1999. Five new species of *Argyrotaenia* (Tortricidae: Archipini) from Mexico and the southwestern United States. Journal of the Lepidnpterists' Society 53(3): 114-125.

Brown, J.W. and J.P. Donahue. 1989. The Sphingidae (Lepidoptera) of Baja California, Mexico. Journal of the Lepidopterists' Society 43(3): 184-209.

Brown, J.W. and J. Lewis, 2004. Catalogue of the Type Specimens of Tortricidae (Lepidoptera) in the Collection of the National Museum of Natural History, Smithsonian Institution, Washington, D.C. Systematic Entomology Laboratory, Plant Sciences Institute, Agricultural Research Service, U.S. Department of Agriculture, National Museum of Natural History. http://www.sel.barc.usda.gov/lep/tort_types_intro.html

Brown, J.W. and S. Passoa. 1998. Larval foodplants of Euliini (Lepidoptera: Tortricidae): from *Abies* to *Vitis*. Pan-Pacific Entomolgist 74: 1-11.

Brown, J.W. and J.A. Powell. 1991. Systematics of the *Chrysoxena* group of genera (Lepidoptera: Tortricidae: Euliini). University of California Publications in Entomology. 111. 87 pp.

Brown, J.W. and J.A. Powell. 2000. Systematics of *Anopina* Obraztsov (Lepidoptera: Tortricidae: Euliini). University of California Publications in Entomology 120. 128 pp + figs.

Brown, J.W. and J. Razowski. 2003. Description of *Ptychocroca*, a new genus from Chile and Argentina, with comments on the *Bonagota* Razowski group of genera (Lepidoptera: Tortricidae: Euliini). Zootaxa 303(1): 1-31.

Brown, J.W. and J.B. Sullivan. 2018. A new species of *Cenopis* Zeller, 1876, from the southeastern United States (Lepidoptera: Tortricidae: Sparganothini). Proceedings of the Entomological Society of Washington 120(3): 493-499.

Brown, J.W. and A.E. Timm. 2017. Descriptions of two new species of *Ecdytolopha* Zeller, 1875 (Lepidoptera: Tortricidae: Olethreutinae), one from the Southwestern US and One from Venezuela, with comments on *Ecdytolopha* Barcodes. Proceedings of the Entomological Society of Washington 119(1): 9-17.

Brown, J.W. and C. Zachariades. 2007. A new species of *Dichrorampha* (Lepidoptera: Tortricidae: Grapholitini) from Jamaica: A potential biocontrol agent against Chromolaena odorata (Asteraceae). Proceedings-Entomological Society of Washington 109(4): 938-947.

Brown, J.W., D. Adamski, R.H. Hodges and S.M. Bahr II. 2004a. Catalog of the type specimens of Gelechioidea (Lepidoptera) in the collection of the National Museum of Natural History, Smithsonian Institution, Washington, D.C. Zootaxa 510: 1-160.

Brown, J.W., M.E, Epstein and E.R. Day. 2004b. First report of *Pryeria sinica* (Lepidoptera: Zygaenidae) in North America. Proceedings of the Entomological Society of Washington 106: 239-242.

Brown, J.W., G. Robinson, and J. A. Powell. 2008. Food plant database of the leafrollers of the world (Lepidoptera: Tortricidae) (Version 1.0). http://www.tortricid.net/foodplants.asp.

Brown, J.W., M.E. Epstein, T.M. Gilligan, S.C. Passoa, and J.A. Powell. 2010. Biology, identification, and history of the light brown apple moth, *Epiphyas postvittana* (Walker) (Lepidoptera: Tortricidae: Archipini) in California: An example of the importance of local faunal surveys to document the establishment of exotic insects. American Entomologist 56(1): 34-43.

Brown, J.W., R. Segura, Q. Santiago-Jiménez, J. Rota, and T.A. Heard. 2011. Tortricid moths reared from the invasive weed Mexican palo verde, *Parkinsonia aculeata*, with comments on their host specificity, biology, geographic, distribution, and systematics. Journal of Insect Science 11(7): 1-17.

Brown, J.W., L. Aarvik, M. Heikkilä, R. Brown, and M. Mutanen. 2020a. A molecular phylogeny of Cochylina, with confirmation of its relationship to *Euliina* (Lepidoptera: Tortricidae). Systematic Entomology 45(1): 160-174.

Brown, J.W., S. Gripenberg, Y. Basset, O. Calderón, I. Simon, C. Fernandez, M. Cedeno, and M. Rivera. 2020b. Host records for Tortricidae (Lepidoptera) reared from seeds and fruits in Panama. Proceedings of the Entomological Society of Washington 122(1): 12-24.

Brown, L.N. 1972. The silkmoths of Florida. Florida Naturalist 45: 40-4.

Brown, L.R. and C.O. Eads. 1967. Insects affecting ornamental conifers in southern California. University of California. California Agriculture Station Bulletin 834.

Brown, M.W. 1990. Abundance and identification of the leafmining guild on apple in the Mid-Atlantic states. The Great Lakes Entomologist 23(4): 179-188.

Brown, R.L. 1979. Nomenclatural changes in Eucosmini (Tortricidae). Journal of the Lepidopterists’ Society 33(1): 21-28.

Brown, R.L. 1980a. A revision of the genus *Epinotia* (Hübner) (Tortricidae: Eucosmini), Part 1: The North American species of the stroemiana lineage. Ph.D. Thesis. Cornell University, Ithaca, NY. 460 pp.

Brown, R.L. 1980b. A new species of *Epinotia* Hübner (Lepidoptera: Tortricidae). Proceedings of the Entomological Society of Washington. 82: 504-509.

Brown, R.L. 1982. Notes on *Gretchena*: A new species and the synonymy of *Gwendolina* (Lepidoptera: Tortricidae). Proceedings of the Entomological Society of America 84(3): 594-602.

Brown, R.L. 1983. Taxonomic and morphological investigations of Olethreutinae: *Rhopobota*, *Griselda*, *Melissopus*, and *Cydia* (Lepidoptera: Tortricidae). Entomography 2: 97-120.

Brown, R.L. 1984. Review of *Corticivora* (Lepidoptera: Tortricidae) with analysis of its tribal relationships and descriptions of new species. Proceedings of the Entomological Society of Washington 86(2): 278-286.

Brown, R.L. 1986. Resurrection of *Catastega* Clemens and revision of the *Epinotia vertumnana* (Zeller) species-group (Tortricidae: Olethreutinae). Journal of the Lepidopterists' Society 40(4): 327-346.

Brown, R.L. 1992. Six new species of *Catastega* (Lepidoptera: Tortricidae: Eucosmini) from Mexico and Southwestern United States. Journal of the New York Entomological Society 100 (2): 209-227

Brown, R.L. 2003. A Preliminary Survey of Moths in Cedar Glades and Adjacent Forests of Central Tennessee. Report to the Tennessee Division of Natural Heritage and The Nature Conservancy of Tennessee. Mississippi Entomological Museum, Mississippi State, Mississippi.

Brown, R.L. and R.T. Allen, 1974. Larval food plants and parasites of some Lepidoptera in southeast Arkansas. Journal of the Lepidopterists’ Society 28(2): 168-170.

Brown, R.L. and C. Jaeger. 2014. A new species of *Eucosma* Hübner and two new species of *Cydia* Hübner (Lepidoptera: Tortricidae) from the United States. Zootaxa 3860(3): 226-234.

Brown, R.L. and W.E. Miller. 1983. Valid names of the spruce seed moth and a related *Cydia* species (Lepidoptera: Tortricidae). Annals of the Entomological Society of America. 76: 110-111.

Brown, R.L. and J.A. Powell. 1991. Description of a new species of *Epiblema* (Lepidoptera: Tortricidae: Olethreutinae) from coastal redwood forests in California with an analysis of the forewing pattern. Pan-Pacific Entomologist 67(2): 107-114

Brown, R.L., J.F.G. Clarke and D.H. Habeck. 1983. New host records for Olethreutinae (Tortricidae). Journal of the Lepidopterists’ Society 37: 224-227.

Brown, R.M. 1975. Revision of the North American *Comadia* (Cossidae). Journal of Research on the Lepidoptera 14(4): 189-212.

Brown, R.M. 1987. A new genus and species from the southwestern United States (Noctuidae: Acontiinae). Journal of Research on the Lepidoptera 25(2): 136-145.

Brown, R.M. 1998. A new species of *Hulstina* from California (Geometridae: Ennominae). Journal of the Lepidopterists' Society 52(3): 335-337.

Brown, R.M. and P.A. Opler. 1965. Biological observations on *Callophrys viridis* (Lycaenidae). Journal of the Lepidopterists' Society 21(2): 113-114.

Browne, F.G. 1968. Pests and diseases of forest plantation trees: an annotated list of the principal species occurring in the British Commonwealth. Clarendon Press, Oxford University Press, Oxford, United Kingdom.

Bruner, S.C., L.C. Scaramuzza, and A.R. Otero. 1975. Catalogo de los insectos que atacan a las plantas económicas de Cuba: segunda edición revisada y aumentada. Academeia de Ciencisa de Cuba, Instituto Zoologica, La Habana. 399 pp.

Brunet, B.M.T., G.S. Blackburn, K. Muirhead, L.M. Lumley, B. Boyle, R.C. Levesque, M. Cusson, and F.A.H. Sperling. 2017. Two's company, three's a crowd: New insights on spruce budworm species boundaries using genotyping‐by‐sequencing in an integrative species assessment (Lepidoptera: Tortricidae). Systematic Entomology 42(2): 317-328.

Bruzzese, D.J., D.L. Wagner, T. Harrison, T. Jogesh, R.P. Overson, N.J. Wickett, R.A. Raguso, and K.A. Skogen. 2019. Phylogeny, host use, and diversification in the moth family Momphidae (Lepidoptera: Gelechioidea). PloS one 14(6): e0207833.

Bryant, S., C. Thomas, and J. Bale. 1997. Nettle‐feeding nymphalid butterflies: temperature, development and distribution. Ecological Entomology 22(4): 390-398.

Bucheli, S.R. and J. Wenzel. 2005. Gelechioidea (Insecta: Lepidoptera) systematics: a reexamination using combined morphology and mitochondrial DNA data. Molecular phylogenetics and evolution, 35(2): 380-394.

Bucheli, S., J.F. Landry, and J. Wenzel. 2002. Larval case architecture and implications of host‐plant associations for North American *Coleophora* (Lepidoptera; Coleophoridae) 1. Cladistics 18(1): 71-93.

Bucheli, S.R., D.J. Horn, and J.W. Wenzel. 2006. Sampling to assess a re-established Appalachian forest in Ohio based on gelechioid moths (Lepidoptera: Gelechioidea). Biodiversity & Conservation 15(1): 503-516.

Buck, S.E. III. 2004. Insect Fauna Associated with Eastern Hemlock, *Tsuga canadensis* (L.), in the Great Smoky Mountains National Park. " Master's Thesis, University of Tennessee, Knoxville, Tennessee.

Buckett, J.S. 1964a. Revision of the North American genus *Behrensia* with a description of new species. Journal of Research on the Lepidoptera 3(3): 129-144.

Buckett, J.S. 1965a. Identity of *Heliosea celeris melicleptroides* with notes on its habits. Journal of Research on the Lepidopter 4: 79-80.

Buckett, J.S. 1966a.The little known moth *Euxoa sculptilis* (Harvey) in Arizona, with descriptions, illustrations, and notes on *Euxoa violaris* (Grote and Robinson) (Noctuidae- Agrotiinae). Journal of Research on the Lepidoptera 5: 255-261.

Buckett, J.S. 1967a. Description of a new species of *Xylomiges* from California, with notes and illustrations (Lepidoptera: Noctuidae: Hadeninae). Journal of Research on the Lepidoptera 6: 23-30.

Buckett, J.S. 1967b. A new species of *Feralia* from Santa Catalina Island of California, with notes on the immature stages of *Feralia februlis* Grote (Noctuidae: Cuculliinae). Journal of Research on the Lepidoptera 6(4): 43-51.

Buckett, J.S. 1967c. A new species of armyworm belonging to the genus *Faronta* Smith from the western United States (Noctuidae). Journal of Research on the Lepidoptera 6:2 68-274.

Buckett, J.S. 1968a. Revision of the Nearctic moth genus *Abagrotis* Smith with descriptions of new species. Part 1. California Department Agriculture Bureau of Entomology Occasional Papers 12: 1-21.

Buckett, J.S. 1968b. Revision of the Nearctic moth genus *Abagrotis* Smith with descriptions of new species. Part 2. California Department Agriculture Bureau of Entomology Occasional Papers 14: 1-16.

Buckett, J.S. 1968c. Revision of the Nearctic moth genus *Abagrotis* Smith with descriptions of new species. Part 3. California Department Agriculture Bureau of Entomology Occasional Papers 15: 1-29.

Buckett, J.S. 1968d. Revision of the Nearctic moth genus *Abagrotis* Smith with descriptions of new species. Part 4. California Department Agriculture Bureau of Entomology Occasional Papers 16: 1-27.

Buckett, J.S. 1968e. Species in the genera *Polia* Ochsenheimer and *Euxoa* Hubner from the western United States (Lepidoptera, Noctuidae). Journal of Research on the Lepidoptera 7(2): 87-94.

Buckett, J.S. 1968f. Identity of the moth "*Stretchia*" *behrensiana* (Grote) with new synonymy (Noctuidae). Journal of Research on the Lepidoptera 7: 57-63.

Buckett, J.S. 1969. Revision of the Nearctic moth genus *Abagrotis* Smith with descriptions of new species. Part 5. California Department Agriculture Bureau of Entomology Occasional Papers 17: 1-27.

Buckett, J.S. 1971a. Revision of the Nearctic genus *Philtraea* Grote with notes on the biology and the descriptions of new species (Geometridae). Journal of Research on the Lepidoptera 9(1): 29-64.

Buckett, J.S. 1972a. Identity of the moth *Oncocnemis semicollaris* J. B. Smith. Journal of Research on the Lepidoptera 10(3): 248-254.

Buckett, J.S. 1972b [1973]. A new species of Nephelodes Guenee from the Great Basin area of northeastern California (Noctuidae: Hadeninae). Journal of Research on the Lepidoptera 11: 260-268.

Buckett, J.S. and W.R. Bauer 1964a. A new species of *Euxoa* Hbn. (Lepidoptera: Noctuidae) from the Sierra Nevada of California. The Canadian Entomologist 96(07): 967-970.

Buckett, J.S. and W.R. Bauer 1964b. Review of the *depicta* group of the genus *Annaphila*. Journal for Research on Lepidoptera 2(2): 95-101.

Buckett, J.S. and W.R. Bauer 1964c. *Petaluma*, a new genus, with description of a new species. Journal for Research on Lepidoptera 3(3): 193-196.

Buckett, J.S. and W.R. Bauer 1966a. A new species of *Oncocnemis* from the western United States (Noctuidae: Cuculiinae). Journal for Research on Lepidoptera 5(4): 197-208.

Buckett, J.S. and W.R. Bauer 1966b. Key to the generaof Psaphidini, with descriptions of a new genus and species from the western North America (Noctuidae: Cuculiinae). Journal of the Lepidopterists’ Society 20(2): 83-91.

Buckett, J.S. and W.R. Bauer 1966c. A new species of *Polia* Ochsenheimer from California and notes on *Polia discalis* (Grote) (Noctuidae: Hadeninae). Journal for Research on Lepidoptera 5(4): 221-228.

Buckett, J.S. and W.R. Bauer 1967. Description of a new species of *Anepia* Hampson from the Sierra Nevada of California (Noctuidae). Journal of the Lepidopterists’ Society 21(4): 235-240.

Buckholtz, O. 1919. The larvae of *Datana chiridnensis*. The Lepidopterists 3(1): 102.

Buckingham, G.R. 2002. Alligatorweed pp. 5-15 In: R. Van Driesche, S. Lyon, B. Blossey, M. Hoddle, and R. Reardon (coord.). Biological control of invasive plants in the eastern United States. USDA Forest Service Publication FHTET-2002-04.

Buckingham, G.R. and C.A. Bennett. 1996. Laboratory biology of an immigrant Asian moth, *Parapoynx diminutalis* (Lepidoptera: Pyralidae), on *Hydrilla verticillata* (Hydrocharitaceae). Florida Entomologist 79(3): 353-363.Busck. 1917. Descriptions of new North American Microlepidoptera. Proceedings of the Entomological Society of Washington 18: 147-156.

BugGuide.Net. 2020. BugGuide.Net 2003-2021. Iowa State University, Ames, Iowa. https://bugguide.net.

Buhr, H. 1935a. Mecklenburgische Minen. III. Lepidopteren-Minen. Stettiner Entomologische Zeitung 96: 131-159.

Buhr, H. 1935b. Mecklenburgische Minen. III. Lepidopteren-Minen. Stettiner Entomologische Zeitung 96: 262-292.

Bunker, R. 1877. Notes on the food plant of *Hemileuca maia*. The Canadian Entomolgist 3: 119.

Burke, J.T. and R.S. Peigler. 2009. Phylogenetic analysis of *Anisota* (Insecta: Lepidoptera: Saturniidae) based on scolus size and structure of mature larvae. Southeastern Naturalist 8(4): 739-745.

Burns, J.M. 1974. The polytypic genus *Celotes* (Lepidoptera: Hesperiidae: Pyrginae) from the southwestern United States and northern Mexico. Psyche 81(1): 51-69.

Burns, J.M. 1983. Queen of the Carolinas (Nymphalidae: Dananinae: *Danaus gilippus*). Proceedings of the Entomological Society of Washington 85(2): 388-396.

Burns, J.M. 1994a. Split skippers: Mexican genus *Poanopsis* goes in the origenes group - and *Yvretta* forms the *rhesus* group - of *Polites* (Hesperiidae). Journal of the Lepidopterists' Society 48(1): 24-45.

Burns, J.M. 1994b. Genitalia at the generic level: *Atrytone* restricted, *Anatrytone* resurrected, new genus *Quasimellana*-and yes! we have no *Mellanas* (Hesperiidae). Journal of the Lepidopterists’ Society 48(4): 273-337.

Burns, J.M. 1996. Genitalia and the proper genus: *Codatractus* gets *Mysie* and *Uvydlxa*-in a compact *Cyda* Group-as well as a *Hysterectomy*, while C*ephise* gets part of Polythrix (Hesperiidae: Pyrginae). Journal of the Lepidopterists' Society 50(3): 173-216.

Burns, J.M. 2000. *Pyrgus communis* and *Pyrgus albescens* (Hesperiidae: Pyrginae) are separate transcontinental species with variable but diagnostic valves. Journal Lepidopterists Society 54(2): 52-71.

Burns, J.M. 2015. Speciation in an insular sand dune habitat: *Atrytonopsis* (Hesperiidae: Hesperiinae) - mainly from the Southwestern United States and Mexico - off the North Carolina coast. Journal of the Lepidopterists' Society 69: 275-292.

Burns, J.M., 2020. Taxonomic Status of a Florida Differentiate in the *Erynnis brizo* Species Group: Classical Evidence (Lepidoptera: Hesperiidae: Pyrginae). Proceedings of the Entomological Society of Washington 122(1): 25-41.

Burns, J.M. and D.H. Janzen. 2001. Biodiversity of Pyrrhopyginae skipper butterflies (Hesperiidae) in the Area de Conservación Guanacaste, Costa Rica. Journal of the Lepidopterists Society 55(1): 15-43.

Burns, J.M., D.H. Janzen, M. Hajibabaei, W. Hallwachs, and P.D.N. Hebert. 2008. DNA barcodes and cryptic species of skipper butterflies in the genus Perichares in Area de Conservacion Guanacaste, Costa Rica. Proceedings of the National Academy of Sciences 105(17): 6350-6355.

Burns, J.M., D.H. Janzen, W. Hallwachs, M. Hajibabaei, and P.D.N. Hebert. 2010. Genitalia, DNA barcodes, larval facies, and foodplants place the mimetic species *Neoxeniades molion* in *Rhinthon* Hesperiidae: Hesperiinae. Journal of the Lepidopterists Society 64(2): 69-78.

Burton, R. and W. A. Drew. 1969. The sphinx moths (Lepidoptera: Sphingidae) of Oklahoma. Proceedings of the Oklahoma Academy of Sciences 48: 16-22.

Bury, J. and V. Savchuk. 2015. New data on the biology of ten lycaenid butterflies (Lepidoptera: Lycaenidae) of the genera Tomares Rambur, 1840, Pseudophilotes Beuret, 1958, *Polyommatus* Latreille, 1804, and *Plebejus* Kluk, 1780 from the Crimea and their attending ants (Hymenoptera: Formicidae). Actaentomologica Silesiana 23(online 018): 1-16.

Busck, A. 1900a. New species of moths of the Superfamily Tineina from Florida. Proceedings of the United States National Museum 23(1208): 225-254.

Busck, A. 1900b. New American Tineina. Journal of the New York Entomological Society 8(4): 234-248. [actual pubishe date 1901]

Busck, A. 1903a. A revision of the American moths of the family Gelechiidae, with descriptions of new species. Proceedings of the United States National Museum 25(1304): 767-930.

Busck, A. 1903b. Notes on Brackenridge Clemens' types of Tineina. Proceedings of the Entomological Society of Washington 5(3): 181-220.

Busck, A. 1903c. Notes on the Cerostoma group of Yponomeutidæ, with descriptions of new North American species. Journal of the New York Entomological Society 11(1): 45-59

Busck, A. 1903d. Supplement to the revision American Gelechiidae. Proceedings of the United States National Museum 25(1304): 931-938.

Busck, A. 1904a. Tineid moths from British Columbia, with descriptions of new species. Proceedings of the United States National Museum 27(1375): 745-778.

Busck, A. 1904b. A new Tineid genus from Arizonia. Proceedings of the Entomological Society of Washington 6(2): 123-124.

Busck, A. 1906a. Descriptions of American moths of the genus *Cerostoma*. Entomological News and Proceedings of the Entomological Section Academy of Natural Sciences, Philadelphia 17(3): 96-99.

Busck, A. 1906b. Tineid moths from southern Texas, with descriptions of new species. Proceedings of the United States National Museum 30: 721-736.

Busck, A. 1906c. Notes on some Tortricid genera with description of New American species. Proceedings of the Biological Society of Washington 19: 173-182.

Busck, A. 1906d. A Review of the American moths of the genus *Cosmopteryx* Hubner. Proceedings of the United States National Museum 30(1463): 707-718.

Busck, A. 1906e. New American Tineina. Canadian Entomologist 38: 121-125.

Busck, A. 1907a. Revision of the American moths of the genus *Argyresthia.* Proceedings of the United States National Museum 32: 5-24 & plates IV-V.

Busck, A. 1907b. New genera and species of American Microlepidoptera. Journal of the New York Entomological Society 15: 134-140.

Busck, A. 1907c. A review of the Tortricid Subfamily Phaloniinae with descriptions of New American species. Journal of the New York Entomological Society 15(1): 19-36.

Busck, A. 1907d. New American Tineina. Proceedings of the Entomological Society of Washington 8(3-4): 86-99.

Busck, A. 1908a. A generic revision of American moths of the family Œcophoridœ, with descriptions of new species. Proceedings of the United States National Museum 35: 187-207.

Busck, A. 1908b. Descriptions of some new microlepidopetra from Pennsylvania. Canadian Entomologist 40(6): 193-196.

Busck, A. 1908c. Descriptions of two new Gelechidae from California. Entomology News 19: 316-317.

Busck, A. 1908d. Descriptions of North American Tineina. Proceedings of the Entomological Society of Washington 9(1-4): 85-95.

Busck, A. 1909a. New Microlepidoptera from New Mexico and California and a synoptic table of the North American species of Heliodines Stainton. Proceedings of the Entomological Society of Washington 11: 175-188.

Busck, A. 1909b. Notes on Microlepidoptera with descriptions of new North American species. Proceedings of the Entomological Society of Washington 11(2): 87-103.

Busck, A. 1910. New Central-American Microlepidoptera introduced into the Hawaiian Islands. Proceedings of the Entomological Society of Washington 12 (3): 132-135.

Busck, A. 1911. Descriptions of tineoid moths (Microlepidoptera) from South America. Proceedings of the United States National Museum 40(1815): 205-230 plus 2 plates.

Busck, A. 1912a. New Microlepidoptera from Mexico. Proceedings of the Entomological Society of Washington 14: 83-87.

Busck, A. 1912b. A new injurious *Plutella*. Proceedings of the Entomological Society of Washington 14: 219.

Busck, A. 1913. New Californian Microlepidoptera. Journal of Entomology and Zoology 5(2); 96-102.

Busck, A. 1914. Seven new species of *Ethmia* from Tropical America. Insecutor Inscitiae Menstruus 2(4): 53-57.

Busck, A. 1915. Descriptions of new North American Microlepidoptera. Proceedings of the Entomological Society of Washington 17(2): 79-94.

Busck, A. 1916. Descriptions of new North American Microlepidoptera. Proceedings of the Entomological Society of Washington 18: 147-154.

Busck, A. 1919. Two Microlepidoptera injurious to strawberry. Proceedings of the Entomological Society of Washington 21(3): 52-52.

Busck, A. 1920. A new *Gracilaria* injurious to avacado (Lepid.) Canadian Entomologist 52: 239.

Busck, A. 1922 [dated 1921]. Microlepidoptera from British Columbia. Canadian Entomologist 8(12): 276-280.

Busck, A. 1932. Description of a new Cosmopterygid leaf miner (Lepidoptera) on Helianthus. Proceedings of the Entomological Society of Washington 4(2): 17-23.

Busck, A. 1934 [dated 1933]. Microlepidoptera of Cuba. Entomologica Americana 13(4): 151-217.

Busck, A. 1934a. *Tortilia viatrix*, new species. An African moth on senna imported into the United States. Proceedings of the Entomological Society of Washington 36(3): 68-70.

Busck, A. 1934b. Two new microlepidoptera from California. Bulletin of the Southern California Academy of Sciences 33(2): 74-78.

Butler, A.G. 1878. Descriptions of new species of Heterocera from Japan. Part-II. Noctuites. The Annals and Magazine of Natural History 5^th^ series 1: 77-85.

Butler, L. 1986. Biology and description of immature stages of *Phigalia strigateria* (Minot) (Geometridae). Journal of the Lepidopterists Society 40(4): 289-297.

Butler, L. 1987. A new species of Nearctic *Bomolocha* (Noctuidae) from the Appalachian area. Journal of the Lepidopterists' Society 41(2): 104-107.

Butler, L. 1989. Observations on *Meganola spodia* Franclemont (Lepidoptera: Noctuidae) with description of the mature larva. Proceedings of the Entomological Society of Washington 91(4): 615-619.

Butler, L. 1992. The community of macrolepidopterous larvae at Cooper's Rock State Forest, West Virginia: a baseline study. Canadian Entomologist 124: 1149-1156.

Butler, L. and J. Strazanac. 2000. Occurrence of Lepidoptera on selected host trees in two central Appalachian national forests. Annals of the Entomological Society of America 93(3): 500-511.

Butler, L., V. Kondo, and J. Strazanac. 2001. Light trap catches of Lepidoptera in two central Appalachian forests. Proceedings of the Entomological Society of Washington 103(4): 879-902.

Butler, M. and B. Hammond. 2001. Grass-feeding moths collected in commercial Kentucky bluegrass fields of central and eastern Oregon, 2000. Pp. 45-48 In: 2000 Seed Production Research at Oregon State University USDA-ARS Cooperating, Ext/CrS 115, Corvallis, Oregon.

Butler, M. and B. Hammond. 2002. Grass-feeding moths collected in Kentucky bluegrass seed fields treated with post-harvest burning or bale-only in the Grande Ronde Valley, 2001. Pp. 86-88 In: W.C. Young III. 2001 Seed Production Research at Oregon State University. Oregon State University, Department of Crop and Soil Science, Corvallis, Oregon.

Byers, J.R. 1988. Wooly-bear caterpillars of *Grammia blakei* (Grote) (Lepidoptera: Arctiidae) - an occasional pest of native range pastures and adjacent crops in southern Alberta and southwestern Saskatchewan. Canadian Entomologist 120: 927-935.

Byers, B.A. 1989. Biology and immature stages of *Schinia masoni* (Noctuidae). Journal of the Lepidopterists' Society 43(3): 210-216.

BYU:BYUC. 2015. Brigham Young University Arthropod Museum, Brigham Young University, Provo, Utah. In: SCAN. P.L. Heinrich, E. Gilbert, N.S. Cobb, and N. Franz. Symbiota collections of arthropods network (SCAN): A data portal built to visualize, manipulate, and export species occurrences. [Dataset]. http://scan-bugs.org/portal/collections/index.php [Accessed 08 March 2019]

Byun, B., Y. Bae, and K. Park. 1998. Illustrated catalogue of Tortricidae of Korea (Lepidoptera). In: K.T. Park, (ed.). Insects of Korea, 2. Korea Research Institute of Bioscience and Biotechnology and Center for Insect Systematics. 317 pp.

CABI. 2010. *Duponchelia fovealis*. Crop Protection Compendium, CD-ROM. CAB International, Wallingford, UK.

Cabrera-Asencio, I., and C.E. de Jensen. 2023. *Atheloca subrufella* (Hulst) (Lepidoptera: Pyralidae: Phycitinae): The Coconut Moth in Puerto Rico. The Journal of Agriculture of the University of Puerto Rico 107(2): 183-186.

Cain, R., J. Cota, and C. Ward. 1990. Conifer Pests in New Mexico. United States Department of Agriculture, Forest Service, Southwest Region. 48 pp.

Calhoun, J.H. 1995. The biogeography and ecology of *Euphyes dukesi* (Hesperidae) in Florida. Journal of the Lepidopterists’ Society 49(1): 6-23.

Calhoun, J.V. 1997. Updated list of the butterflies and skippers of Florida (Lepidoptera: Papilionoidea and Hesperioidea). Holarctic Lepidoptera 4(2): 39-50.

Calhoun, J.V. 2018. John Abbot, Jacob Hübner and *Oreas helicta* (Nymphalidae: Satyridae) 60(4): 159-163.

Calhoun, J.V. and S.F. Kolterman. 2018. *Papilio* (*Heraclides*) *caiguanabus* Poey (Papilionidae): A new US record from the Florida Keys, with notes on its name and food plant. News of The Lepidopterists’ Society 62(2): 84-87.

Calhoun, J.V., J.R. Slotten, and M.H. Salvato. 2002. The rise and fall of tropical blues in Florida: *Cyclargus ammon* and *Cyclargus thomasi* bethunebakeri. Holarctic Lepidoptera 7(1): 13-20.

Calvo, J. R. 1966. The lesser cornstalk borer, *Elasmopalpus lignosellus* (Zeller), and its control. Ph.D. dissertation, University of Florida, Gainesville, Florida.

Capek, M. 1971. The possibility of biological control of imported weeds of the genus Solidago L. in Europe. Acta Institui Forestalis Zvolenensis 1971: 429-441.

Capinera, J.L. 2010. Sugarcane Borer, *Diatraea saccharalis* (Fabricius) (Insecta: Lepidoptera: Pyralidae). University of Florida Extension EENY-217.

Capps, H.W. 1943. Some American Geometrid moths of the subfamily Ennominae heretofore associated with or closely related to *Ellopia* Treitschke. Proceedings United States National Museum 93: 120-128.

Capps, H.W. 1965. A review of the genus *Haimbachia* Dyar with descriptions of new species (Lepidoptera: Crambidae). Proceedings of the United States Nation Museum 117(3520): 629-653.

Capps, H.W. 1966. Review of New World moths of genus *Euchromius* Guenie with descriptions of two new Species (Lepidoptera: Crambidae). Proceedings of the National Museum 119(3551): 1-14.

Capps, H.W. 1967. Review of some species of *Loxostege* Hubner and descriptions of new species (Lepidoptera, Pyraustidae: Pyraustinae). Proceedings of the National Museum 3561(120): 1-75.

Capinera, J.L. 2001. Handbook of Vegetable Pests. Academic Press, San Diego, California.

Carbonell, E.E.T. 1977. Morfologia del “barrenador menor de la cana de azucar” *Elasmopalpus lignosellus* (Zeller) (Lepidoptera: Phycitidae). Saccharum 5: 18-50.

Carlson, C.E., W.E. Bousfield, and M.D. McGregor. 1974. The relationship of an insect to fluorides emitted from a nearby aluminum plant in Montana. Insect Disease Report, USDA Forest Service, Northern Region Report Number 74-14: 1-21.

Carlton, C.E. and T.J. Kring. 1994. *Melitara prodenialis* Walker on prickly pear in Arkansas. Southwestern Entomologist 19(1): 23-31.

Carpenter, J., S. Bloem, and H. Hofmeyr. 2007. Area-wide control tactics for the false codling moth *Thaumatotibia leucotreta* in South Africa: a potential invasive species. Pp. 351-359 in: M.J.B. Vreysen, A.S. Robinson and J. Hendrichs (eds.). Area-Wide Control of Insect Pests. Springer, Dordrecht.

Carpenter, R.W. 2015. The Funereal Duskywing, *Erynnis funeralis* (Hesperiidae): Seasonal range expansion into Eastern North America. Journal of the Lepidopterists' Society 69(2): 114-124.

Carroll, M.R. and W.H. Kearby. 1978. Microlepidopterous oak leaftiers (Lepidoptera: Gelechioidea) in central Missouri. Journal of the Kansas Entomological Society 51(3): 457-471.

Carroll, M.R., M.T. Wooster, W.H. Kearby, and D.C. Allen. 1979. Biological Observations on three oak leaf tiers: *Psilocorsis quercicella,* *P. reflexella*, and *P. cryptolechiella* in Massachusetts and Missouri. Annals of the Entomological Society of America 72(3): 441-447

Carter, D.J. 1984. Pest Lepidoptera of Europa with special reference to the British Isles. Series Entomologica 31: 431.

Carter, D. 1992. Smithsonian’s Handbooks Butterflies and Moths. Dorling Kindersley, Inc. New York, NY.

Carvajal-Acosta, A.N., S.R. Abella, and D.B. Thompson. 2015. Initial vegetation response to fuel mastication treatments in rare butterfly habitat of the Spring Mountains, Nevada. Journal of the Arizona-Nevada Academy of Science 46(1); 6-18.

Cary, M.M. 1951. Distribution of Sphingidae (Lepidoptera: Heterocera) in the Antillean-Caribbean region. Transactions of the American Entomological Society 77: 63-129.

Cary, S.J. 1994. Gray Ranch: Fire and butterflies in Southwestern New Mexico. Holarctic Lepidoptera 1: 65-68.

Cary, S.J. and R. Holland. 1992. New Mexico butterflies: checklist, distribution and conservation. Journal of Research on the Lepidoptera. 31(1-2): 57-82.

Casagrande, R.A., G. Balme and B. Blossey, 2003. *Rhizedra lutosa*, a Natural Enemy of *Phragmites australis* in North America. Estuaries 26(2): 602-606.

Cashatt, E.D. 1972. Notes on the *balanotes* (Meyrick) group of *Oidaematophorus* Wallengren with description of a new species (Pterophoridae). Journal of the Lepidopterists' Society 26(1): 1-13.

Cashatt, E.D. 1984. Revision of the genus *Parachma* Walker (Pyralidae: Chrysauginae) of North America north of Mexico with description of a new genus. Journal of the Lepidopterists' Society 38(4): 268-280.

Cashatt, E.D. and G.L. Godfrey. 1990. Database of Illinois Lepidoptera Phase II. Illinois Natural History Survey Center for Biodiversity Technical Report. Project NGW895 Report.

Cashatt, E.D., G.L. Godfrey, and J.R. Wiker. 1990. Occurrences of *Eumorpha fasciata,* *Hyles gallii*, *Sphinx franckii* and *S. vashti* (Lepidoptera: Sphingidae) in Illinois. The Great Lakes Entomologist 23(4): 223-226.

Cassani, J.R., D.H. Habeck, and D.L. Matthews. 1990. Life history and immature stages of a plume moth *Sphenarches anisodactylus* (Lepidoptera: Pterophoridae) in Florida. The Florida Entomologist 73(2): 257-266.

Casanova, R.I. 1977. Notes on the life cycle of the bean leaf skeletonizer, *Syngrapha egena* (Guenee) (Lepidoptera: Noctuidae) in Puerto Rico. Journal of Agriculture of University Of Puerto Rico 61: 253-255.

Cassie, B., J, Glassberg, A, Swengel, and G. Tudor. 2001. North American Butterfly Association (NABA) Checklist & English names of North American Butterflies. North American Butterfly Association, Morristown, New Jersey.

Cassino, S.E. 1920. A new *Stamnodes* and *Marmopterx*. The Lepidopterists 3(3): 115-117.

Cassino, S.E. 1927a. Some new Geometridae. The Lepidopterists 4(8-9): 57-71.

Cassino, S.E. 1927b. Some new Geometridae. The Lepidopterists 4(10): 73-80.

Cassino, S.E. 1928a. Some new Lasiocampidae. The Lepidopterists 5(12): 90-95.

Cassino, S.E. 1928b. New Geometridae. The Lepidopterists 5(1): 1-8.

Cassino, S.E. 1931a. New Geometridae. The Lepidopterists 5(2): 1-8[9-16].

Cassino, S.E. 1931b. Some new Geometrids. The Lepidopterists 5(3): 17-24.

Cassino, S.E. and L.W. Swett. 1922a. Some new Geometrids. The Lepidopterists 3(6-7): 144-150.

Cassino, S.E. and L.W. Swett. 1922b. Some new Geometrids. 3(8): 155-158.

Cassino, S.E. and L.W. Swett. 1922c. New Geometrids. The Lepidopterists 3(9): 159-166.

Cassino, S.E. and L.W. Swett. 1922d. Some new Geometridae. The Lepidopterists 3(10): 167-174.

Cassino, S.E. and L.W. Swett. 1922e. Some new Geometridae. The Lepidopterists 3(11): 175-180.

Cassino, S.E. and L.W. Swett. 1922f. Some new Geometridae. The Lepidopterists 3(12): 183-187.

Cassino, S.E. and L.W. Swett. 1923a. Some new Geometridae. The Lepidopterists 4(1): 1-8.

Cassino, S.E. and L.W. Swett. 1923b. New Geometrids. The Lepidopterists 4(2): 13-16.

Cassino, S.E. and L.W. Swett. 1923c. New Geometrids. The Lepidopterists 4(3): 18-24.

Cassino, S.E. and L.W. Swett. 1925a. Some new Geometridae. The Lepidopterists 4(5): 34-40.

Cassino, S.E. and L.W. Swett. 1925b. Some new Geometridae. The Lepidopterists 4(6-7): 41-47.

Cassino, S.E. and L.W. Swett. 1927. A new Geometrid genus The Lepidopterists 5(11): 41-47.

Castresana, L., A. Notario, and C. Iglesias. 1996. Nota sobre un Tortrícido, *Cacoecimorpha pronubana* (Hübner), que ataca a los pinos. [Note on a tortricid *Cacoecimorpha pronubana* (Hübner) which attacks pine trees.] Boletin de Sanidad Vegetal Plagas 22(2): 469-473.

Catling, P.M. 1997. Biology of the black-antenna race of *Phyciodes tharos tharos* (Nymphalidae) in Ontario. Journal of the Lepidopterists Society 51(3): 218-226.

Caton, B.P., T.T. Dobbs, and C.F. Brode. 2006. Arrivals of hitchhiking insect pests on international cargo aircraft at Miami International Airport. Biological Invasions 8(4): 765-785.

CDFA. 1993. Apple pith moth. California Plant Pest and Disease Report 12(1-2): 17-19.

Center, T.D., F.A. Dray, G.P. Jubinsky and M.J. Grodowitz. 1999. Insects and other arthropods that feed on aquatic and wetland plants. U.S. Department of Agriculture, Agricultural Research Service, Technical Bulletin No. 1870.

Chambers, V.T. 1871a. Micro-Lepidoptera. Canadian Entomologist 3(3): 54-58.

Chambers, V.T. 1871b. Micro-Lepidoptera. Canadian Entomologist 3(5): 84-88.

Chambers, V.T. 1871c. Micro-Lepidoptera. Canadian Entomologist 3(6): 108-112.

Chambers, V.T. 1871d. Micro-Lepidoptera. Canadian Entomologist 3(7): 127-130.

Chambers, V.T. 1871e. Micro-Lepidoptera. Canadian Entomologist 3(8): 146-149.

Chambers, V.T. 1871f. Micro-Lepidoptera. Canadian Entomologist 3(9): 161-166.

Chambers, V.T. 1871g. Micro-Lepidoptera. Canadian Entomologist 3(10): 182-185.

Chambers, V.T. 1871h. Micro-Lepidoptera. Canadian Entomologist 3(11): 205-209.

Chambers, V.T. 1871i. Micro-Lepidoptera. Canadian Entomologist 3(12): 221-224.

Chambers, V.T. 1872a. Micro-Lepidoptera. Canadian Entomologist 4(1): 126-133.

Chambers, V.T. 1872b. Micro-Lepidoptera. Canadian Entomologist 4(1): 169-175.

Chambers, V.T. 1872c. Micro-Lepidoptera. Canadian Entomologist 5(1): 12-15.

Chambers, V.T. 1874a. Micro-Lepidoptera. Canadian Entomologist 6(7): 128-130.

Chambers, V.T. 1874b. Micro-Lepidoptera. Canadian Entomologist 6(8): 149-153.

Chambers, V.T. 1874c. Micro-Lepidoptera. Canadian Entomologist 6(3): 49-52.

Chambers, V.T. 1874d. Tineina from Texas. Canadian Entomologist 6(12): 230-249.

Chambers, V.T. 1875a. Teneina (sic) of Colorado. Cincinnati Quarterly Journal of Science 2(4): 289-305.

Chambers, V.T. 1875b. Tineina from Texas. Canadian Entomologist 7(2): 30-35.

Chambers, V.T. 1875c. Tineina from Texas. Canadian Entomologist 7(3): 51-56.

Chambers, V.T. 1877. The Tineina of Colorado. Bulletin of the United States Geological Survey 3(1): 121-145.

Chambers, V.T. 1878. Art. III. Descriptions of new Tineina from Texas, and others from more northern localities. Bulletin of the United States Geological and Geographical Survey of the Territories 4(1): 79-106.

Chambers, V.T. 1880. Descriptions of some new Tineina, with notes on a few old species. Journal of the Cincinnati Society of Natural History 2(4): 179-194.

Chambers, V.T. 1881. New species of Tineina. The Journal of the Cincinnati Society of Natural History 3(4): 289-296.

Chang, P.E.C. 2018. Panama moths: notes on the life history of *Gonodonta incurva* (Sepp, [1840]) (Erebidae, Calpinae). nternational Journal of Avian & Wildlife Biology 3(6): 405-407.

Chapman, P.J. and S.E. Lienk. 1971. Tortricid Fauna of Apple in New York (Lepidoptera: Tortricidae). State Agricultural Experimental Station, Special Publication, Cornell University. Ithaca, New York.

Charlet, L.D. 2001. Biology and seasonal abundance of parasitoids of the banded sunflower moth (Lepidoptera: Tortricidae) in sunflower. Biological Control 20(2): 113-121.

Chazal, A.C. and C.S. Hobson. 2002. Status of the rare skipper (*Problema bulenta*) in Virginia. Banisteria (Virginia Natural History Society) 19: 20-22.

Chazal, A. C. and N. E. Van Alstine. 2002. Plants and animals recorded from the Jamestown Island 400 Project Area, Colonial National Historical Park. Natural Heritage Technical Report 02-01. Virginia Department of Conservation and Recreation, Division of Natural Heritage, Richmond, Virginia. Unpublished report submitted to Colonial National Historical Park. January 2002. 69 pages.

Chen, T.B., X.H. Dai, and C. Eiseman. 2022. A checklist of gymnosperm-feeding leafminers (Arthopoda, Insecta) in North America and Europe. Biodiversity Data Journal 10 (2022): e91313; 1-25.

Chesmore, D. 2003. *Scythris inspersella* (Hübner) (Lepidoptera: Scythrididae): A new species in Yorkshire. British Journal of Entomology and Natural History 16(1): 6.

Chippendale, G.M., F. Breden, and D.E. McCauley. 1992. Factors controlling the distribution of a subtropical corn borer, *Diatraea grandiosella* (Lepidoptera: Pyralidae). Studies on Neotropical Fauna and Environment 27(2-3): 83-90.

Cho, S.W., A. Mitchell, C. Mitter, J. Regier, M. Matthews, and R. Robertson. 2008. Molecular phylogenetics of heliothine moths (Lepidoptera: Noctuidae: Heliothinae), with comments on the evolution of host range and pest status. Systematic Entomology 33: 581-594.

Choi, S.W. 2001. Phylogeny of *Eulithis* Hübner and related genera (Lepidoptera: Geometridae), with an implication of wing pattern evolution. American Museum Novitates 3318: 1-37.

Choi, S.W. and J.C. Miller. 2013. Species richness and abundance among macromoths: A comparison of taxonomic, temporal and spatial patterns in Oregon and South Korea. Entomological Research 43(6): 312-321.

Chou, I. (ed.). 1994. Monographia Rhopalocerorum Sinensium. [Monograph of Chinese Butterflies, Second Volume]. 1-2: 1-854. Henan Science and Technology Publishing House, China.

Chuah, H. and D.S. Cushing. 1995. *Eurema albula* (Pieridae) and *Anthanassa argentea* (Nymphalidae): new records for the United States. Tropical Lepidoptera 6(1): 43-44.

Chun, S., A. Hara, R. Niino-DuPonte, W. Nagamine, P. Conant, and C. Hirayama. 2005. Stinging Nettle Caterpillar *Darna pallivitta.* Pest Alert. College of Tropical Agriculture and Human Resources, University of Hawai‘i at Mänoa, Manoa, Hawaii., U.S.A.

Claflin, S.H. and D.C. Allen. 1981. Biology of *Ancylis discigerana* (Lepidoptera: Tortricidae). The Canadian Entomologist 113(4): 265-270.

Clark, A.H. and L.F. Clark. 1952. The butterflies of Virgina. Smithsonian Miscellaneous Collections 116(7): 1-129.

Clark, S.E., R.G. Van Driesche, N. Sturdevant, and S. Kegley. 2001. Comparative efficacy of adult and larval sampling for detection of the root-boring insects *Agapeta zoegana (*Lepidoptera: Cochylidae) and *Cyphocleonus achates* (Coleoptera: Curculionidae) released for biological control of spotted knapweed. Environmental Entomology 30(3): 589-594.

Clarke, J.F.G. 1932. New Microlepidoptera from the Pacific Coast (Gelechiidae). The Canadian Entomologist 64: 63-69.

Clarke, J.F.G. 1933. Notes and new species of Microlepidoptera from Washington State. Canadian Entomologist 65(4): 84-93, 2 plates.

Clarke, J.F.G. 1935. Notes and new species of Microlepidoptera from Washington State. Canadian Entomologist 67: 244-254.

Clarke, J.F.G. 1939. A new geometrid genus from North America with a discussion of its type (Lepidoptera). Proceedings of the Entomological Society of Washington 41(3): 73-75.

Clarke, J.F.G. 1940. United States records of tropical American Lepidoptera. Proceedings of the Entomological Society of Washington 42(7): 155-156.

Clarke, J.F.G. 1941a. Revision of the North American moths of the family Oecophoridae, with descriptions of new genera and species. Proceedings of the United States National Museum 90: 33-286.

Clarke, J.F.G. 1941b. The North American moths of the genus *Arachnis*, with one new species. Proceedings of the United States National Museum 91(3123): 59-70, plus plates 10-12.

Clarke, J.F.G. 1942a. Notes and new species of Microlepidoptera from Washington state. Proceedings of the United States National Museum 92(3149): 267-276 plus plates 27-32.

Clarke, J.F.G. 1946. Synopsis of the genus *Nealyda* Dietz, with descriptions of new species (Gelechiidae: Lepidoptera). Journal of the Washington Academy of Sciences 36(12): 425-427.

Clarke, J.F.G. 1947a. Notes on, and new species of, American moths of the genus *Filatima* Busck (Gelechiidae: Lepidoptera). Journal of the Washington Academy of Science 37: 263-275.

Clarke, J.F.G. 1947b. New North American species and new assignments in the genus Chionodes (Lepidoptera: Gelechiidae). Journal of the Washington Academy of Sciences 37(7): 243-254.

Clarke, J.G. 1947c. Notes on Oecophoridae, with descriptions of new species. Journal of the Washington Academy of Sciences 37(1): 2-18.

Clarke, J.G. 1947d. The fauna and flore of the El Segundo Sand Dunes. 16. A new Eucosma from the El Segundo Sand Dunes. Bulletin of the Southern California Academy of Sciences 66: 51-53.

Clarke, J.G. 1950. A new genus and three new species of Microlepidoptera from California (Ethmiidae). Journal of the Washington Academy of Sciences 40(5): 161-163.

Clarke, J.G. 1951. A new genus and species of North American Olethreutidae (Lepidoptera: Laspeyresiinae). Journal of the Washington Academy of Sciences 41(1): 46-47.

Clarke, J.F.G. 1953. Notes, new synonymy, and new assignments in American Gelechiidae. Journal of the Washington Academy of Science 43(10): 317-320.

Clarke, J.F.G. 1963. Catalogue of the type specimens of Microlepidoptera in the British Museum (Natural History) described by Edward Meyrick. Volume 5. Timyridae Cosmopterigidae Hyponomeutidae Walshiidae Ethmiidae Blastodacnidae Metachandidae Scythridae. Trustees of the British Museum of Batural History.

Clarke, J.F.G. 1965. Microlepidoptera of Juan Fernandez Island. Proceedings of the United States National Museum 117(3508): 1-105.

Clarke, J.F.G. 1969. Catalogue of the type specimens of Microlepidoptera in the British Museum (Natural History) described by Edward Meyrick. Volume 7. Gelechiidae (D-Z). Trustees of the British Museum of Natural History.

Clarke, J.F.G. 1973. The genus *Eumarozia* Heinrich (Olethreutidae). Journal of the Lepidopterists’ Society 27(4): 268-274.

Clarke, J.F.G. 1987. The correct identity of *Acleris inana* (Robinson) (Lepidoptera: Tortricidae). Proceedings of the Entomological Society of Washington 89(1): 175-176.

Clarke, J.F.G. 1990. A new species of *Mompha* (Momphidae) from the Queen Charlotte Islands, British Columbia. Journal of the Lepidopterists' Society 44(4): 252-256.

Clarke, J.F.G. and F.H. Benjamin. 1938. A study of some North American moths allied to the thyatirid genus Bombycia Hübner. Bulletin of the Southern California Academy of Sciences 37: 55-73.

Clarke, J.M. 1920. Additions to collections, October 16, 1917 - October 15, 1818. 34th report of the state Entomologist on injurious and ther insects of the state of New York 1918. New York State Museum Bulletin 231: 73-79.

Clavijo, A.J.A. 1990. Systematics of black and white species of the genus Diaphania Hübner (1818) (Lepidoptera: Pyralidae: Pyraustinae). Doctoral dissertation, Dissertation. McGill University, Montreal, Canada. 215 p.

Clavijo-Albertos, J. and Q. Arias-Celis. 2017. Catálogo de los Spilomelinae de Venezuela (Lepidoptera: Crambidae). SHILAP Revista de Lepidopterología 45(177): 129-141.

Clemens, B. 1864. North American Micro-Lepidoptera. Proceedings of the Entomological Society of Philadelphia 2: 423.

CLEV:CMNHENT. 2015. Cleveland Museum of Natural History Invertebrate Zoology Collection, Cleveland, Ohio. In: SCAN. P.L. Heinrich, E. Gilbert, N.S. Cobb, and N. Franz. Symbiota collections of arthropods network (SCAN): A data portal built to visualize, manipulate, and export species occurrences. [Dataset]. http://scan-bugs.org/portal/collections/index.php [Accessed 09 March 2019]

Cock, M.J.W. 1986. The skipper butterflies (Hesperiidae) of Trinidad. Part 4, Pyrginae (second section). Living World, Journal of the Trinidad and Tobago Field Naturalists’ Club 1985-1986: 33-47.

Cock, M.J.W. 2003a. On the number of moths (Lepidoptera) that occur in Trinidad and Tobago. Living World Journal of Trinidad and Tobago Field Naturalist’s Club 2003: 49-58.

Cock, M.J.W. 2003b. The skipper butterflies (Hesperiidae) of Trinidad. Part 11, Hesperiinae, genera group O. Living World, Journal of the Trinidad and Tobago Field Naturalists' Club 2003: 14-48.

Cock, M.J.W. 2012. The skipper butterflies (Hesperiidae) of Trinidad. Part 19. Hesperiinae, Moncini: the remaining genera with pale spots: *Cymaenes,* *Cobalopsis*, *Arita*, *Lerema*, *Morys* and *Tigasis*. Living World, Journal of the Trinidad and Tobago Field Naturalists’ Club 2012: 20-40.

Cock, M.J.W. 2013. The skipper butterflies (Hesperiidae) of Trinidad. Part 20. Hesperiinae, Moncini: The remaining genera of mostly unmarked brown species: *Eutocus, Eprius, Mnasicles, Methionopsis, Sodalia, Thargella, Nastra, Mnasilus, Mnasitheu*s and *Papias.* Living World, Journal of the Trinidad and Tobago Field Naturalists’ Club 2013: 1-18.

Cock, M.J.W. 2014. Observations on the biology of skipper butterflies in Trinidad, Trinidad and Tobago: *Phocides, Chioides, Typhedanus*, and *Polythrix* (Hesperiidae: Eudaminae). Living World, Journal of the Trinidad and Tobago Field Naturalists’ Club 2014: 1-11.

Cock, M.J.W. 2015. Observations on the biology of skipper butterflies in Trinidad, West Indies: *Urbanus*, *Astraptes* and *Narcosius* (Hesperiidae: Eudaminae). Living World, Journal of the Trinidad and Tobago Field Naturalists’ Club 2015: 1-14.

Cock, M.J.W. 2017. A preliminary catalogue of the moths (Lepidoptera except Papilionoidea) of Tobago, West Indies. Insecta Mundi 0585: 1-58.

Cock, M.J.W. 2018. Hawk-moths (Lepidoptera: Sphingidae) of Trinidad, West Indies: An illustrated and annotated list. Living World, Journal of Trinidad and Tobago Field Naturalists’ Club 2018: 10-81.

Cock, M.J.W. and D.H. Burris. 2013. Neotropical palm-inflorescence feeding moths (Lepidoptera: Batrachedridae, Blastobasidae, Cosmopterigidae, Gelechiidae, Pyralidae, Tineidae): A review of the literature and new records from Trinidad, West Indies. Journal of Research on the Lepidoptera 46: 1-21.

Cock, M.J.W., R.N. Deo, M.C. Gibson, A.E. Deacon, S.M. Tran, M. Kelly, and A. Wheeler. 2023. New records of butterflies and moths (Lepidoptera) from Tobago, West Indies, with two new combinations and one new synonym in Erebidae. Living World, Journal of the Trinidad and Tobago Field Naturalists Club 2023: 99-125.

Cock, M.J.W., C. Mejias, A.E. Deacon, R.N. Deo, M. Kelly, and D.R.W. Pandey. 2024. More than 100 new records of moths and butterflies (Lepidoptera) from Tobago, West Indies, with a new synonym in Crambidae. Living World, Journal of the Trinidad and Tobago Field Naturalists Club 2024: 52-100.

Cockerell, T.D.A. 1898. Second note on a new *Hemileuca*. Psyche 8(272): 298-298.

Cockerell, T.D.A. 1913. Lepidoptera bredding on evening primrose. Journal of Economic Entomology 6(6): 489.

Cockerell, T.D.A. 1920. The generic position of *Sphinx separatus* Neum. Canadian Entomologist 52(2): 33-34.

Cognato, A.I., W. Taft, R.K. Osborn, and D. Rubinoff. 2023. Multi‐gene phylogeny of North American clear‐winged moths (Lepidoptera: Sesiidae): a foundation for future evolutionary study of a speciose mimicry complex. Cladistics 39(1): 1-17.

Collins, M.M. 1973. Notes on the taxonomic status of *Hyalophora columbia* (Saturniidae). Journal of the Lepidopterists’ Society 27: 225-235.

Collins, M.M. 1984. Genetic and ecology of a hybrid zone in *Hyalophora* (Lepidoptera: Saturniidae). University of California Publications in Entomology 104: 1-93.

Collins, M.M. and R.D. Weast, 1961. Wild silk moths of the United States. Saturniinae. Experimental studies and observations of natural living habits and relationships. Collins Radio Company, Cedar Rapids. 138 pp.

Combs, J. K., S.H. Reichard, M.J. Groom, D.L. Wilderman, and P.A. Camp. 2011. Invasive competitor and native seed predators contribute to rarity of the narrow endemic *Astragalus sinuatus* Piper. Ecological Applications 21(7): 2498-2509.

Common, I.F.B. 1990. Moths of Australia. Melbourne University Press, Carlton. 535 pp.

Common, I.F.B. 1997. Oecophorine genera of Australia. II. The Chezala, Philobota and Eulechria groups (Lepidoptera: Oecophoridae), Monographs on Australian Lepidoptera. CSIRO Publishing, Australia 5: 1-407.

Comstock, J.H. 1880. The pale *Dakruma*. Report of the Commissioner of Agriculture for the Year 1879 pp. 243-244.

Comstock, J.A. 1920. An Introduction to Entomology. The Comstock Publishing Co., Ithaca, New York.

Comstock, J.A. 1930. Studies in the Pacific Coast Lepidoptera (continued). Bulletin of the Southern California Academy of Sciences29: 22-31.

Comstock, J.A. 1938. A new race of *Euproserpinus phaeton* from the Mojave Desert (Lepidoptera: Sphingidae). Bulletin of the Southern California Academy of Science37: 33-42.

Comstock, J.A. 1947. *Phlegethontius* Fabr. in California (Lepidoptera; Sphingidae). Bulletin of the Southern California Academy of Sciences 45: 145-147.

Comstock, J.A. 1949. An Introduction to Entomology. 9th Edition. The Comstock Publishing Co., Ithaca, New York.

Comstock, J.A. 1955a. The Egg, and first larval instar of a Geometrid moth from Arizonia. Bulletin of the Southern California Academy of Sciences 54(2): 103.

Comstock, J.A. 1957a. Scientific notes. Bulletin of the Southern California Academy of Sciences 56(3): 178-179.

Comstock, J.A. 1957b. Early stages of *Eutachyptera psidii* (Lasiocampldae), a rare moth from southern Arizona. The Lepidopterists' News 11(4-5): 99-102.

Comstock, J.A. 1958. The ova and first larval stages of three southwestern moths. Bulletin of the Southern California Academy of Sciences 57(1): 34-38.

Comstock, J.A. 1959. Entomological briefs. Bulletin of the Southern California Academy of Sciences 58(3): 169-171.

Comstock, J.A. 1960. Life history notes on a Saturniid and two Lasiocampid Moths from California. Bulletin of the Southern California Academy of Sciences 59: 170-181.

Comstock, J.A. 1962. Life history notes on *Epiplatymetra grotearia* Packard.. Bulletin of the Southern California Academy of Sciences 61 (1): 3-7.

Comstock, J.A. 1963a. Notes on the early stages of two California geometrids. Journal of Research on the Lepidoptera 1(3): 195-200.

Comstock, J.A. 1963b. Notes on the ova of six California moths. Bulletin of the Southern California Academy of Sciences 62(4): 202-208.

Comstock, J.A. and A.B. Comstock.1920. A Manual for the Study of Insects,

Comstock, J.A. and C.M. Dammers. 1936. Notes on the early stages of five moths from southern California. Bulletin of the Southern California Academy of Sciences 35(2): 99-107.

Comstock, J.A. and C.M. Dammers. 1937. Notes on the early stages of three California moths. Bulletin of the Southern California Academy of Sciences 36: 68-78.

Comstock, J.A. and C.M. Dammers. 1946. Notes on the life history of *Anacamptodes* (*Cleora*) *fragilaria* Grossb. (Lepidoptera). Bulletin of the Southern California Academy of Sciences 45: 17-20, plates 3-5.

Comstock, J.A. and L.V. García. 1963. Estudios de los ciclos biológicos en lepidópteros Mexicanos II. Heterocera. Anales del Instituto de Biología México 34: 217-273.

Comstock, J.A. and C. Henne. 1942. Notes on the life history of *Tolype glenwoodii* Barnes. Bulletin of the Southern California Academy of Sciences 41: 86-90.

Comstock, J.A. and C. Henne. 1943a. The early stages of *Aretonotus lucidus* Bdv. (Lepidopt.) Bulletin of the Southern California Academy of Sciences 41:167-171.

Comstock, J.A. and C. Henne. 1964. Studies in life histories of North American Lepidoptera. California *Annaphilas.* Journal of Research on the Lepidoptera 3(3): 173-191.

Comstock, J.A. and C. Henne. 1965a. Notes on the life history *Philotes enoptes dammersi*. Bulletin of the Southern California Academy of Science 64(3): 153-156.

Comstock, J.A. and C. Henne. 1966a. The larva and pupa of *Orthosia hibisci quinquefasciata* (Noctuidae). Journal of the Lepidopterist Society 20(4): 213-215.

Comstock, J.A. and C. Henne. 1966b. Studies in life histories of North American Lepidoptera. California *Annaphilas* II*.* Journal of Research on the Lepidoptera 5(1): 15-26.

Comstock, J.A. and C. Henne. 1967. Early stages of *Lycomorpha regulus* Grinnell, with notes on the imago (Lepidoptera: Amatidae). Journal of the Lepidopterist Society 21(4): 275-280.

Comstock, J.A. and C. Henne. 1969. Life history notes on *Lithophane subtilis* (Noctuidae). Journal of the Lepidopterist Society 23(1): 15-18.

Comstock, J.A. and L.G. Vázquez. 1964. Estudios de los ciclos biologicos en Lepidopteros Mexicanos. Anales del Instituto de Biologia. Universidad Autonoma de México, (Serie Zoologia) 34: 217-273.

CONABIO:CCNBM. 2015. Computarización de la Colección Nacional de insectos Dr. Alfredo Barrera Marín del Museo de Historia Natural de la Ciudad de México, istrito Federal, México. In: SCAN. P.L. Heinrich, E. Gilbert, N.S. Cobb, and N. Franz. Symbiota collections of arthropods network (SCAN): A data portal built to visualize, manipulate, and export species occurrences. [Dataset]. http://scan-bugs.org/portal/collections/index.php [Accessed 08 March 2019]

CONABIO-BST. 2015. Biodiversidad de la Sierra Tarahumara. Lepidoptera: Mimallonoidea, Lasiocampoidea, Bombycoidea y Pyraloidea. Comisión nacional para el conocimiento y uso de la biodiversidad. In: SCAN. P.L. Heinrich, E. Gilbert, N.S. Cobb, and N. Franz. Symbiota collections of arthropods network (SCAN): A data portal built to visualize, manipulate, and export species occurrences. [Dataset]. http://scan-bugs.org/portal/collections/index.php [Accessed 08 March 2019]

Conant, P. 2002. Classical biological control of *Clidemia hirta* (Melastomataceae) in Hawaii using multiple strategies. pp. 13-20 in Smith, Clifford W., Julie Denslow, and Stephen Hight (eds). 2002. Proceedings of workshop on biological control of native ecosystems in Hawaii. Pacific Cooperative Studies Unit (University of Hawaii at Manoa, Department of Botany) Technical Report 129.

Condamine, F.L., R. Allio, E. L. Reboud, J.R. Dupuis, E.F.A. Toussaint, N. Mazet, S-J. Hu, D.S. Lewis, K. Kunte, A.M. Cotton, F.A.H. Sperling, 2023. A comprehensive phylogeny and revised taxonomy illuminate the origin and diversification of the global radiation of Papilio (Lepidoptera: Papilionidae). Molecular Phylogenetics and Evolution, 183: 1-22.

Condrashoff, S.F. 1964. Bionomics of the aspen leaf miner, *Phyllocnistis populiella* Cham. (Lepidoptera: Gracillariidae). Canadian Entomologist 96(6): 857-875.

Cong, Q. and N.V. Grishin. 2014. A new *Hermeuptychia* (Lepidoptera, Nymphalidae, Satyrinae) is sympatric and synchronic with *H. sosybius* in southeast US coastal plains, while another new *Hermeuptychia* species - not *hermes* - inhabits south Texas and northeast Mexico. ZooKeys 379: 43-91. doi: 10.3897/zookeys.379.6394.

Cong, Q., J. Zhang, J. Shen, and N.V. Grishin, 2019. Fifty New Genera of Hesperiidae (Lepidoptera). Insecta Mundi, 0731: 1-56.

Cong, Q., J. Zhang, J.H. Shen, X.L. Cao, C. Brévignon, and N.V. Grishin. 2020. Speciation in North American *Junonia* from a genomic perspective. Systematic Entomology 45(4): 803-837.

Cooley, T.J. and R.C. Reardon. 2022. Field guide to the slug moths (Lepidoptera: Limacodidae) of West Virginia. United States Department of Agriculture, Forest Service, Forest Health Assessment and Applied Sciences Team, FHAAST-2019-06.

Copley, C.R. and R.A. Cannings. 2005. Notes on the status of the Eurasian moths *Noctua pronuba* and *Noctua comes* (Lepidoptera: Noctuidae) on Vancouver Island, British Columbia. Journal of the Entomological Society of British Columbia 102: 83-84.

Cordero, R.J., R.L. Brown, and H.N. Pitre. 1999. Description of life stages and distribution of *Metaponpneumata rogenhoferi* (Lepidoptera: Noctuidae). Tropical Lepidoptera Research 10(2): 59-67.

Cordero, R.J., H.N. Pitre, and R.D. Cave. 2002. Development of *Metaponpneumata rogenhoferi* on maize, sicklepod, and wheat germ artificial diet. Tropical agriculture 79(2): 133-136.

Cordo, H.A. and C.J. DeLoach. 1987. Insects That Attack Mesquite ( *Prosopis* spp.) in Argentina and Paraguay. United States Department of Agriculture, Agriculture Research Service ARS-62. 32 pp.

Corley, M.F.V. 1992. The summer brood of *Dichrorampha acuminatana* (Lienig & Zeller) (Lep., Tortricidae). Entomologist’s Record and Journal of Variation 104: 159-160.

Corrette, K. B. and H.H. Neunzig. 1979. Descriptions of and notes on larval habits of four immature phycitines in the southeastern United States (Lepidoptera: Pyralidae). Annals of the Entomological Society of America 72(5): 690-699.

Csóka, G. and C. Szabóky. 2005. Checklist of herbivorous insects of native and exotic oaks in Hungary I. (Lepidoptera). Acta Silvatica et Lignaria Hungarica 1: 59-72.

Corro Chang, P.E., O. Bidzilya, M.A. Metz, J.-F. Landry, and J. Hepppner. 2021. An updated and annotated checklist of the Gelechiidae (Lepidoptera) of Panamá. Proceedings of the Entomological Society of Washington 123(1): 55-74.

COSEWIC. 2003. COSEWIC assessment and status report on the sand-verbena moth *Copablepharon fuscum* in Canada. Committee on the Status of Endangered Wildlife in Canada. Ottawa. vii + 39 pp. ([www.sararegistry.gc.ca/status/status_e.cfm](http://www.sararegistry.gc.ca/status/status_e.cfm)) [author N. Page]

COSEWIC. 2005. COSEWIC assessment and status report on the White Flower Moth Schinia bimatris in Canada. Committee on the Status of Endangered Wildlife in Canada. Ottawa. vi + 20 pp.

Cossentine, J.E., G.J.R. Judd, J.D. Bissett, and L.A Lacey. 2010. Susceptibility of apple clearwing moth larvae, *Synanthedon myopaeformis* (Lepidoptera: Sesiidae) to *Beauveria bassiana* and *Metarhizium brunneum*. Biocontrol Science and Technology 20(7): 703-707. DOI: 10.1080/09583151003690390

Coulson, R.N. and R.T. Franklin. 1970. The occurrence of *Dioryctria amatella* and other insects in *Cronartium fusiforme* cankers. The Canadian Entomologist 102(3): 353-357.

Covell, C.V. 1965. A revision of the North American species of the genus Scopula (Lepidoptera, Geometridae). PhD dissertation, Virginia Polytechnic Institute, Blacksburg, VA.

Covell, C.V. Jr. 1970. A revision of the North American species of the genus *Scopula* (Lepidoptera, Geometridae). Transactions of the American Entomological Society 96(2): 101-221.

Covell, C.V. Jr. 1984. A field guide to the moths of Eastern North America. Houghton Mifflin Co., Boston, MA.

Covell, C.V. Jr. 1989. Additions to the Kentucy faunal list. Kentucy Lepidopterist 15(3): 2-3.

Covell, C.V. Jr. 1999. The Butterflies and Moths (Lepidoptera) of Kentucky: An Annotated Checklist. Kentucky State Nature Preserves Commission Scientific and Technical Series, Frankford, Kentucy. 220 pages.

Covell, C.V. Jr. 2011a. Reports of state coordinators. Florida. Southern Lepidopterists' News 33(4): 188-189.

Covell, C.V. Jr. 2011b. *Idaea asceta* (Prout) (Geometridae: Sterrhinae) from Texas, new to the North American fauna. News of the Lepidopterists’ Society 53(3): 79.

Covell, C.V. Jr. 2013 Two Mexican Geometridae new to the United States, with a new synonymy. Tropical Lepidoptera Research 24(1): 59-60.

Covell, C.V. Jr. 2015. Three new species of *Idaea* Treitschke (Geometridae: Sterrhinae) from the Southwestern United States and northern Mexico. The Journal of the Lepidopterists' Society 69(4): 317-325.

Covell, C.V. Jr. and L.D. Gibson. 2008. More new moth records (Lepidoptera) from Kentucky. Journal of the Kentucky Academy of Science 69(2): 193-196.

Covell, C.V. Jr. and J.E. Hayden. 2014. *Lineodes multisignalis* Herrich-Schäffer (Crambidae, Spilomelinae) -new to the U. S. News of the Lepidopterists’ Society 56(3): 135-136.

Covell, C.V. Jr. and E.H. Metzler. 1992. Two new species of moths (Noctuidae: Acronictinae, Cuculliinae) from midland United States. Journal of the Lepidopterists' Society, 46(3), 220-232.

Covell, C.V. Jr., I.L. Finkelstein, and A.A. Towers, 1984. A new species of *Narraga* Walker (Geometridae, Ennominae) from Georgia, with biological notes. Journal of Research on the Lepidoptera 23(2): 161-168

Covell, C.V. Jr., D.C. Ferguson, and G.B. Straley. 1986. *Ennomos alniaria* (Lepidoptera: Geometridae), a European moth recently discovered in British Columbia. The Canadian Entomologist 118(5): 499-501.

Covell, C.V. Jr., L.D. Gibson, and D.J. Wight. 2000. New state records and new available names for species of Kentucky moths (Insect: Lepidoptera). Journal of the Kentucky Academy of Science 62(2): 105-107.

Cowper, G. 2008. Society meeting of November 16, 2005. Entomological News 119(3): 306-306.

Coyle, D.R., J. Adams, E. Bullas-Appleton, J. Llewellyn, A. Rimmer, M.J. Skvarla, S.M. Smith, and J. H. Chong. 2022. Identification and management of *Cydalima perspectalis* (Lepidoptera: Crambidae) in North America." Journal of Integrated Pest Management 13(1): 24; 1-8.

Coyne, J.F. 1968. *Laspeyresia ingens*, a seedworm infesting cones of longleaf pine. Annals of the Entomological Society of America 61(5): 1116-1122.

Crabo, A. and L. Crabo. 2007. A new species of *Euxoa* (Noctuidae) from Washington State. Journal of the Lepidopterists’ Society 61(2): 87-89.

Crabo, L.G. 2015. A new species of *Ogdoconta* Butler (Lepidoptera, Noctuidae, Condicinae, Condicini) from southeastern Arizona, USA. In: B.C. Schmidt and J.D. Lafontaine, (Eds.) Contributions to the systematics of New World macromoths VI. ZooKeys 527: 51-56. doi: 10.3897/zookeys.527.9771

Crabo, L.G. 2018. A new genus and three new species of noctuid moths from western United States of America and Mexico (Lepidoptera, Noctuidae, Noctuinae, Eriopygini). In: B.C. Schmidt and J.D. Lafontaine, (eds.). Contributions to the systematics of New World macro-moths VII. ZooKeysZooKeys 788: 183-199. https://doi.org/10.3897/zookeys.788.26068

Crabo, L.G. 2024. A new noctuid genus and species (Lepidoptera, Noctuidae, Amphipyrinae, Psaphidini, Triocnemidina) from New Mexico and Texas, United States of America. ZooKeys 1200: 199-213.

Crabo, L.G. and P.C. Hammond, 1996. A revision of *Mesogona* Boisduval (Lepidoptera: Noctuidae) for North America with descriptions of two new species. Journal for Research on the the Lepidoptera 34: 83-98.

Crabo, L.G. and D.G. Holden. 2020. A review of established accidentally-introduced Noctuoidea in the Pacific Northwest Region of North America, with first report of the Eurasian Cutworm *Mesapamea secalis* (L.) (Noctuidae, Noctuinae, Apameini) for North America. Journal of the Lepidopterists' Society 74(2): 95-105.

Crabo, L.G., and K. Keegan. 2025. Revision of the North American genus *Supralathosea* Barnes & Benjamin (Lepidoptera, Noctuidae, Oncocnemidinae) with description of two genera and three species. ZooKeys 1228: 197-223.

Crabo, L.G. and J.D. Lafontaine. 1997. A revision of the *Cerastis cornuta* group of the genus *Cerastis* subgenus *Metalepsis* (Noctuidae). Journal of the Lepidopterists’ Society 51(3): 237-248.

Crabo, L.G. and J.D. Lafontaine. 2009. A revision of *Lasionycta* Aurivillius (Lepidoptera, Noctuidae) for North America and notes on Eurasian species, with descriptions of 17 new species, 6 new subspecies, a new genus, and 2 new species of *Tricholita* Grote. ZooKeys 30: 1-156.

Crabo, L.G. and B.C. Schmidt. 2018. A revision of *Admetovis* Grote, with the description of a new species from western North America (Noctuidae, Noctuinae, Hadenini). In: B.C. Schmidt and J.D. Lafontaine, (eds.). Contributions to the systematics of New World macro-moths VII. ZooKeys 788: 167-181. https://doi.org/10.3897/zookeys.788.26480

Crabo, L.G., M. Davis, P. Hammond, T. Mustelin, and J. Shepard. 2013. Five new species and three new subspecies of Erebidae and Noctuidae (Insecta, Lepidoptera) from Northwestern North America, with notes on *Chytolita* Grote (Erebidae) and Hydraecia Guenée (Noctuidae). In: B.C. Schmidt J.D. Lafontaine, (Eds.). Contributions to the systematics of New World macro-moths IV.ZooKeys 264: 85-123.

Crabo, L.G., R. Zack, and M. Peteerson. 2014. Pacific Northwest Moths. http://pnwmoths.biol.wwu.edu/ [Accessed 1April2014] [accessed MARCH 2020]

Crabo, L.G., P.C. Hammond, T. Mustelin, and D.L. Wikle. 2018. Six new species and one new subspecies of noctuid moths from western United States of America and Mexico (Lepidoptera, Noctuidae). In: B.C. Schmidt and J.D. Lafontaine, (eds.). Contributions to the systematics of New World macro-moths VII. ZooKeys 788: 201-239. https://doi.org/10.3897/zookeys.788.26282

Crabtree, L.L. and R. Leuschner. 2000. Records for the utilization of *Prunus* as a larval foodplant by 71 species of Lepidoptera in northeast California. The Taxonomic Report of the International Lepidoptera Survey 2(7): 1-7.

Cram, W.T. and N.V. Tonks. 1959. Note on the occurrence in British Columbia of the omnivorous leaf tier, *Cnephasia longana* (Haw.) (Lepidoptera: Tortricidae), as a pest of strawberry. Canadian Entomologist 91: 155-156.

Cranshaw, W., D. Leatherman, and B. Kondratieff. 1994. Insects that feed on Colorado trees and shrubs. Colorado State University Cooperative Extension Bulletin 506A.

Cranshaw, W. 2019. Note on predation of elm leaf beetle by a noctuid larva, *Caradrina montana* (Bremer). Southwestern Entomologist 44(1): 343-344.

Crawford, C.S. 1971a. Primary geo-orientation in moths, sod webworm moths. Journal of Research on the Lepidoptera 9(2): 65-74.

Crawford, C.S. 1971b. Comparative reproduction of *Crambus harpipterus* and *Agriphila plumbifimbriella* in northern New Mexico. Annals of the Entomological Society of America 64(1): 52-59.

Crevecoeur, F.F. ‎1919. Additions to the List of Kansas Lepidoptera. Transactions of the Kansas Academy of Science 30: 377-385.

Crolla, J.P. 2004. 2003 Season Summary. Zone 7 Ontario/Quebec Ontario, Quebec. News of the Lepidopterist’ Society 46(S1): 49-56.

Crolla, J.P. 2005. 2004 Season Summary. Zone 7 Ontario/Quebec Ontario, Quebec. News of the Lepidopterist’ Society 47(S1): 58-68.

Crolla, J.P. 2006. 2005 Season Summary. Zone 7 Ontario/Quebec Ontario, Quebec. News of the Lepidopterist’ Society 48(S1): 68-80.

Crolla, J.P. 2007. 2006 Season Summary. Zone 7 Ontario/Quebec Ontario, Quebec. News of the Lepidopterist’ Society 49(S1): 77-86.

Crolla, J.P. 2008. 2007 Season Summary. Zone 7 Ontario/Quebec Ontario, Quebec. News of the Lepidopterist’ Society 50(S1): 98-111.

Crolla, J.P. 2009. 2008 Season Summary. Zone 7 Ontario/Quebec Ontario, Quebec. News of the Lepidopterist’ Society 51(S1): 83-90.

Crolla, J.P. 2011. 2010 Season Summary. Zone 7 Ontario/Quebec Ontario, Quebec. News of the Lepidopterist’ Society 53(S1): 100-108.

Cromartie, W.J. and D.F. Schweitzer, 1993. Biology of the rare skipper, Problema bulenta (Hesperiidae), in southern New Jersey. Journal of the Lepidopterists’ Society 47(2): 125-133.

Cronin, J.P. 2006. Explaining Variation in Insect Herbivore Control over Plant Communities. Doctoral Dissertation, University of Pittsburgh, Pittsburgh, Pennsylvania.

Crotch, W.J.B. 1956. A silkmoth rearer’s handbook being volume twelve of “The Amateur Entomologist” based on volume six edited by Beowulf. A. Cooper, B.Sc., A.R.C.S. in 1942 but substantially re-written, revised, re-illustrated and expanded. Amateur Entomologist 12: 1-165.

Crumb, S.E. 1956. The larvae of the Phalaenidae. United States Department of Agriculture Technical Bulletin no. 1135. U.S. Government Printing Office, Washington, DC.

Cryan, J.F. and R.E. Dirig. 1977. The moths of autumn. Vol. 1. Moths at the Pine Bush. Pine Bush Historic Preservation Project Occasional Paper 1: 1-19.

CSU:CSUC. 2015. C.P. Gillette Museum of Arthropod Diversity, Colorado State University, Fort Collins, Colorado. In: SCAN. P.L. Heinrich, E. Gilbert, N.S. Cobb, and N. Franz. Symbiota collections of arthropods network (SCAN): A data portal built to visualize, manipulate, and export species occurrences. [Dataset]. http://scan-bugs.org/portal/collections/index.php [Accessed 08 March 2019]

CU:CUAC. 2015. Clemson University Arthropod Collection, Clemson, South Carolina. In: SCAN. P.L. Heinrich, E. Gilbert, N.S. Cobb, and N. Franz. Symbiota collections of arthropods network (SCAN): A data portal built to visualize, manipulate, and export species occurrences. [Dataset]. http://scan-bugs.org/portal/collections/index.php [Accessed 08 March 2019]

Cuda, J.P. and J.H. Frank. 2012. Florida’s established arthropod weed biological control agents and their target. University of Florida, Entomology & Nematology Department, UF/IFAS Extension document ENY-853 (IN779).

Cullenward, M.J., P.R. Ehrlich, R.R. White and C.E. Holdren. 1979. The ecology and population genetics of an Alpine checkerspot butterfly, *Euphydryas anicia*. Oecologia 38(1): 1-12.

Cunningham, K.J. 2011. A new Louisiana state record *Problema byssus byssus* (W. H. Edwards). Southern Lepidopterists’ News 33(4): 149.

Cuthrell, D.L. 1999a. Special animal abstract for *Papaipema sciata* (culver’s root borer). Michigan Natural Features Inventory, Lansing, Michigan. 3 pp.

Cuthrell, D.L. 1999b. Special animal abstract for *Papaipema beeriana* (blazing star borer). Michigan Natural Features Inventory, Lansing, Michigan. 3 pp.

Czerwinski, E.J. and W.D. Biggs. 1991. Results of Forest Insect and Disease Surveys in the Southwestern Region Of Ontario 1990. Forestry Canada, Ontario Region, Great Lakes Forestry Centre. Misceilanous Report No. 105.

DaCosta, M.A., P. Larson, J.P. Donahue, and S.J. Weller. 2006. Phylogeny of milkweed tussocks (Arctiidae: Arctiinae: Phaegopterini) and its implications for evolution of ultrasound communication. Annals of the Entomological Society of America 99(5): 723-742.

Damaiyani, J. and D.A. Lestari. 2011. Inventarisasi hama dan penyakit pada koleksi Paku-Pakuan Kebun Raya Purwodadi. Berkala Penelitian Hayati Edisi Khusus7A: 173-177.

Dana, R.P., C. Lane, and D. Hansen. 2005. New larval host plant for *Lycaeides melissa melissa* in Wisconsin and Minnesota and potential threat to *Lycaeides melissa samuelis* (Lycaenidae). Journal of the Lepidopterists' Society 59: 175-177.

Danderson, C.A. 2006. Notes on aspects of the life history and behavior of *Coleotechnites eryngiella* (Gelechidae). Journal of the Lepidopterists' Society 60(2): 103-106.

Dang, P.T. 1984. A new species of *Cochylis* Treitschke (Lepidoptera: Cochylidae) from Saskatchewan. The Canadian Entomologist 116(2): 253-256.

Dang, P.T. 1985. Key to adult males of conifer-feeding species of *Choristoneura* Lederer (Lepidoptera: Tortricidae) in Canada and Alaska. The Canadian Entomologist 117(01): 1-5.

Dang, P.T. 1992a. Holarctic distribution of *Choristoneura albaniana* (Walker), with new synonymy (Tortricidae). Journal of the Lepidopterists' Society 46(4): 269-272.

Dang, P.T. 1992b. Morphological study of male genitalia with phylogenetic inference of *Choristoneura* Lederer (Lepidoptera: Tortricidae). The Canadian Entomologist 124(1): 7-48.

Dang, P.T. 1993. First North American records of *Epinotia abbreviana* (Tortricidae), a European pest of *Ulmus* species. Journal of the Lepidopterists' Society 47(1): 55-59.

Dang, P.T. and D.J. Parker. 1990. First records of *Enarmonia formosana* (Scopoli) in North America (Lepidoptera: Tortricidae). Journal of the Entomological Society of British Columbia 87: 3-6.

Dang, P.T., R.W. Duncan and S. Fitzpatrick, 1996. Occurrence of two palaearctic species of *Clepsis* Guenee, *C. spectana* Treitschke and *C. consimilana* (Hübner) (Torticidae) in British Columbia. Journal of the Lepidopterists’ Society 50(4): 321-328.

Daniels, J.C., M.C. Minno, T.C. Emmel, P.J. Eliazar, and L.L. Groce. 1993. Egg color dimorphism in the endangered Schaus Swallowtail butterfly, *Papilio aristodemus ponceanus*, of south Florida (Lepidoptera: Papilionidae). Tropical Lepidoptera Research 4: 65-66.

Daniels, J.C., J.A. Pence, and T.C. Emmel, 2005, New hostplant record for *Strymon martialis* (Lycaenidae: Theclinae) in the Florida Keys. Journal of the Lepidopterists' Society 59 (3): 174-175.

Danilevsky, A.A. and V.I. Kuznetsov. 1968. Tortricidae: Tribe Laspeyresiini, Fauna USSR (Insecta-Lepidoptera) 5(1): 1-635. Moscow Leningrad.

Danks, H.V. 1980. Arthropods of Polar Bear Pass, Bathurst Island, Arctic Canada. Syllogeus 25: 1-68.

Danks, H.V. 1992. Arctic insects as indicators of environmental change. Arctic 45(2): 159-166.

Dantas, J., I.O. Motta, L.A. Vidal, E.F.M.B. Nascimento, J. Bilio, J.M. Pupe, A.Veiga, C. Carvalho, R.B. Lopes, T.L. Rocha, L.P. Silva, J.R. Pujol-Luz JR, and É.V.S. Albuquerque, 2021. A comprehensive review of the Coffee Leaf Miner Leucoptera coffeella (Lepidoptera: Lyonetiidae) - A major pest for the coffee crop in Brazil and others Neotropical countries. Insects. 12(12): 1130.

Darbyshire, S.J., A. Francis, A. DiTommaso, and D.R. Clements. 2012. The Biology of Canadian weeds. 150 *Erechtites hieraciifolius* (L.) Raf. ex DC. Canadian Journal of Plant Science 92(4): 729-746.

Darlington, E.P. 1945a. Notes on some Microlepidoptera. Entomological News 56(3): 70-74.

Darlington, E.P. 1945b. Notes on some Microlepidoptera. Entomological News 56(4): 89-93.

Darlington, E.P. 1949. Notes on some North American Lepidoptera reared on sweet fern (*Comptonia asplenifolia* Linnaeus) with description of new species. Transactions of the American Entomological Society 74(3-4): 173-185.

Darlington, E.P. 1952. Notes on blueberry Lepidoptera in New Jersey. Transactions of the American Entomology Society 78: 33-57.

Da Silva, G.C. 2008. Diversidade de Borboletas Nymphalidae na Mata Atlântica do Parque Municipal da Lagoa do Peri, Florianópolis, SC. Bacharel Thesis. Universidade Federal de Santa Catarina, Florianópolis, Santa Catarina, Brazil.

Daterman, G E., R.G. Robbins, T.D. Eichlin, and J. Pierce. 1977. Forest Lepidoptera attracted by known sex attractants of western spruce budworms, *Choristoneura* spp. (Lepidoptera: Tortricidae). The Canadian Entomologist 109(06): 875-878.

Dauphin, J., D. Dauphin, E. Knudson, and C. Bordelon. 2005. Three new butterflies new to the United States from south Texas. News of the Lepidopterists' Society 47(2): 43-46.

Davenport, K. 2001. 2000 Season Summary. Zone 3. Southwest, Hawaii Arizona, California, Nevada, and Hawaii. News of the Lepidopterists' Society 43(S1): 7-11.

Davenport, K. 2002. 2001 Season Summary. Zone 3. Southwest, Hawaii Arizona, California, Nevada, and Hawaii. News of the Lepidopterists' Society 44(S1): 8-14.

Davenport, K. 2003. 2002 Season Summary. Zone 3. Southwest, Hawaii Arizona, California, Nevada, and Hawaii. News of the Lepidopterists' Society 45(S1): 11-16.

Davenport, K. 2004a. 2005 Season Summary. Zone 3. Southwest, Hawaii Arizona, California, Nevada, and Hawaii. News of the Lepidopterists' Society 46(S1): 10-20.

Davenport, K. 2004b. The Yosemite butterflies - updated survey of this sector of The Sierra Nevada. The Taxonomic Report of the International Lepidoptera Survey 5(1): I,1-74.

Davenport, K. 2005. 2004 Season Summary. Zone 3. Southwest, Hawaii Arizona, California, Nevada, and Hawaii. News of the Lepidopterists' Society 47(S1): 6-16.

Davenport, K. 2006. 2005 Season Summary. Zone 3. Southwest, Hawaii Arizona, California, Nevada, and Hawaii. News of the Lepidopterists' Society 48(S1): 12-22.

Davenport, K. 2007a. 2006 Season Summary. Zone 3. Southwest, Hawaii Arizona, California, Nevada, and Hawaii. News of the Lepidopterists' Society 49(S1): 7-14.

Davenport, K. 2007b. The Yosemite butterflies (text): supplement of additional information, collections and corrections for volume 5, No. 1. The Taxonomic Report of the International Lepidoptera Survey 5(1 supplement): 1-9.

Davenport, K. 2008. 2007 Season Summary. Zone 3 Southwest/Hawaii, California, Arizona, Nevada and Hawaii. News of the Lepidopterists’ Society 50(S1): 6-15.

Davenport, K. 2009. 2008 Season Summary. Zone 3 Southwest/Hawaii, California, Arizona, Nevada and Hawaii. News of the Lepidopterists’ Society 51(S1): 9-17.

Davenport, K. 2010. 2009 Season Summary. Zone 3. Southwest, Hawaii Arizona, California, Nevada, and Hawaii. News of the Lepidopterists' Society 52(S1): 6-13.

Davenport, K. 2011. 2010 Season Summary. Zone 3. Southwest, Hawaii Arizona, California, Nevada, and Hawaii. News of the Lepidopterists' Society 53(S1): 18-29.

Davenport, K. 2012. 2011 Season Summary. Zone 3. Southwest, Hawaii Arizona, California, Nevada, and Hawaii. News of the Lepidopterists' Society 54(S1): 11-21.

Davenport, K. 2013. 2012 Season Summary. Zone 3. Southwest, Hawaii Arizona, California, Nevada, and Hawaii. News of the Lepidopterists' Society 55(S1): 8-16.

Davenport, K. 2014. 2011 Season Summary. Zone 3. Southwest, Hawaii Arizona, California, Nevada, and Hawaii. News of the Lepidopterists' Society 56(S1): 16-23.

Davenport, K. 2015. 2014 Season Summary. Zone 3. Southwest, Hawaii Arizona, California, Nevada, and Hawaii. News of the Lepidopterists' Society 57(S1): 18-28.

Davenport, K. 2017. 2016 Season Summary. Zone 3. Southwest/Hawaii: Arizona, California, Nevada, and Hawaii. News of the Lepidopterists' Society 57(S1): 33-42.

Davenport, K. 2018. Lepidoptera of North America 15, Butterflies of southern California in 2018: updating Emmel and Emmel’s 1973 Butterflies of southern California. Contributions of the C.P. Gillette Museum of Arthropod Diversity, Colorado State University, Fort Collins, Colorado.

Davenport, K. 2020. Lepidoptera of North America 16: Butterflies of the Sierra Nevada. Contributions of the C.P. Gillette Museum of Arthropod Diversity, Colorado State University, Fort Collins, Colorado.

Daviault, L. and R. Ducharme. 1966. Life history and habits of the green spruce leaf miner, *Epinotia nanana* (Treitschke) (Lepidoptera: Tortricidae). The Canadian Entomologist, 98(7): 693-699.

Davis, C.J. 1969. Notes on the grass webworm, H*erpetogramma licarsisalis* (Walker) (Lepidoptera: Pyraustidae), a new pest of turfgrass in Hawaii and its enemies. Proceedings of the Hawaiian Entomological Society 20(2): 311-316.

Davis, C.J., E. Yoshioka, and D. Kageler. 1992. Biological control of lantana, prickly pear, and Hamakua pamakani inhawah: a review and update. Pp. 411-431 in: C.P. Stone, C.W. Smith, and J.T. Tunison. (eds.). Alien Plant Invasions in Native Ecosystems of Hawai’i: Management and Research. University of Hawai’i Press, Honolulu, Hawai’i.

Davis, D.R. 1964. Bagworm moths of the Western Hemisphere. United States National Museum Bulletin 244: 1-233.

Davis, D.R. 1967. A Revision of the moths of the Subfamily Prodoxinae (Lepidoptera: Incurvariidae). United States National Museum Bulletin 255: 1-170.

Davis, D.R. 1969a. A revision of the American moths of the family Carposinidae (Lepidoptera: Carposinoidea). United States National Museum Bulletin 289: 1-105.

Davis, D.R. 1969b. A review of the genus *Acanthopteroctetes* with description of a new species (Eriocraniidae). Journal of the Lepidopterists’ Society 23: 137-47.

Davis, D.R. 1972. *Careospina quercivora*, a new genus and species of moth infesting live oaks in California (Lepidoptera: Incurvariidae). Proceedings of the Entomological Society of Washington 74(1): 121-128.

Davis, D.R. 1975. A Review of Ochsenheimeriidae and the introduction of the cereal stem moth, *Ochsenheimeria vacculella i*nto the United States (Lepidoptera: Tineodea). Smithsonian Contributions to Zoology 192: 1-20.

Davis, D.R. 1978a. A revision of the North American moths of the superfamily Eriocranioidea, with the proposal of a new family, Acanthopteroctetidae (Lepidoptera). Smithsonian Contributions to Zoology 251: 1-131.

Davis, D.R. 1978b. The North American moths of the genera *Phaeoses, Opogona*, and *Oinophila,* with a discussion of their supergeneric affinities. Smithsonian Contributions to Zoology 282: 1-39.

Davis, D.R. 1978c. New leaf-mining moths of the family Nepticulidae from Florida. 61(4): 209-244.

Davis, D.R. 1984. A new *Acanthopteroctetes* from the northwestern United States (Acanthopteroctetidae). Journal of the Lepidopterists' Society 38(3): 47-50.

Davis, D.R. 1986. Neotropical Tineidae, 1: The types of H.B. Moschleer (Lepidoptera: Tineoidea). Proceedings of the Entomological Society of Washington 88(1): 83-92.

Davis, D.R. 1989. Generic revision of the Opostegidae, with a synoptic catalog of the world's species (Lepidoptera: Nepticuloidea). Smithsonian Contributions to Zoology 251: 1-131.

Davis, D.R. 1990. Blastobasidae. In: Miller, S.E. and R.W. Hodges, (eds.). Primary types of Microlepidoptera in the Museum of Comparative Zoology (with a discussion of V.T. Chambers’ work). Bulletin of the Museum of Comparative Zoology 152(2): 45-87.

Davis, D.R. 1990b. Three new species of *Acrolophus* from the southeastern United States with remarks on the status of the family Acrolophidae (Lepidoptera: Tineoidea). Proceedings of the Entomological Society of Washington 92(4): 694-704.

Davis, D.R. 2019. *Anarsioses*, a new generic name for *Phyllonorycter aberrans* (Braun) (Lepidoptera: Gracillariidae). Zootaxa 4701 (6):574-580.

Davis, D.R. and M.M. Davis. 2017. First report of the genus *Telamoptilia* from the western hemisphere with descriptions of two new species (Gracillariidae). Journal of the Lepidopterists’ Society 71(4): 261-273.

Davis, D.R. and J. De Prins. 2011. Systematics and biology of the new genus *Macrosaccus* with descriptions of two new species (Lepidoptera, Gracillariidae). ZooKeys 98: 29-82.

De Prins J. and W. De Prins. 2011-2024. Global Taxonomic Database of Gracillariidae (Lepidoptera). World Wide Web electronic publication http://www.gracillariidae.net [28 March 2025].

Davis, D.R. and G. Deschka. 2001. Biology and Systematics of the North American *Phyllonorycter* leafminers on Salicaceae, with a Synoptic Catalog of Palearctic Species (Lepidoptera: Gracillariidae). Smithsonian Contributions to Zoology 614: 1-89.

Davis, D.R. and S.H. Faeth. 1986. A new oak-mining eriocraniid moth from southeastern United States (Lepidoptera: Eriocraniidae). Proceedings of the Entomological Society of Washington 88(1): 145-153.

Davis, D.R., and G.R. Graves. 2016. A new leafmining moth (*Cameraria cotinivora*, Lepidoptera: Gracillariidae) of the American Smoketree (*Cotinus obovatus*). Proceedings of the Entomological Society of Washington 118(2): 244-253.

Davis, D.R. and J.-F. Landry. 2012. A review of the North American genus *Epimartyria* (Lepidoptera, Micropterigidae) with a discussion of the larval plastron. ZooKeys 183: 37-83. doi: 10.3897/zookeys.183.2556

Davis, D.R. and M.J. Medeiros. 2023. A revision of the family Adelidae of the Western Hemisphere (Lepidoptera: Adeloidea). Smithsonian Contributions to Zoology 656: 1-215.

Davis, D.R. and H. Mendel. 2013. The genus *Erechthias* Meyrick of Ascension Island, including discovery of a new brachypterous species (Lepidoptera, Tineidae). ZooKeys 341: 1-20.

Davis, D.R. and S.E. Miller, 1984. Chapter 20. Gracillariidae, pp. 25-27 in J.B. Heppner (ed.). Atlas of Neotropical Lepidoptera, checklist part I Micropterigoidea - Immoidea. Dr, W. Junk Publishers, The Hague.

Davis, D.R. and E.G. Milstrey. 1988. Description and biology of *Acrolophus pholeter*, (Lepidoptera: Tineidae), a new moth commensal from gopher tortoise burrows in Florida. Proceedings of the Entomological Society of Washington 90: 164-178.

Davis, D.R. and J.E. Peña. 1990. Biology and morphology of the banana moth, *Opogona sacchari* (Bojer), and its introduction into Florida (Lepidoptera: Tineidae). Proceedings of the Entomological Society of Washington 92(4): 593-618.

Davis, D. R. and G.S. Robinson. 2013. The Tineoidea and Gracillarioidea. pp. 91-118 In: N.P. Kristensen (ed.). Handbook of Zoology, Volume 1: Evolution, Systematics, and Biogeography. de Gruyter, Berlin.

Davis, D.R. and J.R. Stonis. 2007. A revision of the New World plant-mining moths of the family Opostegidae (Lepidoptera: Nepticuloidea). Smithsonianibutions to Zoology 625: i-v, 1-212.

Davis, D.R. and D.L. Wagner. 2005. Biology and systematics of the Neotropical leafminer genus *Eucosmophora* (Lepidoptera: Gracillariidae). Biología y sistemática del género neotropical de minadores de hojas Eucosmophora (Lepidoptera: Gracillariidae). Tropical Lepidoptera 13(1-2): 1-40.

Davis, D.R. and D.L. Wagner. 2011. Biology and systematics of the New World *Phyllocnistis* Zeller leafminers of the avocado genus *Persea* (Lepidoptera, Gracillariidae). ZooKeys 97: 39-73. doi: 10.3897/zookeys.97.753

Davis, D.R., R.C. Kassulke, K.L.S. Harley, and J.D. Gillett. 1991. Systematics, morphology, biology, and host specificity of *Neurostrota gunniella* (Busck) (Lepidoptera: Gracillariidae), an agent for the biological control of *Mimosa pigra* L. Proceedings of the Entomological Society of Washington 93(1): 16-44.

Davis, D.R., O. Pellmyr and J.N. Thompson, 1992. Biology and systematics of *Greya* Busck and *Tetragma*, a new genus (Lepidoptera: Proxidae). Smithsonians Contributions to Zoology 524: 1-88.

Davis, D.R., F. McKay, M. Oleiro, M. Diniz Vitorino, and G.S. Wheeler. 2011. Biology and systematics of the leafmining Gracillariidae of Brazilian pepper tree, *Schinus terebinthifolus* Raddi, with descriptions of a new genus and four new species. Journal of the Lepidopterists' Society 65(2): 61-93.

Davis, D.R., M.M. Davis, and C. Mannion. 2012. Neotropical Tineidae IX: a review of the West Indian *Xystrologa* and biology of *Xystrologa* *grenadella* (Wlsm.), an invasive pest of cultivated greenhouse plants in Southern Florida, USA and Germany (Lepidoptera: Tineoidea). Proceedings of the Entomological Society of Washington 114(4): 439-455.

Davis, D.R., M.S. Fox, R.F. Hazen. 2013a. Systematics and biology of *Caloptilia triadicae* (Lepidoptera: Gracillariidae), a new speces of leaf-mining moth of the invasive Chinese tallow tree (T*riadica sebifera* (L.) Euphorbiaceae). Journal of the Lepidopterists' Society 67(4): 281-290.

Davis, D. R., R. Diaz, and W.A. Overholt. 2013b. Systematics and biology of *Cremastobombycia chromolaenae*, new species (Gracillariidae), a natural enemy of *Chromolaena odorata* (L.) King and H. Robinson (Asteraceae). The Journal of the Lepidopterists' Society 67(1) 35-41.

Davis, E.G., J.R. Horton, C.H. Gable, E.V. Walter, and R.A. Blanchard. 1933. The southwestern corn borer. United States Department of Agriculture Technical Bulletin Number 388.

Davis, H.G., L.M. McDonough, P.S. Chapman, C.L. Smithhisler, D.D. Bowers, and S. Voerman. 1993. Sugar beet crown borer, *Hulstia undulatella* (Clemens) 1: Identification and field tests of female sex pheromone gland components. Journal of Chemical Ecology 19(3): 433-440.

Davis, K., M. Stangeland and A.D. Warren. 2005. *Mylon pelopidas*: A new record for Arizona and the United States with notes on its history, distribution, biology and identification. News of the Lepidopterists' Society 47(4): 103-104, 106, 110-111.

Davis, S. and D. Davis. 2009. First report of the Old World genus *Pelecystola* in North America, with description of a new species (Lepidoptera, Tineidae). ZooKeys 25: 69-78.

Day, K.R., S.R. Leather and R. Lines. 1991. Damage by *Zeiraphera diniana* (Lepidoptera: Tortricidae) to lodgepole pine (*Pinus contorta*) of various provenances. Forest Ecology and Management 44(2-4): 133-145.

Dean, C.-A.E., A. Katz, W.-Y. Wu, and M.R. Berenbaum. 2022. Degree of dietary specialization on furanocoumarin-containing hostplants in a newly invasive species, *Depressaria depressana* (Lepidoptera: Depressariidae). Journal of Chemical Ecology 48: 850-866.

de Armas, L.F and R. Nunez. 2005. Malpighiaceae: Nueva familia de plantas hospederas de *Platynota rostrana* Walcker, 1863 (Lepidoptera: Tortricidae) [Malpighiaceae: a new family of host plant for *Platynota rostrana* Walker, 1863 (Lepidoptera: Tortricidae)]. Boletín Sociedad Entomológica Aragonesa 36: 354.

Dearness, J. 1927. Mycophagic note on *Chytonix palliatricula*. Mycologia 19(6): 322.

de Benedictis, J.A. 1984. On the taxonomic position of *Ellabella* Busck, with description of the larva and pupa of *E. bayensis* (Lepidoptera: Copromorphicae). Journal of Research on Lepidoptera 23(1): 74-82.

de Benedictis, J.A. 1985. The pupa of *Lotisma trigonan*a and some characteristics of the Copromorphidae (Lepidoptera). Journal of Research on Lepidoptera 24(2): 132-135.

de Benedictis, J.A., D.L. Wagner, and J.B. Whitfield. 1990. Larval hosts of the Microlepidoptera of the San Bruno Mountains, California. Atala 16: 14-35.

Decker, G.C. 1930. The Biology of the four-lined borer, *Luperina stipata* (Morr.). Iowa Agricultural Experiment Station Research Bulletin 125: 127-164.

Decker, G.C. 1931. The biology of the stalk borer *Papaipema nebris* (Gn.). Iowa Agricultural Experiment Station Research Bulletin 43: 329-351.

Deedat, Y. A., C.R. Ellis and R.J. West. 1983. Life history of the potato stem borer (Lepidoptera: Noctuidae) in Ontario, Canada. Journal of Economic Entomology 76: 1033-1037.

Defoliart, G.R. 1956. An annotated list of southeastern Wyoming Rhopalocera. The Lepidopteristi' News 10(3-4): 91-101.

de Freina, J.J. 1997. Die Bombyces und Sphinges der Westpalearktis. Band 4, Sesiidae. Edition Forschung & Wissenschaft Verlag GmbH, München, Germany.

de Freina, J.J. and T.J. Witt. 1987. Die Bombyces und Sphinges der Westpalaearktis (Insecta, Lepidoptera). Band 1. München: Forschung und Wissenschaft.

Demetriou, J., M. Aristophanous, E. Koutsoukos, E. John, H.E. Roy, A.F. Martinou, and J. Rota. 2025. A cosmopolitan invader - *Choreutis sexfasciella* (Lepidoptera, Choreutidae)-in Cyprus: first record, molecular characterization, and a reared parasitoid. Nota Lepidopterologica 48: 15-28.

De Nova, J.A. V. Sosa and V.W. Steinmann. 2007. A synopsis of *Adelia* (Euphorbiaceae s.s.). Systematic Botany 32(3): 583-595.

De Prins, J. and W. De Prins, 2006. Global Taxonomic Database of Gracillariidae (Lepidoptera). World Wide Web electronic publication (http: //gc.bebif.be) [accessed 23 March 2007].

De Prins, J. and W. De Prins. 2011-2025. Global Taxonomic Database of Gracillariidae (Lepidoptera). World Wide Web electronic publication http://www.gracillariidae.net [accessed 04 Feb 2025].

De Prins, J. and W. De Prins. 2014a. Global Taxonomic Database of Gracillariidae (Lepidoptera). World Wide Web electronic publication (http://www.gracillariidae.net) [accessed 7 August 2014, June 2015]

De Prins, W. and J. De Prins. 2014b. *Metalampra italica* (Lepidoptera: Oecophoridae), also in Belgium. Phegea 42(2): 28; 1-3.

De Prins, J., H.A. Arévalo-Maldonado, D.R. Davis, B. Landry, H.A. Vargas, M.M. Davis, R. Brito, J. Fochezato, I. Ohshima, and G.R.P. Moreira. 2019. An illustrated catalogue of the Neotropical Gracillariidae (Lepidoptera) with new data on primary types. Zootaxa 4575(1): 1-110.

Deschka, G. 1994. *Bucculatrix frigida* sp. nov. aus der borealen Nearktis (Lepidoptera, Lyonetüdae). Entomofauna Zeitschrift für Entomologie 13(33): 545-556.

De Silva, E.C.A., P.J. Silk, P. Mayo, N. K. Hillier, D. Magee, and G.C. Cutler. 2013 Identification of sex pheromone components of blueberry spanworm *Itame argillacearia* (Lepidoptera: Geometridae). Journal of Chemical Ecology 39(9): 1169-1181.

de Souza, B.R.l, W.T. Tay, C. Czepak, S. Elfekih, and T.K. Walsh. 2016. The complete mitochondrial DNA genome of a *Chloridea (Heliothis) subflexa* (Lepidoptera: Noctuidae) morpho-species. Mitochondrial DNA Part A 27(6): 4532-4533.

Dethier, V.G. 1941. Metamorphoses of Cuban Nymphalidae and Lycaenidae. Psyche 48(2-3): 70-78.

Devries, P.J. 1985. Hostplant records and natural history notes on Costa Rica butterflies (Papilionidae, Nymphalidae & Pieridae). Journal of Research on the Lepidoptera 24(4): 290-333.

Devries, P.J. 1987. The Butterflies of Costa Rica and Their Natural History, Volume I: Papilionidae, Pieridae, Nymphalidae. Princeton University Press, New Jersey. 327 pp.

DeWaard, J.R., B.C. Schmidt, G.G. Anweiler, and L.M. Humble. 2008. First Canadian records of *Lampropteryx suffumata* ([Denis & Schiffermiiller], 1775) (Geometridae: Larentiinae). Journal of the Entomological Society of British Columbia 105: 19-25.

DeWaard, J.R., J.-F. Landry, B.C. Schmidt, J. Derhousoff, J.A. McLean, L.M. Humble. 2009. In the dark in a large urban park: DNA barcodes illuminate cryptic and introduced moth species. Biodiversity and Conservation 18(14): 3825-3839.

DeWaard, J.R., L.M. Humble, and B.C. Schmidt. 2010. DNA barcoding identiﬁes the ﬁrst North American records of the Eurasian moth, *Eupithecia pusillata* (Lepidoptera: Geometridae). Journal of the Entomological Society of British Columbia, 107, 25-31.

Dewaard, J.R., P.D.N. Hebert, and L.M. Humble. 2011. A Comprehensive DNA Barcode Library for the Looper Moths (Lepidoptera: Geometridae) of British Columbia, Canada. PLoS ONE 6(3): e18290. doi:10.1371/journal.pone.0018290

Dewey, J E. 1972. A Pine Looper, *Phaeoura mexicanaria* (Lepidoptera: Geometridae), in southeastern Montana with notes on its biology. Annals of the Entomological Society of America 65(2): 306-309.

Deyrup, M., J. Kraus, and T. Eisner. 2004. A Florida caterpillar and other arthropods inhabiting the webs of a subsocial spider (Lepidoptera: Pyralidae; Araneida: Theridiidae). Florida Entomologist 87(4): 554-558.

Deyrup, M., N.D. Deyrup, M. Eisner, and T. Eisner. 2005. A caterpillar that eats tortoise shells. American Entomologist 51(4): 245-248.

Dhakal, K., R. Bika, B. Ghimire, M. Parajuli, S. Neupane, K. Neupane, K.M. Addesso, and F.Baysal-Gurel. 2022. Arthropod and disease management in boxwood production. Journal of Integrated Pest Management 13(1): 18; 1-10.

Diakonoff, A. 1939. Notes on Microlepidoptera II. Remarks on some species of the genus *Blastodacna* Wocke (Fam. Cosmopterygidae). Tijdschrift voor Entomologie 82: 64-77.

Diakonoff, A. 1956a. Records and descriptions of Microlepidoptera (8). Zoologische Verhandelingen 29(1): 1-60.

Diakonoff, A. 1964. Further records and descriptions of the species of *Bactra* Stephens (Lepidoptera, Tortricidae). Zoologische Verhandelingen 70: 3-81 + 1 plate.

Diakonoff, A. 1967. Microleptera of the Philippine Islands. United States Museum Bulletin 257: 1-484.

Diakonoff, A. 1968. Microlepidoptera of the Philippine Islands. Bulletin of the United States National Museum 257: 1-484.

Diakonoff, A. 1973. The South Asiactic Olethreutini (Lepidoptera: Tortricidae). Zoölogische Monographieën van het Rijksmuseum van Natuurlijke Historie, 1. E.J. Brill, Leiden.

Diakonoff, A. 1982. On a collection of some families of Mircolepidoptera from Sri Lanka (Ceylon). Zooloigische Verhandelingen 193: 1-124.

Diakonoff, A. 1986. Microlepidoptera Palaearctica 7: Glyphiterigidae (sensu Meyrick) containing: In: H.G. Amsel, H. Reisser, and F. Gregor, (eds.). Microlepidoptera Palaearctica. Verlag Georg Fromme & Co., Wien, Germany.

Diakonoff, A. 1989. Revision of the Palaearctic Carposinidae with description of a new genus and new species (Lepidoptera: Pyraloidea). Zoologische Verhandelingen 251: 1-155.

Dias, F.M.S., A. Specht, A., G. San Blas, and M.M. Casagrande. 2025. Immature stages of the Greater Cutworm, *Feltia repleta* (Walker, 1857) (Noctuidae: Noctuinae), with notes on its host plants and distribution. Neotrop Entomol 54: 62; 1-20.

Diatloff, G. and W.A. Palmer. 1988. The host specificity and biology of *Aristotelia ivae* Busck (Gelechiidae) and *Lorita baccharivora* Pogue (Tortricidae), two microlepidoptera selected as biological control agents for *Baccharis halimifolia* (Asteraceae) in Australia. Proceedings of the Entomological Society of Washington 90(4): 458-461.

Diaz, R., K. Hibbard, A. Samayoa and W. A. Overholt 2012. Arthropod community associated with tropical soda apple and natural enemies of *Gratiana bolivian*a (Coleoptera: Chrysomelidae) in Florida. Florida Entomologist 95(1): 228-232.

Diaz, R., S. Romero, A. Roda, C. Mannion and W.A. Overholt. 2015 Diversity of arthropods associated with *Mikania* spp. and *Chromolaena odorata* (Asterales: Asteraceae: Eupatorieae) in Florida. Florida Entomologist 98(1): 389-393.

Dickel, T.S. 1991. New records of Noctuid moths from Florida (Lepidoptera: Noctuidae). Tropical Lepidoptera 2: 53-58.

Dickel, T.S. 1992. New records of Microlepidoptera from Florida (Lepidoptera: Oecophoridae, Yponomeutidae) Tropical Lepidoptera 3(1): 55-56.

Dickel, T.S. and J.B. Heppner. 2020. Florida Lepidoptera Notes, 26. *Baphala* in Florida (Lepidoptera: Pyralidae: Phycitinae). Lepidoptera Novae 12: 57-62.

Dickel, T.S., V.A. Brou, Jr., and J.B. Heppner. 2010. New North American records of the Asian species, *Simplicia cornicalis*, in Florida and Louisiana (Lepidoptera: Noctuidae: Herminiinae). Lepidoptera Novae 3(1): 53-56.

Dickens, M. and E. Storey. 1974. The World of Moths. Osprey Publishing Limited, Reading, U.K.

Dickerson, W.A. and W.H. Kearby. 1972. The identification and distribution of the tip moths of the genus *Rhyacionia* (Lepidoptera: Olethreutidae) in Missouri. Journal of the Kansas Entomological Society 45(4): 542-551.

Dickson, C.G.C. and D.M. Kroon, (eds.). 1978. Pennington's Butterflies of Southern Africa. Ad Donker Publishers, Johannesburg, South Africa.

Dietz, W.G. 1900. On *Pigritia* Clem. Transactions of the American Entomological Society 27: 100- 120.

Dietz, W.G. 1905. Revision of the genera and species of the tineid subfamilies Amydriinae and Tineinae inhabiting North America. Transactions of the American Entomological Society 31(1): 1-96.

Dietz, W.G. 1907. The North American species of the genus *Ornix* Tr. Transactions of the American Entomological Society 33: 287-297.

Dietz, W.G. 1910. Revision of the Blastobasidæ of North America. Transactions of the American Entomological Society 36(1): 1-72.

Dietz, R.E. IV and W. Duckworth. 1976. A review of the genus *Horama* Hubner and reestablishment of the genus *Poliopastea* Hampson (Lepidoptera: Ctenuchidae). Smithsonian Contributions to Zoology 215: 1-53.

Diez-Rodríguez, G.I., L.K. Hübner, L.E.C. Antunes, and D.E. Nava. 2013. *Herpetogramma bipunctalis* (Lepidoptera: Crambidae) biology and techniques for rearing on leaves of the blackberry (*Rubus* spp., Rosaceae). Brazilian Journal of Biology 73(1): 179-184.

Diniz, I.R. and H.C. Morais. 1995. Larvas de Lepidoptera e sus plantas hospedrias en un cerrado de Brasilia, D. F., Brazil. Revista Brasileiro de Entomologia 39: 755-770.

Diniz, I.R., H.C. Marais, and A.J.A. Camargo. 2001. Host plants of lepidopteran caterpillars in the cerrado of the Distrito Federal, Brasil. Revista Brasileiro de Entomologia 45: 107-122.

Diodato, M.A. and J.H. Pedrosa-Macedo. 1996. Presencia de *Condylorrhiza vestigialis* (Guenée, 1854) (Lepidóptera, Crambidae) sobre *Populus* spp. en el Brasil. Quebracho (4): 17-19.

Dirimanov, M. and G. Sengalevich. 1962. [Pests on the French grape.] Izvestiia na Instituta po lozarstvo i vinarstvo. Pleven 3: 34-38.

Diškus, A. and J.R. Stonis. 2015. *Astrotischeria neotropicana* sp. nov.-a leaf-miner on *Sida*, Malvaceae, currently with the broadest distribution range in the Neotropics (Lepidoptera, Tischeriidae). Zootaxa 4039(3): 456-466.

Disque, H. 1908. Versuch einer microlepidopterologischen Botanik. Deutsch Entomologische Zeitschrift Iris 21: 34-147.

Dix, M.E. and M. Jacobson. 1986. Trap preferences of *Retinia metallica* and seasonal flight behavior of *Retinia* spp., *Rhyacionia* spp. (Tortricidae), and Chionodes spp. (Gelechiidae) in the Dakotas. Journal of the Lepidopterists' Society 40(4): 1986-298.

Dixon, P.L. and K. Carl. 2002. Chaper 17. *Croesia curvalana* (Kearfott), Blueberry Leaftier (Lepidoptera: Tortricidae). Pp. 87-89 In: P.G. Mason and J.T. Huber, (eds.). Biological Control Programmes in Canada, 1981-2000. CAB International, Wallingford, United Kingdom.

DMNS:Ento. 2015. Denver Museum of Nature & Science - Entomology, Zoology Department, Denver, Colorado. In: SCAN. P.L. Heinrich, E. Gilbert, N.S. Cobb, and N. Franz. Symbiota collections of arthropods network (SCAN): A data portal built to visualize, manipulate, and export species occurrences. [Dataset]. http://scan-bugs.org/portal/collections/index.php [Accessed 08 March 2019]

Doane, R.W., E.C. Van Dyke, W.J. Chamberlin and H.E. Burke. 1936. Forest Insects. A Textbook for the use of Students in Forest Schools, Colleges and Universities, and for Forest Workers, 1^st^ Edition. American Forest Series. McGraw Hill Book Company, New York and London.

Dobrynina, V., J.R. Stonis, A. Diškus, M.A. Solis, S.V. Baryshnikova, and Y.-M. Shin. 2022. Global Nepticulidae, Opostegidae, and Tischeriidae (Lepidoptera): temporal dynamics of species descriptions and their authors. Zootaxa. 5099(4): 450-474.

Dod, F.H.W. 1913a. Further notes on Alberta Lepidoptera, with description of a new species. The Canadian Entomologist 60(3): 61-68.

Dod, F.H.W. 1913b. Further notes on Alberta Lepidoptera, with description of a new species. The Canadian Entomologist 60(2): 29-34.

Dodd, L.E., M.J. Lacki, and L.K. Rieske. 2011. Habitat Associations of Lepidoptera in the Ozark Mountains of Arkansas. Journal of the Kansas Entomological Society 84(4): 271-284.

Dodd, L.E., Z. Cornett1, A. Smith, and L.K. Rieske, 2013. Variation in Lepidopteran occurrence in hemlock-dominated and deciduous-dominated forests of central Appalachia. The Great Lakes Entomologist 46(1-2): 1-12.

Doǧanlar, M. 1979a. A new species of *Swammerdamia* Hbn. (Lepidoptera: Yponomeutidae) from British Columbia. The Canadian Entomologist 11(8): 967-969.

Doǧanlar, M. 1979b. *Pardia cynosbatella* (Lepidoptera: Tortricidae), an European leafroller new to North America. The Canadian Entomologist 11(8): 969.

Doǧanlar, M. 1979c. Pandemis heparana (Lepidoptera: Tortricidae), a Tortricid new to North America, eastablished in British Columbia. The Canadian Entomologist 11(8): 970.

Doǧanlar, M. and B.P. Beirne. 1978. *Croesia holmiana* (Lepidoptera: Tortricidae), a fruit tree leafroller new to North America, established in British Columbia. The Canadian Entomologist 110(6): 639-640.

Doǧanlar, M. and B.P. Beirne. 1981. The apple-and-thorn-skeletonizer; *Eutromula pariana*; Lepidoptera; Choreutidae; parasites. Journal of the Entomological Society of British Columbia 78: 51-52.

Dognin, P. 1905. Papillons nouveaux de l'Amerique du sud. Le Naturaliste 2(19/445): 215.

Dole, J.M., W.B. Gerard, and J.M. Nelson. 2004. Butterflies of Oklahoma, Kansas, and North Texas. University of Oklahoma Press, Norman, Oklahoma.

Dombroskie, J.J. 2003. The metalmark Moths (Lepidoptera: Choreutidae) of Ontario. Pp. 59-75 In: C.D. Jones and J.P. Crolla. 2003. Ontario Lepidoptera 2002. Toronto Entomologists' Association, Toronto, Ontario.

Dombroskie, J.J. 2011a. Aspects of archipine evolution (Lepidoptera: Tortricidae). Doctor of Philosophy Dissertation, University of Alberta, Edmonton, Alberta.

Dombroskie, J.J. 2011b. A matrix key to families, subfamilies, and tribes of Lepidoptera of Canada. Canadian Journal of Arthropod Identification 17: 1-129.

Dombroskie, J.J. and J.W. Brown. 2009. A new species of *Clepsis* Guenee, 1845 (Lepidoptera: Tortricidae) from the Sky Islands of southeastern Arizona. Proceedings of the Entomological Society of Washington 111(4): 769-774.

Dombroskie, J.J. and F.A. Sperling. 2012. Phylogeny of Nearctic *Pandemis (*Lepidoptera: Tortricidae), with focus on species boundaries in the *P. limitata* group. Annals of the Entomological Society of America 105(6): 768-780.

Dombroskie, J.J. and F.A. Sperling. 2013. Phylogeny of the tribe Archipini (Lepidoptera: Tortricidae: Tortricinae) and evolutionary correlates of novel secondary sexual structures. Zootaxa 3729(1): 1-62.

Dominick, C.B. 1964. Notes on the ecology and biology of the corn root webworm. Journal of Economic Entomology 57(1): 41-42.

Dominick, R.B. 1973 Life history of *Isoparce cupressi* (Sphingidae). Journal of the Lepidopterists' Society 27(1): 1-8.

Donahue, J.P. 1967. New records of Acrolophidae (Lepidoptera) from Kentucky. The Michigan Entomologist 1(4): 130-131.

Donahue, J.P. 1979. Strategies for survival. The cause of a caterpillar. Terra 27: 3-9.

Donahue, J.P. 1980. Resurrection of the genus *Morpheis* (Cossidae), with description of a new species in the Cognatus group from southern Arizona. Journal of the Lepidopterists' Society 34(2): 173-181.

Donahue, J.P. 1993a. New distribution records of the tiger moth genus *Phragmatobia* in North America (Lepidoptera: Arctiidae: Arcliinae). The Great Lakes Entomologist 26(1): 21-30.

Donahue, J.P. 1993b. Six species of tiger moths (Arctiidae: Lithosiinae, Ctenuchinae) new to the United States fauna, with notes on their nomenclature and distribution in Middle America. Journal of the Lepidopterists' Society 47(3): 199-210.

Donahue, J.P. 1993c. The distribution of three broadly sympatric species of *Symmerista* moths (Lepidoptera: Notodontidae) in the Great Lakes and Midwest regions of the United States. Great Lakes Entomologist 26(3): 161-171.

Donahue, J.P. and H.H. Neunzig. 2003. Two new species of *Dioryctria* from California (Lepidoptera: Pyralidae: Phycitinae). Holarctic Lepidoptera 8: 33-36.

Donahue, J.P. and J.H. Newman. 1966. The genus *Phragmatobia* in North America, with the description of a new species (Lepidoptera: Arctiidae). The Great Lakes Entomologist 1(2): 35-39.

Dornfeld, E.J. 1980. The butterflies of Oregon. Timber Press, Forest Grove, Oregon.

Doucette, C.F. 1941. *Eucordylea huntella* Keifer as a pest of *Rhododendron.* Journal of Economic Entomology 34(4): 588-589.

Dowell, R.V., R.J. Gill, D.R. Jeske, and M.S. Hoddle. 2016. Exotic terrestrial macro-invertebrate invaders in California from 1700 to 2015: an analysis of records. Proceedings of the California Academy of Sciences 63: 63-157.

Downer, R. and T. Ebert. 2014. Macrolepidoptera biodiversity in Wooster, Ohio from 2001 through 2009. ZooKeys 452: 79-105.

Downes, J.A. 1966. The Lepidoptera of Greenland; some geographic considerations. The Canadian Entomologist 98(11): 1135-1144.

Downey, J.C. and A.C. Allyn, 1979. Morphology and biology of the immature stages of *Leptotes cassius theonus* (Lucas) (Lepidoptera: Lycaenidae). Bulletin of the Allyn Museum, 55: 1-27.

Doyle, J.F. III. 1979. Temporary range extension and larval foodplant of *Dynamine dyonis* (Nymphalidae) in Texas. Journal of the Lepidopterist’s Society 33(1): 20.

Doyle, J.F. and T. Collins 2008. *Gorgythion begga pyralina* (Moschler, 1877): now comes an uncommon visitor from the south. Southern Entomologist’s News 30(1): 23-24.

Dozier, H.L. 1920. An ecological study of hammock and piney woods insects in Florida. Annals of the Entomological Society of America 13(4): 325-380.

Draudt, M.W.K. 1921-24. B. Grypocera, breitköpfige Tagfalter. In: Seitz, A. (Ed.). Die Gross-Schmetterlinge der Erde. Stuttgart, Alfred Kernen. 5: 836-1011.

Drew, W.A. 1962. Oklahoma Arctiidae (Lepidoptera). Proceedings of the Oklahoma Academy of Sciences 42: 93-100.

Drew, W.A. and D.C. Arnold. 1983. The Lasiocampidae of Oklahoma (Lepidoptera). Proceedings of the Oklahoma Academy of Sciences 63: 68-69.

Drooz, A.T. (ed). 1985. Insects of Eastern Forests. U.S. Department of Agriculture, Forest Service. Washington, DC. Miscellaneous Publication 1426.

Drooz, A.T. and H.H. Neunzig. 1988. Notes on the biology of two phycitines (Lepidoptera: pyrolidae) associated with Toumeyella pini (Homoptera: coccidae) on pine. Proceedings of the Entomological Society of Washington 90: 44-46.

Druce, H. 1881-1900. Biologia Centrali-Americana. Insecta. Lepidoptera-Heterocera. Vol. I. 490 pp.

Druce, H. 1889-1900. Biologia Centrali-Americana. Insecta. Lepidoptera-Heterocera. Vol. I.

Druce, H. 1887-1901. Biologia Centrali-Americana. Insecta. Lepidoptera-Heterocera. Vol. II. 622 pp.

Druce, H. 1889a. Descriptions of some new species Lepidoptera, chiefly from Central America. The Annals and Magazine of Natural History; Zoology, Botany, and Geology, Series 4(6): 77-94.

Druce, H. 1898. Descriptions of some new species *Syntomide,* chiefly in Oxford Museum. The Annals and Magazine of Natural History; Zoology, Botany, and Geology, Series 7 1(5): 401-408.

Drummond, B.A. III and K.S. Brown Jr. 1987. Ithomiinae (Lepidoptera: Nymphalidae): Summary of Known Larval Food Plants. Annals of the Missouri Botanical Garden 74(2): 341-358

Dubatolov, V.V. 2015. Taxonomic review of *Manulea* subgenus *Setema* (Lepidoptera: Erebidae: Arctiinae: Lithosiini). Canadian Entomologist 147: 541-552.

Dubatolov, V.V. and K.W. Philip. 2013. Review of the northern Holarctic *Arctia caja* complex (Lepidoptera: Noctuidae: Arctiinae). Canadian Entomologist 145: 147-154.

Duckworth, D.R. and T.D. Eichlin. 1977a. Two new species of clearwing moths (Sesiidae) from eastern North America clarified by sex pheromones. Journal of the Lepidopterists’ Society 31(3): 191-196.

Duckworth, D.R. and T.D. Eichlin. 1977b. A classification of the Sesiidae of America north of Mexico (Lepidoptera: Sesioidea). Occasional Papers in Entomology 26: 1-55.

Duckworth, D.R. and T.D. Eichlin. 1978. The clearwing moths of California (Lepidoptera: Sesiidae). Occasional Papers in Entomology of the California Department of Food and Agriculture 27: 1-80, plus 8 plates & index.

Duckworth, D.R. and T.D. Eichlin. 1983a. The type-material of North American Clearwing Moths (Sesiidae). Smithsonian Contributions to Zoology 148: 1-34.

Duckworth, D.R. and T.D. Eichlin. 1983b. Revision of the clearwing moth genus *Osminia* (Lepidoptera--Sesiidae). Smithsonian Contributions to Zoology 361.

Duckworth, W.D. 1964. North American Stenomidae (Lepidoptera: Gelechioidae). Proceedings of the United States National Museum 116(3495): 23-72.

Duckworth, W.D. 1965. North American moths of the genus *Swammerdamia* (Lepidoptera: Yponomeutidae). Proceedings of the United States National Museum 116(3507): 549-555.

Duckworth, W.D. 1970. Neotropical Microlepidoptera XVIII: Revision of the genus *Peleopoda* (Lepidoptera: Oecophoridae). Smithsonian Contributions to Zoology 48: 1-30.

Dugdale, J.S., D. Gleeson, L.H. Clunie, and P.W. Holder. 2005. A diagnostic guide to Tortricidae encountered in field surveys and quarantine inspections in New Zealand: morphological and molecular characters. Ministry of Agriculture and Forestry Biosecurity Authority, Wellington, New Zealand. 163 pp.

DUGWAY:DUG-ENT 2015. Dugway Proving Ground Natural History Collection, Dugway, UT. In: SCAN. P.L. Heinrich, E. Gilbert, N.S. Cobb, and N. Franz. Symbiota collections of arthropods network (SCAN): A data portal built to visualize, manipulate, and export species occurrences. [Dataset]. http://scan-bugs.org/portal/collections/index.ph

Dumont, C. 1930a. Note biologique sur *Pericyma acrosticta* Püng. (Lep. Catocalinae). Bulletin de la Société Entomologique de France 35: 13-16.

Duncan, C., J. Story, and R. Sheley. Revised by H. Parkinson and J. Mangold. 2011. Biology, Ecology and Management of Montana Knapweeds. U.S. Department of Agriculture (USDA), Montana State University and Montana State University Extension, Montana State University, Bozeman, Montana.

Dunford, J.C. and K.A. Barbara. 2005. Tetrio Sphinx, Giant Gray Sphinx, Frangipani Hornworm, *Pseudosphinx tetrio* (Linnaeus) (Insecta: Lepidoptera: Sphingidae). Reviewed 2011. Florida Cooperative Extension Service, Institute of Food and Agricultural Sciences, University of Florida EENY-344.

Dunn, K.L. and L.E. Dunn, 1991. Review of Australian Butterflies: Distribution, life history and taxonomy. Pushlished by authors, Melbourne, Australia.

Durden, L.A. and J.K. Adams 2011. Some new distributional records for *Eumorpha intermedia* (Sphingidae). Southern Lepidopterists’ News 33(4): 187-188.

Durden, L.A. and J.M. Anderson. 2008. Some records of "Florida" Lepidoptera from Georgia: Global Warming? Southern Entomologist’s News 30(2): 48-50.

Dussourd, D.E., M. Van Valkenburg, K. Rajan, and D.L. Wagner. 2019. A notodontid novelty: *Theroa zethus* caterpillars use behavior and anti-predator weaponry to disarm host plants. PLoS ONE 14(7): e0218994; 1-23.

Dyar, H.G. 1890. Preparatory stages of *Dilophonota edwardsii* Butler and *D. ello* Linnaeus. Entomologica Americana 6: 141-146.

Dyar, H.G. 1893. The larvae of the Clisiocampidae. Canadian Entomologist 25: 37-44.

Dyar H.G. 1895. A new *Datana*. Journal of the New York Entomological Society 3(4): 167.

Dyar, H.G. 1896a. A new *Gloveria.* Journal of the New York Entomological Society 4: 22-26.

Dyar, H.G. 1898. New American moths and synonymical notes. Journal of the New York Entomological Society 6: 33-44.

Dyar, H.G. 1899a. On the larvae of North American Nolidae, with description of new species. Canadian Entomolgist 31: 61-64.

Dyar, H.G. 1899b. Description of larvae of *Ingura delineata,* Guen. Canadian Entomologist 31: 27-28.

Dyar, H.G. 1899c. A new Lithosian. Psyche, 8: 359-360.

Dyar, H.G. 1900. Notes on the larval-cases of Lacosomidae (Perophoridae) and life-history of *Lacosoma chiridota* Grote. Journal of the New York Entomological Society 8: 177-180.

Dyar, H.G. 1901a. Notes on some winter Lepidoptera of Lake Worth, Florida. Proceedings of the Entomological Society of Washington 4: 446-485.

Dyar, H.G. 1901b. Life histories of some North American moths. Proceedings of the United States National Museum 23: 255-284.

Dyar, H.G. 1902a [1903]. A list of the North American Lepidoptera and key to the literature of this order of Insects. Bulletin of the United States National Museum 52. 723 pp.

Dyar, H.G. 1902b. Descriptions of the larvae of some moths from Colorado. Proceedings of the United States National Museum 25(1280): 369-412.

Dyar, H.G. 1903a. New North American Lepidoptera with notes on larvae. Proceedings of the Entomological Society of Washington. 5(4): 290-297.

Dyar, H.G. 1903b. List of Lepidoptera taken at William Arizonia by Messrs. Schwartz and Barber - I. Papilionoidea, Sphingoidea, Bombycoidea, Tineoidea (in part). Proceedings of the Entomological Society of Washington 5(3): 223-232.

Dyar, H.G. 1903c. A list of North American Lepidoptera and key to the literature of this order of insects. Bulletin of the United States National Museum 52: 1-723.

Dyar, H.G. 1904a. New species of North American Lepidoptera and a new Limacodid larva. Journal of the New York Entomological Society 12(1): 39-44.

Dyar, H.G. 1904b. Additions to the list of North American Lepidoptera, No. 1. Proceedings of the Entomolgical Society of Washington 6(2): 62-65.

Dyar, H.G. 1904c. The Lepidoptera of the Kootenai District of British Columbia. Proceedings of the United States National Museum 27(1376): 779-938.

Dyar, H.G. 1905a. North American Lepidoptera and synonymical notes. Proceedings of the Entomological Society of Washington 7: 29-39.

Dyar, H.G. 1905b. A list of North American Cochlidian moths, with descriptions of new gnera and species. Proceedings of the United States National Museum 29: 359-396.

Dyar, H.G. 1905c. New North American Lepidoptera and synonymical notes. Proceedings of the Entomological Society of Washington 7(1): 29-39.

Dyar, H.G. 1906. A few notes on the Streckler collection. Proceedings of the Entomological Society of Washington 7(2-3): 92-94.

Dyar, H.G. 1908a. A review of the North American Chrysauginae (Lepidoptera, Pyralidae). Proceedings of the Entomological Society of Washington 10: 92-102.

Dyar, H.G. 1908b. A review of the North American Pyralinae (Lepidoptera, Pyralidae). Proceedings of the Entomological Society of Washington 10: 96-102.

Dyar, H.G. 1908c. A revision of some species of Noctuidae heretofore referred to the genus Homoptera Boisduval. Proceedings of the United States National Museum 34(1645): 209-275 plus plates.

Dyar, H.G. 1910a. The larva and foodplant of *Glyptocera consobrinella* Zeller (Lepidoptera, Pyralidae). Proceedings of the Entomological Society of Washington 12: 52.

Dyar, H.G. 1910b. Notes on Megalopygidae. Proceedings of the Entomological Society of Washington 12(4): 161-189.

Dyar, H.G. 1910c. Descriptions of some new species and genera of Lepidoptera from Mexico. Proceedings of the United States National Museum 38(1742): 229-273.

Dyar, H.G. 1913a. Family: Cossidae. pp. 1263-1287 In: A. Seitz (ed.).The Macrolepidoptera of the American Region; The American Bombyces and Sphinges. Alfred Kernen Verlag, Stuttgart, Germany. Volume 6.

Dyar, H.G. 1913b. A note on the Macrothecinae. Insecutor Inscitiae Menstruus 1(2); 22-24.

Dyar, H.G. 1913c. Descriptions of new Lepidoptera, chiefly from Mexico Proceedings of the United States National Museum 44(1951) 279-324.

Dyar, H.G. 1914a. Report on the Lepidoptera of the Smithsonian Biological Survey of the Panama Canal Zone. Proceedings of the United States National Museum 47(2050): 139-350.

Dyar, H.G. 1914b[1915]: Descriptions of new species and genera of Lepidoptera from Mexico. Proceedings of the United States National Museum, Washington 47(2054): 365-409.

Dyar, H.G. 1914c. New American Lepidoptera. Insecutor Inscitiae Menstruus 2(11): 161-164.

Dyar, H.G. 1914d. A note on *Phobolosia* and *Melanomma*. Insecutor Inscitiae Menstruus 2(1): 19-10.

Dyar, H.G. 1914e. The noctuid moths of the genera *Palindia* and *Dyomyx*. Proceedings of the United States National Museum 47(2046): 95-116.

Dyar, H.G. 1915a. Report on the Lepidoptera of the Smithsonian biological survey of the Panama Canal Zone. Proceedings of the United States National Museum 47(2050): 139-350.

Dyar, H.G. 1917a. Notes on North American Schoenobiinae (Lepidoptera, Pyralidae). Insecutor Inscitiae Menstruus 5(4-6): 79-84.

Dyar, H.G. 1917b. Seven new Crambids from the United States. Insecutor Inscitiae Menstruus 5(4-6): 84-97.

Dyar, H.G. 1921a. New forms of American moths (Lepidoptera). Insecutor Inscitiae Menstruus 9: 59-68.

Dyar, H.G. 1921b. New American Noctuidae and notes (Lepidoptera). Insecutor Inscitiae Menstruus 9(1/3): 40-45.

Dyar, H.G. 1923a. New American Lepidoptera. Insecutor Inscitiae Menstruus 11: 12-30.

Dyar, H.G. 1924. A new noctuid from Louisiana (Lepidoptera, Noctuidae). Insecutor Inscitiae Menstruus 12: 21-22.

Dyar, H.G. 1925a. Notes on some American Phycitinae (Lepidoptera, Pyralidae). Insecutor Inscitiae Menstruus 13: 220-226.

Dyar, H.G. 1925b. Some new American moths (Lepidoptera). Insecutor Inscitiae Menstruus 13(1-3): 1-19.

Dyar, H.G. 1928. Notes on cactus moths allied to *Melitara*, with two new genera and one new species. Proceedings of the Entomology Society of Washington 30(8): 133-137.

Dyar, H.G. 1935. Limacodidae. Pp. 1104-1139, pl. 164-167 In: A. Seitz (ed.). Die Gross-Schmetterlinge Amerikanischen Faunengebietes. 6 Band, Die Amerikanischen Spinner und Schwarmer. Verlag des Seitzschen Werkes, Stuttgart, Germany.

Dyar, H.G. and C. Heinrich. 1927. The American moths of the genus *Diatraea* and allies. Proceedings of the United States National Museum 71: 1-48, plates 1-19.

Dyer, L.A., D.L. Wagner, H.F. Greeney, A.M. Smilanich, T.J. Massad, M.L. Robinson, M.S. Fox, R.F. Hazen, A.E. Glassmire, N.A. Pardikes, K.B. Fredrickson, C.V. Pearson, G. Gentry, and J.O. Stireman III. 2012. Novel insights into tritrophic interaction diversity and chemical ecology using 16 years of volunteer-supported research. American Entomologist 58(1): 15-19.

Easterbrooke, M.A. 1986. Damage to blueberry (*Vaccinium corymbosum*) by *Cacoecimorpha pronubana* (Hübner). Entomologist’s Record and Journal of Variation 98: 218.

Eaton, D.T. 2011. Borers in New Hampshire Apple Trees. University of New Hampshire Cooperative Extension Fact Sheet. extension.unh.edu

Eckberg, J.R. 2011. Las Vegas Wash Invertebrate Inventory, 2000-2010. Southern Nevada Water Authority Las Vegas Wash Project Coordination Team, Research and Environmental Monitoring Study Team & Las Vegas Wash Coordination Committee.

Edwards, H. 1875. Pacific coast Lepidoptera. No. 11. List of the Sphingidae of California and adjacent districts, with description of new species. Proceedings of the California Academy of Sciences 6: 86-95.

Edwards, H. 1877. Pacific coast Lepidoptera. No. 16. Notes on the transformations of some species of Lepidoptera, not hitherto recorded. Proceedings of the California Academy of Sciences, 7: 19-24.

Edwards, H. 1882. Notes on N. American AEgeridae, with descriptions of new forms. Papilio 2(4): 52-57.

Edwards, H. and S.L. Elliot. 1883. On the transformation of some species of Lepidoptera. Papilio 3: 127-136.

Edwards, W.H. 1882. Description of butterflies taken in Arizonia by Jacob Doll, 1881. Papilio 2(2): 19-29.

Efetov, K.A. and G.M. Tarmann. 2024. An annotated catalogue of the Procridinae of the World (Lepidoptera: Zygaenidae). SHILAP Revista de Lepidopterología 52(207): 409-547.

EFSA PLH Panel (EFSA Panel on Plant Health), C. Bragard, P. Baptista, E. Chatzivassiliou, F. Di Serio, P. Gonthier, J.A. Jaques Miret, A.F.Justesen, C.S. Magnusson, P. Milonas, J.A. Navas-Cortes, S. Parnell, R. Potting, P.L. Reignault, E. Stefani, H.-H.Thulke, W. Van der Werf, A. Vicent Civera, J. Yuen, L. Zappalà, J.-C. Grégoire, C. Malumphy, A. Akrivou. V. Kertesz and A. MacLeod. 2024. Pest categorization of Diaphania indica. EFSA Journal 22(5): e8806.

Ehrlich, P.R. 1976. *Papilio xuthus* (Papilionidae) in Hawaii. Journal of the Lepidopterists' Society 30(2): 149-150.

Eichlin, T.D. 1975. Guide to the adult and larval Plusiinae of California (Lepidoptera: Noctuidae). Occasional Papers in Entomology 21: 1-74.

Eichlin, T.D. 1980. *Stenolechia bathrodya*s Meyrick, a recently introduced pest of ornamental conifers in southern coastal California (Lepidoptera: Gelechiidae). Pan-Pacific Entomologist 56(3): 213-219.

Eichlin, T.D. 1992. A new Texas clear wing moth (Sesiidae: Sesiinae). Journal of the Lepidopterists' Society 46(4): 265-268

Eichlin, T.D. 1995. A new North American clear wing moth and notes on a rare species (Sesiidae). Journal of the Lepidopterists' Society 49(2): 114-118.

Eichlin, T.D. and H.B. Cunningham, 1972. Larvae of *Haploa clymene* (Arctiidae) hibernating on concrete bridges. Journal of the Lepidopterists' Society 24(4): 271.

Eichlin, T.D. and H.B. Cunningham. 1978. The Plusiina (Lepidoptera: Noctuidae) of American North of Mexico, emphasizing genitalic and larval morphology. United States Department of Agriculture Technical Bulletin no. 1567. U.S. Government Printing Office, Washington, DC.

Eichlin, T.D. and W.D. Duckworth. 1988. Sesioidea: Sesiidae. Fascicle 5.1. The Moths of North America North of Mexico. The Wedge Entomological Research Foundation, Washington.

Eichlin, T.D. and W.H. Taft. 1988. A new *Sesia* clearwing moth from Michigan (Sesiidae). Journal of the Lepidopterists' Society 42(3): 231-235.

Eiseman, C.S. 2014a. New host and distributional record for *Dichrorampha incanana* (Clemens, 1860) (Tortricidae: Olethreutinae). Journal of the Lepidopterists’ Society 68(4): 295-296.

Eiseman, C.S. 2014b. Notes on the larval habits and parasitoids of Rhopobota dietziana (Kearfott, 1907) (Tortricidae: Olethreutinae). Journal of the Lepidopterists’ Society 68(3): 218-220.

Eiseman, C.S. 2016. North American leafminers (Lepidoptera: Gelechiidae, Momphidae) on the evening primrose family (Onagraceae): new host, parasitoid, and distributional records. Proceedings of the Entomological Society of Washington 118(4): 510-518.

Eiseman, C.S. 2018. A new host plant and parasitoid record for the snapdragon plume moth (*Stenoptilodes antirrhin*a (Lange)), with a summary of records of leafmining in Pterophoridae (Lepidoptera). Proceedings of the Entomological Society of Washington, 120(2): 452-457. <https://doi.org/10.4289/0013-8797.120.2.452>

Eiseman, C.S. 2019. A review of natural history data for Nearctic *Epermenia* Hübner (Lepidoptera: Epermeniidae), with the first account of the larval habits of *E. albapunctella* Busck. Proceedings of the Entomological Society of Washington 121(1): 107-114. doi.org/10.4289/0013-8797.121.1.107.

Eiseman, C.S. 2020. Leafminers of North America, 2nd Edition. Self Published Digital, avaiable at http://charleyeiseman.com/leafminers/. <September 2020 update>

Eiseman, C.S. 2022. Leafminers of North America. Second Edition. Charley Eiseman. URL: http://charleyeiseman.com/leafminers/ [as reported in supplement data: Chen, T.B., X.H. Dai, and C. Eiseman. 2022. A checklist of gymnosperm-feeding leafminers (Arthopoda, Insecta) in North America and Europe. Biodiversity Data Journal 10 (2022): e91313; 1-25]

Eiseman, C.S. 2024a. New rearing records of Lepidoptera from wild sarsaparilla (Araliaceae: *Aralia nudicaulis* L.), with a review of species reported from Nearctic *Aralia* spp. Proceedings of the Entomological Society of Washington 125(4): 471-492.

Eiseman, C.S. 2024b. On the hosts and larval Habits of *Framinghamia helvalis* (Walker) (Lepidoptera: Crambidae). Proceedings of the Entomological Society of Washington 125(2): 268-272.

Eiseman, C.S. and D.R. Davis. 2017. A new species of *Macrosaccus* (Lepidoptera: Gracillariidae: Lithocolletinae) from Arizona, USA. Zootaxa 4358(2): 385-392.

Eiseman, C.S. and D.R. Davis. 2020. Wrong side of the leaf: assigning some Lithocolletinae species (Lepidoptera: Gracillariidae) to their proper genera. Zootaxa 4751(2): 201-237.

Eiseman, C.S. and A.S. Jensen. 2015. Insects feeding on sea lavender (Plumbaginaceae: *Limonium carolinianum* [Walt.] Britt) along the New England coast. Entomological News 124(5): 364-369.

Eiseman, C.S., D.R. Davis, J.A. Blyth, D.L. Wagner, M.W. Palmer, and T.S. Feldman. 2017a. A new species of *Marmara* (Lepidoptera: Gracillariidae: Marmarinae), with an annotated list of known hostplants for the genus. Zootaxa 4337(2): 198-222.

Eiseman, C.S, T.S. Feldman, E.F. Lopresti, and M.W. Palmer. 2017b. First North American records of *Porphyrosela minuta* Clarke (Lepidoptera: Gracillariidae), with notes on its native congener, *P. desmodiella* (Clemens). Proceedings of the Entomological Society of Washington 119(1): 18-23.

Eiseman, C.S., K.A. Austin, J.A. Blyth, and T.S. Feldman. 2020a. New records of leaf-mining Tortricidae (Lepidoptera) in North America, with the description of a new species of Grapholita. Zootaxa 4748(3): 514-530.

Eitschberger, U. 2002. Revidierte Checkliste der Gattungen *Paonias* Hübner, [1819] sowie Smerinthus Latreille, [1802] mit der Beschreibung neuer Paonias-Arten (Lepidoptera, Sphingidae). Neue Entomologische Nachrichten 23: 1-49.

Eitschberger, U. 2015. Die Gattung *Protambulyx* Rothschild & Jordan, 1903 mit der Beschreibung von zwei neuen Arten (Lepidoptera, Sphingidae). Neue Entomologische Nachrichten 70: 153-187.

El Anbri, C., T. Eddaya, A. Boughdad, P. Chaimbault, and A. Zaid. 2021. Biological characteristics of *Orthonama obstipata* (Fabricius, 1794) (Lepidoptera: Geometridae), an emerging defoliator of mint (*Mentha spicata* L., 1753) in Morocco. Moroccan Journal of Agricultural Sciences 2(3): 119-129.

Eldredge, E.P. and C.R. Baird. 1995. First record of orange mint moth (Lepidoptera: Pyralidae) on commercial peppermint in Idaho. Journal of the Entomological Society of British Columbia 92: 107-109.

Eliot, L.M. and C.G. Soule. 1902. Caterpillars and their moths. The Century Company, New York.

Elkinton, J.S., A. Liebhold, G.H. Boettner, and M. Sremac. 2014. Invasion spread of *Operophtera brumata* in northeastern United States and hybridization with *O. bruceata*. Biological Invasions 16: 2263-2272.

Elkinton, J., G. Boettener, A. Liebhold, and R. Gwiazdowski. 2015. Biology, Spread, and Biological Control of Winter Moth in the Eastern United States. USDA Forest Service Publication FHTET-2014-07, Morgantown, West Virginia.

Ellis, A. and M.D. Brown, 1998. Effects of hostpalnt species and artificial diet on the growth of buckeye (*Junonia coenia*) and painted lady (*Vanessa cardui*) caterpillars (Nymphalidae). Journal of the Lepidopterists’ Society 52(1): 73-83.

Ellison, A.M. 1991. Ecology of case-bearing moths (Lepidoptera: Coleophoridae) in a New England salt marsh. Environmental Entomology 20(3): 857-864.

Ellsbury, M.M. 2016. 2015 Season Summary. Zone 5 Great Plains: Saskatchewan, Manitoba, North Dakota, South Dakota, Nebraska, Kansas, Oklahoma. News of the Lepidopterist’ Society 58(S1): 80-99.

Ellsbury, M.M. 2017. 2016 Season Summary. Zone 5 Great Plains: Saskatchewan, Manitoba, North Dakota, South Dakota, Nebraska, Kansas, Oklahoma. News of the Lepidopterist’ Society 59(S1): 75-95.

Ellwood, C.V. and J.M.V. Harvey. 1990. The Lady Blake Collection: Catalogue of Lady Edith Blake's collection of drawings of Jamaican Lepidoptera and plants. Bulletin of the British Museum (Natural History) 18(2): 145-202.

Elmore, J.C. and A.F. Howland 1943. Life history and control of the tomato pinworm. United States Department of Agriculture Technical Bulletin 841: 1-30.

Ely, C.R. 1910. New species of North American Microlepidoptera. Proceedings of the Entomological Society of Washington 7: 67-73.

Ely, C.R. 1915. New species of the genus *Gracilaria* and notes on two already described species (Lepidoptera, Gracilariidae). Insecutor Inscitiae Menstruus 3(5-7): 51-62.

Ely, C.R. 1917[1918]. A revision of the North American Gracilariidae from the standpoint of venation. Proceedings of the Entomological Society of Washington 19B(1-4): 29-77.

EMEC:EMEC. 2015. Essig Museum of Entomology, University of California, Berkeley California. In: SCAN. P.L. Heinrich, E. Gilbert, N.S. Cobb, and N. Franz. Symbiota collections of arthropods network (SCAN): A data portal built to visualize, manipulate, and export species occurrences. [Dataset]. http://scan-bugs.org/portal/collections/index.php [Accessed 08 March 2019]

Emery, V.J., J.F. Landry and C.G. Eckert. 2009. Combining DNA barcoding and morphological analysis to identify specialist floral parasites (Lepidoptera: Coleophoridae: Momphinae: *Mompha*). Molecular Ecology Resources 9(s1): 217-223.

Emmel, T.C. and J.F. Emmel. 1973. The butterflies of southern California. Natural History Museum of Los Angeles County, Science Series 26.

Emmel, T.C. and J.F. Emmel. 1974. Ecological studies of Rhopalocera in a Sierra Nevadan community - Donner Pass, California. V. Faunal additions and foodplant records since 1962. Journal of the Lepidopterists' Society. 28(4): 344-347.

Emmel, T.C. and J.B. Heppner. 1991. An Advertisement for “Atlas of North American Lepidoptera”. Gainesville: Scientific Publishers.

Emmel, J.F. and R. Mattoni. 1989 [1990]. A new subspecies of *Satyrium auretorum* (Lycaenidae) from the Santa Monica mountains of southern California. Journal of Research on the Lepidoptera 28(1-2): 100-104.

Emmel, J.F., T.C. Emmel, and S.O. Mattoon. 2012. A new species of *Cercyonis* (Lepidoptera: Satyridae) from northern California. Bulletin of the Allyn Museum 163: 12 pp.

Emmet, A.M. 1985. Ochsenheimeriidae. Lyonetiidae. pp. 208-239 in: A.M. Emmet (Ed.), The Moths and Butterflies of Great Britain and Ireland, Volume 2, Cossidae - Heliodinidae. Harley Books, Colchester, Essex, England.

Emmet, A.M. 1987. 1986 Annual Exhibition. British Microlepidoptera. Species from Essex. Proceedings and Transactions of the British Entomological and Natural History Society 20(2): 50-51.

Emmet, A.M. 1998. Larvae of *Trachycera suavella* (Zincken, 1818) (Lep.: Pyralidae) found feeding gregariously. Entomologists Record and Journal of Variation 110: 35-36.

Emmet, A.M., I.A. Watkinson and A.M. Emmet, 1985. Gracillariidae. pp. 244-362 *in* A.M. Emmet (ed.), The Moths and Butterflies of Great Britain and Ireland, Volume 2, Cossidae - Heliodinidae. Harley Books, Colchester, Essex, England.

Emmet, A.M., R.J. Heckford, J.R. Langmaid, and S.M. Palmer 2002. Chelariinae. pp. 221-226 *in* A.M. Emmet and J.R. Langmaid (eds.), The Moths and Butterflies of Great Britain and Ireland, Volume 4, part 2, Gelechiidae. Harley Books, Colchester, Essex, England.

Engel, H. 1908-1909. A preliminary list of the Lepidoptera of western Pennsylvania collected in the vicinity of Pittsburgh. Annals of the Carnegie Museum 5(1): 27-136.

Engelhardt, G.P. 1925. Studies of North American Aegeriidae (Lepidoptera). Bulletin of the Brooklyn Entomological Society 20(4): 153- 158.

Engelhardt, G.P. 1946. The North American clearwing moths of the family Aegeriidae. United States National Museum Bulletin 190: 1-222.

Epstein, M.E. 1996. Revision and phylogeny of the limacodid-group families, with evolutionary studies on slug caterpillars (Lepidoptera: Zygaenoidea). Smithsonian Contributions to Zoology 582: 1-102.

Epstein, M.E. 1997. Biology of *Dalcerides ingenita* (Lepidoptera: Dalceridae). Tropical Lepidoptera 8(2): 49-59.

Epstein, M.E. 2005. Lepidoptera lab report, 2004. P. 35 in: U.C. Kodira, (ed.). 2004 plant pest diagnostics laboratory report. California Plant Pest Diagnostics Branch Annual Report, California Department of Food and Agriculture.

Epstein, M.E. and V.O. Becker. 1993. Combinations and synonymies in new world Limacodidae, Megalopygidae, Lasiocampidae and Arctiidae (Lepidoptera). Revista Brasileira de Zoologia 10(s): 289-319.

Epstein, M.E., S.R. Smedley, and T. Eisner. 1994. Sticky integumental coating of a dalcerid caterpillar: a deterrent to ants. Journal of the Lepidopterists' Society 48(4): 381-386.

Ersch, E.A. 2009. Effects of Plant Community Characteristics on Insect Abundance: Implications for Sage-grouse Brood-rearing Habitat. Masters’ Thesis. Oregon State University, Corvallis, Oregon.

Espindola, C.B. and L. Gonçalves. 2000. Biology of *Oxydia vesulia* (Cramer, 1779) (Lepidoptera: Geometridae). Floresta e Ambiente 7(1): 80-87.

Evans, D. 1960. A revision of the genus *Enypia* (Lepidoptera: Geometridae). Annals of the Entomological Society of America 53(5): 560-574.

Evans, D. 1968. Field Key to Geometrid Larvae of the British Columbia Coast Forest. Revised. Forest Research Laboratory Victoria, British Columbia, Internal Report BC-7.

Evans, D.C. and E. Zambrano. 1991. Insect damage in maize of highland Ecuador and its significance in small farm pest management." International Journal of Pest Management 37(4): 409-414.

Evans, D.E., O. Andrade, and W.M. Mathenge. 1968. The biology and control of *Archips occidentalis* (Wals.) and *Tortrix dinota* Meyr. (Lepidoptera: Tortricidae) on coffee in Kenya. Journal of the Entomological Society of South Africa 31: 133-140.

Evans, J.W. 1952. The Injurious Insects of the British Commonwealth. Commonwealth Institute of Entomology, London.

Evans, W.H. 1955. A Catalogue of the American Hesperiidae in the British Museum (Natural History). Part IV. Hesperiinae and Megathyminae. British Museum, London pls 54-88, pp 1-499.

Ezzat, Y.M. and N.H. Nazmi. 1970. Survey and Classification of the Leaf-roller Moths in U.A.R. (Tortricidae: Lepidoptera). United Arab Republic Ministry of Agriculture Technical Bulletin 1: 1-58.

Fales, J.H. 1987. The butterflies of Rock Creek Park, Washington D.C. Maryland Naturalist 31(1): 5-24.

Falkovitsh, M. I. 2006. Host-plant relationships of the casebearers (Lepidoptera, Coleophoridae): Communication III. Entomological Review 86(3): 264-286.

Farahbakhsh, G. 1961. Checklist of economically important insects and other enemies of plants and agricultural products in Iran. Department of Plant Protection, Ministry of Agriculture, Publication No. 1. 153 pp.

Farquhar, D.W. 1934. Notes on a Psychid new to North America (Fumea Casta Pallas, Lepidoptera: Psychidæ). Psyche 41: 19-29.

Fasoranti, J.O. 1984. The life history and habits of a ceanothus leaf miner, *Tischeria immaculata* (Lepidoptera: Tischeriidae). The Canadian Entomologist 116(11): 1441-1448.

Faubert, H. and R.A. Casagrande 2002. 15. Cypress spurge. Pp. 195-207 In: R. Van Driesche, et al. (eds.). Biological Control of Invasive Plants in the Eastern United States. USDA Forest Service Publication FHTET-2002-04. 413 pp.

Faucheux, M.J. 2013. Sensillum types on the proboscis of the Lepidoptera: a review." In Annales de la Société Entomologique de France, vol. 49(1): 73-90.

Fauske, G. and R. Snow. 1985. Moth communities of the North Unit of Theodore Roosevelt National Park. Proceedings of the North Dakota Academy of Science 39: 31.

Favre, E. 1906. Contributions à l'étude de la faune des microlépidoptères du Valais. Bulletin de la Murithienne 35: 146-239.

Fazekas, I. 2008. The species of the genus *Aethes* Billberg 1821 of Hungary (Lepidoptera: Tortricidae). Natura Somogyiensis 12: 133-168.

Feltwell, J. 1993. The Encyclopedia of Butterflies. Prentice Hall, New York.

Ferge, L.A. 1996. 1997 Season Summary Zone 8 Midwest, Missouri, Kentucky, West Virginia, Ohio, Indiana, Illinois, Iowa, Michigan. News of the Lepidopterists’ Society 38(2): 43-50.

Ferge, L.A. 1997. 1996 Season Summary Zone 8 Midwest, Missouri, Kentucky, West Virginia, Ohio, Indiana, Illinois, Iowa, Michigan. News of the Lepidopterists’ Society 39(2): 54-65.

Ferge, L.A. 1998. 1997 Season Summary Zone 8 Midwest, Missouri, Kentucky, West Virginia, Ohio, Indiana, Illinois, Iowa, Michigan. News of the Lepidopterists’ Society 40: 45-52.

Ferge, L.A. 1999. 1998 Season Summary Zone 8 Midwest, Missouri, Kentucky, West Virginia, Ohio, Indiana, Illinois, Iowa, Michigan. News of the Lepidopterists’ Society 41: 53-64.

Ferge, L.A. 2000. 1999 Season Summary Zone 8 Midwest, Missouri, Kentucky, West Virginia, Ohio, Indiana, Illinois, Iowa, Michigan. News of the Lepidopterists’ Society 41(S1): 52-63.

Ferge, L.A. 2001. 2000 Season Summary Zone 8 Midwest, Missouri, Kentucky, West Virginia, Ohio, Indiana, Illinois, Iowa, Michigan. News of the Lepidopterists’ Society 43(S1): 41-50.

Ferge, L.A. 2002. 2001 Season Summary Zone 8 Midwest, Missouri, Kentucky, West Virginia, Ohio, Indiana, Illinois, Iowa, Michigan. News of the Lepidopterists’ Society 44(S1): 44-56.

Ferge, L.A. 2003. 2004 Season Summary Zone 8 Midwest, Missouri, Kentucky, West Virginia, Ohio, Indiana, Illinois, Iowa, Michigan. News of the Lepidopterists’ Society 45(S1): 46-56.

Ferge, L.A. 2004. 2003 Season Summary Zone 8 Midwest, Missouri, Kentucky, West Virginia, Ohio, Indiana, Illinois, Iowa, Michigan. News of the Lepidopterists’ Society 46(S1): 56-64.

Ferge, L.A. 2005. 2004 Season Summary Zone 8 Midwest, Missouri, Kentucky, West Virginia, Ohio, Indiana, Illinois, Iowa, Michigan. News of the Lepidopterists’ Society 47(S1): 69-76.

Ferge, L.A. 2006. 2005 Season Summary Zone 8 Midwest, Missouri, Kentucky, West Virginia, Ohio, Indiana, Illinois, Iowa, Michigan. News of the Lepidopterists’ Society 48(S1): 80-86.

Ferge, L.A. 2007. 2006 Season Summary Zone 8 Midwest, Missouri, Kentucky, West Virginia, Ohio, Indiana, Illinois, Iowa, Michigan. News of the Lepidopterists’ Society 49(S1): 86-95.

Ferge, L.A. 2008. 2007 Season Summary. Zone 8. Midwest: Missouri, Kentucy, West Virgina, Ohio, Indiana, Illinois, Iowa, Minnesota, Wisconsin, Michigan. News of the Lepidopterists' Society 50(S1): 111-122.

Ferge, L.A. 2009. 2008 Season Summary. Zone 8. Midwest: Missouri, Kentucy, West Virgina, Ohio, Indiana, Illinois, Iowa, Minnesota, Wisconsin, Michigan. News of the Lepidopterists' Society 51(S1): 91-100.

Ferge, L.A. 2010a. 2009 Season Summary Zone 8 Midwest, Missouri, Kentucky, West Virginia, Ohio, Indiana, Illinois, Iowa, Michigan. News of the Lepidopterists’ Society 52(S1): 102-118.

Ferge, L. 2010b. Part II: 2009 Wisconsin Lepidoptera Season Summary. The Wisconsin Entomological Society Newsletter 37(2): 4-9.

Ferge, L.A. 2011a. 2010 Season Summary Zone 8 Midwest, Missouri, Kentucky, West Virginia, Ohio, Indiana, Illinois, Iowa, Michigan. News of the Lepidopterists’ Society 53(S1): 108-130.

Ferge, L.A. 2011b. Checklist of Wisconsin Butterflies. 2002 (revised 2011). Wisconsin Entomological Society Special Publication 5.

Ferge, L.A. 2012. 2011 Season Summary Zone 8 Midwest, Missouri, Kentucky, West Virginia, Ohio, Indiana, Illinois, Iowa, Michigan. News of the Lepidopterists’ Society 54(S1): 88-120.

Ferge, L.A. 2013. 2012 Season Summary Zone 8 Midwest, Missouri, Kentucky, West Virginia, Ohio, Indiana, Illinois, Iowa, Minnesota, Wisconsin, Michigan. News of the Lepidopterists’ Society 55(S1): 94-125.

Ferge, L.A. 2014. 2013 Season Summary Zone 8 Midwest, Missouri, Kentucky, West Virginia, Ohio, Indiana, Illinois, Iowa, Minnesota, Wisconsin, Michigan. News of the Lepidopterists’ Society 56(S1): 124-149.

Ferge, L.A. 2015. 2014 Season Summary Zone 8 Midwest, Missouri, Kentucky, West Virginia, Ohio, Indiana, Illinois, Iowa, Minnesota, Wisconsin, Michigan. News of the Lepidopterists’ Society 57(S1): 150-172.

Ferge, L.A., G.J. Balogh, and K.E. Johnson. 2108. Checklist of Wisconsin Moths (Superfamilies Mimallonoidea, Drepanoidea, Lasiocampoidea, Bombycoidea, Geometroidea, and Noctuoidea). Wisconsin Entomological Society Special Publication No. 6. 45 pp.

Ferguson, D.C. 1954a. The Lepidoptera of Nova Scotia part 1, Macrolepidoptera. Proceedings of the Nova Scotia Institute of Science 23: 161-375.

Ferguson, D.C. 1954b. A revision of the genus *Hypenodes* Doubleday with descriptions of new species (Lepidoptera, Phalaenidae). The Canadian Entomologist 86(7): 289-298.

Ferguson, D.C. 1955a. The Lepidoptera of Nova Scotia 1. Macrolepidoptera. Bulletin of the Nova Scotia Museum of Science, No.2.

Ferguson, D.C. 1955b. The North American species of *Calocalpe* Hubner (Lepidoptera, Geometridae). Canadian Entomologist 87(8): 325-330.

Ferguson, D.C. 1958. Notes on Larentiinae - New records and corrections (Lepidoptera: Geometridae). Canadian Entomologist 90(1): 42-43.

Ferguson, D.C. 1969. A Revision of the Moths of the Subfamily Geometrinae of America North of Mexico (Insects, Lepidoptera). Peabody Museum of Natural History Bulletin 29: 1-251.

Ferguson, D.C. 1971. The Moths of North America North of Mexico. Fascicle 20.2A Bombycoidea, Saturniidae comprising subfamilies Citheroniina, Hemileucinae (Part). E.W. Classey Limited and R.B.D. Pub, Inc. London, UK.

Ferguson, D.C. 1972a. The Moths of North America North of Mexico. Fascicle 20.2B. Bombycoidea, Saturniidae comprising subfamilies Hemileucinae (Conclusion), Saturniinae. E.W. Classey Limited and R.B.D. Pub, Inc. London, UK.

Ferguson, D.C. 1972b. New records of Lepidoptera from the United States (Arctiidae, Geometridae, Epiplemidae). Journal of the Lepidopterist Society 26(4): 222-225.

Ferguson, D.C. 1972c. Bombycoidea, Saturniidae (in part). In R.B. Dominick et aI. The Moths of America North of Mexico, fasc. 20.2B. E.W. Classey Ltd., London, England.

Ferguson, D.C. 1973. The species of the genus *Tacparia* Walker (Lepidoptera, Geometridae). Proceeding of the Entomological Society of Washington 75: 467-478.

Ferguson, D.C. 1974. A new species of the genus *Semiothisa* from the southeastern United States (Geometridae). Journal of the Lepidopterist Society 28(4): 297-301.

Ferguson, D.C. 1975. Host records of Lepidoptera reared in eastern North America. United States Department of Agriculture Technical Bulletin, no. 1521. U.S. Government Printing Office, Washington, DC.

Ferguson, D.C. 1977. A new North American species of *Apamea* formerly confused with *A. verbascoides* (Guenee) (Noctuidae). Journal of the Lepidopterists' Society 31(1): 57-62.

Ferguson, D.C. 1978. The Moths of North America North of Mexico including Greenland. Fascicle 22.2. Noctuoidea, Lymantriidae. E.W. Classey Limited and R.B.D. Pub, Inc. London, UK.

Ferguson, D.C. 1979. A new ghost moth from the southern Appalachian Mountains (Hepialidae). Journal of the Lepidopterists Society 33: 192-196.

Ferguson, D.C. 1982a. First occurrence of *Perizoma alchemillata* (Lepidoptera: Geometridae) on the mainland of North America. Canadian Entomologist 114(6): 543-543.

Ferguson, D.C. 1983a. A new genus and new species of Geometrid moth from Texas. Journal of the Lepidopterists' Society 37(1): 24-28.

Ferguson, D.C. 1983b. The identity of two monotypic Geometrid genera wrongly attributed to the Nearctic fauna (Geometridae). Journal of the Lepidopterists' Society 37(2): 146-147.

Ferguson, D.C. 1983c. On the status of *Pseudothyatlra expultrix* (Grt.) and *Euthyatira pennsylvanicii*.J. B. Smith (Thyatiridae). Journal of the Lepidopterists' Society 37(2): 179-180.

Ferguson, D.C. 1985a. Geometroidae: Geometridae (part): Geometrinae. In: R.B. Dominick et al. (eds). The Moths of North America North of Mexico. Fascicle 18.1. The Wedge Entomological Research Foundation, Washington.

Ferguson, D.C. 1985b. Contributions toward reclassification of the world genera of the tribe Arctiini, part 1: Introduction and a revision of the *Neoarctia*-*Grammia* group (Lepidoptera: Arctiidae: Arctiinae). Entomography 3: 181-275.

Ferguson, D.C. 1987. *Xanthorhoe clarkeata* (Geometridae), a new species and possible endemic of the Queen Charlotte Islands, British Columbia. Journal of the Lepidopterists’ Society 42(2): 98-103.

Ferguson, D.C. 1988 [1989]. New species and new nomenclature in the American Acronictinae (Lepidoptera: Noctuidae). Journal of Research on the Lepidoptera 26: 201-218.

Ferguson, D.C. 1991a. First record of the genus *Acrapex* from the New World, with description of a new species from the Carolinas and Virginia (Noctuidae: Amphipyrinae). Journal of the Lepidopterists’ Society 45(3): 209-214.

Ferguson, D.C. 1991. Review of the genus *Epimorius* Zeller and first report of the occurrence of *E. testaceellus* Ragonot in the United States (Pyralidae: Galleriinae). Journal of the Lepidopterists’ Society 45: 117-123.

Ferguson, D.C. 1992. *Franclemontia interrogans* (Walker), a new genus for an old species (Lepidoptera: Noctuidae: Amphipyrinae). Journal of the New York Entomological Society 100(2): 257-266.

Ferguson, D.C. 1993. A revision of the species *Nematocampa* (Geometridae: Ennominae) occuring in the Unites States and Canada. Journal of the Lepidopterists’ Society 47(1): 60-77.

Ferguson, D.C. 1997. Review of the New World Bagisarinae with description of two new species from the southern United States (Noctuidae). Journal Lepidopterists Society 51: 344-357.

Ferguson, D.C. 2008. Geometroidea, Geometridae (part: Abaxini, Cassymini, Macariini). In Dominick R.B. et al. (eds): The Moths of North America, Fascicle 17.2. The Wedge Entomological Research Foundation, Washington, DC. 431 pp.

Ferguson, D.C. 2009. A revision of the red-brown Caberine geometrids of the southeastern United States (Geometridae: Caberini). Tropical Lepidoptera Research 19(1): 35-51.

Ferguson, D.C. and V.A. Brou Jr. 1981. A new species of *Automeris* Hubner (Saturniidae) from the Mississippi river delta. Journal of the Lepidopterists' Society 35: 101-105.

Ferguson, D.C. and S.W. Choi. 2001. A new species of *Eulithis* Hübner (Lepidoptera: Geometridae) from California. Proceedings of the Entomological Society of Washington 301: 367-372.

Ferguson, D.C. and E.C. Knudson. 1986. Four new United States records of moths from Texas. Journal of the Lepidopterists' Society 40(4): 353-354.

Ferguson, D.C. and M.J. Mello. 1996. The introduction and spread of *Chloroclystis rectangulata* (L.) (Geometridae), and its first reported occurrences in the United States. Journal Lepidopterists Society 50: 145-148.

Ferguson, D.C. and P.A. Opler. 2006. Checklist of the Arctiidae (Lepidoptera: Insecta) of the continental United States and Canada. Zootaxa 1299: 1-33.

Ferguson, D.C., A. Blanchard, and E.C. Knudson. 1983. A new genus and species of Geometridae (Lepidoptera) from Big Bend National Park, Texas. Proceedings of the Entomological Society of Washington 85: 552-556.

Ferguson, D.C., A. Blanchard, and E.C. Knudson. 1984. A new species of *Neodavisia* Barnes & McDunnough (Lepidoptera: Pyralidae) from southern Texas. Proceedings of the Entomological Society of Washington 86: 769-772.

Ferguson, D.C., D.J. Hillburn and B. Wright. 1991. The Lepidoptera of Bermuda: their food plants, biogeography, and means of dispersal. Memoirs of the Entomological Society of Canada 158: 2-100.

Ferguson, D.C., C.E. Harp, P.A. Opler, R.S. Peigler, J.A. Powell, and M.J. Smith. 1999. Moths of North America. Jamestown, ND: Northern Prairie Wildlife Research Center Home Page. http://www.npwrc.usgs.gov/resource/distr/lepid/moths/mothsusa.htm (Version 12DEC2003).

Ferguson, D.C., P.A. Opler, M.J. Smith, and J.P. Donahue. 2000. Moths of Western North America 3. Distribution of Arctiidae of Western North America. Part 1. Text, Maps, and References. C.P. Gillette Arthropod Biodiversity Museum, Colorado State University, Ft. Collins, Colorado.

Ferguson, G.R. 1940. Life history of the western strawberry leaf roller with notes on its control. Journal of Economic Entomology 33(1) : 121-123.

Fernald, C.H. 1879. On the genus *Argyria* Huebner. North American Entomologist 1(12): 100-102.

Fernald, C. H. 1882a. A synonymical catalogue of the described Tortricidae of North America, north of Mexico. Transactions of the American Entomological Society 10: 1-64.

Fernald, C.H. 1886. The Sphingidae of New England. Privately published, Augusta, ME.

Fernald, C.H. 1896. The Crambidae of North America. Massachusetts Agricultural College.

Fernald, C.H. 1900. On the North American species of *Choreutis* and its allies. The Canadian Entomologist 32(8): 236-245.

Fernández Henandez, D.M. 2004. New range extensions, larval hostplant records and natural history observations of Cuban butterflies. Journal of the Lepidopterists' Society 58: 48-50.

Fernández-Hernández, D.M. 2007. Butterflies of the agricultural experiment station of tropical roots and tubers, and Santa Ana, Camagüey, Cuba: An annotated list. Acta Zoológica Mexicana 23(2): 43-75.

Ferner, J.W. and M. Rosenthal. 1981. A cryptic moth, *Schinia masoni* (Noctuidae), on *Gaillardia aristata* (Compositae) in Colorado. The Southwestern Naturalist 88-90.

Ferris, C.D. 1973a. A revision of the *Colias alexandra* complex (Pieridae) aided by ultraviolet reflectance photography with designation of a new subspecies. Journal of the Lepidopterists Society 27(1): 57-73.

Ferris, C.D. 1973b. Life history of *Callophrys sheridanii sheridanii* (Lycaenidae) and notes on other species. Journal of the Lepidopterists Society 27(4): 279-283.

Ferris, C.D. 1977. Taxonomic revision of the Species *dorcas* Kirby and *helloides* Boisduval in the genus *Epidemia* Scudder (Lycaenidae, Lycaeninae). Bulletin of the Allyn Museum 45: 1-42.

Ferris, C.D. 1985. A new subspecies of *Apodemia hypoglauca* (Godman & Salvin) from the Yucatan Peninsula (Lycaenidae: Riodininae). Bulletin of the Allyn Museum 494: 1-7.

Ferris, C.D. 2003. A new species of *Pero* from Wyoming and Idaho (Lepidoptera: Geometridae). Bulletin of the Allyn Museum 142: 1-4.

Ferris, C.D. 2004a. Taxonomic note on four poorly known Arizona *Eupithecia* Curtis (Lepidoptera: Geometridae: Eupitheciini). Zootaxa 738: 1-19.

Ferris, C.D. 2004b. A new species of *Passadenoides* Neunzig from Wyoming and New Mexico (Lepidoptera: Pyralidae: Phycitinae). Zootaxa 705: 1-8.

Ferris, C.D. 2004c. A new species of *Nasusina* Pearsall from Colorado (Lepidoptera: Geometridae: Eupitheciini). Zootaxa 467: 1-9.

Ferris, C.D. 2005. A new species of *Eucosma* Hübner from the Western United States (Lepidoptera: Tortricidae: Eucosmini). Zootaxa 806: 1-8.

Ferris, C.D. 2006a. Taxonomic note on *Eupithecia cupressata* Pearsall (Lepidoptera: Geometridae: Eupitheciini). Zootaxa 1255: 63-68.

Ferris, C.D. 2006b. Arizona *Phyllodonta* revisited (Geometridae). News of the Lepidopterists' Society 48(2): 41.

Ferris, C.D. 2006c. An interesting Geometrid from eastern Utah. News of the Lepidopterists' Society 48(1): 9.

Ferris, C.D. 2007a. First United States record for *Bulia schausi* Richards: Noctuidae. News of the Lepidopterists’ Society 49: 9.

Ferris, C.D. 2007b. Three new species of *Eupithecia* Curtis from Arizona and New Mexico with discussion of associated species (Lepidoptera: Geometridae: Eupitheciini). Zootaxa 1516: 49-60.

Ferris, C.D. 2007c. *Astalotesia hollandi* Rindge (Geometridae) from Arizona. News of the Lepidopterists' Society 49(2): 65.

Ferris, C.D. 2009a. *Synaxis triangulata* (Barnes & McDunnough) moved to *Caripeta* Walker (Geometridae: Ennominae). Journal of the Lepidopterists’ Society 63(3): 164-165.

Ferris, C.D. 2009b. *Metanema brunneilinearia* Grossbeck misplaced in *Synaxis* Hulst (Geometridae: Ennominae). Journal of the Lepidopterists’ Society 63(3): 166-168.

Ferris, C.D. 2010a. A new Geometrid genus and species from southeastern Arizona (Ennominae: Nacophorini). Journal of the Lepidopterists’ Society 64(3): 147-153.

Ferris, C.D. 2010b. A revision of the genus *Antepione* Packard with description of the new genus *Pionenta* Ferris (Lepidoptera, Geometridae, Ennominae). ZooKeys 71: 49-70.

Ferris, C.D. 2010c. A new *Antaeotricha* species from Southeastern Arizona (Gelechioidea, Elachistidae, Stenomatinae). ZooKeys 57: 59-62.

Ferris, C.D. 2010d. Appendix B. Preliminary lists of butterflies and moths (Lepidoptera) documented on the Pitchfork Ranch. Moths. Pp. 22-24 In: A.T. Cole and C. Cole. Cienaga Restoration at the Pitchfork Ranch (Grant County, New Mexico). Proceedings of the Second Natural History of the Gila Symposium, October 2008, The New Mexico Botanist, Special Issue No. 2: 11-28.

Ferris, C.D. 2012a. A new *Antaeotricha* species from Utah and New Mexico (Gelechioidea: Elachistidae: Stenomatinae). Journal of the Lepidopterists’ Society 66(3): 168-170.

Ferris, C.D. 2012b. A new phycitine species from New Mexico (Pyraloidea: Pyralidae: Phycitinae). Journal of the Lepidopterists' Society 66 (3): 165-167.

Ferris, C.D. 2012c. A new phycitine genus and species from Utah (Pyraloidea: Pyralidae: Phycitinae). The Journal of the Lepidopterists' Society 66(2): 76-80.

Ferris, C.D. 2013. Two new species of *Antaeotricha* Zeller from southeastern Arizona (Gelechioidea: Elachistidae: Stenomatinae). Journal of the Lepidopterists’ Society 67(1): 42-48.

Ferris, C.D. 2018. Lepidoptera of North America 14. Geometroidea Geometridae: Larentiinae: Eupitheciini (Part). Contributions of the C.P. Gillette Museum of Arthropod Diversity, Colorado State University, Fort Collins, Colorado.

Ferris, C.D. 2019. Lepidoptera of North America 14. Geometroidea Geometridae: Larentiinae: Eupitheciini (Part) [2019 version]. C.P. Gillette Museum of Arthropod Diversity, Department of Bioagricultural Sciences and Pest Management, Colorado State University, Fort Collins, Colorado.

Ferris, C.D. 2022. Revised Supplement to Lepidoptera of North America. 14. Geometroidea Geometridae: Larentiinae: Eupitheciini (Part) [2022 version]. Contributions of the C.P. Gillette Museum of Arthropod Diversity, Colorado State University, Fort Collins, Colorado.

Ferris, C.D. and J.J. Kruse. 2008. A new species of *Zeiraphera* Treitschke (Tortricidae). Journal of the Lepidopterists’ Society 62(1): 31-35.

Ferris, C.D. and J.D. Lafontaine. 2009. Review of the *Acontia areli* group with descriptions of three new species (Lepidoptera, Noctuidae, Acontiinae). In: B.C. Schmidt and J.D. Lafontaine, (Eds.). Contributions to the Systematics of New World Macro-Moths. ZooKeys 9: 27-46.

Ferris, C.D. and J.D. Lafontaine. 2010. Review of the North American species of *Marimatha* Walker with descriptions of three new species (Lepidoptera, Noctuidae, Eustrotiinae) and the description of *Pseudomarimatha flava* (Noctuinae, Elaphriini), a new genus and species confused with *Marimatha*. In: B.C. Schmidt and J.D. Lafontaine, (Eds.). Contributions to the systematics of New World macro-moths II. ZooKeys 39: 117-135.

Ferris, C.D. and N. McFarland. 2010. A new species of *Plataea* (Geometridae: Ennominae) from southeastern Arizona. Journal of the Lepidopterists’ Society 64(2): 98-102.

Ferris, C.D. and G. Mironov. 2007. Replacement name for *Eupithecia deserticola* (Lepidoptera: Geometridae: Eupitheciini). Canadian Entomologist 139: 131-132.

Ferris, C.D. and J.S. Nordin. 2002. A new species of *Glaucina hulst* from Wyoming and Colorado, and description of the female of *G. nephos* Rindge (Lepidoptera: Geometridae). The Pan-Pacific Entomologist 78(4): 247-254.

Ferris, C.D. and J.S. Nordin. 2004. Taxonomic note on *Sarata tephrella* Ragonot (Lepidoptera: Pyraloidea: Pyralidae: Phycitinae). Zootaxa 569: 1-7.

Ferris, C.D. and P.A. Opler. 2008. A New Species of Eupithecia Curtis (Lepidoptera: Geometridae: Eupitheciini) from Arizona and New Mexico, USA, and Sonora, Mexico. Proceedings of the Entomological Society of Washington 110(1): 87-94.

Ferris, C.D. and J. Russo. 2023a. Review of the *Prorella remorata* group with description of a new species from Arizona (Geometridae: Larentiinae: Eupitheciini). The Journal of the Lepidopterists' Society 77(4): 226-234.

Ferris, C.D. and J. Russo. 2023b. A new *Eupithecia* species from Arizona (Geometridae: Larentiinae: Eupitheciini). The Journal of the Lepidopterists' Society 77(3): 180-182.

Ferris, C.D. and B.C. Schmidt. 2010. Revision of the North American genera *Tetracis* Guenée and synonymization of *Synaxis* Hulst with descriptions of three new species (Lepidoptera: Geometridae: Ennominae). Zootaxa 2347: 1-36.

Ferris, C.D. and B.C. Schmidt. 2011. *Pterospoda nigrescens* (Hulst), a synonym of *Ixala klotsi* Sperry (Lepidoptera, Geometridae, Ennominae). In: B.C. Schmidt and J.D. Lafontaine, (Eds.). Contribution to the systematics of New World macromoths III. ZooKeys 149: 31-37. doi: 10.3897/zookeys.149.2343

Ferris, C.D., J.J. Kruse, J.D. Lafontaine, and K. W. Philip. 2012. A Checklist of the Moths of Alaska. Zootaxa, 3571: 1-25.

Fiance, S.B. and R.E. Moeller. 1977. Immature stages and ecological observations of *Eoparargyractis plevie* (Pyralidae: Nymphalidae). Journal of the Lepidopterists’ Society 31(2): 80-87.

Fibiger, M. and H. Hacker. 2007. Amphipyrinae, Condicinae, Eriopinae, Xyleninae. Noctuidae Europaeae, Vol. 9. Entomological Press, Sorø, Denmark. 410 pp.

Fibiger, M. and J.D. Lafontaine. 2005. A review of the higher classification of the Noctuoidea (Lepidoptera) with special reference to the Holarctic fauna. Esperiana 11: 7-92.

Ficht, G.A. 1940. Notes on Indiana Noctuidae. Proceedings of the Indiana Academy of Science 49: 243-253.

Fiedler, K. 1991. Systematic, evolutionary, and ecological implications of myrmecophily within the Lycaenidae (Insecta: Lepidoptera: Papilionoidea). Bonner Zoologische Monographien 31. 210 pp.

Field, W.D. 1938. A manual of the butterflies and skippers of Kansas (Lepidoptera, Rhopalcocera). Bulletin of the University of Kansas 39(10): 3-302.

Field, W.D. 1971. Butterflies of the genus *Vanessa* and of the resurrected genera *Bassaris* and *Cynthia* (Lepidoptera: Nymphalidae). Smithsonian Contributions to Zoology 84: 1-105.

Fife, L.C. 1939. Insects and a Mite found on Cotton in Puerto Rico, with Notes on their Economic Importance and Natural Enemies. Puerto Rico Experimental Station Bullentin 39. 14pp.

Fink, L.S. 1995. Foodplant effects on the colour morphs of *Eumorpha fasciata* caterpillars (Lepidoptera: Sphingidae). Biological Journal of the Linnean Society of London 56: 423-437.

Finkelstein, L.L. 1980. *Callophrys (Mitoura) hesseli* (Lycaenidae) in Georgia: a state record. Journal of the Lepidopterists’ Society 34(2): 100.

Fitch, H.S. 1965. The University of Kansas Natural History reservation in 1965. University of Kansas Miscellaneous Publications 42: 1-60.

Fitt, G.P. 1989. The ecology of *Heliothis* species in relation to agroecosystems. Annual Review of Entomology 34(1) : 17-53.

Fitzgerald, T.D. 1973. Coexistence of three species of bark-mining *Mamara* (Lepidoptera, Gracillariidae) on green ash and descriptions of new species. Annals of the Entomological Society of America 66(2): 457-464.

Fitzgerald, T.D. 1975. A new species of bark-mining *Marmara* (Lepidoptera: Gracillariidae) from Douglas-fir. Annals of the Entomological Society of America 68(3) : 545-548.

Fitzgerald, T.D. 1995. The Tent Caterpillars. Cornell University Press, Ithaca, New York.

Fitzgerald, T.D. and K.L. Clark. 1994. Analysis of leaf-rolling behavior of *Caloptilia serotinella* (Lepidoptera, Gracillariidae). Journal of Insect Behavior 7(6): 859-872.

Fitzgerald, T.D. and J.B. Simeone. 1971. Serpentine miner *Marmara fraxinicola* (Lepidoptera: Gracillariidae) in stems of white ash. Annals of the Entomological Society of America 64(4) 770-773.

Fitzpatrick, S.M., J.T. Troubridge, and D. Henderson. 2000. *Ochropleura implecta* (Lepidoptera: Noctuidae), a new cutworm pest of cranberries. The Canadian Entomologist 132(3): 365-367.

Fjelddalen, J. 1964. Insect species recorded as new pests on cultivated plants in Norway 1946-62. Norsk Entomologisk Tidsskrift 12: 129-141.

Fleishman, E., G.T. Austin, and D.D. Murphy. 1997. Natural history and biogeography of the butterflies of the Toiyabe Range, Nevada (Lepidoptera: Papilionoidea). Holarctic Lepidoptera, 4(1): 1-18.

Fletcher, D.S. 1954. A revision of the genus *Eubaphe* (Lepidoptera: Geometridae). Zoologica : scientific contributions of the New York Zoological Society 39(13): 153-166, I-II.

Fletcher, T.B. 1921. Life histories of Indian insects, Microlepidoptera. II. Carposinidae, Phaloniadae, Tortricidae and Eucosmidae. Memoirs of the Department of Agriculture in India 6(2): 33-68.

Fletcher, T.B. 1932. Life histories of Indian insects, Microlepidoptera, second series. ALucitidae (Pterophoridae), Tortricina and Gelechiadae.The Imperial Council of Agricultural Research, Scientic Monograph 2 : 1-58.

Florence, P. 2011. New moth (Insecta: Lepidoptera) records from Kingdom Come State Park, Harlan County, Kentucky. Kentucky Naturalist News 69(1): 11-16.

Florida Department of Agriculture and Consumer Services. 2020. Report 91699. <https://www.fdacs.gov/content/download/91699/file/Ento_59_1.xlsx>

Fonseca-Medrano, M. A. Specht, F.A.M. Silva, P. N. Otanásio, and J. V. Malaquias. 2019. The population dynamics of three polyphagous owlet moths (Lepidoptera: Noctuidae) and the influence of meteorological factors and ENSO on them. Revista Brasileira de Entomologia, 63(4): 308-315.

Fontaine, A.R., N. Olsen, R.A. Ring, and S.J. Singla. 1991. Cuticular netal hardening of mouthparts and claws of some forest insects of British Columbia. Journal of the Entomological Society of British Columbia 88: 45-55.

Forbes, W.T.M. 1913. An artificial table of the species of *Hadena*, etc., of Eastern North America, north of the Carolinas. Journal of the New York Entomological Society 21(3): 179-186.

Forbes, W.T.M. 1917. The genera of Hydriomeninæ of the United States (Lep.). Journal of the New York Entomological Society 25(1): 44-67.

Forbes, W.T.M. 1920. Notes on the Crambinae (Lepidoptera). Journal of the New York Entomological Society 28(3/4): 214-227.

Forbes, W.T.M. 1923. Lepidoptera of New York and neighboring states. Part I. Primitive forms, Microlepidoptera, Pyraloids, Bombyces. Memoirs, Cornell University Agricultural Experimental Station, No. 68.

Forbes, W.T.M. 1925. Records of rare Lepidoptera from Ithaca, New York. Canadian Entomologist 57(1): 1-5.

Forbes, W.T.M. 1926. Order Lepidoptera. Pp. 532-687 In: M.D. Leonard (ed.). A List of the insects of New York : with a list of the spiders and certain other allied groups. Cornell University Agricultural Experimental Station Memoir 101.

Forbes, W.T.M. 1930. Insects of Porto Rico and the Virgin Islands. Heterocera or Moths (Excepting Noctuidae, Geometridae and Pyralidae). Scientific Survey of Porto Rico and the Virgin Islands, New York Academy of Sciences XII(1): 1-171 plus 2 plates.

Forbes, W.T.M. 1931. Supplementary report of the Heterocera or moths of Porto Rico.The Journal of the Department of Agriculture of Porto Rico 15(4): 339-393.

Forbes, W.T.M. 1932. The *rubidella* group of *Aristotelia* (Lepidoptera, Gelechildae). Journal of the New York Entomological Society 40(4): 423-433.

Forbes, W.T.M. 1936. The *Cirphis pseudargyria* complex (Lepidoptera: Noctuidæ). Journal of the New York Entomological Society 64: 239-247.

Forbes, W.T.M. 1937. A new "silver-marked" *Eucosma* (Lepidoptera-Olethreutinæ). Journal of the New York Entomological Society 45(1): 131-132

Forbes, W.T.M. 1939. The Lepidoptera of Barro Colorado Island, Panama. Bulletin of the Museum of Comparative Zoology at Harvard College 85(4):III-VII, 99-322, plus 6 plates.

Forbes, W.T.M. 1941. The Lepidoptera of the Dry Tortugas. Psyche 48: 147-148.

Forbes, W.T.M. 1942. The Lepidoptera of Barro Colorado Isalnd, Panama. No. 2. Bulletin of the Museum of Comparative Zoology 90(2): 262-406 plus plates.

Forbes, W.T.M. 1948. Lepidoptera of New York and neighboring states. Part II. Geometridae, Notodontidae, Sphingidae, Lymantriidae. Memoirs, Cornell University Agricultural Experimental Station, No. 274.

Forbes, W.T.M. 1954. Lepidoptera of New York and neighboring states. Part III. Noctuidae. Memoirs, Cornell University Agricultural Experimental Station, No. 329.

Forbes, W.T.M. 1960. Lepidoptera of New York and neighboring states. Part IV. Agaristidae through Nympalidae Including Butterflies. Memoirs, Cornell University Agricultural Experimental Station, No. 371.

Ford, L. T. 1949. A guide to the smaller British Lepidoptera. South London Entomological and Natural History Society, London, England. 230 pp.

Formentini, A.C., D.R. Sosa-Gómez, S.V. de Paula-Moraes, N.M. de Barros, and A. Specht. 2015. Lepidoptera (Insecta) associated with soybean in Argentina, Brazil, Chile and Uruguay. Ciência Rural 45(12): 2113-2120.

Forster W. and T.A. Wohlfahrt. 1971. Die Schmetterlinge Mitteleuropas. Eulen (Noctuidae). W. Keller and Co., Stuttgart.

Foster, S.P. and A.J. Howard. 1998. Influence of stimuli from *Camellia japonica* on oviposition behavior of generalist herbivore *Epiphyas postvittana*. Journal of Chemical Ecology 24: 1251-1275.

Foster, S.P., B.D. Morris, L.D. Charlet, T. Gross, and S. Grugel. 2003. Identification of the sex pheromone of *Cochylis arthuri* (Lepidoptera: Cochylidae). The Canadian Entomologist 135(5): 713-719.

Fothergill, K., M.G. Smart, and P.E. Koenig. 2012. *Cisthene kentuckiensis* (Dyar) (Lepidoptera: Erebidae: Arctiinae): First record for Missouri. Midsouth Entomologist 5: 6-8.

Fox, B. W. 2004. A study of the guild of Lepidoptera foraging on honeysuckle, *Lonicera periclymenum* L. Entomologist’s Gazette 55: 35-43.

Franclemont, J. G. 1939. A revision of the American species of the genus Enargia Hubner (Lepidoptera, Phalaenidae, Amphipyrinae). The Canadian Entomologist 71(5): 113-116.

Franclemont, J.G. 1941a. The Pulverulenta group of the genus *Pseudanarta* Grote (Lepidoptera, Phalaenidae, Cuculliinae). The Canadian Entomologist 73(7): 132-136.

Franclemont, J.G. 1941b. Some new noctuids from New York state with notes on other well known species (Lepidoptera, Phalaenidae, Amphipyrinae). The Canadian Entomologist 73(6): 111-114.

Franclemont, J.G. 1946. A revision of the species of Symmerista Hübner known to occur north of the Mexican border (Lepidoptera, Notodontidae). The Canadian Entomologist, 78(5) : 96-103.

Franclemont, J.G. 1948. S*ymmerista*. pp 222-224 in W.T.M. Forbes, Lepidoptera of New York and neighboring states. Part II. Memoirs, Cornell University Agricultural Experimental Station, No. 274.

Franclemont, J.G. 1951. The species of the *Leucania unipuncta* group, with a discussion of the generic names for the various segregates of *Leucania* in North America. Proceedings of the Entomological Society of Washington 53 : 57-85.

Franclemont, J.G. 1966. Two new species of Arctiidae from southern Arizona (Lepidoptera, Arctiidae, Arctiinae). Proceedings of the Entomological Society of Washington 68: 250-257.

Franclemont, J.G. 1966b. A new genus *Nychioptera* with descriptions of 2 new species and notes on genus *Hemeroplanis* (Lepidoptera-Noctuidae). Proceedings of the Entomological Society of Washington 68(4): 295-306.

Franclemont, J.G. 1968. A new species of *Metaxaglaea* (Lepidoptera, Noctuidae, Cuculliinae). Entomological News 79(3): 75-63.

Franclemont, J.G. 1969. Two new species of Lithophane from California (Noctuidae, Cuculliinae). Journal of the Lepidopterists’ Society 23: 10-14.

Franclemont, J.G. 1973a. Mimallonoidea, Mimallonidae and Bombycoidea, Apatelodidae, Bombycidae, Lasiocampidae. In R. B. Dominick et al. (eds.). The Moths of America North of Mexico. Fasc. 20.1. E.W. Classey Ltd. and Richard B. Dominick Publicatiosn, London, United Kingdom. 86 p.

Franclemont, J.G. 1973b. A new noctuid from Arizona (Lepidoptera: Noctuidae: Cuculliinae). Proceedings of the Entomological Society of Washington 75(2): 172-176.

Franclemont, J.G. 1976. New species of Arizona noctuids, 1: (Lepidoptera: Noctuidae: Hadeninae). Proceedings of the Entomological Society of Washington 78: 409-416.

Franclemont, J.G. 1983. Arctiidae. pp. 114-119 In R.W. Hodges et al. (eds.). Check list of the Lepidoptera of America north of Mexico. E. W. Classey Ltd. & Wedge Entomological Research Foundation, London.

Franclemont, J.G. 1985a. A new species of *Parascotia* with notes on the genera *Mycterophora* and *Parascotia* (Lepidoptera: Noctuidae: Incertae sedis). Proceedings of the Entomological Society of Washington 87(4): 826-833.

Franclemont, J.G. 1985b. A new species of *Meganola* Dyar from eastern North America (Lepidoptera: Noctuidae: Nolinae). Proceedings of the Entomological Society of Washington 87(4): 871-874.

Franclemont, J.G. 1986a. New species and new genera of noctuids from Arizona, Texas, and Mexico: the genera *Matigramma* and *Acitogramma* (Lepidoptera: Noctuidae: Catocalinae). Journal of the Kansas Entomological Society 59: 143-172.

Franclemont, J.G. 1986b. New species of tortricid moths from eastern North America (Lepidoptera: Tortricidae). Proceedings of the Entomological Society of Washington 88(1): 56-62.

Franclemont, J.G. and E.L. Todd. 1983. Noctuidae. pp. 120-159 In: R.W. Hodges, T. Dominick, D.R. Davis, D.C. Ferguson, J.G. Franclemont, E.G. Munroe, and J.A. Powell (eds.) Check List of the Lepidoptera of America North of Mexico. Fasc. 24. The Wedge Entomological Research Foundation, EW Classey Ltd, London and Washington.

Frank, J.H. and E.D. McCoy. 1992. The immigration of insects to Florida, with a tabulation of records published since 1970. Florida Entomologist 75(1): 1-28.

Frank, J.H. and E.D. McCoy. 1993. Introduction to the behavioral ecology of introduction. The introduction of insects into Florida. The Florida Entomologist 76(1): 1-53.

Franklin, R.T. and R.N. Coulson. 1968. Insects affecting seed production of shortleaf pine in the Georgia Piedmont. Canadian Entomologist 100: 807-813.

Freeman, H.A. 1964. Four new species of *Agathymus* from Texas (Mecathymidae). Journal of the Lepidopterists’ Society 18 (3): 171-185.

Freeman, H.A. 1967d. Three new species of Hesperiidae from Mexico. Journal of the Lepidopterists' Society 21: 115-119.

Freeman, H.A. 1969a. Records, new species, and a new genus of Hesperiidae from Mexico. Journal of the Lepidopterists' Society 23(supplement 2): 1-62.

Freeman, H.A. 1969b. Systematic review of the Megathymidae. Journal of the Lepidopterists' Society, 23 (Supplement 1): 1-59

Freeman, H.A. 1993. Notes on *Amblyscirtes* Scudder, with the description of two new subspecies (Insecta: Lepidoptera: Hesperiidae: Hesperiinae). Annals of the Carnegie Museum 62(4): 341-350.

Freeman, T.N. 1940. Two apparently new Canadian species of *Sparganothis.* Canadian Entomologist 72: 206-208.

Freeman, T.N. 1944. A review of the North American species of the genus *Argyrotaenia* Stephens, (Lepidoptera, Tortricidae). Scientific Agriculture 25: 81-95.

Freeman, T.N. 1953. The Spruce Budworm, *Choristoneura fumiferana* (Clemens) and an allied new species on pine (Lepidoptera: Tortricidae). Canadian Entomologist 85: 121-152.

Freeman, T.N. 1958. The Archipinae of North America (Lepidoptera: Tortricidae). Canadian Entomologist 90(7): 1-89.

Freeman, T.N. 1960. Needle-mining Lepidoptera of pine in North America. The Canadian Entomologist 92(Supplement 16): 1-51.

Freeman, T.N. 1962. A new species of *Eucordylea* from spruce (Lepidoptera: Gelechiidae). Canadian Entomologist 94: 1029-1031.

Freeman, T.N. 1962b. A new *Laspeyresia* species on red pine. Canadian Entomologist 94: 1272-1273.

Freeman, T.N. 1963. Two new species of coniferous needle miners from Louisiana and the description of a new genus (Lepidoptera: Gelechiidae). The Canadian Entomologist 95(7): 727-730.

Freeman, T.N. 1965. A new species of *Parapandemis* Obraztsov, a genus new to Canada (Lepidoptera: Tortricidae). The Canadian Entomologist 97(10): 1114-1116.

Freeman, T.N. 1965b. New Canadian species of leaf-mining Lepidoptera of conifers. Journal of Research on the Lepidoptera 4(3): 209-220.

Freeman, T.N. 1966a. A new species of *Epinotia* Hubner from British Columbia (Olethreutidae). Journal of Research on the Lepidoptera 5: 13-14.

Freeman, T.N. 1966b. [1967]. A new species of *Nepticula* on bur oak in Ontario (Nepticulidae). Journal of Research on the Lepidoptera 6: 19-21.

Freeman, T.N. 1967a. Annotated keys to some Nearctic leaf-mining Lepidoptera on conifers. Canadian Entomologist 99: 419-435.

Freeman, T.N. 1967b. On coniferophagous species of *Choristoneura* (Lepidoptera: Tortricidae) in North America. Canadian Entomologist 99: 449-455.

Freeman, T.N. 1967c. A new Nearctic species o*f Exoteleia* Wallengren (Gelechiidae) on pine. Journal of the Lepidopterists' Society 21(1): 9-11.

Freeman, T.N. 1970a. A new species of Cameraria on bur oak in Manitoba (Gracillaridae). Journal of the Lepidopterists' Society 24: 86-87.

Freeman, T.N. 1972. The coniferous feeding species of *Argyresthia* in Canada (Lepidoptera: Yponomeutidae). The Canadian Entomologist 104(05): 687-697.

Frey, H. and J. Boll. 1876. Einige Tineen aus Texas. Entomologische Zeitung zu Stettin 37: 209-228.

Frey, H. and J. Boll. 1878. Tineen aus Texas. Entomologische Zeitung, Stettin 39(7-9): 249-279.

Frick, K.E. and R.B. Hawkes. 1970. Additional insects that feed upon tansy ragwort, *Senecio jacobaea*, an introduced weedy plant, in the western United States. Annals of the Entomological Society of America 63: 1085-1090.

Friedman, M.A. 2016. Host choices of *Celastrina neglecta* (W. H. Edwards) (Lepidoptera: Lycaenidae: Polyommatinae) in Okaloosa County, Florida. Southern Lepidopterists’ News 38(2): 18-21.

Friedman, M.A., D.M. Wright, and M.C. Minno. 2019. New host plant record for *Celastrina neglecta* (W. H. Edwards) (Lepidoptera: Lycaenidae: Polyommatinae) in Okaloosa County, Florida. News of the Lepidopterists’ Society 61(1): 6-7.

Frielander, T.P. 1986 [1988]. Taxonomy, phylogeny and biogeography of *Asterocampa* Rüber 1916. Journal of Research on Lepidoptera 25(4): 215-338.

Fronza, E., A. Specht, and E. Corseuil. 2011. Butterflies and moths (Insecta: Lepidoptera) associated with erva-mate, the South American Holly (*Ilex paraguariensis* St. Hil.), in Rio Grande do Sul, Brazil. Check List 7(4): 496-504.

Frost, S.W. 1969. Supplement to Florida insects taken in light traps. The Florida Entomologist 2(2): 91-101.

Frost, S.W. 1972. Notes on *Urodus parvula* (Henry Edwards) (Yponomeutidae). Journal of the Lepidopterists’ Society 26(3): 173-177.

Frost, S.W. 1975. Third supplement to insects taken in light traps at the Archbold Biological Station, Highlands County, Florida. The Florida Entomologist 58(1): 35-42.

FTN Associates, Ltd. 2007. Lepidoptera and Odonata Survey of Fort Chaffee Maneuver Training Center, Arkansas. Prepared for Fort Chaffee Maneuver Training Center Environmental Branch, Fort Chaffee, Arkansas.

Fuentes, E.G., E. Hernández-Suárez, O. Simón, T. Williams, and P. Caballero. 2018. *Chrysodeixis chalcites*, a pest of banana crops on the Canary Islands: Incidence, economic losses and current control measures. Crop Protection 108: 137-145.

Funakoshi, S. 2008. Tortricid moths feeding on *Quercus glauca* Thunb. Japan Heterocerists’ Journal 247: 399-401.

Fung, J. and G.S. Wheeler. 2016. Life history and host range of *Oxydia vesulia* transpeneus, an unsuitable biological control agent of Brazilian Peppertree. Biocontrol Science and Technology 26(3): 298-304.

Furneaux, W. 1897. Butterflies and moths (British). Longmans, Green & Co., London, England.

Furniss, M.M. and W.F. Barr. 1975. Insects Affecting Important Native Shrubs of the Northwestern United States. Idaho Agriculture Experimental Station Research Paper 7466. 65 pp.

Furniss, M.M., D.C. Ferguson, K.W. Voget, J.W. Burkhardt, A.R. Tiedemann, and J.L. Oldemeyer.1988. Taxonomy, Life History, and Ecology of a Mountain-mahogany Defoliator, *Stamnodes animata* (Pearsall), in Nevada. United States Department of the Interior Fish and Wildlife Service, Fish and Wildlife Research 3. 25 pp.

Furniss, R.L. and V.M. Carolin, 1977. Western Forest Insects. U.S. Department of Agriculture, Forest Service, Miscellaneous Publication No. 1339: 1-653.

Gabel, B., F. Marion-Poll, V. Suchy, R. Roehrich, P. Hradsky, and D. Thiery. 1994. Olfactory responses of *Lobesia botrana* females (Lepidoptera: Tortricidae) to *Tanacetum vulgare* (Asteraceae) flower extracts and fractions. Entomological Problems 25(1): 1-7.

Gaedike, R. 1977. Revision der Nearktischen und Neotropischen Epermeniidae (Lepidoptera). Beitrage Zur Entomologie 27: 301-312.

Gaedike, R. 1984. Revision der Nearktischen und Neotropischen Acrolepiidae (Lepidoptera) [Revision of the Nearctic and Neotropical Acrolepiidae (Lepidoptera)]. Entomologische Abhandlungen 47(10): 179-194.

Gaedike, R. 1990. Revision der Nearktischen Douglasiidae (Lepidoptera). Beitrage Zur Entomologie 40(2): 287-300.

Gaedike, R. 1994. A new species of *Acrolepiopsis* and the description of the female of *A. californica* (Acrolepiidae). Journal of the Lepidopterists’ Society 48(1): 46-50.

Gaedike, R. 2008. New species and records of the Nearctic Epermeniidae (Lepidoptera). Tijdschrift voor Entomologie 151(1): 57-64.

Gaedike, R. 2009. Some new and interesting “Microlepidoptera” from the collection of the Zoologisches Forschungsmuseum Alexander Koenig (ZFMK), Bonn (Lepidoptera: Tineidae, Epermeniidae; Acrolepiidae; Douglasiidae). Bonner Zoologische Beiträge 56: 101-106.

Gaedike, R. 2010. New and poorly known Palaearctic Microlepidoptera. Nota Lepidopterologica 33(1): 9-24.

Gaedike, R. 2018. New or poorly known Douglasiidae from the Palaearctics (Lepidoptera: Douglasiidae). SHILAP Revista de Lepidopterología 46(181): 57-63.

Gaither, J.C. and J. De Bendictis. 1993. Verification of the occurrence of *Lotisma trigonana* (Copromorphidae) in Alaska. Journal of the Lepidopterists' Society 47(4): 328.

Gall, L.F. 1990a. Evolutionary ecology of sympatric *Catocala* moths (Lepidoptera: Noctuidae). III. Experiments on female oviposition preference. Journal of Research on the Lepidoptera 29(3): 217-233.

Gall, L.F. 1990b [1991]. Evolutionary ecology of sympatric *Catocala* moths (Lepidoptera: Noctuidae) II. Sampling for wild larvae on their foodplants. Journal of Research on Lepidoptera, 29(3): 195-216.

Gall, W.K. and R.F. Andrle. 1994. Significant range extension of *Leptotes marina* (Lepidoptera: Lycaenidae) into New York State. The Great Lakes Entomologist 26(4): 319-320.

Gall, L.F. and D.C. Hawks. 1990. Systematics of moths in the genus *Catocala* (Lepidoptera: Noctuidae). I. Type material in the Strecker collection, with lectotype designations. Fieldiana, Zoology, New Series 59(1414): 1-16.

Gall, L.F. and D.C. Hawks. 2010. Systematics of moths in the genus *Catocala* (Lepidoptera, Erebidae) IV. Nomenclatorial stabilization of the Nearctic fauna, with a revised synonymic check list. In: B.C. Schmidt and J.D. Lafontaine, (Eds.). Contributions to the systematics of New World macro-moths II. ZooKeys 39: 37-83.

Gall, L.F. and D.C. Hawks. 2015. Systematics of moths in the genus *Catocala* (Lepidoptera: Noctuidae). V. Neotypification of names in the Nearctic fauna. Bulletin of the Peabody Museum of Natural History 56(1):47-54.

Gall, L.F., J.W. Peacock and S.W. Bullington, 1991. *Catocala* (Noctuidae) taken at Shenandoah National Park, Virginia, with comparative notes on adult flight phenologies in eastern North America. Journal of the Lepidopterists’ Society 45(3): 226-231.

Gall, L.F., J.W. Peacock, and J.R. Slotten. 2002. Life history and immature stages of *Catocala atcala* (Noctuidae). Journal of the Lepidopterists' Society 56(1): 1-4.

Gall, W.K., and R.F. Andrle. 1994. Significant range extension of *Leptotes marina* (Lepidoptera: Lycaenidae) into New York State. The Great Lakes Entomologist 26(4): 319-320.

Gambino, P. 1995. *Dolichovespula (*Hymenoptera: Vespidae), hosts of *Aphomia sociell*a (L.) (Lepidoptera: Pyralidae). Journal of the New York Entomological Society 103(2): 165-169.

García, A., H. Valdes, and N. Triguero. 1990. Anomis illita (Lepidoptera, Noctuidae), an important defoliator of *Hibiscus elatus* (majagua). Revista Forestal Baracoa 20(2); 7-17.

Gardiner, B.O.C. 1982a. A silkmoth rearer’s handbook. Third edition. Amateur Entomologist 12: 1-255.

Gardiner, B.O.C. 1982b. *Cacoecimorpha pronubana* Hnb. (Lep.: Tortricidae) successfully reared on artificial diet, with a note on its diapause requirements. Entomologist’s Record and Journal of Variation 94: 122-123.

Garraway, E., J.A. Bailey, T. Farr, and J. Woodley. 1993. Studies on the Jamaican Kite Swallowtail, E*urytides* (*Protesilaus*) *marcellinus* (Lepidoptera: Papilionidae). Tropical Lepidoptera Research 4(2): 151-154.

Garth, J.S. and J.W. Tilden. 1986. California Butterflies. University of California, Berkeley, California.

Garzón-Orduña, I.J., A.V.Z. Brower, M. Kamilari, Amaia Iribar, and J. Murienne. 2018. Cracking the code: examination of species delimitations amon*g Hamadryas* butterflies with DNA barcodes suggests Caribbean Cracker is *Hamadryas februa* Hübner (Nymphalidae: Biblidinae)." The Journal of the Lepidopterists' Society 72(1): 53-73.

Gaskin, D.E. 1986. Genus *Diptychophora* Zeller and a related new genus *Steneromene* from the Neotropical region (Pyralidae: Crambinae). Journal of the Lepidopterists' Society 40: 107-123.

Gassmann, A. 1999. Final report, the biological control of leafy spurge (*Euphorbia esula* L.) in North America Work done in 1994-1998. CABI Bioscience, A Division of CAB International, Centre, Switzerland. 48 pp.

Gates, M.W., J.M. Heraty, M.E. Schauff, D.L. Wagner, J.B. Whitfield, and D.B. Wahl. 2002. Survey of the parasitic Hymenoptera on leafminers in California. Journal of Hymenoptera Research 11(2): 213-270.

Gates, M.W., J.T. Lill, R.R. Kula, J.E. O'Hara, D.B. Wahl, D.R. Smith, J.B. Whitfield, S.M. Murphy, and T.M. Stoepler. 2012. Review of parasitoid wasps and flies (Hymenoptera, Diptera) associated with Limacodidae (Lepidoptera) in North America, with a key to genera. Proceedings of the Entomological Society of Washington 114(1): 24-110.

Gatrelle, R.R. 1998. The rediscovery, taxonomy, and biology of *Chlosyne gorgone* gorgone and *Chlosyne ismeria* (Nymphalidae) in Burke County, Georgia. The Taxonomic Report of the International Lepidoptera Survey 1(2): 1-9.

Gatrelle, R.R. 1999. Hübner’s *Helicta*: The forgotten *Neonympha*. The recognition and elevation of *Neonympha* Helicta (Nymphalidae: Satyrinae) to specific status. The designation of neotypes for *N. helicta and N. areolatus*. The subspecific transfer of *septentrionalis* to *Helicta* and the description of a third *Helicta* subspecies from south Florida. The Taxonomic Report of the International Lepidoptera Survey. 18): 1-8.

Gatrelle, R.R. 2004. Description of a multilevel cryptic new species of *Phyciodes* (Nymphalidae: Melitaeinae) from the southern Appalachian Mountains. The Taxonomic Report of the International Lepidoptera Survey. 4(8): 1-19.

Gatrelle, R., J. Coffman and B. Cavanaugh. 1983. Current zone reports, Zone V, Virgina, North & South Carolina. Southern Lepidopterists' News 4: 25-26.

Gautam, S.G., G.P. Opit, and E. Hosoda. 2016. Phosphine resistance in adult and immature life stages of *Tribolium castaneum* (Coleoptera: Tenebrionidae) and *Plodia interpunctella* (Lepidoptera: Pyralidae) populations in California. Journal of Economic Entomology 109(6): 2525-2533.

Gayton, D. and V. Miller. 2012. Impact of biological control on two knapweed species in British Columbia. Journal of Ecosystems and Management 13(3): 1-14.

GBIF.org (24 April 2025) GBIF Occurrence Download for *Scythropiodes issikii*. https://doi.org/10.15468/dl.bh3jhz.

Genoways, H.H. and F.J. Brenner. 1985. Species of Concern in Pennsylvania. Special publication (Carnegie Museum of Natural History) 11: 1-430.

Gerlach, J. and P. Matyot. 2006. Lepidoptera of the Seychelles islands. Backhuys Publishers, Leiden, The Netherlands.

Ghesquière J. 1940. Lépidoptères Microlépidoptères (première partie). Annales du Musée du Congo belge, Zoologie [3, Arthropodes] section 2, Catalogues raisonnés 7(1): 1-120.

Gibson, A. 1900. The life-history of *Arctia phalerata* Harr. The Canadian Entomologist 32: 369-376.

Gibson, G.A. 2011. The species of *Eupelmus* (*Eupelmus*) Dalman and *Eupelmus* (*Episolindelia*) Girault (Hymenoptera: Eupelmidae) in North America north of Mexico. Zootaxa 2951: 1-97.

Gibson, L.D. and C.V. Covell, Jr. 2006. New records of butterflies and moths (Lepidoptera) from Kentucky. Journal of the Kentucky Academy of Science 67(1): 19-21.

Gibson, L.D. and J.A. Merkle. 2006. Discovery of a larval host plant for *Olethreutes monetiferana* (Riley) (Tortricidae) in northern Kentucky. Journal of the Lepidopterists’ Society 61(2): 113.

Gibson, L.D. and W.E. Miller. 1994. Two new synonymies in Nearctic *Eucosma* (Tortricidae: Olethreutinae). Journal of The Lepidopterists' Society 48: 69-71.

Gielis, C. 1993. Generic revision of the superfamily Pterophoroidea (Lepidoptera). Zoologische Verhandelingen 290: 1-139.

Gielis, C. 2003. World Catalogue of Insects, vol. 4: Pterophoroidea & Alucitoidea Lepidoptera). Apollo Books, Stenstrup, Denmark. 198 pp.

Gielis, C. 2006. Review of the Neotropical species of the family Pterophoridae, part I: Ochyroticinae, Deuterocopinae, Pterophorinae (Platyptiliini, Exelastini, Oxyptilini) (Lepidoptera). Zoologische Mededelingen 80(1): 1-290.

Gielis, C. 2008. Additions to the species complex *Paraplatyptilia auriga* (Barnes & Lindsey, 1921) in the USA (Lepidoptera: Pterophoridae). Entomologische Zeitschrift 118(1): 3-7.

Gielis, C. 2011. Review of the Neotropical species of the family Pterophoridae, part 2: Pterophorinae (Oidaematophorini, Pterophorini) (Lepidoptera). Zoologische Mededelingen 85: 589-824.

Gielis, C. 2025. Catalogue of the Pterophoroidea of the World, version 1.1.25.33. (02 Feb 2025). https://pterophoroidea.hobern.net. [Accessed Feb 2025].

Gifford, S.M. and P.A. Opler. 1983. Natural history of seven hairstreaks in coastal North Carolina. Journal of the Lepidopterists' Society 37(2): 97-105.

Gill, R.J. 2001. New states records. The California Plant Pest and Disease Report 19(3-6): 25-31.

Gillespie, D.R. and B.I. Gillespie. 1982. A list of plant-feeding Lepidoptera introduced into British Columbia. Journal of the Entomological Society of British Columbia 79: 38-48.

Gillette, C.P. 1898. Colorado Lepidoptera. Colorado State Agriculture College Experimental Station Bulletin 43: 3-31.

Gilligan, T.M., and J.W. Brown. 2016. A new genus for *Tortrix druana* Walsingham, 1914 and a new species from the northern Neotropics (Lepidoptera: Tortricidae: Cochylini: Euliina). The Journal of the Lepidopterists' Society 70(2): 139-144.

Gilligan, T.M. and M.E. Epstein. 2014. Tortricids of Agricultural Importance. <http://idtools.org/id/leps/tortai/index.html>.

Gilligan, T.M. and S.C. Passoa. 2014. Crambidae - *Maruca vitrata* (Fabricius), LepIntercept - An identification resource for intercepted Lepidoptera larvae. USDA-APHIS-PPQ Identification Technology Program (ITP). Fort Collins, CO. Last updated February 2014. <http://idtools.org/id/leps/lepintercept/vitrata.html> [accessed 15 March 2019].

Gilligan, T.M. and D.J. Wright. 2013a. Revised world catalogue of Eucopina, Eucosma, Pelochrista, and Phaneta (Lepidoptera: Tortricidae: Eucosmini). Zootaxa 3746(2): 301-337.

Gilligan, T.M. and D.J. Wright. 2013b. The type species of *Eucosma* Hübner (Lepidoptera: Tortricidae: Eucosmini). Zootaxa 3630(3): 489-504

Gilligan, T.M., D.J. Wright, and L.D. Gibson. 2008. Olethreutine moths of the Midwestern United States: An Identification Guide. Ohio Biological Survey, Columbus, Ohio. New Series 16: 1-334.

Gilligan, T.M., T. Harrison, and L.D. Gibson. 2009. Rediscovery and redescription of *Hystrichophora loricana* (Grote) (Tortricidae: Olethreutinae). Zootaxa 2117: 65-68.

Gilligan, T.M., J.W. Brown, and M.S. Hoddle. 2011a. A new avocado pest in Central America (Lepidoptera: Tortricidae) with a key to Lepidoptera larvae threatening avocados in California. Zootaxa 3137: 31-45.

Gilligan, T.M., M.E. Epstein, S.C. Passoa, J.A. Powell, O.C. Sage, and J.W. Brown. 2011b. Discovery of *Lobesia botrana* ([Denis & Schiffermüller]) in California: An Invasive Species New to North America (Lepidoptera: Tortricidae). Proceedings of the Entomological Society of Washington 113(1): 14-30.

Gilligan, T.M., M. E. Epstein, and K.M. Hoffman. 2011. Discovery of false codling moth, *Thaumatotibia leucotreta* (Meyrick), in California (Lepidoptera: Tortricidae). Proceedings of the Entomological Society of Washington 113(4): 426-435.

Gilligan, T.M., J. Baixeras, J.W. Brown, and K.R. Tuck. 2012. T@RTS: Online World Catalogue of the Tortricidae (Ver. 2.0). <http://www.tortricid.net/catalogue.asp>.

Gilligan, T.M., J. Baixeras, J.W. Brown, and K.R. Tuck. 2014a. T@RTS: Online World Catalogue of the Tortricidae (Ver. 3.0). <http://www.tortricidae.com/catalogueSearch.asp>

Gilligan, T.M., D.J. Wright, J. Munz, K. Yakobson, and M.P. Simmons. 2014b. Molecular phylogeny and revised classification of *Eucosma* Hübner and related genera (Lepidoptera: Tortricidae: Eucosmini). Systematic Entomology 39(1): 49-67.

Gilligan, T.M., P. Huemer, and B. Weismair. 2016. Different continents, same species? Resolving the taxonomy of some Holarctic *Ancylis* Hübner (Lepidoptera: Tortricidae). Zootaxa 4178(3): 347-370.

Gilligan, T. M., J. Baixeras, and J. W. Brown. 2018. T@RTS: Online World Catalogue of the Tortricidae (Ver. 4.0). http://www.tortricid.net/catalogue.asp.

Gilligan, T.M., J.W. Brown, and J. Baixeras. 2020a. Immigrant Tortricidae: Holarctic versus introduced species in North America. Insects 11(9): 594; 1-59.

Gilligan, T.M., D.J. Wright, R.L. Brown, B.A. Augustinus, and U. Schaffner. 2020b. Taxonomic issues related to biological control prospects for the ragweed borer, *Epiblema strenuana* (Lepidoptera: Tortricidae). Zootaxa 4729(3): 347-358.

Girault, A.A. 1916. Descriptions of and observations on some chalcidoid Hymenoptera II. The Canadian Entomologist 48: 265-272.

Glassberg, J. 2001. Butterflies through Binoculars: The West: A Field Guide to the Butterflies Western North America. Oxford University Press,

Glassberg, J., 2017a. A Swift Guide to butterflies of North America. Princeton University Press, Princeton, New Jersey.

Glassberg, J., 2017b. A Swift Guide to Butterflies of Mexico and Central America: Second Edition. Princeton University Press, Princeton, New Jersey.

Glenn, R.A. 1941. The genus *Arugisa* in the United States, with the description of a new species (Lepidoptera, Phalaenidae). American Museum Novitates 1114: 1-4.

Glime, J. M. 2017. Terrestrial Insects: Holometabola - Lepidoptera: Micropterigoidea - Gelechioidea. Chapt. 12-13. In: J.M. Glime. Bryophyte Ecology. Volume 2. Bryological Interaction. Ebook sponsored by Michigan Technological University and the International Association of Bryologists. Last updated 21 April 2017 and available at <http://digitalcommons.mtu.edu/bryophyte-ecology2/>

Glime, J.M. 2021. Terrestrial Insects: Holometabola - Lepidoptera: Micropterigoidea - Gelechioidea. Chapter 12 In: J.M. Glime. Bryophyte Ecology. Volume 2. Bryological Interaction. Ebook sponsored by Michigan Technological University and the International Association of Bryologists. Last updated 16 April 2021 and available at <http://digitalcommons.mtu.edu/bryophyte-ecology2/>.

GMNH-UGCA 2015. University of Georgia Collection of Arthropods, University of Georgia, Athens, Georgia. In: SCAN. P.L. Heinrich, E. Gilbert, N.S. Cobb, and N. Franz. Symbiota collections of arthropods network (SCAN): A data portal built to visualize, manipulate, and export species occurrences. [Dataset]. http://scan-bugs.org/portal/collections/index.php [Accessed 08 March 2019]

Gochfeld, M. and J. Burger, 1997. Butterflies of New Jersey. A Guide to their Status, Distribution, Conservation and Appreciation. Rutgers Univ. Press, New Brunswick, NJ.

Godfrey, G.L. 1971. The larvae of *Lithacodia muscosula*, *L. carneola*, and *Neoerastria apicosa*. Journal of the Kansas Entomological Society 44(3): 390-397.

Godfrey, G.L. 1972a. A Review and Reclassification of the Larvae of the Subfamily Hadenina (Lepidoptera, Noctuidae) of America North of Mexico. United States Department of Agriculture Technical Bulletin no. 1450: 1-265. U.S. Government Printing Office, Washington, DC.

Godfrey, G.L. 1972b. Tha last instar caterpillars of *Oxycilla tripla* Grote and *Colobochyla interpuncta* (Grote) (Lepidoptera: Noctuidae). Proceedings of the Entomological Society of Washington 74(1): 78-85.

Godfrey, G.L. 1972c. The last larval instar of *Lithacodia albidula* (Lepidoptera: Noctuidae). Journal of the Kansas Entomological Society 45(4): 427-430.

Godfrey, G.L. 1980. Larval descriptions of *Renia hutsoni*, *Renia rigida,* and *Renia mortualis* with a key to larvae of *Renia* (Lepidoptera, Noctuidae). Proceedings of the Entomological Society of Washington 82(3): 457-468.

Godfrey, G.L. 1981a. An *Oxalis* (Oxalidaceae) feeding larva, *Galgula partita* (Noctuidae). Journal of the Lepidopterists’ Society 35(2): 132-136.

Godfrey, G.L. 1981b. Identification and descriptions of the ultimate instar larvae of Hydraecia immanis (hop vine borer) and H. micacea (potato stem borer) (Lepidoptera: Noctuidae). Biological Notes, Illinois Natural History Survey 114: 1-8.

Godfrey, G.L. 1984. Notes on the larva *Cargida pyrrha* (Notodontidae). Journal of the Lepidopterists’ Society 38(2): 88-91.

Godfrey, G.L., E.D. Cashatt, and M.O. Glenn. 1987. Microlepidoptera from the Sandy Creek and Illinois River Region: An annotated checklist of the suborders Dacnonypha, Monotrysia, and Ditrysia (in part) (Insecta). Illinois Natural History Survey Special Publication 7: 1-44.

Godman, F.D. and O. Salvin. 1879-1901. Insecta. Lepidoptera-Rhopalocera. Volume I. Biologia Centralii-Americana. Entomological Society of London. Taylor & Francis, London, England.

Godman, F.D and O. Salvin. 1901. Insecta. Lepidoptera-Rhopalocera. Volume. II. Biologia Centrali-Americana. Entomological Society of London. Taylor & Francis, London, England.

Goeden, R.D. 1971. Insect ecology of silverleaf nightshade. Weed Science 19(1): 45-51.

Goeden, R.D., D.W. Ricker, and R.B. Hawkes. 1978. Establishment of *Coleophora parthenica* (Lep.: Coleophoridae) in southern California for the biological control of Russian thistle. Environmental Entomology 7(2): 294-296.

Gohle, K.B. 1963. Diurnal and nocturnal Lepidoptera of Bay Ridge Waterfront. Journal of the New York Entomological Society 71(3): 148-157.

Goldstein, P.Z. and M.W. Nelson. 2017. Two psammophilic noctuids newly associated with beach plum, *Prunus maritima* (Rosaceae): The Dune Noctuid (*Sympistis riparia*) and Coastal Heathland Cutworm (*Abagrotis benjamini*) in Northeastern North America (Lepidoptera, Noctuidae). ZooKeys 661: 61-89. <https://doi.org/10.3897/zookeys.661.10867>.

Goldstein, P.Z. and E. Quinter. 2003. Host plant associations of the western species of Papaipema (Noctuidae) with particular reference to the apiaceous plants. Journal of the Lepidopterists’ Society 57(2): 153-156.

Goldstein, P.Z., M. A. Metz, and M.A. Solis. 2013. Phylogenetic systematics of *Schacontia* Dyar with descriptions of eight new species (Lepidoptera, Crambidae). ZooKeys 291: 27-81. doi: 10.3897/zookeys.291.3744.

Gómez-Nucamendi, O.L., R.W. Jones, and A. Morón-Ríos. 1999.The Sphingidae (Heterocera) of the "El Ocote" Reserve, Chiapas, México. Journal of the Lepidopterists' Society 53(4): 153-158.

Gompert, Z., J.A. Fordyce, M. L. Forister, and C.C. Nice. 2008. Recent colonization and radiation of North American Lycaeides (*Plebejus*) inferred from mtDNA. Molecular Phylogenetics and Evolution 48(2)8): 481-490.

Goodger, K. and P.R. Ackery. 2002. Bates, and the beauty of butterflies. The Linnean 18(1): 21-59, 6 pls.

Goodpasture, C. 1974. Foodplant specificity in the *Plebejus* (*Icaricia*) *acmon* group Lycaenidae). Journal of the Lepidopterists’ Society 28(1): 53-63.

Goodson, R.L. and H.H. Neunzig, 1993. Taxonomic revision of the genera *Homoeosoma* Curtis and *Patagonia* Ragonot (Lepidoptera: Pyralidae: Phycitinae) in America north of Mexico. North Carolina Agricultural Research Service Technical Bulletin 303: 1-105.

Gorbunov, O.G. 2020. Establishment of *Eichlinia* gen.n. for the Western hemisphere Melittiini (Lepidoptera: Sesiidae), with a catalogue of the genus. Russian Entomological Journal 29(3): 276-284.

Grafton-Cardwell, E.E., J.G. Morse, N.V. O’Connell, P.A. Phillips, H.D. Ohr, J.A. Menge, B.B. Westerdahl, C.W. Coggins, T.S. Prather, and D.W. Cudney. 2002. Integrated pest management guide for citrus. University of California Statewide Integrated Pest Management Project, Division of Agriculture and Natural Resources, Publication 3441.

Graham, S.P. 2010.Visitors to southeastern hawkmoth flowers. Southeastern Naturalist 9: 413-426.

Graves, G.R. 2017. First record of *Citheronia regalis* (Lepidoptera: Saturniidae) feeding on *Cotinus obovatus* (Anacardiaceae). Florida Entomologist 100(2): 474-475.

Graves, S.D. and A.M. Shapiro. 2003. Exotics as host plants of the California butterfly fauna. Biological Conservation 110(3): 413-433.

Greene, G.M. 1913. Doings of societies. Entomological News, and Proceedings of the Entomological Section of the Academy of Natural Sciences 23(3): 139-142.

Greeney, H.F. and A.D. Warren. 2003. Notes on the life history of *Eantis thraso* (Hesperiidae: Pyh.Ginae) in Ecuador. Journal of the Lepidopterists’ Society 57(1): 43-46.

Greenfield, M.D. and M.G. Karandinos. 1995. A new species of *Paranthrene* (Lepidoptera: Sesiidae). Proceedings of the Entomological Society of Washington 81(3): 449-504.

Grefenstette, W., O. El-Lissy, and R.T. Staten. 2009. Pink bollworm eradication plan in the U.S. APHIS USDA Publication. <http://www.aphis.usda.gov/plant_health/plant_pest_info/cotton_pests/downloads/pbw-erad-plan2-08.pdf>.

Grehan, J.R., B.L. Parker, and R.G. Dearborn. 1994. Description of the first and final instar of the hemlock loopers *Lambdina athasaria* (Walker) and Lambdina fiscellaria (Guenée) (Lepidoptera: Geometridae). The Canadian Entomologist 126(06): 1505-1514.

Grehan, J.R., M. Sabourin and P.M. Hanson, 1995a. Maple feeding Tortricidae of the northeastern United States. Guide to identification of adults. Agricultural Experimental Station of the University of Vermont, Miscellaneous Publications 117: 1-47.

Grehan, J.R., B.L. Parker, G.R. Nielsen, D.H. Miller. J.D. Hedbor, M. Sabourin, and M.S. Griggs. 1995b. Moths and Butterflies of Vermont (Lepidoptera), A Faunal Checklist. Vermont Department of Forests, Parks and Recreation, Miscellaneous Publication 116, Vermont Monitoring Cooperative Bulletin No.1.

Grevstad, F.S., E.M. Coombs and P.B. McEvoy. 2013 Revisiting release strategies in biological control of weeds: Are we using enough releases? 368-376 Pp. In: Y. WU T. Johnson, S. Sing, S. Raghu, G. Wheeler, P. Pratt, K. Warner, T. Center, J. Goolsby, and R. Reardon. (eds.). Proceedings of the XIII International Symposium on Biological Control of Weeds, September 11-16, 2011, Waikoloa, Hawaii, USA.

Grieshuber, J. and G. Lamas. 2007. A synonymic list of the genus *Colias* Fabricius, 1807. Mitteilungen der Münchner Entomologischen Gesellschaft 97: 131-171.

Griggs, M.S. and J.R. Grehan 2001. Light Trap Survey of Moth Biodiversity of Mount Mansfield for 2000. Vermont Monitoring Cooperative, Essex Junction, Vermont.

Grimble, G.D., R. Beckwith, and P.R. Hammond. 1992. A survey of the Lepidoptera fauna from the Blue Mountains of eastern Oregon. Journal of Research on Lepidoptera 31(1-2): 83-102.

Grimble, D.G. R.C. Beckwith, and P.C. Hammond. 1993. New Lepidoptera Records for the Blue Mountains of Eastern Oregon. United States Department of Agriculture, Forest Service Reserch Paper PNW-RP-469.

Grimes, L.R. and H.H. Neunzig. 1984. The larvae and pupae of three phycitine species (Lepidoptera: Pyralidae) that occur in Florida [Hypargyria slossonella, Davara caricae, Sarasota plumigerella, Hippocratea volubilis, Carica papaya, Coccoloba uvifera]. Proceedings-Entomological Society of Washington 86(2): 411-421.

Grisham, C.H. 1999a. State coordinator reports. Alabama. Southern Lepidopterists' News 21(1): 13-14.

Grisham, C.H. 1999b. State coordinator reports, Alabama. News of Southern Lepidopterists' Society 21(3): 51-53.

Grisham, C.H. 2000. State Coordinator Reports, Alabama. News of Southern Lepidopterists' Society 22(2) 35-36.

Grisham, C.H. 2019a. Genomes of skipper butterflies reveal extensive convergence of wing patterns. Appendix. In: Li, W.L., Q. Cong, J.H. Shen, J. Zhang, W. Hallwachs, D.H. Janzen, and N.V. Grishin. 2019. PNAS 116(13): 6232-6237.

Grishin, N.V. and C.J. Durden. 2012. New Bromeliad-feeding *Strymon* species from Big Bend National Park, Texas, USA and its vicinity (Lycaenidae: Theclinae). Journal of the Lepidopterists’ Society 66(2): 81-110.

Grosman, A.H., M van Breemen, A. Holtz, A. Pallini, A.M. Rugama AM, Harvey Pengel, M. Venzon, J.C. Zanuncio, M.W. Sabelis, and A. Janssen. 2005. Searching behaviour of an omnivorous predator for novel and native host plants of its herbivores: a study on arthropod colonization eucalyptus in Brazil. Entomologia Experimentalis et Applicata 116: 135-142.

Groman, J.D. and O. Pellmyr. 2000. Rapid evolution and specialization following host colonization in a yucca moth. Journal of Evolutionary Biology 13: 223-236.

Gross, P. 1986. Life histories and geographic distributions of two leafminers, *Tildenia georgei* and *T. inconspicuella* (Lepidoptera: Gelechiidae), on solanaceous weeds. Annals of the Entomological Society of America 9(1): 48-55.

Grossbeck, J.A. 1906a. Some new species of Geometridae. The Canadian Entomologist 38: 272-275.

Grossbeck, J.A. 1908a. Additions to the list of North American Geometridae, with notes on some described species. Proceedings of the Entomological Society of Washington 10(1/2): 85-91.

Grossbeck, J.A. 1908b. New Moths of the Family Geometridæ. Journal of the New York Entomological Society 16(1): 19-31.

Grossbeck, J.A. 1909a. Some new species of North American Geometridae. The Canadian Entomologist 41(5): 153-157.

Grossbeck, J.A. 1910. New species and one new genus of Geometridae. Journal of the New York Entomological Society 18(4): 199-207.

Grossbeck, J.A. 1912. A review of the species comprising the *Glaucina*-*Coenocharis* group. Bulletin of the Bulletin of the American Museum of Natural History 31(31): 387-407.

Grossbeck, J.A. 1917. Insects of Florida. IV. Lepidoptera. Bulletin of the American Museum of Natural History 37: 1-147.

Grote, A.R. 1864. [dated 1863]. Additions to the catalogue of U.S. Lepidoptera, No. 5. Proceedings of the Entomological Society of Philadelphia 2(3): 273-276.

Grote, A.R. 1874a. A list of the Noctuidae of North America. Bulletin of the Buffalo Society of Natural Sciences 2: 1-78.

Grote, A.R. 1874b. X. Notes on American Lepidoptera with descriptions of twenty-one new species. Bulletin of the Buffalo Society of Natural Sciences 2: 145-163.

Grote, A.R. 1875a. Descriptions of North American moths. Transactions of the American Entomological Society 5: 113-118.

Grote, A.R. 1878. New species of *Acopa* and *Heliothis*, and note on *Hamadryas*. The Canadian Entomologist 10(4): 67-69.

Grote, A.R. 1879a. Article 13. On *Lithophane* and new Noctuidae. Bulletin of the United States Geological and Geographical Survey of the Territories 5(2): 201-208.

Grote, A.R. 1882a. New species and structure of moths and genera. The Canadian Entomologist 14(11): 212-218

Grote, A.R. 1882b. New species and structure of moths and genera. The Canadian Entomologist 14(12): 234-237.

Grote, A.R. 1883a. VII. The Moths of New Mexico. The Annals and Magazine of Natural History 51: 49-58.

Grote, A.R. 1883b. Notes on some new species in Mr. Neumoegen's collection. Papilio 3(4): 73-80

Grote, A.R. 1883c. New species and structure of moths and genera. The Canadian Entomologist 15(1): 3-13

Grote, A.R. 1883d. New species and structure of moths and genera. The Canadian Entomologist 15(1): 23-31.

Grote, A.R. 1883e [dated 1882]. Some new Noctuidae and notes. Papilio 2(9/10): 183-187.

Grote, A.R. 1886. North American Lepidoptera. The hawk moths of North America. Privately published, Bremen.

Grote, A.R. 1895. List of North American Eupterotidae, Ptilodontae, Thyatiridae, Apatelidae and Agrotidae. Privately published, Bremen.

Grote, A.R. and C.T. Robinson. 1868. Notes on the North American Lepidoptera in the British Museum and described by Mr. Francis Walker. Transactions of the American Entomological Society 2: 67-88.

Gruber, J.W., T.A. Matson, and D.L. Wagner. 2021. *Alypiodes flavilinguis* Grote, 1883 as a valid species (Noctuidae, Agaristinae). Journal of the Lepidopterists' Society 75(2): 81-87.

Guagliumi, P. 1962. Las plagas de la caña de azúcar en Venezuela. Ministerio de Agricultura y Cría, [MAG (Ministry of Agriculture and Livestock)], Centro de Investigaciones Agronomicas, Maracay, Venezuela.

Guedes, R. da S., T.L.S. Fernandes, and F.C.V. Zanella. 2021. First record of *Numia terebintharia* Gueneé (Lepidoptera: Geometridae) in *Ziziphus joazeiro mart* (Rhamnaceae) in Brazil. Revista Caatinga 34(1): 236-241.

Guillén, M., D.R., Davis, and J.M. Heraty. 2001. Systematics and biology of a new, polyphagous species of *Marmara* (Lepidoptera: Gracillariidae) infesting grapefruit in the southwestern United States. Proceedings of the Entomological Society of Washington 103(3): 636-654.

Guillermet, C. 2009. Les Hétérocères, ou papillons de nuit, de l'île de La Réunion. Volume 3. Familles des Pyralidae et Crambidae. Nature Découverte et Partage, Parc National de La Réunion. 552. Pp.

Gulmahamad, H. 1999. Establishment of an exotic plaster bagworm in California (Lepidoptera: Tineidae). Pan-Pacific Entomogist 75: 165-169.

Guppy, C.S. and J.H. Shepard. 2001. Butterflies of British Columbia: Including Western Alberta, southern Yukon, the Alaska Panhandle, Washington, Northern Oregon, Northern Idaho, Northwestern Montana. University of British Columbia Press, Vancouver, Canada. 414pp.

Gurney, A.B. 1963. A brief look at the Dismal Swamp and its Natural History, especially the insects. Journal of the Washington Academy of Science 53: 57-63.

Gurule, S.A., S.M. Nikam, A.J. Kharat, and J.H. Gangurde. 2010. Check-list of owlet and underwing moth (Lepidoptera: Noctuidae) from Nashik district, (MS) India. Flora and Fauna (Jhansi) 16(20): 295-304.

Gutiérrez, J.L.S., A. D. Warren,and A.L. Martínez. 2005. Hesperioidea (Lepidoptera: Rhopalocera) del occidente de México. Folia Entomológica Mexicana 44(3): 305-320.

Gwiazdowski, R.A., J.S. Elkinton, J.R. Dewaard, and M. Sremac. 2013. Phylogeographic diversity of the winter moths *Operophtera brumata* and *O. bruceat*a (Lepidoptera: Geometridae) in Europe and North America. Annals of the Entomological Society of America 106(2): 143-151.

Habeck, D.H. 1974. Caterpillars of *Poropoynx* in relation to aquatic plants In Florida. Florida Agriculture Experimental Station Journal Series 5331: 15-18.

Habeck, D. H. 1988. *Neargyractis slossonalis* (Lepidoptera: Pyralidae, Nymphulinae): larval description and biological notes. Florida Entomologist 71(4): 588-592.

Habeck, D.H. 2007. Report of an unusual Arctiid catch in Alachua County, Florida. Southern Lepidopterists' News 29(1): 1-2.

Habeck, D.H. and F.D. Bennett. 1990. *Cactoblastis cactorum* Berg (Lepidoptera: Pyralidae), a Phycitine new to Florida. Florida Department of Agriculture and Consumer Services. Entomology Circular No 333: 11-4.

Habeck, D. and M.A. Solis. 1994. Transfer of *Petrophila drumalis* (Dyar) to *Argyractis* based on immature and adult characters with a larval description of *Argyractis subornata* (Hampson) (Lepidoptera: Crambidae: Nymphulinae). Proceedings of the Entomological Society of Washington 96 (4): 726-734.

Habeck, D.H., R.T. Arbogast, and L.D. Cline 1974. Biology and immature stages of *Schinia mitis* (Grote) (Noctuidae). Journal of the Lepidopterists’ Society 28: 152-157.

Hackland, K.W. 2004. South Texas Nature Guide, featuring the remarkable lower Rio Grande Valley. RGV Nature Marketing Co-op, Weslaco, Texas.

Haimbach, F. 1907. Two new species of Crambus and a new variety of *Haematopsis grataria* Frabriciys. Entomological News 18(2); 44-45

Haimbach, F. 1915. New Heterocera (Lep.). Entomological News and Proceedings of the Entomological Section of the Academy of Natural Sciences of Philadelphia 26(7): 321-325.

Haimbach, F. 1928. A list of the species and descriptions of new forms of the American genus *Zale* and a new form of *Safia* (Lepidoptera: Noctuidae, Catocalinae). Transactions of the American Entomological Society 54(3): 215-232.

Hain, F.P. and W.E. Wallner. 1973. The life history, biology, and parasites of the pine candle moth, *Exoteleia nepheos* (Lepidoptera: Gelechiidae), on Scotch pine in Michigan. The Canadian Entomologist 105(1): 157-164.

Halbert, S.E. 2016. Entomology. Tri-Ology 55(3): 6-10.

Halbert, S.E. 2019a. Entomology. Tri-Ology 58(1): 8-9.

Halbert, S.E. 2019b. Entomology specimen report. Tri-Ology 58(2): 10-14.

Hall, D. 2009. American lady, American painted lady, *Vanessa virginiensis* (Drury) (Insecta: Lepidoptera: Nymphalidae: Nymphalinae). University of Florida, IFAS Extension. EENY-449.

Hall, D. 2014. Eastern Black Swallowtail: *Papilio polyxenes asterius* (Stoll) (Insecta: Lepidoptera: Papilionidae). Revised. Entomology and Nematology Department, University of Florida, IFAS Extension. EENY-504. Original publication date November 2011.

Hall, P. 2007. The European Common Blue (*Polyommatus icarus*): A new alien butterfly to canada and North America. News of the Lepidopterists’ Society 29(2): 111.

Hall, J.P.W. and D.J. Harvey 2002. A Phylogenetic Review of Charis and Calephelis (Lepidoptera: Riodinidae). Annals of the Entomological Society of America 95(4): 407-421.

Hall, S. 2000. State coordinator reports, North Carolina. News of Southern Lepidopterists' Society 22(2) 40-41.

Hall, S. 2002. State coordinator reports, North Carolina. News of Southern Lepidopterists' Society 24(4): 105-108.

Hall, S. 2011. State coordinator reports, North Carolina. News of Southern Lepidopterists' Society 33(3): 132-134.

Hall, S.P., J.B. Sullivan, and D.F. Schweitzer. 1999. Assessment of Risk to Non-Target Macro-Moths after *Bacillus thuringiensis* var. *kurstaki* Application to Asian Gypsy Moth in the Cape Fear Region of North Carolina. United States Department of Agriculture FHTET-98-16.

Hall, S.P., J.B. Sullivan, J.W. Petranka, T.Feldman, D. Georgek, J. Nizni, P. Backstrom, and T. Howard. 2025. The Moths of North Carolina [Internet]. Raleigh (NC): North Carolina Biodiversity Project and North Carolina State Parks. Available from https://auth1.dpr.ncparks.gov/moths/index.php.

Hallman, G.J., and G.G. Sanchez. 1982. Possibilities for biological control of *Antigastra catalaunalis* [Lep.: Pyralidae], a new pest of sesame in the western hemisphere. Entomophaga 27(4): 425-429.

Halperin, J. and W. Sauter. 1992. An annotated list with new records of Lepidoptera associated with forest and ornamental trees and shrubs in Israel. Israel Journal of Entomology 25-26: 105-147.

Hamilton, J.G. and M.P. Zalucki. 1993a. Interactions between a specialist herbivore, *Crocidosema plebejana,* and its host plants *Malva parviflora* and cotton, *Gossypium hisutum*: larval performance. Entomologia Experimentalis et Applicata 66(3): 199-205.

Hammer, R.L. 1998. Satinleaf, *Chrydophyllum oliviforme*, a new larval hostplant record for the Ello Sphinx, *Erinnyis ello*. News of the Lepidopterists’ Society 40: 19.

Hammon, R. 2009. Insect Pests of Grass and Forb Seed Production. Colorado State University Extension. Pp. 66-67 In: Great Basin Native Plant Selection and Increase Project FY08 Progress Report. USDI Bureau of Land Management, Great Basin Restoration Initiative.

Hammond, P.C. and E.J. Dornfeld. 1983. A new subspecies of *Speyeria egleis* from the pumice region of central Oregon. Journal of the Lepidopterists' Society 37(2): 115-120.

Hammond, P.C. and D.G Grimble. 1997. Distribution of a northern fauna of Noctuidae in the mountains of Oregon. Journal of the Lepidopterists' Society 51: 97-101.

Hammond, P.C. and D.V. McCorkle. 2003. A new desert subspecies of *Colias occidentalis* (Pieridae) from southeastern Oregon. Journal of the Lepidopterists’ Society 57(4): 274-278.

Hammond, P.C. and D.V. McCorkle. 2008. A review of geographic variation and possible evolutionary relationships in the *Colias* *scudderii-gigantea* complex of North America (Pieridae). Journal of the Lepidopterists’ Society 62(4): 201-215.

Hammond, P.C. and D.N. McCorkle, 2017. Taxonomy, ecology, and evolutionary theory of the genus *Colias* (Lepidoptera: Pieridae: Coliadinae). Published by the authors, Corvallis, Oregon. 265 pp.

Hampson, G.F. 1895. On the Geometridae, Pyralidae, and allied families of Heterocera of the Lesser Antilles. Annals and Magazine of Natural History, 6^th^ series 16: 329-349.

Hampson, G.F. 1896. The Fauna of British India, Ceylon and Burma. Moths. Vol. 4. Taylor and Francis, London.

Hampson, G.F. 1898a. Catalogue of the Lepidoptera Phalaenae in the British Museum, Volume I. Catalogue of the Lepidoptera Syntomidae in the collection of the British Museum. Taylor and Francis, London.

Hampson, G.F. 1900. Catalogue of the Lepidoptera Phalaenae in the British Museum, Volume 2. Arctiadae (Nolinae, Lithosianae). Taylor and Francis, London.

Hampson, G.F. 1901. Catalogue of the Lepidoptera Phalaenae in the British Museum, Volume 3. Catalogue of the Lepidoptera Arctiadae (Arctianae) and Agaristidae in the collection of the British Museum. Taylor and Francis, London.

Hampson, G.F. 1903. Catalogue of the Lepidoptera Phalaenae in the British Museum, Volume 4. Catalogue of the Lepidoptera Noctuidae in the Collection of the British Museum. Taylor and Francis, London.

Hampson, G.F. 1905. Catalogue of the Lepidoptera Phalaenae in the British Museum, Volume 5. Catalogue of the Lepidoptera Noctuidae in the collection of the British Museum. Taylor and Francis, London.

Hampson, G.F. 1906a. On new Thyrididae and Pyralidae. Annals and Magazine of Natural History, including Zoology, Botany and Geology, London (ser. 7) 17: 112-147, 189-222, 253-269, 344-359.

Hampson, G.F. 1906b. Catalogue of the Lepidoptera Phalaenae in the British Museum, Volume 6. Catalogue of the Noctuidae in the collection of the British Museum. British Museum of Natural History, London.

Hampson, G.F. 1906c. LV - Descriptions of new Pyralidae of the subfamilies Hydrocampinae and Scoparianae. Annals and Magazine of Natural History, including Zoology, Botany and Geology, London (ser. 7) 18: 373-393.

Hampson, G.F. 1908. Catalogue of the Lepidoptera Phalaenae in the British Museum, Volume 7. Catalogue of the Noctuidae in the collection of the British Museum. British Museum of Natural History, London.

Hampson, G.F. 1909. Catalogue of the Lepidoptera Phalaenae in the British Museum, Volume 8. Catalogue of the Noctuidae in the collection of the British Museum. British Museum of Natural History, London.

Hampson, G.F. 1910a. Catalogue of the Lepidoptera Phalaenae in the British Museum, Volume 9. Catalogue of the Noctuidae in the collection of the British Museum. British Museum of Natural History. Taylor and Francis, London.

Hampson, G.F. 1910b. Catalogue of the Lepidoptera Phalaenae in the British Museum, Volume 10. Catalogue of the Noctuidae in the collection of the British Museum. British Museum of Natural History. Taylor and Francis, London.

Hampson, G.F. 1912. Catalogue of the Lepidoptera Phalaenae in the British Museum, Volume 11. Catalogue of the Noctuidae in the collection of the British Museum. British Museum of Natural History. Taylor and Francis, London. 689 pp.

Hampson, G.F. 1913. Catalogue of the Lepidoptera Phalaenae in the British Museum, Volume 13. Catalogue of the Noctuidae in the collection of the British Museum. British Museum of Natural History. Taylor and Francis, London.

Hampson, G.F. 1914. Catalogue of the Lepidoptera Phalaenae in the British Museum, Supplement Volume 1. Catalogue of the Amatidae and Arctiadae (Nolinae and Lithosianae in the collection of the British Museum. British Museum of Natural History. Taylor and Francis, London.

Hampson, G.F. 1917. A classification of the Pyralidae, subfamily Gallerianae. Novitates Zoologicae 24(1): 17-58.

Hampson, G.F. 1920. Catalogue of the Lepidoptera Phalaenae in the British Museum, Supplement Volume 2. Catalogue of the Lepidoptera Lithosiadae (Arctinae) and Phalaenoididae in the collection of the British Museum. British Museum of Natural History, London.

Hampton, N. 2005. Insects of the Idaho National Laboratory: A Compilation and Review. 116-130. In: N.L. Shaw, M. Pellant, and S.B. Monsen, (compilers). Sagegrouse habitat restoration symposium proceedings; 2001 June 4-7, Boise, ID. U.S. Department of Agriculture, Forest Service Proceedings RMRS-P-38.

Handfield, L. 1997. Liste des Lépidoptères du Québec et du Labrador. Fabreries, Supplement 7. 155 pp.

Handfield, L. 1999. Le guide des papillons du Québec. Version populaire. Broquet, Ottawa, 536 pp. + 123 plates.

Handfield, L. 2002. Additions, corrections et radiations à la liste des Lépidoptères du Québec. Fabreres 27: 1-46.

Handfield, D. and L. Handfield, 2006a. A new species of *Plusia* (Lepidoptera: Noctuidae) from North America. Canadian Entomologist 138(6): 853-859.

Handfield, L. and D. Handfield. 2006b. A new species of *Capis* (Lepidoptera: Noctuidae) from Québec, Canada. Canadian Entomolgist 138: 333-338.

Handfield, L. and D. Handfield. 2010a. *Cucullia umbratica* (Lepidoptera, Noctuidae), a new European noctuid in North America. In: B.C. Schmidt and J.D. Lafontaine, (Eds.). Contributions to the systematics of New World macro-moths II. ZooKeys 39: 183-186.

Handfield, L. and D. Handfield. 2011. A new species of *Herpetogramma* (Lepidoptera, Crambidae, Spilomelinae) from eastern North America. ZooKeys 149: 5-15.

Handfield, L. and N. Handfield. 2021a. New species of the genus *Paranthrene* Hbn., 1819 (Lepidoptera, Sesiidae, Sesiinae). Journal of th Lepidopterist Society 75(4): 252-8.

Handfield, L. and D. Handfield. 2021b. A revision of the Canadian species of the genus *Herpetogramma* Lederer, 1863 (Lepidoptera: Crambidae: Spilomelinae: Herpetogrammatini), with descriptions of three new species. Bonn Zoological Bulletin 70(1): 173-199.

Handfield, L. and N. Handfield, 2024. A new species of *Sesia* from northeastern North America and a resurrected species (Lepidoptera: Sesiidae: Sesiinae). Lepidoptera Novae 17(3): 89-116.

Hanham, A.W. 1901. A list of Manitoba moths - part V. Canadian Entomologist 33(8): 213-220.

Hannemann, H.J. 1961. Kleinschmetterlinge oder Microlepidoptera. I. Die Wickler (s. str.) (Tortricidae). Die Tierwelt Deutschlands 48: i-xi 1-233 & plates 1-22.

Hannon, E.R., R.A. Rodstrom, J.M. Chong, and J.J. Brown. 2017. Carpenterworm moth. Insect Pest Management in Hybrid Poplars Series. Washington State University, Pullman, WA.

Hanock. E.F. and K.P. Bland. 2015. The Moths and Butterflies of Great Britain and Ireland, Voulme 5. Tortricidae, part 2: Olethreutinae. Brill, Leiden, The Netherlands.

Hanson, T.C. 1975. Notes on Brazilian butterflies. Bulletin of the Amateur Entomologists' Society 34: 80-83.

Hanson, D.L., E. Knudson, and C. Bordelon. 2003. *Phocides belus* Godman & Salvin (Hesperiidae), new to US and Texas with a review of P*hocides* & similar species of the USA and northern Mexico. News of the Lepidopterists' Society 45(2): 42-43.

Hanson, P., K. Nishida, P. Allen, E. Chacón, B. Reichert, A. Castillo, M. Alfaro, L.Madrigal, E. Rojas, F. Badenes-Perez, and T. Johnson. Insects that feed on Miconia calvescens in Costa Rica. 2009 International Miconia Conference, Maui Invasive Species Committee, Keanae, Hawaii. 12pp.

Hansson, C., M.A. Smith, D.H. Janzen, and W. Hallwachs. 2015. Integrative taxonomy of New World *Euplectrus* Westwood (Hymenoptera, Eulophidae), with focus on 55 new species from Area de Conservación Guanacaste, northwestern Costa Rica. Zookeys 485: 1-236.

Hanus, J. and M.-L. Theye. 2010. *Parnassius phoebus* (Fabricius, 1793), a misidentified species (Lepidoptera: Papilionidae). Nachrichten des Entomologischen Vereins Apollo N. F. 31(1/2): 71-84.

Hanus, J. and M.-L. Theye. 2011. Supplements to "*Parnassius phoebus* (Fabricius, 1793), a misidentified species" (Lepidoptera: Papilionidae). Nachrichten des Entomologischen Vereins Apollo N. F. 32(1/2): 25-27.

Harding, J.W. 1971. Observations on *Lampides boeticus* (L.) (Lepidoptera: Lycaenidae). New Zealand Entomologist 1971 Vol. 5 No. 1 pp. 70-73.

Hardwick, D.F. 1958. Taxonomy, life history, and habits of the elliptoid-eyed species of Schinia, with notes on the Heliothidinae. Memoirs of the Entomological Society of Canada 90(Suppl. 6): 1-116.

Hardwick, D.F. 1965a. The *ochrogaster* group of the genus *Euxoa* (Lepidoptera: Noctuidae), with description of a new species. The Canadian Entomologist 97(07): 673-678.

Hardwick, D.F. 1965b. A new species belonging to the *divergens* group of the genus *Euxoa* Hbn. (Lepidoptera: Noctuidae). The Canadian Entomologist, 97(08), 822-826.

Hardwick, D.F. 1965c. A synopsis of the *ridingsiana* group of the Genus *Euxoa* (Lepidoptera: Noctuidae) with a description of a new species. The Canadian Entomologist 97(11): 1221-1226.

Hardwick, D.F. 1966. The life history of *Schinia niveicosta* (Noctiudae). Journal of the Lepidopterists' Society 20(1): 29-33.

Hardwick, D.F. 1970a. The life history of *Pyrrhia exprimens* (Noctuidae). Journal of the Lepidopterists’ Society 24: 234-239.

Hardwick, D.F. 1970b. The life history of *Schinia florida* (Noctuidae). Journal of the Lepidopterists’ Society 24: 282-287.

Hardwick, D.F. 1970c. The Genus *Euxoa* (Lepidoptera: Noctuidae) in North America. I. subgenera *Orosagrotis*, *Longivesca*, *Chorizagrotis*, *Pleonectopoda*, and *Crassivesica*. Memoirs of the Entomological Society of Canada 67: 1-177.

Hardwick, D.F. 1971a. The life history of *Schinia separata* (Noctuidae). Journal of the Lepidopterists' Society 25(3): 177-180.

Hardwick, D.F. 1971b. The life history of *Schinia walsinghami* (Noctuidae). Journal of the Lepidopterists' Society 25(3): 181-185.

Hardwick, D.F. 1971c. The life history of *Schinia cupes deserticola* (Noctuidae). Journal of the Lepidopterists' Society 25(2); 109-114.

Hardwick, D.F. 1971d. The life history of *Schinia ligeae* (Noctuidae). Journal of the Lepidopterists' Society 25(4): 274-277.

Hardwick, D.F. 1971e. The life history of *Heliothis oregonica* (Noctuidae). Journal of the Lepidopterists' Society 25(1): 1-6.

Hardwick, D.F. 1971f. The life history of *Schinia cupes deserticola* (Noctuidae). Journal of the Lepidopterists' Society 25(2): 109-114.

Hardwick, D.F. 1971g. The life history of *Heliolonche pictipennis* (Noctuidae). Journal of the Lepidopterists' Society 25(4): 287-29.

Hardwick, D.F. 1972a. The life history of *Schinia citrinellus* (Noctuidae). Journal of the Lepidopterists' Society 26(2): 82-86.

Hardwick, D.F. 1972b. The life history of *Schinia intrabilis* (Noctuidae). Journal of the Lepidopterists' Society 26(1): 24-28.

Hardwick, D.F. 1972c. The life history of *Schinia jaegeri* (Noctuidae). Journal of the Lepidopterists' Society 26(2): 89-93.

Hardwick, D.F. 1972d. The life history of *Schinia pallicincta* (Noctuidae). Journal of the Lepidopterists' Society 26(1): 29-33.

Hardwick, D.F. 1973a. A new species of *Schinia* (Lepidoptera: Noctuidae) from Steens Mt., Oregon. The Canadian Entomologist 105(7): 1003-1004.

Hardwick, D.F. 1983a. A new species of *Schinia* (Noctuidae) from central Florida, with description of its life history. Journal of the Lepidopterists' Society 37(2): 148-154.

Hardwick, D.F. 1983b. A new species of *Schinia* (Noctuidae) from Manitoba and Saskatchewan with description of its life history. Journal of the Lepidopterists’ Society 37(1): 18-23.

Hardwick, D.F. 1994. A review of the *phloxiphaga* group of the genus *Heliothis* (Noctuidae: Heliothentinae) with description of a new species. Journal of the Lepidopterists' Society 38(2): 106-110.

Hardwick, D.F. 1996. A monograph to the North American Heliothentinae (Lepidoptera: Noctuidae). Privately published, Ottawa.

Hardwick, D.F. and K. Stead. 1998. The life history of *Schinia sanguinea* (Geyer) (Noctuidae: Heliothentinae) with a report on a survey for *Heterocera* in southwestern Ontario. Journal of the Lepidopterists' Society 52(4): 381-385.

Hardy, D., M.A. Rickard, A.D. Warren, and N.V. Grishin. 2011. *Achalarus tehuacana* (Hesperiidae: Eudaminae): a new United States record from southern Texas. News of the Lepidopterists' Society. 52(4): 107-111, 127.

Hardy, G.A. 1943. Field observations on the forest tent caterpillar, *Malacosoma disstria* var. *erosa* Stretch. Proceedings of the Entomological Society of British Columbia, 40: 28-29.

Hargrove, W.W. 1986. An annotated species list of insect herbivores commonly associated with black locust, *Robinia pseudoacacia*, in the Southern Appalachians. - Entomological News 97: 36-40.

Harms, N.E. and M.J. Grodowitz. 2009. Insect herbivores of aquatic and wetland plants in the United States: A checklist from literature. Journal of Aquatic Plant Management 47: 73-96.

Harmsen, R., P.D.N. Hebert, and P.S. Ward. 1973. On the origin of austral elements in the moth fauna of south-eastern Ontario, including a number of species new for Canada. Journal of Research on the Lepidoptera 12(3): 127-134.

Harp, C.E. 2007. 2006 Season Summary. Zone 4 Rocky Mountains, Alberta, Montana, Wyoming, Utah, Colorado, New Mexico. News of the Lepidopterists’ Society 49(S1): 14-35.

Harp, C.E. 2008. 2007 Season Summary. Zone 4 Rocky Mountains, Alberta, Montana, Wyoming, Utah, Colorado, New Mexico. News of the Lepidopterists’ Society 50(S1): 15-50.

Harp, C.E. 2009. 2008 Season Summary. Zone 4 Rocky Mountains, Alberta, Montana, Wyoming, Utah, Colorado, New Mexico. News of the Lepidopterists’ Society 51(S1): 17- 44

Harp, C.E. 2010. 2009 Season Summary. Zone 4 Rocky Mountains, Alberta, Montana, Wyoming, Utah, Colorado, New Mexico. News of the Lepidopterists’ Society 52(S1): 14-57.

Harp, C.E. 2011. 2010 Season Summary. Zone 4 Rocky Mountains, Alberta, Montana, Wyoming, Utah, Colorado, New Mexico. News of the Lepidopterists’ Society 53(S1): 29-71.

Harp, C.E. 2012. 2011 Season Summary. Zone 4 Rocky Mountains, Alberta, Montana, Wyoming, Utah, Colorado, New Mexico. News of the Lepidopterists’ Society 54(S1): 21-60.

Harp, C.E. 2013. 2012 Season Summary. Zone 4 Rocky Mountains, Alberta, Montana, Wyoming, Utah, Colorado, New Mexico. News of the Lepidopterists’ Society 55(S1): 16-16.

Harp, C.E. 2014. 2013 Season Summary. Zone 4 Rocky Mountains, Alberta, Montana, Wyoming, Utah, Colorado, New Mexico. News of the Lepidopterists’ Society 56(S1): 23-59.

Harp, C.E. 2015. 2014 Season Summary. Zone 4 Rocky Mountains, Alberta, Montana, Wyoming, Utah, Colorado, New Mexico. News of the Lepidopterists’ Society 57(S1): 28-78.

Harp, C.E. 2016. 2015 Season Summary. Zone 4 Rocky Mountains, Alberta, Montana, Wyoming, Utah, Colorado, New Mexico. News of the Lepidopterists’ Society 58(S1): 40-80.

Harp, C.E. 2017. 2016 Season Summary. Zone 4 Rocky Mountains, Alberta, Montana, Wyoming, Utah, Colorado, New Mexico. News of the Lepidopterists’ Society 59(S1): 42-75.

Harper, M.W., A.M. Emmet and J.R. Langmaid, 2002. Oecophoridae. pp. 43-177 In: A.M. Emmet and J.R. Langmaid (eds.), The Moths and Butterflies of Great Britain and Ireland, Volume 4, part 1, Oecophoridae - Scythrididae (excluding Gelechiidae). Harley Books, Colchester, Essex, England.

Harrendorf, K. 1959. Occurrence and relative abundance of certain noctuid moths in northwest Arkansas, Fall, 1957. Journal of the Kansas Entomological Society 32(1): 41-44.

Harris, T.W. 1839. Descriptive catalogue of the North American insects belonging to the Linnaean genus *Sphinx* in the cabinet of Thaddeus William Harris, M.D., Librarian of the Harvard University. American Journal of Science and Arts 36: 282-320.

Harris, T.W. 1841. A report on insects of Massachusettes injurious to vegetation. Folsom, Wells, and Thurston, Cambridge, Massachusettes. 459 pp.

Harris, T.W. 1880. A Treatise on Some of the Insects Injurious to Vegetation. Orange Judd Co., New York.

Harrison, T.L. 2005. A new species of Douglasiidae (Lepidoptera) from the eastern Nearctic. Proceedings of the Entomological Society of Washington 107(3): 596-603.

Harrison, T.L. 2011. Microlepidoptera of Illinois Hill Prairies. Dissertation, University of Illinois, Urbana-Champaign, Illinois.

Harrison, T.L. and M.R. Berenbaum. 2005. Rutaceae-feeding *Agonopterix* Hübner (Lepidoptera: Elachistidae) in Illinois. Proceedings of the Entomological Society of Washington 107: 162-175.

Harrison, T.L. and M. Berenbaum. 2013a. A new, prairie-restricted species of *Filatima* Busck (Lepidoptera: Gelechiidae) from Illinois. Zootaxa 3734(4): 469-476.

Harrison, T.L. and M. Berenbaum. 2013b. A new, prairie-restricted species of *Anacampsis* Curtis (Lepidoptera: Gelechiidae) from Illinois. Zootaxa 3741(1): 194-200.

Harrison, T.L. and M.R. Berenbaum. 2013c. Moth diversity in three biofuel crops and native prairie in Illinois. Insect Science 20: 407-419.

Harrison, T.L. and M. Berenbaum. 2014. *Anacampsis rhoifructella* (Clemens): clarification of its identity and larval biology, and differentiation from a similar species, *Anacampsis consonella* (Zeller), revised status (Lepidoptera: Gelechiidae). Zootaxa 3794(4): 545-555.

Harrison, T.L. and S. Passoa. 1995. *Mirabilis*-feeding *Heliodines* (Lepidoptera: Heliodinidae) in central Illinois, with description of a new species. Proceedings of the Entomological Society of Washington 97: 63-70.

Harrison, T.L., L.D. Gibson, and T.M. Gilligan, 2014. A new species of *Grapholita* Treitschke (Lepidoptera: Tortricidae) from the midwestern USA. Zootaxa 3755 (3): 287-294.

Harrison, T., A. Lawrance, and D. Taron. 2019. More on *Lepidotarphius perornatella* (Lepidoptera: Glyphipterigidae) in North America. News of the Lepidopterists’ Society 61(3): 125.

Harry, J.L. 2009. Natural life histories of Alaska *Colias* (Lepidoptera: Pieridae). The Taxonomic Report 7(2): 1-22.

Harvey, G.T. and G. Stehr. 1967. On coniferophagous species of *Choristoneura* (Lepidoptera: Tortricidae) In North America: III. some characters of immature forms helpful in the identification of species. The Canadian Entomologist 99(05): 464-481.

Hasbrouck, F.F. 1964. Moths of the family Acrolophidae in America north of Mexico (Microlepidoptera). Proceedings of the United States National Museum 114(3475): 487-706.

Haskins, M. 2005. New records for *Ascalapha odorata* (L.) (Lepidoptera, Noctuidae) in Missouri (abstract). Transactions of the Missouri Academy of Science 39:10.

Hättenschwiler, P. 1985. Psychidae. pp. 128-151 *in* A.M. Emmet (ed.), The Moths and Butterflies of Great Britain and Ireland, Volume 2, Cossidae- Heliodinidae. Harley Books, Colchester, Essex, England.

Hausmann, A. and J. Viidalepp. 2012. The geometrid moths of Europe. Vol. 3. Apollo Books, Vester Skerninge, Denmark.

Hausmann, A., P. Hebert, and R. Rougerie. 2009. International Barcode of Life Project. http://www.lepbarcoding.org/ [accessed 15 July 2015]

Hawks, D.C. 2010. Review of the *Catocala delilah* species complex (Lepidoptera, Erebidae). In: B.C. Schmidt and J.D. Lafontaine, (Eds.). Contributions to the systematics of New World macro-moths II. ZooKeys 39: 13-35.

Hawksworth, D.L. 1991. Linnaeus, the first report of Lichenophagy in psychid moths, and the identity of *Lichen candelarius*. The Lichenologist 23(1): 92-92.

Hay, C.J. and R.C. Morris. 1970. Carpenterworm. Forest Pest Leaflet 64. US. Department of Agriculture, Forest Service, Washington, DC.

Hayden, J.E. 2008. Notes on *Metrea ostreonalis* Grote. News of the Lepidopterist Society 50(1): 20-21.

Hayden, J.E. 2009a. A new genus of Caribbean Odontiinae with Palaeotropical affinities (Lepidoptera: Crambidae). Journal of the Lepidopterists’ Society 63(4): 185-208.

Hayden, J.E. 2011. *Erelieva parvulella*, a State record. Tri-ology DACS-P-00124 50(4): 6.

Hayden, J.E. 2012. First US records of *Amorbia concavana* (Zeller) (Lepidoptera: Tortricidae). Insecta Mundi 0271: 1-4

Hayden, J.E. 2013. *Sufetula* Walker in Florida (Lepidoptera: Crambidae). Insecta Mundi 0295: 1-15.

Hayden, J.E. 2014a. The stained-glass moth, *Samea ecclesialis* Guenée (Lepidoptera: Crambidae). Florida Department of Agriculture and Consumer Services, Division of Plant Industry, Entomology Circular 431: 1-5.

Hayden, J.E. 2014b. *Epitamyra albomaculalis*, a pyralid moth, a new continental USA record. Tri-ology 53(4): 1,5.

Hayden, J.E. 2014c. *Niditinea orleansella*, a tineid moth, a new Florida state record. Tri-ology 53(2): 1,5.

Hayden, J.E. 2015a. *Banisia argutula*, a moth, a new Continental USA record. Tri-ology 54(3): 1,5.

Hayden, J.E. 2015b. *Schacontia rasa* Solis and Goldstein, a crambid moth, a new Continental USA record. Tri-ology 54(1): 1, 11.

Hayden, J.E. 2015c. *Anatrachyntis simplex*, false pink bollworm, a new Western Hemisphere record. Tri-ology 54(5): 6.

Hayden, J.E. 2016a. *Salbia* sp., a crambid moth, a new continental USA record. Tri-ology 55(4): 7.

Hayden, J.E. 2016b. *Thiotricha* sp., a gelechiid moth, a new state record. Tri-ology 55(2): 8.

Hayden, J.E. 2016c. *Glaphyria decisa*, a crambid moth, a new continental USA record. Tri-ology 52(2): 7.

Hayden, J.E. 2017a. *Acrolophus* sp., an acrolophine moth, a new Continental USA record. Tri-ology 56(3): 8.

Hayden, J.E. 2017b. *Blastobasis inana* (Butler), a blastobasid moth, a new Western Hemisphere record. Tri-ology 56(3): 7.

Hayden, J.E. 2017c. *Phereoeca allutella* (Rebel), a household casebearer moth, a new Continental USA record. Tri-ology 56(3): 9.

Hayden, J.E. 2017d. *Asturodes fimbriauralis* (Guenée), a crambid moth, a new Continental USA record. Tri-ology 56(4): 7.

Hayden, J.E. 2017e. *Glyphodes rubrocinctalis* (Guenée), a crambid moth, a new Continental USA record Tri-ology 56(4): 7.

Hayden, J.E. 2017f. *Prepona laertes* (Lepidoptera: Nymphalidae), an exotic butterfly. Florida Department of Agriculture and Consumer Services, Entomology Circular No. 434.

Hayden, J.E. 2019. *Anatrachyntis simplex* (Walsingham) in Florida and Texas, with diagnostic notes (Lepidoptera: Cosmopterigidae). Southern Lepidopterists’ News 41(3): 194-197.

Hayden, J.E. 2020. *Hyalorista exuvialis* (Guenée) in Florida. Southern Lepidopterists’ News 42(4): 327-328.

Hayden, J.E. 2021a. Two new species of palm-leaf skeletonizers (Lepidoptera: Pterolonchidae: *Homaledra* Busck). Insecta Mundi 0859: 1-24.

Hayden, J.E. 2021b. *Poryphyrosela minuta* Clark, a clover leafminer, a new Florida State record. Tri-ology 60(2): 9.

Hayden, J.E. 2021c. *Leucoptera coffeella* (Guérin-Méneville & Perrottet), coffee leafminer, a new Continental USA record. Tri-ology 60(4): 7.

Hayden, J.E. 2022a. *Chabula acamasalis* (Walker), a crambid moth, a new Continental USA record. Tri-ology 61(4): 8.

Hayden, J.E. 2023a. *Marmara salictella* Clemens, willow stem miner, a new Florida State record. Tri-ology 62(4): 9.

Hayden, J.E. 2023b. *Stereomita andropogonis* Braun, a gelechiid moth, a new Florida State record. Tri-ology 62(4): 9.

Hayden, J.E. and J.K. Adams. 2023. *Dinumma deponens* Walker, an erebid moth, a new Florida State record. Tri-ology 62(4): 8-9.

Hayden, J.E. and L.J. Buss. 2013. Olive shootworm, P*alpita persimilis* Munroe (Insecta: Lepidoptera: Crambidae). Entomology and Nematology Department, Florida Cooperative Extension Service, Institute of Food and Agricultural Sciences, University of Florida. Document EENY556.

Hayden, J.E. and L.J. Buss. 2019. *Tinea translucens* Meyrick, the Tropical casebearing clothes moth (Lepidoptera: Tineidae). Southern Lepidopterists’ News 41(4): 279-281.

Hayden, J.E. and A.I. Derksen. 2016b. Tripudia paraplesia, a noctuid moth, a new Florida state record. Tri-ology 55(4): 8.

Hayden, J.E. and T.S. Dickel. 2014. New North American records of Pyraloidea (Lepidoptera: Crambidae, Pyralidae) from southern Florida. Insecta Mundi 0361: 1-16.

Hayden, J.E. and T.S. Dickel. 2015. A new *Antaeotricha* species from Florida sandhills and scrub (Lepidoptera, Depressariidae, Stenomatinae). ZooKeys 533: 133-150. doi: 10.3897/zookeys.533.6004

Hayden, J.E. and J.-F. Landry. 2020. *Arcola malloi* (Pastrana), the alligatorweed stemborer, a new synonym of *Macrorrhinia endonephele* (Hampson) (Lepidoptera: Pyralidae: Phycitinae). Insecta Mundi 0768: 1-25.

Hayden, J.E. and D. Matthews. 2024. *Emmelina devriesi* (B. Landry & Gielis), a plume moth, a new Continental USA record. Tri-Ology 63(1): 7.

Hayden, J.E. and MR. Moore. 2019. *Monochroa* sp. cf. *cytisella*, a bracken-galling moth, a species new to science, found on *Pteridium aquilinum* (bracken fern). Tri-ology 58(2): 9.

Hayden, J.E. and MR. Moore. 2022. *Papilio demoleus* Linnaeus, lime swallowtail, a new Continental USA record. Tri-ology 61(4): 9.

Hayden, J.E. and J. Troubridge. 2015. *Nacoleia charesalis* (Walker) spreading in Florida. News of the Lepidopterists’ Society 57(1): 8-10.

Hayden, J.E., P. Dennehy, and J. Vargp. 2011. *Pyrausta cardinalis*, a new continental record (Lepidoptera: Crambidae). News of the Lepidopterists’ Society 53(2): 58-59.

Hayden, J.E., S. Lee, S.C. Passoa, J. Young, J.-F. Landry, V. Nazari, R. Mally, L.A. Somma, and K.M. Ahlmark. 2013. Digital Identification of Microlepidoptera on Solanaceae. USDA-APHIS-PPQ. Identification Technology Program (ITP). Fort Collins, CO. <http://idtools.org/id/leps/micro/> [Accessed 15 May 2018].

Hayden, J.E., E.R. Hoebeke, M.A. Bertone, and V.A. Brou, Jr. 2017. *Diaphania costata* (F.) (Lepidoptera: Crambidae: Spilomelinae), a commonly misidentified pest of ornamental Apocynaceae in the southern United States. Proceedings of the Entomological Society of Washington 119(2): 173-190.

Hayden, J.E, K.M. Burnette, M.R. Moore. 2022a. Pest Alert: Papilio demoleus, Lime Swallowtail, on Key West, Florida. Florida Department of Agriculture and Consumer Services. FDACS-P-01665.

Hayden, J.E., J.B. Heppner, and M.R. Moore. 2022b. *Choreutis sexfasciella* (Sauber), a choreutid moth, a new Florida state record. Tri-ology 61(4): 8.

Hayden, J.E., M.R. Moore and M.A. Solis. 2023a. *Herpetogramma stramineata* (Hampson), a crambid moth, a new Continental USA record. Tri-ology 62(4): 8.

Hayes, A.H. 1975. The larger moths of the Galápagos Inlands (Geometroidea: Sphingoidea and noctuoidea). Proceedings of the California Academy of Sciences, Fourth Series 60(7): 145-208.

Hayward, K.J. 1941. La “polilla negra” del duraznero (*Cydia molesta* Busck). Estación Experimental Agricola de Tucumán Cicular 99: 1-10.

Hayward, K.J. 1947. Algunas plantas huéspedes de las larvas de los hespéridos americanos (Lep. Rhop. Hesp.). Acta Zoologica Lilloana 4: 19-54.

Hayward, K.J. 1969. Datos para el estudio de la ontogenia de lepidópteros argentinos. Miscelánea. Instituto Miguel Lillo. Universidad Nacional de Tucumán 31: 1-142.

Heard, T.A. and W. Pettit. 2005. Review and analysis of the surveys for natural enemies of *Mimosa pigra*: What does it tell us about surveys for broadly distributed hosts? Biological Control 34(3): 247-254.

Heard, T.A. and R. Segura. 2004. Agents for biological control of *Mimosa pigra* in Australia: review and future prospects. Pp 126-140 In: M. Julien, G. Flanagan, T. Heard, B. Hennecke, Q. Paynter, and C. Wilson, (eds.). Research and Management of *Mimosa pigra*, Papers presented at the 3rd International Symposium on the Management of *Mimosa pigra* 23-25 September 2002, Darwin, Australia.

Heard, T.A., R. Zonneveld, R. Segura, and M. Martinez. 2004. Limited success of open field tests to clarify the host range of three species of Lepidoptera of *Mimosa pigra*. 277 to 282 in: J.M. Cullen, D.T. Briese, D.T. Kriticos, W.M. Lonsdale, L. Morin and J.K. Scott, (eds.). XI International Symposium on Biological Control of Weeds.CSIRO Entomology.

Heath, J. and E.C. Pelham-Clinton. 1976. Incurvariidae. pp. 277-299 *in* J. Heath (ed.), The Moths and Butterflies of Great Britain and Ireland, Volume 1, Micropterigidae - Heliozelidae. Blackwell Scientific Publications, Ltd. and The Curwen Press Ltd., London.

Hebert, P.D.N. 1983. Egg dispersal patterns and adult feeding behaviour in the Lepidoptera. The Canadian Entomologist 115(11): 1477-1481.

Hebert, P.D.N., S. Ratnasingham, E.V. Zakharov, A.C. Telfer, V. Levesque-Beaudin, M.A. Milton, S. Pedersen, P. Jannetta, and J.R. deWaard. 2016. Counting animal species with DNA barcodes: Canadian insects. Philosophical Transactions of the Royal Society B 371: 20150333.

Heckford, R.J. 2017. *Achroia grisella* (Fabricius, 1794) (Lep.: Pyralidae): Observations on the larva and adult. The Entomologist's Record and Journal of Variation 129: 179-186.

Heckscher, C.M. 2008. Results of inventory for selected rare and uncommon insect species in Delaware. Natural Heritage and Endangered Species Program, Delaware Division of Fish and Wildlife, Department of Natural Resources and Environmental Control, Smyrna, Delaware. 25 pp.

Hedlin, A.F., H.O. Yates III, D.C. Tovar, B.H. Ebel, T.W. Koerber, and E.P. Merkle. 1980. Cone and seed insects of North American conifers. Canadian Forest Service, Forest Service and Secretaria de Agricultura y Recursos Hidraulicos, Mexico, in cooperation with North American Forest Commission.

Heinrich.C. 1914. Notes on some forest *Coleophora* with descriptions of two new species. Proceedings of the Entomological Society of Washington 16: 65-69.

Heinrich, C. 1917. A new *Coleophora* injurious to apple in California (Lepidoptera; Coleophoridae). Proceedings of the Entomological Society of Washington 19: 135-136.

Heinrich, C. 1920a. On some forest Lepidoptera with descriptions of new species, larvae, and pupae. Proceedings of the United States National Museum 57: 53-96 + 1-13 plates.

Heinrich, C. 1920b. *Colephora* notes with description of two new species (Lepid.). Proceedings of the Entomological Society of Washington 22(7): 159-162.

Heinrich, C. 1921. Some Lepidoptera likely to be confused with the pink bollworm. Journal of Agricultural Research 20(11): 807-836.

Heinrich, C. 1923a. Revision of the North American moths of the subfamily Eucosminae of the family Olethreutidae. United States National Museum Bulletin 123: 1-298.

Heinrich, C. 1923b. Family 15. Coleophoridae *in* Forbes, W.T.M., Lepidoptera of New York and neighboring states. Part I. Primitive forms, Microlepidoptera, Pyraloids, Bombyces. Memoirs, Cornell University Agricultural Experimental Station 68: 202-217.

Heinrich, C. 1923c. New Olethreutinae from eastern United States (Lepidoptera). Proceedings of the Entomological Society of Washington 25: 105-122.

Heinrich, C. 1924. North American Eucosminae, notes and new species (Lepidoptera). Journal of the Washington Academy of Sciences 14: 385-393.

Heinrich, C. 1926. A revision of the North American moths of the subfamily Laspeyresiinae and Olethreutinae. United States National Museum Bulletin 132: 1-216.

Heinrich, C. 1929. Notes on some North American moths of the subfamily Eucosminae. United States National Museum Proceedings 75(8): 1-23.

Heinrich, C. 1931. Notes on and descriptions of some American moths. Proceedings of the United States National Museum 79(13): 1-16.

Heinrich, C. 1939. The cactus-feeding Phycitinae: A contribution toward a revision of the American pyraliloid moths of the family Phycitidae. Proceedings of the United States National Museum 86(3053): 331-413.

Heinrich, C. 1940. Correction of a misused generic name (Lepidoptera, Olethreutidae). The Canadian Entomologist 72(12): 242-243.

Heinrich, C. 1956. American Moths of the subfamily Phycitinae. United States National Museum Bulletin 207: 1-581.

Heinrich, C. and J.J. de Gryse. 1915. On *Acrocercops strigifinitella* Clemens. Proceedings of the Entomological Society of Washington 17(1): 6-23

Heitzman, R. 1961. Life history of *Adelocephala quadrilineata* (Saturniidae). Journal of Research on Lepidoptera 15: 233-234.

Heitzman, R.L. 1973a. Life history studies of *Idaea obfusaria* (Walker) (Geometridae). Journal of Research on the Lepidoptera 12(3): 145-150.

Heitzman, R.L. 1973b. An annotated checklist of the Missouri Geometridae (Lepidoptera). Journal of Research on the Lepidoptera 12(3): 169-179.

Heitzman, R.L. 1974. A new species of *Hypacyrtis* (Geometridae). Journal of Research on the Lepidoptera 13(1): 43-48.

Heitzman, R.L. 1981. Description of the mature larva and notes on *Holochroa dissociaria* (Hulst) (Geometridae: Ennominae). Journal of the Lepidopterists' Society 35(4): 290-296.

Heitzman, R.L. and W.R. Enns 1977. Description of a new species of *Eupithecia* and the male of *E. cocata* Pearsall (Geometridae) Journal of Research on the Lepidoptera 16(2): 72-82.

Heitzman, R.L. and W.R. Enns. 1978 [1980]. Male genitalic illustrations and notes on the Larentiinae (Geometridae) of Missouri. The Journal of Research on the Lepidoptera 17(3): 145-167.

Heitzman, J.R. and R.L. Heitzman. 1972. New butterfly records for the United States. Journal of Lepidoptera Research 10(4): 284-286.

Heitzman, J.R. and J.E. Heitzman, 1987. Butterflies and Moths of Missouri. Missouri Department of Conservation, Jefferson City.

Henderson, C.L., S. Adams and A.F. Skutch. 2002. Field Guide to the Wildlife of Costa Rica. Issue 51 of Corrie Herring Hooks Series, University of Texas Press, Austin, Texas.

Henne, C. 1967. Field investgations preliminary to life history studies on the lithosina-miona-casta complex of the genus *Annaphila* (Noctuidae). Journal of Research on the Lepidoptera, 6(4): 249-256.

Henneberry, T.J., and S.E. Naranjo. 1998. Integrated management approaches for pink bollworm in the southwestern United States. Integrated Pest Management Reviews 3(1): 31-52.

Henninger, W. F. 1910. The Macro-Lepidoptera of Seneca County, Ohio. The Ohio Naturalist 11(2): 233-242

Heppner, J.B. 1975. Biological notes on *Loxostege floridalis* (Pyralidae). Journal of the Lepidopterists’ Society 29(4): 236-242.

Heppner, J.B. 1976a. Synopsis of the genus *Parargyractis* (Lepidoptera: Pyralidae: Nymphulinae) in Florida. Florida Entomologist 59(1): 5-19.

Heppner, J.B. 1976b. *Mathildana newmanella* (Oecophoridae) in Arkansas. Journal of the Lepidopterists’ Society 30(1): 18.

Heppner, J.B. 1977. A new genus and new assignments in the American Choreutidae (Lepidoptera: Sesioidea). Proceedings of the Entomological Society of Washington 79(4): 631-636.

Heppner, J.B. 1981a. Revision of the new genus *Diploschizia* (Lepidoptera, Glyphipterigidae) for North America. Florida Entomologist 64(2): 309-336.

Heppner, J.B. 1981b. *Neomachlotica*, a new genus of Glyphipterigidae (Lepidoptera). Proceedings of the Entomological Society of Washington 83(3): 479-488.

Heppner, J.B. 1981c. Two new *Dichrorampha* (Lepidoptera: Tortricidae) from Florida. The Florida Entomologist 64(2): 271-276.

Heppner, J.B. 1981d. A new *Cydia* (Lepidoptera: Tortricidae) from Florida and Cuba. Journal of the Lepidopterists’ Society 35(4): 278-280.

Heppner, J.B. 1981e. *Acleris maccana* (Lepidoptera: Tortricidae): distribution notes and a new record for Virginia. Proceedings Entomological Society of Washington 83(4): 802-803.

Heppner, J.B. 1982a. Synopsis of the Glyphipterigidae (Lepidoptera: Copromorphoidea) of the world. Proceedings of the Entomological Society of America 84(1): 38-66.

Heppner, J.B. 1982b. Synopsis of the Hilarographini (Lepidoptera: Tortricidae) of the world. Proceedings of the Entomological Society of America 84(4): 704-715.

Heppner, J.B. 1982c. Change of name of a North American *Ypsolopha* (Lepidoptera: Plutellidae). Proceedings of the Entomological Society of Washington 84: 602.

Heppner, J.B. 1984a. Checklist: Part 1 Micropterigoidea - Immoidea, Generic Synopsis. In: J.B. Heppner, (ed.). Atlas of Neotropical Lepidoptera. Springer, Dordrecht. <https://doi.org/10.1007/978-94-009-6533-1_1>.

Heppner, J.B. 1984b. Revision of the Oriental and Nearctic genus *Ellabella* (Lepidoptera: Copromorphidae). Journal of Research on the Lepidoptera 23(1): 50-73.

Heppner, J.B. 1985. Sedge Moths of North America. (Lepidoptera: Glyphipterigidae). Flora & Fauna handbook No. 1. Flora & Fauna Publishing Gainesville, Florida.

Heppner, J.B. 1986. Revision of the new-world genus *Lotisma* (Lepidoptera, Copromorphidae). Pan-Pacific Entomologist 62(4): 273-288.

Heppner, J.B. 1988. A new species of *Ethmia* from the Florida Keys (Oecophoridae: Ethmiinae). Journal of Research on the Lepidoptera 42(4): 281-284.

Heppner, J.B. 1989. New *Argyrotaenia* and *Choristoneura* moths from Florida (Lepidoptera: Tortricidae). Florida Entomologist 72(1): 101-106.

Heppner, J.B. 1991a. A new Florida *Ethmia* moth (Lepidoptera: Oecophoridae). Tropical Lepidoptera 2(1): 69-72.

Heppner, J.B. 1991b. A new Florida *Chrysendeton* moth (Lepidoptera: Pyralidae: Nymphulinae). Tropical Lepidoptera 2(2): 119-121.

Heppner, J.B. 1991c. *Hemerophila* metalmark moths of Florida (Lepidoptera: Choreutidae). Tropical Lepidoptera 2(1): 79-84.

Heppner, J. B. 1992a. Biology and immature stages of Bromeliad Pod Borer, *Epimorius testaceellus* Ragonot (Insecta: Lepidoptera: Pyralidae), in Florida. Tropical Lepidoptera 3(1): 57-62.

Heppner, J. B. 1992b. Bromeliad Pod Borer, Epimorius testaceellus Ragonot (Lepidoptera: Pyralidae: Galleriinae). Florida Department of Consumer Services, Division of Plant Industry. Entomology Circular 351. [see update: Heppner & Frank 2016]

Heppner, J.B. 1993. Citrus leafminer, *Phyllocnistis citrella* Stainton in Florida (Insecta: Lepidoptera: Phyllocnistinae). Tropical Lepidoptera 4(1): 49-64.

Heppner, J.B. 1994. *Episimus* moths of North America (Lepidoptera: Tortricidae). Holarctic Lepidoptera 1: 83-107.

Heppner, J.B. 1995. Lacturidae, new family (Lepidoptera: Zygaenoidea). Tropical Lepidoptera 6(2): 146-148.

Heppner, J.B. 1997a. New *Diploschizia* sedge moths from Florida (Lepidoptera: Glyphipterigidae). Holarctic Lepidoptera 4(2): 70-72.

Heppner, J.B. 1997b. Immature stages of the Mediterranean napweed borer, *Pterolonche inspers*a (Lepidoptera: Pterolonchidae). Holarctic Lepidoptera 4(2): 63-66

Heppner, J.B. 1997c. New *Glyphipterix* sedge moths from the southeastern United States (Glyphipterigidae). Holarctic Lepidoptera 4: 67-69.

Heppner, J.B. 1997d. *Wockia asperipunctella* in North America (Lepidoptera: Urodidae: Galacticinae). Holarctic Lepidoptera 4(2): 73-74.

Heppner, J.B. 2000. Spanish moth, *Xanthopastis timais* (Lepidoptera: Noctuidae): A pest of amaryllis and other lilies. Florida Department Agriculture & Consumer Services, Division of Plant Industry, Entomology Circular No. 401.

Heppner, J.B. 2002. Notes on *Euchlaena* '*pectinaria*' in the United States. Lepidoptera News 2002(3-4): 6-7.

Heppner, J.B. 2003. Arthropods of Florida and neighboring land areas. Volume 17: Lepidoptera of Florida. Part 1: Introduction and catalog. Florida Department of Agriculture and Consumer Services, Gainesville, Florida. 670 pp.

Heppner, J.B. 2005. Notes on the plaster bagworm, *Phereoeca uterella,* in Florida (Lepidoptera: Tineidae). Holarctic Lepidoptera 10(1-2): 31-32.

Heppner, J.B. 2007. Lepidoptera of Florida. Part 1. Introduction and Catalog. Arthropods of Florida and neighboring land areas, volume 17, 7^th^ printing. 670 pp.

Heppner, J.B. 2008a. Florida Lepidoptera notes, 6. A new *Cydia* from Florida and southeastern United States. Lepidoptera Novae 1(3-4): 98-100.

Heppner, J.B. 2008b. Florida Lepidoptera Notes, 4. *Cacocharis albimacula* in Florida (Lepidoptera: Tortricidae). Lepidoptera Novae 1(3-4): 95.

Heppner, J.B. 2009a. *Episimus* n. sp. (a tortricid moth), a new Western Hemisphere record. Tri-ology 48(2): 2.

Heppner, J.B. 2011. Nearctic metalmark moths, 3. Genus *Anthophila* (Lepidoptera; Choreutidae: Choreutinae). Lepidoptera Novae 4(1): 11-18.

Heppner, J.B. 2019a. Florida Lepidoptera notes, 24. A new species from the Florida Keys (Lepidoptera: Tortricidae: Olethreutinae). Lepidoptera Novae 11(1-2): 65-67.

Heppner, J.B. 2019b. Reassessment of Old World *Lactura* and validity of *Enaemia* for New World species (Lepidoptera: Zygaenoidea: Lacturidae). Lepidoptera Novae 11(1-2): 7-14.

Heppner, J.B. 2020. Nearctic metalmark moths, 4. Genus *Prochoreutis* and the new genera *Protochoreutis* and *Neochoreutis* (Lepidoptera: Choreutidae: Choreutinae). Lepidoptera Novae 12(1-2): 31-46.

Heppner, J.B. 2021a. Nearctic metalmark moths, 5. The new genus Pseudocaloreas (Lepidoptera: Choreutidae: Choreutinae). Lepidoptera Novae 14: 23-28

Heppner, J.B. 2022. Nearctic metalmark moths, 6. Genus Caloreas and new genus Neocaloreas (Lepidoptera: Choreutidae: Choreutinae). Lepidoptera Novae 15(1-2): 37-88.

Heppner, J.B. 2023a. The Neotropical *Alucita eudactyla* in southern Louisiana and eastern Texas (Lepidoptera: Alucitidae). Lepidoptera Novae 16: 27-30.

Heppner, J.B. 2023b. Florida Lepidoptera notes, 28. The Caribbean *Alucita flavicincta* in Florida (Lepidoptera: Alucitidae). Lepidoptera Novae 16: 23-26.

Heppner, J.B. 2023c. *Choreutis sexfasciell*a introductions in Southern California and Florida (Lepidoptera: Choreutidae: Choreutinae). Lepidoptera Novae 16(1): 49-54.

Heppner, J.B. 2023d. Nearctic Metalmark Moths, 7. Genus *Tebenna* and new genus *Pseudotebenna* (Lepidoptera: Choreutidae: Choreutinae). Lepidoptera Novae 15(3-4): 121-172.

Heppner, J.B. and D.R. Davis. 2008. Notes on the Hawaiian *Dryadaula terpsichorella* and its presence in Florida and California (Lepidoptera: Tineidae). Lepidoptera Novae 1(1-2): 55-58.

Heppner, J.B. and T.S. Dickel. 2008. New record of *Comotia* in Florida and the United States. Lepidoptera Novae 1(3-4): 101-102.

Heppner, J.B. and W.N. Dixon. 1995. Potential spread of *Phyllocnistis citrella* (Lepidoptera: Gracillariidae: Phyllocnistinae) in the United States. American Entomologist 41(2): 110-113.

Heppner, J.B. and T.R. Fasulo. 1998 (revised 2010). Citrus leafminer, *Phyllocnistis citrella* Stainton (Insecta: Lepidoptera: Phyllocnistinae). EENY-038 (IN165). Entomology and Nematology Department, UF/IFAS Extension.

Heppner, J.B. and J.H. Frank 2016. Bromeliad Pod Borer, *Epimorius testaceellus* Ragonot (Insecta: Lepidoptera: Pyralidae). Florida Department of Consumer Services, Division of Plant Industry. Entomology Circular 351.

Heppner, J.B. and D.H. Habeck. 1976. Insects associated with *Polygonum* (Polygonaceae) in north central Florida. I. Introduction and Lepidoptera.Florida Entomologist 59: 231-239.

Heppner, J.B. and J.A. Powell, 1974. *Ethmia bipunctella* in Maryland, Pennsylvania and West Virgina: The expanding range of an introduced European moth (Gelechioidea). Journal of the Lepidopterists’ Society 28(4): 302-305.

Heppner, J.B., C. Bordelon, and E. Knudson. 2007. *Trotorhombia metachromata*: a tropical crenulate moth new to Florida and Texas.Tropical Lepidoptera 17(1-2): 34.

Herbison-Evans, D. and S. Crossley. 1995. Australian Caterpillars and their Butterflies and Moths. <http://lepidoptera.butterflyhouse.com.au> (written 10 August 1995, updated 23 February 2022). [Accessed 22 Feb 2022]

Herlihy, M.V., R.G. Van Driesche, and D.L. D. L. Wagner. 2014. Persistence in Massachusetts of the veined white butterfly due to use of the invasive form of cuckoo flower. Biological Invasions 16: 2713-2724.

Herlong, D.D. 1979. Aquatic Pyralidae (Lepidoptera: Nymphulinae) in South Carolina. Florida Entomologist 62(3): 188-193.

Hernández-Ruiz, A. C.P. Illescas-Riquelme, N. Bautista-Martínez, R. Vargas-Abasolo, J.M. Valdez-Carrasco, and P. Figueroa-Castro. 2017. Identification of fruit-piercing moths (Lepidoptera: Erebidae) and damage caused to papaya fruit in Mexico. Entomological News 126(5): 415-420.

Hetrick, L.A. 1956. Some observations on the plaster bagworm, *Tineola* Walsinghami Busck (Lepidoptera: Tineidiae). Florida Entomologist 40(4): 145-146.

Hetrick, L.A. 1960. *Nepytia semiclusaria* (Wlk.) as a defoliator of pine (Lepidoptera: Geometridae). Florida Entomologist 43(4): 205-206.

Hetz, M.W. and F.G. Werner. 1979. Insects associated with roots of some range-land compositae in southern Arizona. Southwestern Entomogist 4(4): 285-288.

Heungens, A. and E. van Daele. 1981. *Cacoecimorpha pronubana* Hb. (Tortricidae), a new parasite of *Rhododendron praecox* Carr. Mededelingen van de Faculteit Landbouwwetenschappen, Universiteit Gent 46: 581-589.

Haxaire, J. 2020. Description of a new species of Sphingidae from the USA, *Sphinx vanbuskirki* n. sp., and confirmation of the synonymy of *Sphinx coloradus* Smith, 1887 with *Sphinx dollii* Neumoegen, 1881. Lepidoptera, Sphingidae, Sphinginae. The European Entomologist 12(1): 21-36.

Heyerdahl, R.H. and J.D. Dutcher. 1990. Seasonal abundance and overwintering mortality in populations of lepidopterous leafminers of pecan. Journal of Entomological Science 25(3): 394-408

Higgins, L.G. 1981. A revision of the *Phyciodes* Hubner and related genera, with a review of the classification of the Melitaeinae (Lepidoptera: Nymphalidae). Bulletin of the British Museum (Natural History) (Entomology series) 43(3): 77-243.

Hill, D.S. 1987. Agricultural Insect Pests of Temperate Regions and Their Control. Cambridge University Press, Cambridge.

Hill, D.S. 2002. Pests of Stored Foodstuffs and Their Control. Kluwer Academic Publishers, Dordrecht, The Netherlands.

Hillier, N.K., P.L. Dixon, and D.J. Larson. 2004. Trap captures of male *Grapholita libertina* (Lepidoptera: Tortricidae) moths: relationship to larval numbers and damage in wild lingonberry. Environmental Entomology 33(2): 405-417.

Hinckley, A.D. 1964. Ecological notes on the larvae of some pyraloid moths in Fiji. Pacific Insects 6: 234-241.

Hinckley, A.D. 1972. Comparative ecology of two leafminers on white oak. Environmental Entomology 1(3): 358-361.

Hinton, H.E. 1955. The larvae of the species of Tineidae of economic importance. Bulletin of Entomological Research 47: 251-346.

Hinton, H.E. and Greenslade, R.M. 1943. Observations of species of Lepidoptera infesting stored products. XI. Notes on some moths found on bird guano. Entomologist 76: 182-184.

Hinz, L.C. Jr. and J.N. Zahniser. 2015. Review and Update of Non-mollusk Invertebrate Species in Greatest Need of Conservation: Final Report. Illinois Natural History Survey Technical Report 2015(31).

Hitchcox, M.E. and E. LaGasa. 2015. Detection of the dark strawberry tortrix, and other just desserts. Pp. 15-16 in: Research Reports 74th Annual Pacific Northwest Insect Management Conference, Hilton Hotel Portland, Oregon January 12 and 13, 2015.

Ho, K.Y. 1985. Preliminary report on the carambola fruit borers and their control. Plant Protection Bulletin of Taiwan 27: 53-62.

Hoare, R.J.B. and N. Hudson. 2018. Adventive moths (Lepidoptera) established in mainland New Zealand: additions and new identifications since 2001. The Australian Entomologist 45(3): 273-324.

Hodel, D.R., R.Y. Kim, G. Arakelian P.F. Rugman-Jones, J. Kiomen, and P. Webb. 2021. The moth *Choreutis emplecta*: Another new pest of *Ficus microcarpa* in southern California. PalmArbor 2021(11): 1-20.

Hodges, R.W. 1961. A review of the genus *Walshis* Clemens with description of new species (Lepidoptera: Gelechiodea). Bulletin of the Brooklyn Entomological Society 61: 66-80.

Hodges, R.W. 1962a. The genus *Perimede* Chambers in North America north of Mexico. Proceedings of the Entomological Society of Washington 64: 145-154.

Hodges, R.W. 1962b. A revision of the Cosmopterigidae of America north of Mexico, with description of the Momphidae and Walshiidae (Lepidoptera: Gelechioidea). Entomologica Americana 42: 1-171.

Hodges, R.W. 1964. A review of the North American moths of the family Walshiidae (Lepidoptera: Gelechiodea). Proceedings of the United States National Museum 115(3485): 289-345.

Hodges, R.W. 1965. A new genus and species of Oecophoridae (Lepidoptera: Gelechiodea). Entomological News 76: 21-25.

Hodges, R.W. 1966a. Revision of Nearctic Gelechiidae, I. The *Lita* group (Lepidoptera: Gelechiodea). Proceedings of the United States National Museum 119(3547): 1-66.

Hodges, R.W. 1966b. Review of New World species of *Batrachedra*, with description of three new genera (Lepidoptera: Gelechioidea). Transactions of the American Entomological Society (2(4): 585-651.

Hodges, R.W. 1969a. Two species of apple-feeding gelechiids new to science (Lepidoptera) Proceedings of the Entomological Society of Washington 71(2): 202-204.

Hodges, R.W., 1969b. Nearctic Walshiidae: notes and new taxa (Lepidoptera: Gelechioidea). Smithsonian Contributions to Zoology 18: 1-30.

Hodges, R.W. 1971. The Moths of North America North of Mexico including Greenland. Fascicle 21. Sphingoidea Hawkmoths. E.W. Classey Limited, Middlesex, UK and R.B.D. Publications, Inc.

Hodges, R.W. 1974a. The Moths of North America including Greenland. Fascicle 6.2. Gelechioidea, Oecophoridae. E.W. Classey Limited and R.B.D. Pub, Inc. London, UK.

Hodges, R.W. 1974b. A new species of *Aroga* (Lepidoptera: Gelechiidae) from North America. The Canadian Entomologist 106(9): 987-990.

Hodges, R.W. 1975. Oecophoridae from west Texas. Journal of the Lepidopterists’ Society 29(2): 89-94.

Hodges, R.W. 1978. The Moths of North America North of Mexico including Greenland. Fascicle 6.1. Gelechioidea, Cosmopterigidae. The Moths of North America North of Mexico including Greenland. E.W. Classey Limited and The Wedge Entomology Research Foundation. Berks, UK.

Hodges, R.W. (ed.). 1983. Check List of the Lepidoptera of America North of Mexico. E.W. Classey Ltd., Oxfordshire, England, and The Wedge Entomological Research Foundation, Washington, DC.

Hodges, R.W. 1985a. A new species of *Amplypterus* from the Chisos Mountains Texas (Lepidoptera: Sphingidae). Proceedings of the Entomological Society of Washington 87(2): 323-328.

Hodges, R.W. 1985b. A new species of *Exoteleia* (Gelechiidae) reared from ponderosa pine. Journal of the Lepidopterists' Society 39(2): 139-144.

Hodges, R.W. 1985c. A new species of *Tildenia* from Illinois (Gelechiidae). Journal of the Lepidopterists' Society

Hodges, R.W. 1986. The Moths of America North of Mexico Fascicle 7.1, Gelechioidea: Gelechiidae (part). E.W. Classey Ltd., Farington, and R.B.D. Publications Inc., Washington.

Hodges, R.W. 1992 Two new species of *Mompha* from California (Lepidoptera: Momphidae). Journal of the New York Entomological Society 100(2): 203-208.

Hodges, R.W. 1999. Gelechioidea, Gelechiidae (part). In: R.B. Dominick et al. Moths of America North of Mexico: Fascicle 7.6. Wedge Entomological Research Foundation, Washington, D.C.

Hodges, R.W. and D. Adamski. 1997. The identity of *Filatima ornatifimbriella* (Clemens 1864) (Gelechioidea: Gelechiidae). Journal of the Lepidopterists' Society 51(1): 32-46.

Hodges, R.W. and R.E. Stevens. 1978. Two new pine-feeding species of *Coleotechnites* (Gelechiidae). Journal of the Lepidopterists' Society 32(2): 118-122.

Hoebeke, E. R. 1987. *Yponomeuta cagnagella* (Lepidoptera: Yponomeutidae): A Palearctic ermine moth in the United States, with notes on its recognition, seasonal history, and habits. Annals of the Entomological Society of America 80(4): 462-467.

Hoebeke, E.R. and A.G. Wheeler, 1983. Exotic insects reported new to northeastern U.S. and eastern Canada. Journal of the New York Entomological Society 91: 193-222.

Hoebeke, E.R., A.G. Wheeler, and R.E. Degregorio. 1993. *Coleophora colutella* (Lepidoptera: Coleophoridae): A Palearctic pest of crownvetch, *Coronilla vari*a (Fabaceae), new to North America. Annals of the Entomological Society of America 86(2): 134-141.

Hoebeke, E.R., A.G. Wheeler Jr., and J.W. Brown. 2008. *Archips xylosteana* (L.) (Lepidoptera: Tortricidae), a Palearctic leafroller new to North America. Proceedings of the Entomological Society of Washington. 110: 789-795.

Hoffman, K. 2010. A crambid moth, *Duponchelia fovealis* (Zeller). Detection Advisory PD14-10, 16 July 2010 California Department of Food and Agriculture (CDFA); Plant Health and Pest Prevention Services, Pest Detection, Emergency.Projects. http://www.kernag.com/dept/news/2010/2010-san-diego-duponchelia-fovealis-07-16-2010.pdf.

Holden, D.G. 2000. Sex Pheromone Components of Satin Moth, *Leucoma salicis (*L.) (Lepidoptera: Lymantriidae). Master’s Thesis, Simon Fraser University, Vancouver, Canada.

Holland, J.N. and T.H. Fleming. 1999. Mutualistic interactions between *Upiga virescens* (Pyralidae), a pollinating seed-consumer, and *Lophocereus schottii* (Cactaceae). Ecology 80: 2074-2084.

Holland, R. 1974. Butterflies of six central New Mexico mountains, with notes on Callophrys (Sandia) macfarlandi (Lycaenidae). Journal of the Lepidopterists’ Society 28: 38-52.

Holland, R. 1995. Distribution of selected *Anthocharis*, *Euchloe* and *Pontia* (Pieridae) in New Mexico, Texas, Chihuahua and Sonora. Journal of the Lepidopterists’ Society 49(2): 119-135.

Holland, W.J. 1886. Some notes upon Sphingidae of the United States. Canadian Entomologist 18: 101-105.

Holland, W.J. 1903. The Moth Book. A Popular Guide to a Knowledge of the Moths of North America. Doubleday, Page and Company, New York. 479 pp.

Holland, W.J. 1914. The Butterfly Book: A Popular Guide to a Knowledge of the Butterflies of North America. Doubleday, Garden City, New York.

Holland, W.J. 1915. The Butterfly Book: A Popular Guide to a Knowledge of the Butterflies of North America. Doubleday, Page and Company. Garden City, New York.

Holland, W.J. 1916. The Butterfly Book: A Popular Guide to a Knowledge of the Butterflies of North America. Doubleday, Garden City, New York.

Holland, W.J. 1922. The Butterfly Book: A Popular Guide to a Knowledge of the Butterflies of North America. Doubleday, Garden City, New York.

Holland, W.J. 1934. The Moth Book. Double Day, Doran & Co., New York, New York.

Holland, W.J. and W. Schaus. 1925. The Epipaschiinae of the Western Hemisphere; a synonomic catalog of the species hitherto described, with figures of many, which have not heaetofore been depicted. Annals of the Carnegie Museum 16(1): 49-131.

Holloway, J.D. 1993. The moths of Borneo 11: Family Geometridae, subfamily Ennominae. Malayan Nature Journal 47: 1-309.

Homziak, N., H. Hopkins, and K.B. Miller. 2015. Revision of the genus *Heteranassa* Smith, 1899 (Lepidoptera, Erebidae, Omopterini). In: B.C. Schmidt and J.D. Lafontaine, (Eds.). Contributions to the systematics of New World macromoths VI. ZooKeys 527: 31-49. doi: 10.3897/zookeys.527.8771.

Hooper, R. 2007. Checklist of Saskatchewan moths: Part 18-Geometridae (4), Larentiinae. Blue Jay 65(3): 148-157.

Horak, M. 2003. Reassessment of the Anerastiini and their status in the Phycitinae (Pyralidae): a century-long controversy. Invertebrate Systematics 17(1): 89-98.

Horak, M. 2006. Olethreutinae moths of Australia (Lepidoptera: Tortricidae). Monographs on Australian Lepidoptera 10. 522 pp.

Hosseinzade, S., H. Izadi, P. Namvar, and M.A. Samih. 2014. Biology, temperature thresholds, and degree-day requirements for development of the cucumber moth, *Diaphania indica*, under laboratory conditions. Journal of Insect Science 14(61). <http://www.insectscience.org/14.61>

Howard, L.O. 1885. Descriptions of North American Chalcididae from the Collections of the U.S. Department of Agriculture and of Dr. C.V. Riley: with Biological Notes. First Paper. Together with a List of the Described North American Species of the Family. United States Department of Agriculture, Bureau of Entomology Bulletin No. 5. 47 pp.

Howe, W.H. (ed.). 1975. The Butterflies of North America. Doubleday & Co., New York, NY.

Hoyt, C.A. 2014. The spotless comma (*Polygonia haroldii*): a new species for the United States. News of the Lepidopterists’ Society 56(1): 14-15.

Hribar, L.J. 2005. Locality records for some Lepidoptera in the Florida Keys. Florida Scientist 68(1): 8-10.

Hribar, L.J. 2024. A specimen of *Ancylis virididorsana* (Tortricidae: Olethreutinae) from Long Key, Monroe County, Florida. Southern Lepidopterists’ News, 46: 207-208.

Hsu, Y.F. and J.A. Powell. 2005. Phylogenetic relationships within Heliodinidae and systematics of moths formerly assigned to Heliodines Stainton (Lepidoptera: Yponomeutoidea). University of California Publications in Entomology 124: 1-213.

Hu, G.L., C.M. Zhang, Z.Q. Wang, Q. X. Chen, and J.Q. Lu, 2021. Sensilla of the antenna and proboscis of *Athetis lepigone* (Möschler) (Lepidoptera: Noctuidae). Journal of Morphology 282(5): 733-745.

Huang, F. and B. Subramanyam. 2004. Responses of *Corcyra cephalonica* (Stainton) to pirimiphos‐methyl, spinosad, and combinations of pirimiphos‐methyl and synergized pyrethrins. Pest Management Science 60(2): 191-198.

Huang, G.H., L.S. Chen, T, Hirowatari, Y. Nasu, and M. Wang. 2011. A revision of the *Monopis monachella* species complex (Lepidoptera: Tineidae) from China. Zoological Journal of the Linnean Society 163(1): 1-14.

Hübner, J. 1819 [dated 1816]. Schmettlinge. Lepidoptera. Lepidoptera Linnei, Glossata Fabricii. Berzechniss befannter Schmettlinge 17-179.

Hübner, J. 1823. Zutrage zur Sammlung exotischer Schmettlinge, Beſtehend in Bekundigung einzelner Fliegmuster neuer oder rarer nichteuropaischer Gattungen. Zutrage zur Sammlung erotischer Schmettlinge, Second Hundred: 7-32 (plus plates).

Hudon, M. and J.P. Perron. 1970. First record of *Cisseps fulvicollis* (Lepidoptera: Amatidae) as an economic destructive insect on grain corn in Canada. The Canadian Entomologist 102(8): 1052-1054.

Huemer, P. 1988. A taxonomic revision of *Caryocolum* (Lepidoptera: Gelechiidae). Bulletin of the British Museum (Natural History) Entomology 57: 439-571.

Huemer, P. 1993. Review of the *Incurvaria vetulella* species-group in the Alps (Lepidoptera: Incurvariidae). Insect Systematics & Evolution [Entomologica Scandinavica] 24(1), 109-120.

Huemer, P. and O. Karsholt. 1999. Microlepidoptera of Europe. Volume 3 Gelechiidae I (Gelechiinae: Teleiodini, Gelechiini). Apollo Books, Stenstrup, Denmark.

Huemer, P. and O. Karsholt. 2010. Gelechiidae II (Gelechiinae: Gnorimoschemini). In: P. Huemer, O. Karsholt, and M. Nuss, (Eds.), Microlepidoptera of Europe, 6. Apollo Books, Stenstrup, Denmark. 586 pp.

Huemer, P, and M. Mutanen.2015. Alpha taxonomy of the genus *Kessleria* Nowicki, 1864, revisited in light of DNA-barcoding (Lepidoptera, Yponomeutidae). ZooKeys 503: 89-133. doi: 10.3897/zookeys.503.9590

Huertas Dionisio, M. 2002. Lepidópteros de Huelva (I). Especies detectadas en las márgenes del río Guadiana. Boletín de la Sociedad Andaluza de Entomología 4: 9-29.

Huisman, K.J. 2012. The micro moth genus *Agonopterix* in the Netherlands (Lepidoptera: Elachistidae: Depressariinae). Nederlandse Faunistische Mededelingen 37: 45-104.

Huisman, K.J., J.C. Koster, E.J. van Nieukerken, and W.N. Ellis. 2007. Microlepidoptera in Nederland in 2005. Entomologische Berichten 67(1-2): 34-47.

Hulst, G.D. 1879-1880. *Macroglossa thysbe*, Fabricus. Bulletin of the Brooklyn Entomological Society 2: 38-40.

Hulst, G.D. 1881. Descriptions of some new species of North American Lepidoptera. Bulletin of the Brooklyn Entomology Scoiety 3(9): 75-77.

Hulst, G.D. 1886a. Descriptions of new Pyralidae. Transactions of the American Entomological Society 13: 145-168.

Hulst, G.D. 1886b. New Species of Geometridae, No. 2. Entomologica Americana 2(6): 120-124.

Hulst, G.D. 1888. New genera and species of Epipaschiae and Phycitidae. Entomologica Americana 4: 113-118.

Hulst, G.D. 1889. The Epipaschiinae of North America. Entomologica Americana 5(4): 62-77.

Hulst, G.D. 1890. The Phycitidae of North America Transactions of the American Entomological Society 17(2): 93-228.

Hulst, G.D. 1898a. Descriptions of new genera and species of Geometrina of North America. The Canadian Entomologist 30(8): 214-220.

Hulst, G.D. 1900. Some new genera and species of Phycitinae. The Canadian Entomologist 62(6): 169-184.

Humble L.M., J.R. DeWaard, and M. Quinn. 2009. Delayed recognition of the European poplar shoot borer, Gypsonoma aceriana (Duponchel) (Lepidoptera: Tortricidae), in Canada. Journal of the Entomological Society of British Columbia 106: 61−70.

Hunter, W.D., F.C. Pratt, and J.D. Mitchell. 1912. The Principal Cactus Insects of the United States. Bureau of Entomology Bulletin No. 113. U.S. Department of Agriculture.

Huseth, A.S., R.L. Koch, D. Reisig, J.A. Davis, S. V. Paula-Moraes, and E.W. Hodgson. 2021. Current distribution and population persistence of five lepidopteran pests in US soybean. Journal of Integrated Pest Management 12(1): 11.

Hussain, M.A., T. Ahmad, and T. Gilligan. 2021. Insect and avian threats to the industrial production of balanites fruit in Eritrea. International Journal of Tropical Insect Science 41: 1017-1025.

Hutchinson, C.S. and G.B. Seymour. 1982. *Poa annua* L. Journal of Ecology 70(3): 887-901.

Hyatt, J.A. 2004. Moths of a Small Island on the Coast of Georgia (Lepidoptera, Heterocera). Atalanta 35(3/4): 453-465.

ICZN. 2012. Opinion 2291 (Case 3524) *Thecla dumetorum* Boisduval, 1852 (currently *Callophrys dumetorum*), proposed neotype; and *Thecla sheridonii* Carpenter, 1877 (currently *C. sheridanii*) (Lepidoptera, Lycaenidae): current usage and names conserved. Bulletin of Zoological Nomenclature 69(1): 69-71.

Igarashi, S. 1979. Papilionidae and their early stages. 2 volumes. Kodansha, Tokyo.

Igarashi, S. & Fukuda, H., 2000. The life histories of Asian butterflies, Vol. 2. Tokyo University Press, Tokyo.

INHS:INHSIC. 2015. Illinois Natural History Survey Insect Collection, University of Illinois, Champaign, Illinois. In: SCAN. P.L. Heinrich, E. Gilbert, N.S. Cobb, and N. Franz. Symbiota collections of arthropods network (SCAN): A data portal built to visualize, manipulate, and export species occurrences. [Dataset]. http://scan-bugs.org/portal/collections/index.php [Accessed 08 March 2019]

Inoue, H. 1956. A revision of the Japanese Lymantriidae (I). Japanese Journal of Medical Science and Biology.9: 133-188.

Irwin, R.R. and J.C. Downey. 1973. Annotated Checklist of the Butterflies of Illinois. Illinois Natural History Survey. Urbana, Illinois & State Of Illinois, Department of Registration and Education, Natural History Survey Division.

Iruegas-Buentello, H.R., I.G. López-Muraira, H. Flores-Martínez, and F. Gómez-Leyva. 2018. Nuevos registros de Spilomelinae a la fauna de Lepidoptera Mexicanos (Lepidoptera: Crambidae). SHILAP Revista de lepidopterología 46(183): 415-418.

Isley, D. and F.D. Miner. 1944. The lesser cornstalk borer, a pest of fall beans. Journal of the Kansas Entomological Society 17: 51-57.

Ives, W.G.H. and H.R. Wong. 1988. Tree and shrub insects of the prairie provinces. Canadian Forest Service, Northwest Region, Edmonton, AB, Information Report NOR-X-292. 327 pp.

Ivinskis, P. 1982. 138 Lepidoptera species new to the Lithuanian SSR, found in 1968-1982. Pp 28-47 In: V. Jonaitis, (Ed.). New and rare for the Lithuanian SSR insect species. Reports and Descriptions of 1982, Vol. 2. Vilnius, Lithuania.

Jackson, R.V. 1996. Lepidoptera breeding records from *Alphitonia species* (Rhamnaceae) at Paluma, North Queensland. Australian Entomologist 23: 75-76.

Jacobs, S.N.A. 1957. Current notes. The Entomologist’s Record and Journal of Variation 69: 170.

Jacobs, S.N.A. 1978. *Cacoecimorpha pronubana* Hubner (Lep., Tortricidae): a greenhouse pest. Entomologist’s Record and Journal of Variation 90: 266.

Jaeger, C.M., J.J. Dombroskie, and F.A. Sperling. 2013. Delimitation of *Phaneta tarandana* (Möschler 1874) and *P. montanana* (Walsingham 1884) (Tortricidae: Olethreutinae) in western Canada using morphology and DNA. Journal of the Lepidopterists’ Society, 67(4), 253-262.

Jahner, J.P., M.L. Forister, C.C. Nice, J. A. Fordyce, J.S. Wilson, D.D. Murphy, Z.H. Marion and A.M. Shapiro. 2015 Regional population differentiation in the morphologically diverse, elevationally widespread Nearctic skipper *Polites sabuleti*. Journal of Biogeography 42: 1787-1799.

James, D.G. 2008. Comparative studies on the immature stages and developmental biology of five *Argynnis* spp. (Subgenus Speyeria) (Nymphalidae) from Washington. Journal of the Lepidopterists’ Society 62(2): 61-70.

James, D.G. 2009. Comparative studies on the immature stages and biology of *Hesperia colorado idaho* and *Hesperia juba* (Hesperiidae). Journal of the Lepidopterists’ Society 63(3): 129-136.

James, D.G. 2012. Observations on the life history and field biology of an imperiled butterfly *Philotiella leona* (Lepidoptera: Lycaenidae) from South Central Oregon. Journal for Research on the Lepidoptera 45: 93-99.

Jansen, M.G.M. 2005. The Lepidoptera fauna of three brackish salt marshes including two species new for the Belgian fauna (Lepidoptera). Phegea 33(2): 59-68.

Jansen, M. 2008. The Lepidoptera of the salt marshes of Het Zwin including two species new for the Belgian fauna (Lepidoptera). Phegea 36(3): 109-118.

Jantscher, T.A. 2016. 2015 Season Summary. Zone 8 Midwest, Missouri, Kentucky, West Virginia, Ohio, Indiana, Illinois, Iowa, Minnesota, Wisconsin, Michigan. News of the Lepidopterists’ Society 58(S1): 159-205.

Jantscher, T.A. 2017. 2016 Season Summary. Zone 8 Midwest, Missouri, Kentucky, West Virginia, Ohio, Indiana, Illinois, Iowa, Wisconsin, Minnesota, Michigan. News of the Lepidopterists’ Society 58(S1): 130-177.

Janzen, D.H. 1985. A host plant is more than its chemisty. Illisonis Natural History Symposium Bulletin 33(3); 141-174.

Janzen, D.H. and W. Hallwachs. 2009. Dynamic database for an inventory of the macrocaterpillar fauna, and its food plants and parasitoids, of Area de Conservacion Guanacaste (ACG), northwestern Costa Rica. <http://janzen.sas.upenn.edu>. [*Elasmia mandela* Accessed 6-July-2014] [*Gorgythion begga* Accessed 6-December-2014] [*Lerema liris* Accessed 25 April 2018] [*Astraptes anaphus, Astraptes egregius, Pellicia arina, Pellicia dimidiata* Accessed 18-June 2019] [*Eumorpha megaeacus* Accessed 20-Nov-2019] ] [*Protambulyx strigilis* Accessed 21-Oct-2020] [Elasmia mandela Accessed 6-July-2014] [*Protambulyx strigilis Accessed 21-Oct-2020] [Urbanus esmeraldus, Synapte salenus, Sostrata nordic*a & *Noctuana stator* Accessed 14-Aug-2021]

Janzen, D.H., M.J. Sharkey, and J.M. Burns. 1998. Parasitization biology of a new species of Braconidae (Hymenoptera) feeding on larvae of Costa Rican dry forest skippers (Lepidoptera: Hesperiidae: Pyrginae). Tropical Lepidoptera 9 (Suppl. 2): 33-41.

Janzen, D.H., W. Hallwachs, D.J. Harvey, K. Darrow, R. Rougerie, M. Hajibabaei, M.A. Smith, C. Bertrand, I.C. Gamboa, B. Espinoza, J.B. Sullivan, T. Decaens, D. Herbin, L.F. Chavarria, R. Franco, H. Cambronero, S. Rios, F. Quesada, G. Pereira, J. Vargas, A. Guadamuz, R. Espinoza, J. Hernandez, S. Rios, E. Cantillano, R. Moraga, C. Moraga, P. Rios, M. Rios, R. Calero, D. Martinez, D. Briceño, M. Carmona, E. Apu, K. Aragon, C. Umaña, J. Perez, A. Cordoba, P. Umaña, G. Sihezar, O. Espinoza, C. Cano, E. Araya, D. Garcia, H. Ramirez, M. Pereira, J. Cortez, M. Pereira, W. Medina, and P.D.N. Hebert. 2012. What happens to the traditional taxonomy when a well-known tropical saturniid moth fauna is DNA barcoded? Invertebrate Systematics 26: 478-505.

Jaros, J. and K. Spitzer. 2002. Food plants of Lepidoptera associated with an alder carr forest in South Bohemia (Central Europe). Sborník Jihočeského Muzea v Českých Budějovicích, Přírodní Vědy - Supplementum 42: 5-60.

Javala, J. and W.E. Miller. 1998. Boreal Olethreutini 1. (Lepidoptera: Tortricidae): New synonymies and Holarctic records. Entomologica Fennica 9: 137-142.

Jaworski, T. 2018. Chapter 10, Diversity of Saproxylic Lepidoptera. Pp 319-338 In: M.D. Ulyshen (ed.). Saproxylic Insects. Zoological Monographs 1. <https://doi.org/10.1007/978-3-319-75937-1_10>.

Jaworski, T., J. Hilszczański, R. Plewa, and A. Szczepkowski. 2014. Fungus moths (Lepidoptera, Tineidae) of the Białowieża Forest. Polish Journal of Entomology 83(1): 5-21.

Jenkins, D.W. 1984. *Hamadryas* in the United States (Nymphalidae). Journal of the Lepidopterists' Society 38(3): 171-175.

Jenkins, D.W. 1990. Neotropical Nymphalidae VIII. Revision of *Eunica*. Bulletin of the Allyn Museum 131: 1-177.

Jewett, H.S. 1880. Notes on Lepidoptera. Canadian Entomologist 12: 228-231.

Jewett, H.S. 1881. Notes on *Hemaris marginalis*, Grote. Bulletin of the Buffalo Society of Natural Sciences 4: 17-19.

Jez, M. and R. Verovnik. 2012). O Pojavljanju in Ogroæenosti Borovniœevega Mnogooka (*Plebejus optilete* (Knoch, 1781 (Lepidoptera: Lycaenidae) v Sloveniji. Acta Entomologica Slovenica, 20(2): 125-134.

Jinbo, U. 2003. Records of three archipine species (Tortricidae, Tortricinae) little known from Japan. Japan Heterocerists’ Journal 222: 417-422.

Johansson, R., E.S. Neilsen, E.J. van Nieukerken and B. Gustafsson. 1990. The Nepticulidae and Opostegidae (Lepidoptera) of North West Europe. Fauna Entomologica Scandinavica 23(1): 1-413.

Johnson, E.F., J.E. Laing, and R. Trottier. 1976. The seasonal occurrence of *Lithocolletis blancardella* (Gracillariidae) and its major enemies in Ontario apple orchards. Proceedings of the Entomological Society of Ontario 107: 31-45.

Johnson, J.B. and M.P. Stafford. 1985. Adult Noctuidae feeding on aphid honeydew and a discussion of honeydew feeding by adult Lepidoptera. Journal of the Lepidopterists' Society 39(4): 321-327.

Johnson, J.W. 1981. A desert subspecies of *Gloveria medusa* (Lasiocampidae). Journal of the Lepidopterists' Society 35: 147-154.

Johnson, J.W. and E. Walter. 1984. The immature stages of *Catocala erichi* Brower (Lepidoptera: Noctuidae). Journal of Research on the Lepidoptera 23: 231-235.

Johnson, K. 1972. *Juniperus* (Cupressaceae) speciation and the ranges and evolution of two *Callophrys* (Lycaenidae). Journal of the Lepidopterists' Society 26(2): 112-122.

Johnson, K. 1989. Revision of *Chlorostrymon* Clench and description of two new austral Neotropical species (Lycaenidae). Journal of the Lepidopterists' Society 43: 120-146.

Johnson, K. 1992. A new lichen moth record for the United States: *Lycomorphodes sordida* (Arctiidae: Lithosiinae) from south Texas. Journal of the Lepidopterists' Society 46(2): 161.

Johnson, K.J.R. 1984. Identification of *Eoreuma loftini* (Dyar) (Lepidoptera: Pyralidae) in Texas, 1980: forerunner for other sugarcane boring pest immigrants from Mexico. Bulletin of the Entomological Society of America 30(3): 47-52.

Johnson, R.L., P.J. Van Dusen, J.A. Toner, and N.G. Hairston, Jr. 2000. Eurasian watermilfoil biomass associated with insect herbivores in New York. Journal of Aquatic Plant Management 38: 82-88.

Johnson, W.J. 1984[1985]. Immature stages of six California *Catocala* (Lepidoptera, Noctuidae). Journal of Research on the Lepidoptera 23(4): 303-327.

Johnson, W.T., and H.H. Lyon, 1991. Insects that Feed on Trees and Shrubs. Comstock Publishing Company, Ithaca, New York and London, United Kingdom.

Jones, D.A. and R. Turkington. 1986. *Lotus corniculatus* L. Journal of Ecology 74(4): 1185-1212.

Jones, J.R.J.L. 1951. An annotated check list of the Macrolepidoptera of British Columbia. Occasional Papers of the Society of Entomology of British Columbia, 1. 148 pp.

Jones, R.M. 1937. *Cirrophanus triangulifer* Grote (Lepidoptera, Noctuidae) in Delaware. Entomological News 48: 121-124.

Jones, F.M. and C.P. Kimball. 1943. The Lepidoptera of Nantucket and Marthas Vineyard Islands, Massachusetts. Nantucket Maria Mitchell Association, Nantucket, Massachusetts.

Joshi, N.K., D.J. Biddinger, S. Fleischer, and S. Passoa. 2013. First report of the adventive species *Sitochroa palealis* (Lepidoptera: Crambidae) in Pennsylvania and its attraction to the sex pheromone of the European Corn Borer, *Ostrinia nubilalis* (Lepidoptera: Crambidae). Great Lake Entomologist 46(1/2): 99-103.

Julian, J.J. 1991. First record of *Rhyacionia adana/jenningsi* complex in Colorado. Southwestern Entomologist 16(3): 283-284.

Kaila, L. 1992. The Elachistidae of southern Siberia and Central Asia, with descriptions of five new species (Lepidoptera). Entomologica Fennica 3: 177-194.

Kaila, L. 1995a. A review of *Coelopoeta* (Elachistidae), with descriptions of two new species. Journal of the Lepidopterists’ Society 49(2): 171-178.

Kaila, L. 1995b. A revision of the North American *Perittia* (= *Onceroptila*), with first Nearctllc records of the genus *Mendesia* (Elachistidae). Journal of the Lepidopterists' Society 49(3): 208-222.

Kaila, L. 1996. Revision of the Nearctic species of *Elachista* s. 1. II. Insect Systematics & Evolution 27(2): 217-238.

Kaila, L. 1997. Revision of the Nearctic species of *Elachista* s. 1. II. The *argentella* group (Lepidoptera, Elachistidae). Acta Zoologica Fennica 206: 1-93.

Kaila, L. 1999a. A revision of the Nearctic species of the genus *Elachista* s. l. III. The *bifasciella, praelineata, saccharella*, and *freyerella* groups (Lepidoptera, Elachistidae). Acta Zoologica Fennica 211: 1-235.

Kaila, L. 1999b. Phylogeny and classification of the Elachistidae s.s. (Lepidoptera: Gelechioidea). Systematic Entomology 24: 139-169.

Kaila, L. 2019. An annotated catalogue of Elachistinae of the World (Lepidoptera: Gelechioidea: Elachistidae). Zootaxa 4632(1): 1-231.

Kaila, L. 2024. A review of Coelopoetinae (Lepidoptera, Gelechioidea, Pterolonchidae), a moth subfamily confined to western North America, with descriptions of seven new species. Zootaxa 5458(3): 361-384.

Kaitila, J. 2005. Paahdeympäristöjen perhosista. Teoksessa: Paahdeympäristöjen Ekologia ja Uhanalaiset Lajit. Suomen Ympäristö. The Finnish Environment 774. 86 pp.

Kamijo, K., F. Komai, and S. Suzuki. 1983. [Pests of *Rosa rugosa* Thunberg.] Koshunai-Kihou 55: 17-21.

Karsholt, O. and E.S. Nielsen. 1976. Notes on some Lepidoptera described by Linnaeus, Fabricius, and Ström. Insect Systematics & Evolution 7(4):241-251.

Karsholt, O. and J. Razowski. 1996. The Lepidoptera of Europe: A Distributional Checklist. Apollo Books, Naperville, llinois.

Karsholt, O. and S.Y. Sinev. 2004. Contribution to the Lepidoptera fauna of the Madeira Islands, Part 4. Blastobasidae. Beiträge zur Entomologie 54(2): 387-463.

Karsholt, O. and E.J. van Nieukerken, (eds.). 2004. Lepidoptera, Moths. Fauna Europaea version 1.1, http: // [www.faunaeur.org](http://www.faunaeur.org/).

Karsholt, O. L. Aarvik, D. Agassiz, P. Huemer, and K. Tuck. 2005. *Acleris effractana* (Hübner, 1799) - a Holarctic Tortricid. Nota Lepidopterologica 28(2): 93-102.

Karsholt, O. N.P. Kristensen, T.J. Simonsen & M. Ahola. 2015. Chapter 15, Lepidoptera (mothes and butterflies). 302-256 In: J. Böcher, N.P. Kristensen, T. Pape, L. Vilhelmsen, (eds.). The Greenland Entomofauna: An Identification Manual of Insects, Spiders and Their Allies. Fauna Entomologica Scandinavica Book 44, Brill Academic Publishing, Leiden, The Netherlands.

Kartesz, J.T. 2015. The Biota of North America Program (BONAP). Taxonomic Data Center. (http://www.bonap.net/tdc). Chapel Hill, N.C. [maps generated from Kartesz, J.T. 2015. Floristic Synthesis of North America, Version 1.0. Biota of North America Program (BONAP). [Accessed 2015 trhough Feb 2025].

Kaupp, W.J., K.N. Barber, W. E. Fick, P.M. Ebling, T.R. Ladd, and S.B. Holmes. 2011. Host-range testing of a mixture of two nucleopolyhedroviruses of *Choristoneura fumiferana* (Lepidoptera: Tortricidae). The Canadian Entomologist 143(2): 165-177.

Kawabe, A. 1982. Tortricidae and Cochylidae. Pp. 62-151. In: H.S. Inoue, S. Sugi, H. Kuroko, S. Moriuti and A. Kawabe, (eds.). The Moths of Japan, Part 1: 62-258, Part 2: 158-183.

Kawahara, A.Y., K. Nishida, and D. Rubinoff. 2011. Behavior of the Hawaiian Dancing Moth, *Dryadaula terpsichorella* (Tineidae: Dryadaulinae). Journal of the Lepidopterists Society 65(2): 133-135.

Kearfott, W.D. 1902. A revision of the North American species of the genus *Choreutis*. Journal of the New York Entomological Society 10: 106-125.

Kearfott, W.D. 1903. Descriptions of new Tineoidea Journal of the New York Entomological Society 11(3): 145-164 & pl. 9.

Kearfott, W.D. 1905a. Descriptions of new Tortricid moths, from North Carolina, with notes. Proceedings of the United States National Museum 27: 349-364.

Kearfott, W.D. 1905b. Manitoba Microlepidoptera. The Canadian Entomologist 37(6): 205-209.

Kearfott, W.D. 1905c. Manitoba Microlepidoptera. The Canadian Entomologist 37(8): 293-296.

Kearfott, W.D. 1907a. New North American Tortricidae. Transactions of the American Entomological Society 33: 1-98.

Kearfott, W.D. 1907b. Article VIII. Mircrolepidoptera from the Black Mountain region of North Carolina, with description of new species. Bulletin of the American Museum of Natural History 23: 153-167 + 1 plate.

Kearfott, W.D. 1907c. New Microlepidoptera. The Canadian Entomologist 34(1): 1-9.

Kearfott, W.D. 1907d. New Microlepidoptera. The Canadian Entomologist 34(1): 211-212.

Kearfott, W.D. 1907d. New Microlepidoptera. The Canadian Entomologist 34(3): 77-84.

Kearfott, W.D. 1908. Descriptions of new species of North American crambid moths. Proceedings of the United States National Museum 35(1649): 367-393.

Kearfott, W.D. 1910. A new species of Japanese Mico-Lepidoptera. Xanadian Entomologist 62: 346- 348.

Keathley, C.P., L.L. Stelinski and S.L. Lapointe. 2013. Attraction of a native florida leafminer, *Phyllocnistis insignis* (Lepidoptera: Gracillariidae), to pheromone of an invasive citrus leafminer, *P. citrella*: evidence for mating disruption of a native non-target species. Florida Entomologist 96(3): 877-886.

Keegan, K.L. and D.L. Wagner. 2022. A preliminary molecular phylogeny for *Stiria* (Noctuidae, Stiriinae) and description of a new species from Texas. The Journal of the Lepidopterists' Society 76(3): 175-182.

Keegan, K.L., J.D. Lafontaine, N. Wahlberg, and D.L. Wagner. 2019. Towards resolving and redefining Amphipyrinae (Lepidoptera, Noctuoidea, Noctuidae): A massively polyphyletic taxon. Systematic Entomology 44(2): 451-464.

Keen, F.P. 1956. Insect enemies of western forests. United States Department of Agriculture Miscellaneous publication 273.

Keen, F.P. 1958. Cone and seed insects of western forest trees. United States Department of Agriculture Technical Bulletin 1169. 177 pp.

Keifer, H.H. 1931. California Microlepidoptera V. Pan-Pacific Entomologist 8: 61-73.

Keifer, H.H. 1933. California Microlepidoptera VI. The Monthly Bulletin, Department of Agriculture, State of California 22(7-11): 351-365.

Keifer, H.H. 1935. California Microlepidoptera VII. The Monthly Bulletin, Department of Agriculture, State of California 24(4-6): 195-218

Keifer, H.H. 1936a. California Microlepidoptera VIII. Bulletin of the Southern California Academy of Sciences 35: 9-29.

Keifer, H.H. 1936b. California Microlepidoptera IX. The Monthly Bulletin, Department of Agriculture, State of California 25(2): 235-259.

Keifer, H.H. 1936c. California Microlepidoptera X. The Monthly Bulletin, Department of Agriculture, State of California 25(3): 349-359.

Keifer, H.H. 1937a. California microlepidoptera XI. The Monthly Bulletin, Department of Agriculture, State of California 26(2): 177-203.

Keifer, H.H. 1937b. California Microlepidoptera XII. The Monthly Bulletin, Department of Agriculture, State of California 26(3): 334-338.

Keifer, H.H. 1944. Systematic entomology. In H.M. Armitage (ed.). Annual Report of the Bureau of Entomology and Plant Quarantine. California Department of Agriculture Bulletin 33: 248-252.

Kendall, D. M., P.G. Kevan, and J.D. LaFontaine. 1981. Nocturnal flight activity of moths (Lepidoptera) in alpine tundra. The Canadian Entomologist 113(7): 607-614.

Kendall, R.O. 1970. A day-flying moth (Pericopidae) new to Texas and the United States. Journal of the Lepidopterists' Society 24(4): 301-303.

Kendall, R.O. 1974. Two moth species (Pericopidae and Notodontidae) new to Texas and the United States. Journal of the Lepidopterists' Society (28(3): 243-245.

Kendall, R.O. 1975. Larval food plants for seven species of hairstreaks (Lycaenidae) from Mexico. Bulletin of the Allyn Museum 24: 1-4.

Kendall, R.O. 1976a. Larval foodplants and life history notes for some metalmarks (Lepidoptera: Riodinidae) from Mexico and Texas. Bulletin of the Allyn Museum 32: 1-12.

Kendall, R.O. 1976b. Larval foodplants and life history notes for eight moths from Texas and Mexico. Journal of the Lepidopterists' Society 30(4): 264-271.

Kendall, R.O. 1976c. Larval foodplants for thirty species of skippers Lepidoptera: Hesperiidae) from Mexico. Bulletin of the Allyn Museum 39: 1-9.

Kendall, R.O. 1978. Periodic occurrence of Urania fulgens (Uraniidae) in the United States. Journal of the Lepidopterists' Society 32(4): 307-309.

Kendall, R.O. and W.W. McGuire. 1975. Larval food plants for twenty-one species of skippers (Lepidoptera: Hesperiidae) from Mexico. Bulletin of the Allyn Museum 27: 1-7.

Kendall, R.O. and W.W. McGuire. 1984. Some new and rare records of Lepidoptera found in Texas. Bulletin of the Allyn Museum 86: 1-50.

Kendall, R.O. and M.A. Rickard. 1976. Larval foodplants, spatial and temporal distribution for five skippers (Hesperiidae) from Texas. Journal of the Lepidopterists' Society 30(2): 105-110.

Kennedy, P.C. and L.F. Wilson. 1969. Major insect pests in North Dakota shelterbelts; abundance and distribution by climate and host age. U.S.D.A. Forest Service research Paper RM-47. 12pp.

Kennel, J. von. 1908. Die Palaerktischen Torticiden. Zoologica, 21: 1-742.

Kephart, S., R.J. Reynolds, M.T. Rutter, C.B. Fenster, and M.R. Dudash. 2006. Pollination and seed predation by moths on Silene and allied Caryophyllaceae: evaluating a model system to study the evolution of mutualisms. New Phytologist 169: 667-680.

Kerns, D.L., D.G. Wright, and J. Loghry. 2002. Citrus Leafminer (*Phyllocnistis citrella*) In: D.L. Kerns, G.C. Wright, and J. Loghry. Citrus Arthropod Pest Management in Arizona. Arizona Department of Agriculture, Phoenix, Arizona. URL: <http://agriculture.state.az.us/CD&P/citrusipmtext2.pdf>

Kesting-Handly, T. and S. Kloiber. 2019. New host and county records, with a breeding range extension for Protambulyx strigilis (Lepidoptera: Sphingidae). News of the Lepidopterists’ Society 61(1): 38-43.

KEW. 2025. Kew Royal Botanic Gardens. Plants of the World Online <https://powo.science.kew.org> [accessed Jan 2025]

Khlyzova, T.A. and S.A. Kozlov. 2021. Geographical distribution of Satyridae (Lepidoptera, Satyridae) of the Kurgan Oblast. IOP Conference Series: Earth and Environmental Science 839: 042008.

Kim, S.S., C.M. Lee, T.S. Kwon, H.Z. Joo, and J.H. Sung. 2012. Korean Butterfly Atlas [1996~2011]. National Academy of Forest Sciences, 2012.11.

Kimball, C.P. 1953. A proposed revision of the check-list of Florida Lepidoptera. Florida Entomologist 36(3): 103-107.

Kimball, C.P. 1965. Arthropods of Florida and neighboring land areas, 1: Lepidoptera of Florida, an annotated checklist. Gainesville, FL.

Kimmich, H.P. 1966. Notes on the biology of three arctiid moths from British Columbia. Journal of the Entomological Society of British Columbia 63: 10-13.

King, D. R., J.A. Harding, and B C. Langley. 1961. Peanut insects in Texas. Texas Agricultural Experimental Station Miscellaneous Publication 550.

Kingsley, K.J. 1998. Invertebrates of Organ Pipe Cactus National Monument, Arizona. US Geological Survey, Cooperative Park Studies Unit and University of Arizona Tucson, Arizona. Technical Report N. 60.

Kinser, D.P. and H.H. Neunzig. 1981. Description of the immature stages and biology of Synclita tinealis Munroe (Lepidoptera: Pyralidae: Nymphulinae). Journal of the Lepidopterists’ Society, 35(2): 137-146.

Kirichenko N., P. Triberti, M. Mutanen, E. Magnoux, J.-F. Landry, and C. Lopez-Vaamonde. 2016. Systematics and biology of some species of *Micrurapteryx* Spuler (Lepidoptera, Gracillariidae) from the Holarctic Region, with redescription of M. caraganella (Hering) from Siberia. ZooKeys 579: 99-156.

Kirichenko, N.I., N.A. Kolyada, and S. Gomboc. 2023. First discovery of the North American leaf-mining moth *Chrysaster ostensackenella* (Lepidoptera: Gracillariidae) in Russia: The genetic diversity of a novel pest in invaded vs. native range. Insects 14: 642; 1-16.

Kirkland, R.L. 1972. Ecology of *Aroga websteri* Clarke in Curlew Valley, Utah-Idaho. Masters’ Thesis, Utah State University, Logan, Utah.

Kirkwood, C.W. 1961. A new Eupithecia from Arizona (Lepidoptera, Geometridae). Bulletin of the Southern California Academy of Sciences 60(1): 45-46.

Kirti, J.S. and N.S. Gill. 2007. Revival of genus *Patania* Moore and reporting of a new species menoni (Pyraustinae: Pyralidae: Lepidoptera). Journal of Entomological Research 31(3): 265-275.

Kitching, I.J., R. Rougerie, A. Zwick C.A Hamilton, R.A. St Laurent, S. Naumann, L. Ballesteros Mejia, and A.Y. Kawahara. 2018. A global checklist of the Bombycoidea (Insecta: Lepidoptera). Biodiversity Data Journal 6: e22236; 1-13. plus supplemental spreadsheet.

Klein, M.G. and H.C. Coppel. 1969. The pine chrysomelid, *Glyptoscelis pubescens*, in northwestern Wisconsin. Annals of the Entomological Society of America 62(1): 1-7.

Klein, W.H. and M.W. Minnoch. 1971. On the occurrence and biology of *Nepytia freemani* (Lepidoptera: Geometridae) in Utah. The Canadian Entomologist 103(1): 119-124.

Klem, C.C. and J. Zaspel. 2019. Pest injury guilds, Lepidoptera, and placing fruit-piercing moths in context: A review. Annals of the Entomological Society of America 112(5): 421-432.

Klimesch, J. 1961. Ordnung Lepidoptera. Teil 1. Tortricina. In: H. Franz, (ed.), Die Nordost - Alpen in Spiegel ihrer Landtierwelt 2: 543-619.

Klots, A.B. 1931a. New records of Microlepidoptera from New York. Journal of the New York Entomological Society 39(3): 291-293.

Klots, A.B. 1931b. Notes on some moths collected at Silver Lake, Chesham, New Hampshire. Psyche 38(1): 36-37.

Klots, A.B. 1932. New records of Lepidoptera from New York. Journal of the New York Entomological Society 40(3): 385-387.

Klots, A.B. 1936. New North American Microlepidoptera. American Museum Novitates 867: 1-6.

Klots, A.B. 1940. North American Crambus. I. The silvery-striped species of California (Pyralididae). Bulletin of the Southern California Academy of Sciences, Los Angeles 39: 53-70.

Klots, A.B. 1941. Two European Tortricidae (Lepdioptera) not hitherto recorded from North America. Bulletin of the Brooklyn Entomological Society 36: 126-127.

Klots, A. B. 1942a. North American *Crambus* (Pyralididae) II. New species. American Museum Novitates, New York 1191: 1-17.

Klots, A.B. 1951. A Field Guide to the Butterflies of North America, East of the Great Plains. Houghton, Mifflin and Company, Boston, MA.

Klots, A.B. 1967. Two new species of *Crambus* Fabricius from western North America (Lepidoptera: Pyralididae). Journal of the New York Entomological Society 75(3): 54-158.

Klots, A.B. 1968. The North American *Microcrambus* (Lepidoptera: Pyralididae). Journal of the New York Entomological Society 76(1): 9-21.

Klots, A.B. 1970. North American Crambinae - notes on tribe Chiloini and a revision of genera *Eoreuma* Ely and *Xubida* Schaus (Lepidoptera - Pyralidae). Journal of the New York Entomological Society 78(2): 100-120.

Klots, A.B. and C.F. dos Passos, 1981. Studies of North America *Erora* (Scudder) (Lepidoptera, Lycaenidae). Journal of the New York Entomological Sociey 89(4): 295-331.

Knowlton, G.F. 1953. Observations on *Celerio lineata*, the White-lined Sphinx, in Utah. Lepidopterists’ News, 7: 11-12.

Knowlton, C.B. 1967. A revision of the species of *Cisthene* known to occur north of the Mexican border (Lepidoptera: Arctiidae: Lithosiinae). Transactions of the American Entomological Society 93(1): 41-100.

Knudson, E.C. 1980. Current zone reports and planned activities, zone I: Texas. Southern Lepidopterists’ News 2(1): 3-4.

Knudson, E.C. 1981. Current zone reports, zone I: Texas. Southern Lepidopterists’ News 3(3): 15.

Knudson, E.C. 1982. Current zone reports, zone I: Texas. Southern Lepidopterists’ News 4(2): 9-10.

Knudson, E.C. 1984a. Current zone reports, zone I: Texas. Southern Lepidopterists’ News 6(2): 8.

Knudson, E.C. 1984b. Current zone reports, zone I: Texas. Southern Lepidopterists’ News 6(3): 16.

Knudson, E.C. 1986a. New species of Olethreutine moths (Tortricidae) from Texas and Louisiana. Journal of the Lepidopterists' Society 40(4): 322-326.

Knudson, E.C. 1986b. A new species of *Plataea* (Lepidoptera: Geometridae) from Texas. Proceedings of the Entomological Society of Washington 88(2): 351-353.

Knudson, E.C. 1987. Current zone reports, zone I: Texas. Southern Lepidopterists’ News 8(3&4): 19-20.

Knudson, E.C. 1991. Season Summary 1990. Zone 6 South Central: Oklahoma, Texas, Arkansas, Louisiana. News of the Lepidopterists’ Society 33(2): 26-28.

Knudson, E.C. 1994a. State field reports, Texas. Southern Lepidopterists’ News 16(4): 41-42.

Knudson, E.C. 1994b. Season Summary 1993. Zone 6 South Central: Oklahoma, Texas, Arkansas, Louisiana. News of the Lepidopterists’ Society 36(2): 35-36.

Knudson, E.C. 1995. State field reports, Texas state report, Texas. Southern Lepidopterists’ News 17(3): 24-25.

Knudson, E.C. 1999a. Reports of state coordinators, Texas. News of Southern Lepidopterists' Society 21(3): 55-56.

Knudson, E.C. 2000. Reports of state coordinators, Texas. Southern Lepidopterists’ News 22(2): 41-43.

Knudson, E.C. 2001. Reports of state coordinators, Texas. Southern Lepidopterists’ News 23(3): 57-58.

Knudson, E.C. 2003. Reports of state coordinators, Texas. Southern Lepidopterists’ News 25(3): 100.

Knudson, E.C. 2007. Reports of state coordinators, Texas. Southern Lepidopterists’ News 29(3): 116-119.

Knudson, E.C. 2008. Butterflies of Santa Ana National Wildlife Refuge. U.S. Fish & Wildlife Service pamphlet. October 2008.

Knudson, E.C. and C. Bordelon. 1999. Checklist of the Lepidoptera of Texas. Houston, Texas, 48 pp.

Knudson, E.C. and C. Bordelon. 2001. Noctuidae of Texas: species near The Stiriinae. Southern Lepidopterists’ News 23 (3): 47-49, plate B.

Knudson, E.C. and C. Bordelon. 2004a. Checklist of the Lepidoptera of Texas. Texas Lepidoptera Survey, Publ. 6, 1999. 2004 Edition.

Knudson, E.C. and C. Bordelon. 2004b. Ctenuchine Arctiids from Texas. Southern Lepidopterists’ News 26(4): 108-110.

Knudson, E.C. and C. Bordelon. 2008a. *Psilopleura polia minax* Draudt, 1915: (Arctiidae: Ctenuchinae) a new United States record from the Lower Rio Grande Valley, Texas. News of the Lepidopterists’ Society 50: 21, 25.

Knudson, E.C. and C. Bordelon. 2008b. *Purius superpulverea* (Dyar, 1925) Arctiidae; Phaegopterini, in Texas. Southern Entomologist’s News 30(4): 144.

Knudson, E. & C. Bordelon, 2010. Checklist of the Lepidoptera of Texas, Texas Lepidoptera Atlas. Texas Lepidoptera Survey Publication No. 6.

Knudson, E.C. and C. Bordelon. 2013. New U.S. records, state records, and other interesting moths from Texas. News of the Lepidopterists’ Society 55(1): 6-19.

Knudson, E.C. and C. Bordelon. 2018. Checklist of Texas Lepidoptera. Texas Lepidoptera Survey, 52 pp.

Knudson, E.C. and L. Koehn. 2009. The fruit piercing moths (*Gonodonta* Hubner, 1818, Noctuidae) in Texas, with report of a new record for the USA. News of the Lepidopterists’ Society 51(2): 72-74.

Knudson, E.C. and M. Rickard. 1984. Zone Reports: Zone 1 Texas. Southern Lepidopterists’ News 6(2): 8.

Knudson, E.C. and M. Rickard. 1986. Zone Reports: Zone 1 Texas. Southern Lepidopterists’ News 8(3&4): 19-20.

Knudson, E.C., C. Bordelon, and M.G. Pogue. 2003. A new species of *Schinia* Hübner (Lepidoptera: Noctuidae: Heliothinae) from Texas, Oklahoma, and Louisiana. Zootaxa 382: 1-7.

Knudson, E.C., L. Koehn, and M. Rickard. 2013. *Eulepidotis persimilis* (Guenee), (Erebidae: Eulepidoptinae): a new USA record, and *Eulepidotis dominicata* (Guenee): a confirmation of occurrence in the USA. News of the Lepidopterists’ Society 55(1): 16-17.

Koçak, A.Ö. 1986. More notes on the homonymy of the specific names of Lepidoptera. Priamus 4(1/2): 55-60.

Koçak, A.Ö. 2005. Nomenclatural note on the Nearctic Lepidoptera. Priamus 11(3): 59.

Koçak, A.Ö. and M. Kemal. 2008. Some nomenclatural notes on the Geometridae of the World (Lepidoptera). Centre for Entomological Studies, Miscellaneous Papers 138: 8-9.

Koehn, L.C. (ed.). 2012. Season Summery 2011. News of the Lepidopterists' Society 54(S1): 1-176.

Kohler, S. 2007. A description of a new subspecies of *Lycaena phlaeas* (Lycaenidae: Lycaeninae) from Montana, United States, with a comparative study of Old and New World populations. The Taxonomic Report 7(1): 1-20.

Kohler, S. and A.D. Warren. 2021. Review of Montana *Euphilotes* Mattoni, [1978], with Descriptions of New Taxa (Lepidoptera: Lycaenidae: Polyommatinae). The Taxonomic Report 9(4): 1-79.

Kollár, J. 2007. The harmful entomofauna of woody plants in Slovakia. Acta Entomologica Serbica 12(1): 67-79.

Kollár, J. and L. Bakay. 2015. The currant clearwing moth *Synanthedon tipuliformis* (Clerck, 1759) as a new pest for pawpaw (*Asimina triloba* L.) in Slovakia - short communication. Plant Protection Science 51: 153-155.

Komai, F. 1999. A taxonomic review of the genus *Grapholita* and allied genera (Lepidoptera: Tortricidae) in the Palaearctic region. Entomologica Sacndinavica 55(supplement): 1-226.

Kondla, N.G. 1995. Sulphur butterflies of the Colias alexandra complex in Alberta. Blue Jay 53(1): 15-27.
[truncated: 443,696 more chars]
