# Supplementary material for: ﻿Lepidoptera of North America, north of Mexico: an annotated list containing geographic ranges and host-plant records
Source: Zookeys. 2025 Nov 26;1261:101–13. doi: 10.3897/zookeys.1261.160796 (PMC12676573; doi:10.3897/zookeys.1261.160796)
Supplement: Supplementary material 3 — Species-level examinations to catagorize host plant records into proper genus [file zookeys-1261-101_article-160796__-s003.pdf]

APPENDIX 1. Species-level examinations to catagorize records into proper genus.

Genera examined to species. Taxonomic revisions reclassified these genera. Species-level examination of records was required. Searches were confined to host plant records. BONAP (Kartesz 2015) used as a primary source; KEW (2025) used for other records. Host plant records without a species name remain in that genus or are designated unresolved

Family	Examined Genus	Reassignment options
Fabaceae	*Acacia*	possible resolutions: A*caciella Mariosousa Mimosa Senegalia Vachellia* & *Acacia*
Fabaceae	*Acaciella*	some species formerly *Acacia*
Poaceae	*Achnatherum*	possible resolutions: *Amelichloa Jarava Pappostipa Stipa* & *Achnatherum*
Poaceae	*Agropyron*	possible resolutions: *Elymus Eremopyrum Leymus Pseudelymus Pseudoroegneria Thinopyrum* & *Agropyron*
Poaceae	*Agrostis*	possible resolutions: *Apera Bromidium Chloris Gastridium Lachnagrostis Mibora Muhlenbergia Phippsia Piptatherum Podagrostis Polypogon Sporobolus Zoysia* & *Agrostis*
Fabaceae	*Albizia*	possible resolutions: F*alcataria Paraserianthes Samanea* & *Albizia*
Ranunculaceae	*Anemone*	possible resolutions: *Hepatica Pulsatilla Thalictrum* & *Anemone*
Plantaginaceae	*Antirrhinum*	possible resolutions: *Collinsia Epixiphium Howelliella Kickxia Maurandella Misopates Neogaerrhinum Pseudorontium Sairocarpus* & *Antirrhinum*
Apiaceae	*Apium*	possible resolutions: *Cyclospermum Helosciadium Petroselinum Spermolepis* & *Apium*
Brassicaceae	*Arabis*	possible resolutions: *Arabidopsis Boechera Cardamine Crucihimalaya Pennellia Phravenia Planodes Sandbergia Sibara Sibara Streptanthus Turritis Yosemitea* & *Arabis*
Arecaceae	*Arecastrum*	possible resolutions: *Syagrus*
Aristolochiaceae	*Aristolochia*	possible resolutions: *Endodeca Isotrema* & *Aristolochia*
Rosaceae	*Aronia*	some species formerly in: *Photinia* & *Pyrus*
Asteraceae	*Artemisia*	possible resolutions: *Centipeda Delwiensia Matricaria Picrothamnus* & *Artemisia*
Asteraceae	*Aster*	possible resolutions: *Callistephus Canadanthus Chaetopappa Chloracantha Dieteria Doellingeria Erigeron Eucephalus Eurybia Herrickia Ionactis Leucosyris Machaeranthera Neonesomia Oclemena Oreostemma Pallenis Psilactis Sericocarpus Solidago Symphyotrichum Tetramolopium Townsendia Tripolium Xanthisma Xylorhiza* & *Aster*
Amaranthaceae	*Atriplex*	possible resolutions: *Extriplex Grayia Proatriplex Stutzia* & *Atriplex*
Asteraceae	*Bactris*	possible resolutions: *Aiphanes minima in* BONAP; Astrocaryum *Aiphanes* Astrocaryum Desmoncus & *Bactris* in KEW
Asteraceae	*Bahia*	possible resolutions: *Amauriopsis Picradeniopsis Platyschkuhria Schkuhria* & *Bahia*
Fabaceae	*Bauhinia*	possible resolutions: *Phanera* & *Bauhinia*

Family	Examined Genus	Reassignment options
Nyctaginaceae	*Boerhavia*	possible resolutions: *Anulocaulis Commicarpus Cyphomeris* & *Boerhavia*
Cyperaceae	*Bolboschoenus*	possible resolutions: *Schoenoplectus* in BONAP
Araliaceae	*Brassaia*	possible resolutions: *Schefflera* in BONAP; but *Heptapleurum* for South Asia in KEW
Asteraceae	*Cacalia*	possible resolutions: *Arnoglossum Cacaliopsis Emilia Mikania Parasenecio Porophyllum Psacalium Rugelia* & *Senecio*
Onagraceae	*Camissonia*	possible resolutions: *Camissoniopsis Chylismia Chylismiella Eremothera Eulobus Oenothera Neoholmgrenia Taraxia Tetrapteron* & *Camissonia*
Capparaceae	*Capparis*	possible resolutions: *Atamisquea Cynophalla Quadrella* & *Capparis*
Asteraceae	*Carduus*	possible resolutions: C*irsium Silybum* & *Carduus*
Fabaceae	*Cassia*	possible resolutions: Chamaecrista Senna & Cassia
Poaceae	*Cenchrus*	possible resolutions: *Hackelochloa Pennisetum Tragus* & *Cenchrus*
Asteraceae	*Centaurea*	possible resolutions: *Amberboa Mantisalca Plectocephalus Rhaponticum Volutaria* & *Centaurea*
Gentianaceae	*Centaurium*	possible resolutions: *Centaurium Gyrandra Schenkia Zeltnera* & *Centaurium*
Amaranthaceae	*Chenopodium*	possible resolutions: *Blitum Dysphania Lipandra Oxybasis* & *Chenopodium*
Poaceae	*Chloris*	possible resolutions: *Bouteloua Disakisperma Enteropogon Eustachys Leptochloa* & *Chloris*
Asteraceae	*Chrysoma*	possible resolutions: *Ericameria* & *Chrysoma*
Asteraceae	*Chrysopsis*	possible resolutions: *Arnica Bradburia Chrysopsis Eucephalus Heterotheca Ionactis Pityopsis* & *Chrysopsis*
Asteraceae	*Chrysothamnus*	possible resolutions: *Cuniculotinus Ericameria Lorandersonia* & *Chrysothamnus*
Cleomaceae	*Cleome*	possible resolutions: *Arivela Cleoserrat Gynandropsis Hemiscola Peritoma Polanisia Stanleya Tarenaya* & *Cleome*
Asteraceae	*Conyza*	possible resolutions: *Cyanthillium Erigeron Laennecia Neurolaena Pluchea Sericocarpus* & *Symphyotrichum*
Asteraceae	*Coreopsis*	possible resolutions: *Bidens Cosmos Leptosyne* & *Coreopsis*
Fabaceae	*Coronilla*	possible resolutions: *Securigera* & *Coronilla*
Fumariaceae	*Corydalis*	possible resolutions: *Capnoides Pseudofumaria* & *Corydalis*
Boraginaceae	*Cryptantha*	possible resolutions: *Eremocarya Greeneocharis Johnstonella Oreocarya* & *Cryptantha*
Cupressaceae	*Cupressus*	possible resolutions: *Callitropsis Chamaecyparis Hesperocyparis* & *Cupressus*
Fabaceae	*Cytisus*	possible resolutions: *Argyrocytisus Chamaecytisus Genista* & *Cytisus*
Fabaceae	*Dalea*	possible resolutions: *Psorothamnus* & *Dalea*
Asteraceae	*Delwiensia*	Delwiensia pattersonii, formerly *Artemisia*
Fabaceae	*Derris*	possible resolutions: *Millettia Paraderris* & *Derris*

Family	Examined Genus	Reassignment options
Fabaceae	*Desmodium*	possible resolutions: *Hylodesmum Lespedeza* & *Desmodium*
Poaceae	*Dinebra*	possible resolutions: *Bouteloua Enteropogon* & *Dinebra*
Athyriaceae	*Diplazium*	possible resolutions: *Homalosorus* & *Diplazium*
Liliaceae	*Disporum*	possible resolutions: *Prosartes* in North America & *Disporum* is an southeastern Asian genus
Fabaceae	*Dolichos*	possible resolutions: *Enteropogon Lablab Rhynchosia* & *Dolichos*
Alismataceae	*Echinodorus*	possible resolutions: *Baldellia Helanthium* & *Echinodorus*
Apocynaceae	*Echites*	possible resolutions: *Allotoonia Thyrsanthella* & *Echites*
Poaceae	*Elymus*	possible resolutions: *×Elyleymus Leymus Pascopyrum ×Pseudelymus Pseudoroegneria Taeniatherum Thinopyrum* & *Elymus*
Poaceae	*Elytrigia*	possible resolutions: *Elymus Pascopyrum Pseudoroegneria Thinopyrum* & *Elytrigia*
Onagraceae	*Epilobium*	possible resolutions: *Chamaenerion* & *Epilobium*
Asteraceae	*Erechtites*	possible resolutions: *Senecio*
Euphorbiaceae	*Eremocarpus*	possible resolutions: *Croton setiger*
Geraniaceae	*Erodium*	possible resolutions: *California* & *Erodium*
Asteraceae	*Eupatorium*	possible resolutions: *Ageratina Ayapana Brickellia Brickelliastrum Chromolaena Condylidium Conoclinium Critonia Eutrochium Fleischmannia Flyriella Hebeclinium Koanophyllon Tamaulipa* & *Eupatorium*
Asteraceae	*Eutrochium*	some species formerly in: *Eupatorium*
Asteraceae	*Euthamia*	some species formerly in: *Solidago*
Poaceae	*Festuca*	possible resolutions: *Brachypodium Bromus Danthonia Dinebra Eremopoa Glyceria Leucopoa Rostraria Schedonorus Schismus Vulpia* & *Festuca*
Onagraceae	*Gaura*	possible resolutions: *Clarkia Eremothera* & *Oenothera*
Polemoniaceae	*Gilia*	possible resolutions: *Aliciella Allophyllum Eriastrum Giliastrum Gymnosteris Ipomopsis Linanthus Loeseliastrum Microgilia Microsteris Navarretia Polemonium Saltugilia* & *Gilia*
Acanthaceae	*Gerardia*	possible resolutions: *Agalinis Aureolaria* & *Stenandrium*
Asteraceae	*Gnaphalium*	possible resolutions: *Antennaria Euchiton Filago Gamochaeta Helichrysum Logfia Omalotheca Pseudognaphalium Pterocaulon* & *Gnaphalium*
Asteraceae	*Grindelia*	possible resolutions: *Chrysothamnus Prionopsis Xanthocephalum* & *Grindelia*
Asteraceae	*Gutierrezia*	possible resolutions: *Amphiachyris Thurovia Xanthocephalum* & *Gutierrezia*
Brassicaceae	*Halimolobos*	possible resolutions: *Crucihimalaya Sandbergia* & *Halimolobos*
Bignoniaceae	*Handroanthus*	some species formerly in: *Tabebuia*

Family	Examined Genus	Reassignment options
Asteraceae	*Haplopappus*	possible resolutions: *Chrysothamnus Columbiadoria Croptilon Ericameria Eurybia Gundlachia Hazardia Isocoma Lorandersonia Nestotus Oonopsis Oreochrysum Oreostemma Prionopsis Pyrrocoma Rayjacksonia Stenotus Toiyabea Tonestus* & *Xanthisma*
Apiaceae	*Harperella*	some species formerly in: *Ptilimnium*
Cistaceae	*Helianthemum*	possible resolutions: *Crocanthemum Tuberaria* & *Helianthemum*
Brassicaceae	*Hesperidanthus*	some species formerly in: *Schoenocrambe* & *Thelypodiopsis*
Asteraceae	*Heterotheca*	possible resolutions: *Chrysopsis Eucephalus Pityopsis* & *Heterotheca*
Diplaziopsidaceae	*Homalosorus*	some species formerly in: *Diplazium*
Amaryllidaceae	*Hymenocallis*	some species formerly in: *Pancratium*
Poaceae	*Hystrix*	possible resolutions: *Elymus* & *Leymus*
Polemoniaceae	*Ipomopsis*	possible resolutions: *Aliciella Loeseliastrum Microgilia* & *Ipomopsis*
Asteraceae	*Iva*	possible resolutions: *Cyclachaena Euphrosyne Hedosyne* & *Iva*
Onagraceae	*Jussiaea*	possible resolutions: *Ludwigia*
Polemoniaceae	*Leptodactylon*	some alternately: *Leptosiphon* & *Linanthus*
Fabaceae	*Ladeania*	Two species: *Ladeania juncea* , *Ladeania lanceolata*
Fabaceae	*Lespedeza*	possible resolutions: *Kummerowia Rhynchosia* & *Lespedeza*
Poaceae	*Leptochloa*	possible resolutions: *Dinebra* & *Leptochloa*
Asteraceae	*Lessingia*	possible resolutions: *Benitoa Corethrogyne* & *Lessingia*
Fabaceae	*Leucaena*	possible resolutions: *Acaciella Senegalia Lysiloma Schleinitzia* & *Leucaena*
Ericaceae	*Leucothoe*	possible resolutions: *Agarista Eubotrys Senegalia* & *Leucothoe*
Plantaginaceae	*Linaria*	possible resolutions: *Linaria Asclepias Cymbalaria Kickxia* & *Nuttallanthus*
Verbenaceae	*Lippia*	possible resolutions: *Aloysia Lantana Phyla* & *Lippia*
Poaceae	*Lolium*	possible resolutions: *Schedonorus* & *Lolium*
Fabaceae	*Lotus*	possible resolutions: *Acmispon Hosackia Nymphaea* & *Lotus*
Lycopodiaceae	*Lycopodium*	possible resolutions: *Dendrolycopodium Diphasiastrum Huperzia Lycopodiella Palhinhaea Palhinhaea Pseudolycopodiella Pseudolycopodiella Selaginella Spinulum* & *Lycopodium*
Asteraceae	*Machaeranthera*	possible resolutions: *Dieteria Herrickia Leucosyris Psilactis Rayjacksonia Xanthisma Xylorhiza* & *Machaeranthera*
Orchidaceae	*Maxillaria*	possible resolutions: *Heterotaxis* & *Maxillaria*
Asteraceae	*Microseris*	possible resolutions: *Nothocalaiss Stebbinsoseris Uropappus* & *Microseris*
Saxifragaceae	*Mitella*	possible resolutions: *Mitellastra Ozomelis Pectiantia* & *Mitella*
Myricaceae	*Myrica*	possible resolutions: *Comptonia Morella* & *Myrica*
Hydrophyllaceae	*Nemophila*	all species: *Nemophila*
Cactaceae	*Neolloydia*	possible resolutions: *Echinomastus* & *Neolloydia*
Orobanchaceae	*Orobanche*	possible resolutions: *Phelipanche Conopholis* & *Orobanche*
Orobanchaceae	*Orthocarpus*	possible resolutions: *Castilleja Orthocarpus Plagiobothrys* & *Triphysaria*

Family	Examined Genus	Reassignment options
Poaceae	*Oryzopsis*	possible resolutions: *Achnatherum Patis Piptatheropsis Piptatherum Piptochaetium Ptilagrostis* & *Oryzopsis*
Hyacinthaceae	*Othocallis*	possible resolutions: *Scilla* & *Othocallis*
Amaryllidaceae	*Pancratium*	possible resolutions: *Hymenocallis* & *Pancratium*
Poaceae	*Panicum*	possible resolutions: *Coleataenia Dichanthelium Digitaria Oplismenus Sacciolepis Setaria Steinchisma Urochloa Zuloagaea Panicum* & others
Fabaceae	*Pediomelum*	some specie formerly *Psoralea* & *Psoralidium*
Poaceae	*Pennisetum*	possible resolutions: *Cenchrus Setaria* & *Pennisetum*
Plantaginaceae	*Penstemon*	possible resolutions: *Keckiella* & *Penstemon*
Asteraceae	*Petradoria*	possible resolutions: *Cuniculotinus* & *Petradoria*
Fabaceae	*Phanera*	some species formerly in: *Bauhinia*
Orobanchaceae	*Phelipanche*	possible resolutions: *Orobanche* & *Phelipanche*
Rosaceae	*Photinia*	possible resolutions: *Aronia Heteromeles* & *Photinia*
Polygalaceae	*Polygala*	possible resolutions: *Asemeia Hebecarpa Polygaloides Rhinotropis* & *Polygala*
Polygonaceae	*Polygonum*	possible resolutions: *Aconogonon Bistorta Coccoloba Fagopyrum Fallopia Muehlenbeckia Persicaria Reynoutria* & *Polygonum*
Dryopteridaceae	*Polystichum*	possible resolutions: *Cyrtomium falcatum* & *Rumohra adiantiformis* or *Polystichum*
Asteraceae	*Prenanthes*	possible resolutions: *Askellia Nabalus* & *Prenanthes*
Primulaceae	*Primula*	possible resolutions: *Dodecatheon* & *Primula*
Liliaceae	*Prosartes*	possible resolutions: *Disporum Streptopus* & *Prosartes*
Zygophyllaceae	*Porlieria*	possible resolutions: *Guaiacum* & *Porlieria*
Rosaceae	*Potentilla*	possible resolutions: *Comarum Dasiphora Drymocallis Horkelia Ivesia Sibbaldia* & *Potentilla*
Fabaceae	*Psoralea*	possible resolutions: *Bituminaria Cullen Hoita Ladeania Orbexilum Otholobium Pediomelum Rupertia* & *Psoralea*
Fabaceae	*Psoralidium*	possible resolutions: *Ladeania* & *Pediomelum*
Apiaceae	*Ptilimnium*	possible resolutions: *Harperella* & *Ptilimnium*
Ericaceae	*Pyrola*	possible resolutions: *Moneses Orthilia* & *Pyrola*
Capparaceae	*Quadrella*	possible resolutions: *Quadrella incana* & *Quadrella jamaicensis* in BONAP; but *Morisonia* in KEW
Fagaceae	*Quercus*	*Quercus* is a focus species
Rhamnaceae	*Rhamnus*	possible resolutions: *Ceanothus Frangula Krugiodendron Paliurus Sageretia* & *Rhamnus*
Euphorbiaceae	*Sapium*	possible resolutions: *Sebastiania Triadica* & *Sapium*
Euphorbiaceae	*Savia*	possible resolutions: *Phyllanthopsis Heterosavia* & *Savia*
Saxifragaceae	*Saxifraga*	possible resolutions: *Boykinia Cascadia Darmera Leptarrhena Luetkea Micranthes Saxifragopsis Sullivantia Telesonix* & *Saxifraga*

Family	Examined Genus	Reassignment options
Poaceae	*Schedololium*	resolutions: ×*Schedololium holmbergii* [=F*estulolium holmbergii*] & ×*Schedololium loliaceum* [=×*Festulolium loliaceum*] in BONAP; BUT *Lolium × holmbergii* [=*Festulolium holmbergii*] & *Lolium × elongatum* [=×*Festulolium loliaceum]* in KEW
Brassicaceae	*Schoenocrambe*	possible resolutions: Hesperidanthus & Sisymbrium
Liliaceae	*Scilla*	possible resolutions: *Hyacinthoides Othocallis* & *Scilla*
Cyperaceae	*Scirpus*	possible resolutions: *Blysmopsis Bulbostylis Cyperus Eleocharis Eriophorum Fimbristylis Fuirena Isolepis Lipocarpha Oxycaryum Rhynchospora Schoenoplectiella Scirpoides Scleria Trichophorum* & *Scirpus*
Crassulaceae	*Sedum*	possible resolutions: *Aizopsis Diamorpha Hylotelephium Lenophyllum Phedimus Rhodiola* & *Sedum*
Asteraceae	*Senecio*	possible resolutions: *Arnoglossum Barkleyanthus Delairea Jacobaea Ligularia Malacothrix Packera Tephroser Tetramolopium* & *Senecio*
Apiaceae	*Selinum*	possible resolutions: *Cnidium Conioselinum* or *Selinum*
Fabaceae	*Senegalia*	resolutions not North American from KEW: *Leucaena Mariosousa Parasenegalia* & others
Asteraceae	*Serratula*	possible resolutions: *Cirsium arvense* in BONAP; but also *Klasea Jurinea Lucilia Vernonia Serratula* & others in KEW
Caryophyllaceae	*Silene*	possible resolutions: *Atocion Eudianthe Saponaria* & *Silene*
Fabaceae	*Sophora*	possible resolutions: *Dermatophyllum Styphnolobium Cladrastis* & *Sophora*
Onagraceae	*Stenosiphon*	possible resolutions: *Oenothera*
Poaceae	*Stipa*	possible resolutions: *Achnatherum Amelichloa Hesperostipa Jarava Nassella Pappostipa Piptochaetium Ptilagrostis Trachypogon* & *Stipa*
Bignoniaceae	*Tabebuia*	possible resolutions: *Handroanthus Roseodendron* & *Tabebuia*
Bignoniaceae	*Tecoma*	possible resolutions: *Campsis Podranea* & *Tecoma*
Brassicaceae	*Thelypodiopsis*	possible resolutions: *Hesperidanthus* & *Thelypodiopsis*
Brassicaceae	*Thelypodium*	possible resolutions: *Caulanthus Iodanthus Pennellia Sibar* a & *Thelypodium*
Brassicaceae	*Thlaspi*	possible resolutions: *Capsella Noccaea Teesdalia* & *Thlaspi*
Bromeliaceae	*Tillandsia*	possible resolutions: *Catopsis Vriesea* & *Tillandsia*
Poaceae	*Trisetum*	possible resolutions: *Graphephorum Sphenopholis Schizachne* & *Trisetum*
Poaceae	*Uniola*	possible resolutions: *Chasmanthium Distichlis Leptochloopsis* & *Uniola*
Fabaceae	*Vachellia*	some species formerly in: *Acacia*
Asteraceae	*Verbesina*	possible resolutions: *Adenostemma Eclipta Lasianthaea Synedrella* & *Verbesina*
Asteraceae	*Viguiera*	possible resolutions: *Bahiopsis Heliomeris Zaluzania* & *Viguiera*
Apocynaceae	*Vincetoxicum*	possible resolutions: *Cynanchum Gonolobus Matelea* m, but *Vincetoxicum* is not a New World species
Santalaceae	*Viscum*	possible resolutions: *Phoradendron* & *Viscum*

Family	Examined Genus	Reassignment options
Asteraceae	*Wedelia*	possible resolutions: *Allionia Pascalia Sphagneticola* & *Wedelia*
Asteraceae	*Xanthocephalum*	possible resolutions: *Amphiachyris Gutierrezia Gymnosperma* & *Xanthocephalum*
Asteraceae	*Xylorhiza*	some species formerly in: *Machaeranthera*
Agavaceae	*Yucca*	possible resolutions: *Hesperoyucca* & *Yucca*
Onagraceae	*Zauschneria*	resolution: *Epilobium*
Melanthiaceae	*Zigadenus*	possible resolutions: *Amianthium Anticlea Stenanthium Toxicoscordion* & *Zigadenus*

Clarification of some genera.

Antirrhinum : Only *Antirrhinum majus* retains the genus name in North America, other species assigned to 9 different genera, "*Antirrhinum* sp." retained in *Antirrhinum*.
Aster : The genus *Aster* is now primarily an Old World name. North American records for *Aster* sp. are tentatively assigned to *Symphyotrichum* sp. It is possible to filter out & reject this assignment.
Haplopappus :The genus *Haplopappus* is now a South American name. North American records for *Haplopappus* sp. are tentatively assigned to *Isocoma* sp. It is possible to filter out & reject this assignment.
Cupressus : North American records for *Cupressus* sp. are assigned to *Hesperocyparis*.
Pyrus : The genus *Pyrus* is valid but it was also the former genus of *Aronia melanocarpa* [=*Pyrus melanocarpa*], *Sorbus american* [=*Pyrus americana*], *Cydonia oblonga* [=*Pyrus cydonia*], *Malus sylvestris[=Pyrus malus*], ×*Sorbaronia hybrida* [=*Pyrus ×hybrida*], and other *Malus*, *Aronia* & *Sorbus* species. Robinson et al (2002) and others have corrected known records, but many "*Pyrus* sp." records are left and may be in doubt.
Rhus : *Rhus* was the former genus name of poison ivy (*Toxicodendron*). Old *Rhus* sp. records may refer to another genus.

Redirecting for unresolved species. The following genus records are designated [unresolved] in host plant column and [unassigned] in dicreet host plant column. The purpose is to facilitate excluding imprecise records. The unresolved record may or may not exclude genus as listed.

HOST_GENERA (Column U)	HOST_GENUS_DISCREET_SEARCH (Column V)
Acacia[unresolved]	Acacia.[unassigned]
Arabis[unresolved]	Arabis.[unassigned]
Aster[unresolved]	Symphyotrichum.[unassignedAster] or [unassignedAster] if host plant Symphyotrichum is already listed
Bombax[unresolved]	Bombax.[unassigned]
Chenopodium[unresolved]	Chenopodium.[unassigned]
Coleus[unresolved]	Coleus.[unassigned]
Comarum[unresolved]	Comarum.[unassigned]
Dolichos[unresolved]	Dolichos.[unassigned]
Elytrigia[unresolved]	Elytrigia.[unassigned].
Eupatorium[unresolved]	Eupatorium.[unassigned]
Gnaphalium[unresolved]	Gnaphalium.[unassigned]
Haplopappus[unresolved]	Isocoma.[unassignedHaplopappus]

HOST_GENERA (Column U)	HOST_GENUS_DISCREET_SEARCH (Column V)
Hemizonia[unresolved]	Hemizonia.[unassigned]
Leptodactylon[unresolved]	Leptodactylon.[unassigned]
Lonicera[unresolved]	Lonicera.[unassigned]
Lotus[unresolved]	Lotus.[unassigned]
Myrica[unresolved]	Myrica.[unassigned]
Oxypolis[unresolved]	Oxypolis.[unassigned]
Polygonum[unresolved]	Polygonum.[unassigned]
Potentilla[unresolved]	Potentilla.[unassigned]
Psoralea[unresolved]	Psoralea.[unassigned]
Senecio[unresolved]	Senecio.[unassigned]
Viscum[unresolved]	Viscum.[unassigned]
